# Supplementary material for: Higher-generation type III-B rotaxane dendrimers with controlling particle size in three-dimensional molecular switching
Source: Nat Commun. 2018 Feb 5;9:497. doi: 10.1038/s41467-018-02902-z (PMC5799186; doi:10.1038/s41467-018-02902-z)
Supplement: Supplementary file 1 — Supplementary Information [file 41467_2018_2902_MOESM1_ESM.pdf]

Higher-Generation Type III-B Rotaxane Dendrimers with Controlling Particle Size in Three-Dimensional Molecular Switching - *Kwan et al.*

Supplementary Methods

General Methods

Unless otherwise stated, all the chemicals were purchased from Sigma-Aldrich, Acros, IL, Fluka, J&K, and Dieckmann. The AR grade solvents were purchased commercially and used without further purification. All the reactions were conducted under anhydrous, high purity nitrogen atmospheric protection. Thin-layer chromatography (TLC Silica gel 60, Merck) was performed on aluminum plate. The plates were observed under 254 nm UV light, visualized by staining with phosphomolybdic acid (PMA) solution or ninhydrin solution with general heating. Column chromatography was carried out on silica gel SiO_2 60F (Merck 9385, 0.040–0.063 mm, Germany) as the stationary phase. ^{1}H NMR, ^{13}C NMR, ^{31}P NMR, Nuclear Overhauser Effect Spectroscopy (NOESY) spectra were recorded at 298K with Bruker Avance-III (^{1}H: 400 MHz, ^{13}C: 101 MHz, ^{31}P: 162 MHz). Diffusion-Ordered Spectroscopy (DOSY) was recorded at 298K with Bruker Avance-III HD 500 MHz spectrometer. Chemical shifts of solvent were calibrated to the solvent residue reference peak, for ^{1}H NMR ($CDCl_3$ = 7.26 ppm, CD_2Cl_2 = 5.32 ppm, CD_3CN = 1.94 ppm, DMSO-d_6 = 2.50 ppm), for ^{13}C NMR ($CDCl_3$ = 77.16 ppm, CD_2Cl_2 = 54.00 ppm, CD_3CN = 1.39 ppm, DMSO-d_6 = 39.51 ppm). Coupling constants (J) are reported in hertz (Hz). Standard abbreviations indicate the multiplicity of the peaks (s = singlet, d = doublet, t = triplet, q = quartet, quin = quintet, m = multiplet, br = broad, dd = doublet of doublets, ddd = doublet of doublet of doublets *etc.*). High-resolution mass spectrometry (HRMS) was carried out with Bruker Autoflex spectrometer (MALDI-TOF), and Thermofinnigan MAT 95 XL spectrometer (ESI-MS) with quadrupole ion trap (QIT) and fourier-transform ion cyclotron resonance (FTICR). Melting points of the compounds were recorded with Electrothermal 9100 digital melting point apparatus. Dynamic light scattering (DLS) was performed by Beckman Coulter DelsaMax CORE. Atomic force microscopy (AFM) was performed with scanning probe microscope (SPM) Veeco MultiMode IVa.

Synthesis of DB24C8-OSu

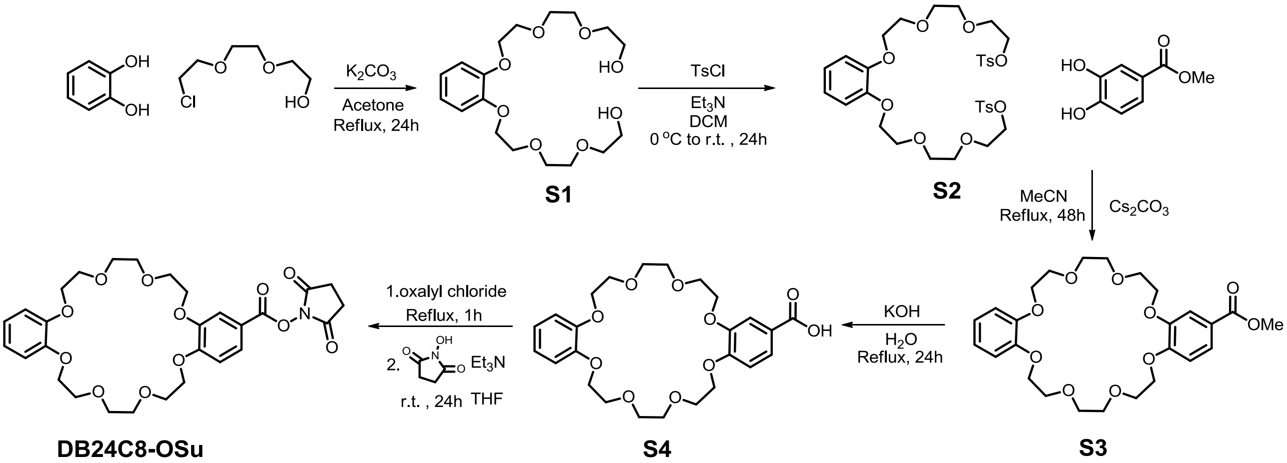

Supplementary Figure 1: Synthesis of DB24C8-OSu.

DB24C8-OSu was synthesized according to literature with modifications.[1]

Preparation of **S1**: 2-(2-(2-chloroethoxy)ethoxy)ethanol (20 g, 120 mmol) was dissolved in 100 mL acetone, follow with the addition of catechol (6.5 g, 0.06 mol) and K_2CO_3 (33 g, 240 mmol) and reflux overnight. The reaction mixture was then filtered to remove the K_2CO_3 before concentrated under vacuum. The residue was redissolved in $CHCl_3$ (150 mL) and extracted with 1M HCl (50 mL), then 1M NaOH (50 mL). The combined organic layers were dried over anhydrous $MgSO_4$ and concentrated under vacuum to give a pale yellow liquid, (15 g, 67%) which was pure enough without further purification. ^{1}H NMR (400 MHz, $CDCl_3$) δ 6.90 (s, 4H), 4.20 – 4.13 (m, 4H), 3.90 – 3.84 (m, 4H), 3.77 – 3.65 (m, 12H), 3.62 – 3.56 (m, 4H).

Preparation of **S2**: **S1** (15 g, 40 mmol) was dissolved in 100 mL DCM, follow with the addition of Et_3N (22 mL, 160 mmol), cool to 0 ^{o}C and stirred for 10 minutes. TsCl (16 g, 84 mmol) was dissolved in 30 mL DCM and added to the reaction mixture with catalytic amount of DMAP. The reaction mixture was then stirred overnight at room temperature. The reaction mixture was extracted with acidic aqueous solution. The combined organic layers were dried over anhydrous $MgSO_4$ and concentrated under vacuum. The yellow liquid was purified by column chromatography (SiO_2; Hexane/EtOAc 1:1.5) yielding a pale yellow thick oil.(19 g, 70%) ^{1}H NMR (400 MHz, $CDCl_3$) δ 7.79 (d, J = 8.3 Hz, 4H), 7.32 (d, J = 8.0 Hz, 4H), 6.91 (s, 4H), 4.14 (q, J = 5.1 Hz, 8H), 3.86 – 3.79 (m, 4H), 3.70 – 3.65 (m, 8H), 3.60 (dd, J = 5.7, 3.1 Hz, 4H), 2.42 (s, 6H).

Preparation of **S3**: **S2** (19 g, 28 mmol), Cs_2CO_3 (9.1 g, 28 mmol), K_2CO_3 (19 g 140

mmol) were dissolved in 300 mL MeCN. Methyl 3,4-dihydroxybenzoate (4.7 g, 0.028 mol) was dissolved in 100 mL MeCN and added to the reaction mixture dropwise through dropping funnel. The reaction mixture was refluxed for 48 h. The reaction mixture was then filtered before concentrated under vacuum. The residue was redissolved in $CHCl_3$ (200 mL) and extracted with 1 M HCl (50 mL). The combined organic layers were dried over anhydrous $MgSO_4$ and concentrated under vacuum. The yellow liquid was purified by column chromatography (SiO_2; EtOAc) yielding a white solid (9.5 g, 67%). ^1H NMR (400 MHz, $CDCl_3$) δ 7.64 (dd, *J* = 8.4, 2.0 Hz, 1H), 7.51 (d, *J* = 2.0 Hz, 1H), 6.92 – 6.81 (m, 5H), 4.21 – 4.17 (m, 4H), 4.16 – 4.13 (m, 4H), 3.93 (p, *J* = 4.3 Hz, 8H), 3.87 (s, 3H), 3.84 (d, *J* = 2.7 Hz, 8H).

Preparation of **S4**: **S3** (7 g, 14 mmol) was dissolved in 180 mL water containing KOH (8 g, 14 mmol). The reaction mixture was refluxed overnight to give a clear solution. The reaction mixture was cooled to low temperature, and conc. HCl was added until no further precipitation. The white precipitate was then filtered, washed with water (50 mL x 3), air dried overnight to give a white solid in quantitative yield. ^1H NMR (400 MHz, DMSO-d_6) δ 7.52 (dd, J = 8.4, 2.0 Hz, 1H), 7.41 (d, J = 2.0 Hz, 1H), 7.01 (d, J = 8.5 Hz, 1H), 6.92 (dd, J = 6.0, 3.6 Hz, 2H), 6.88 – 6.81 (m, 2H), 4.15 – 4.10 (m, 2H), 4.08 (dd, J = 5.2, 3.4 Hz, 2H), 4.06 – 4.01 (m, 4H), 3.75 (ddd, J = 8.7, 6.6, 3.8 Hz, 8H), 3.65 (s, 8H).

Preparation of DB24C8-OSu: **S4** (4.5 g, was 9 mmol) was dissolved in excess oxayl chloride (20 mL) with one drop of DMF. The orange solution was clear and stirred for 2.5 h. Excess oxayl chloride was removed by distillation to give a white solid. The white solid was redissolved in THF (160 mL) containing *N*-Hydroxysuccinimide (5 g, 43 mmol). Et_3N (6 mL, 43 mmol) was added finally to give a pale yellow turbid solution and stirred overnight. The reaction mixture was filtered and concentrated under vacuum. The residue was redissolved in $CHCl_3$ (100 mL) and extracted with 1 M HCl (50 mL). The combined organic layers were dried over anhydrous $MgSO_4$ and concentrated under vacuum to give a pale yellow solid. The pale yellow solid was recrystallized in hot EtOH yielding a white solid (5.2 g, 98%). ^1H NMR (400 MHz, $CDCl_3$) δ 7.77 (dd, *J* = 8.5, 2.0 Hz, 1H), 7.55 (d, *J* = 2.1 Hz, 1H), 6.94 – 6.82 (m, 5H), 4.24 – 4.12 (m, 8H), 3.98 – 3.90 (m, 8H), 3.84 (d, *J* = 5.8 Hz, 8H), 2.90 (s, 4H).

Synthesis of Thread H·PF₆

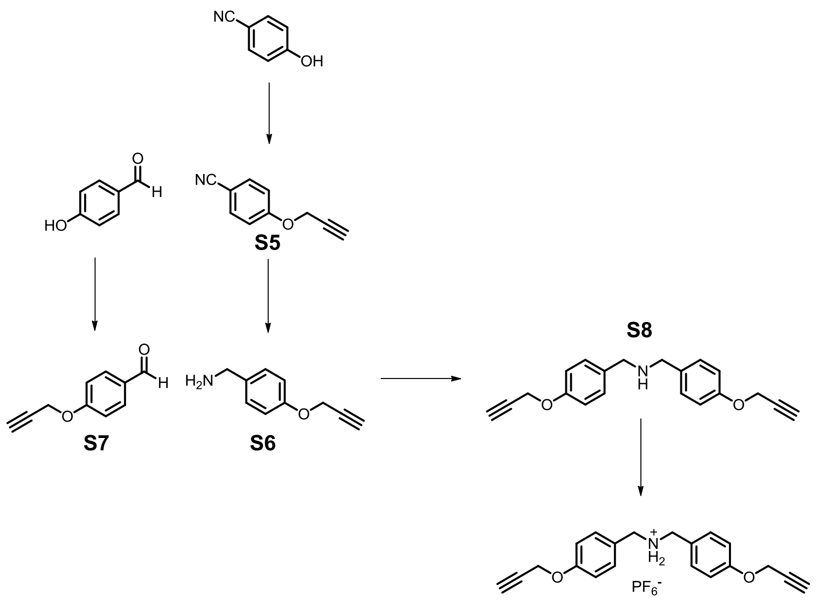

Supplementary Figure 2: Synthesis of Thread H·PF_6.

Thread H·PF_6 was synthesized according to literature with modifications.[2]

Preparation of **S5**: 4-cyanophenol (8 g, 60 mmol) was dissolved in 70 mL acetone. K_2CO_3 (18 g, 130 mmol) and propargyl bromide (80 wt. % in toluene) (15 mL, 120 mmol) was added. The reaction mixture was refluxed overnight. The reaction mixture was then filtered to remove the K_2CO_3 before concentrated under vacuum. The residue was redissolved in $CHCl_3$ (60 mL) and extracted with water. The combined organic layers were dried over anhydrous $MgSO_4$ and concentrated under vacuum to give a pale yellow solid. The pale yellow solid was recrystallized in hot EtOH yielding a pale yellow needle crystal (6.5 g, 69%). ^{1}H NMR (400 MHz, $CDCl_3$) δ 7.64 – 7.58 (m, 2H), 7.07 – 7.01 (m, 2H), 4.75 (d, J = 2.4 Hz, 2H), 2.57 (t, J = 2.4 Hz, 1H).

Preparation of **S6**: **S5** (6.5 g, 40 mmol) was dissolved in 200 mL THF at 0 °C, LAH (3 g, 80 mmol) was added slowly to the reaction mixture, and stirred overnight at room temperature. The excess LAH was quenched with water at 0 °C in ice bath, filtered the residue through Celite and concentrated under vacuum. The residue was redissolved in EtOAc (100 mL) and extracted with water. The combined organic layers were dried over anhydrous Na_2SO_4 and concentrated under vacuum to give a brown oil. The brown oil was then purified through a plug of silica gel (SiO_2: EtOAc → DCM/MeOH 2:1) to yield a yellow liquid, which solidify to yellow waxy solid in

fringe (4 g, 62%). ^{1}H NMR (400 MHz, $CDCl_3$) δ 7.27 – 7.21 (m, 2H), 6.97 – 6.92 (m, 2H), 4.68 (d, *J* = 2.4 Hz, 2H), 3.81 (s, 2H), 2.51 (t, *J* = 2.4 Hz, 1H).

Preparation of **S7**: 4-Hydroxybenzaldehyde (10 g, 80 mmol) was dissolved in 70 mL acetone. K_2CO_3 (22 g, 160 mmol) and propargyl bromide (80 wt. % in toluene) (17 mL, 160 mmol) was added. The reaction mixture was refluxed overnight. The reaction mixture was then filtered to remove the K_2CO_3 before concentrated under vacuum. The residue was redissolved in DCM (70 mL) and extracted with water. The combined organic layers were dried over anhydrous $MgSO_4$ and concentrated under vacuum to give a pale yellow solid. The pale yellow solid was recrystallized in hot EtOH yielding a pale yellow crystal (8 g, 62%). ^{1}H NMR (400 MHz, $CDCl_3$) δ 9.90 (s, 1H), 7.86 (d, *J* = 8.8 Hz, 2H), 7.09 (d, *J* = 8.8 Hz, 2H), 4.78 (d, *J* = 2.4 Hz, 2H), 2.57 (t, *J* = 2.4 Hz, 1H).

Preparation of **S8**: **S6** (3 g, 18 mmol) and **S7** (3 g, 18 mmol) were dissolved in 130 mL MeOH, and refluxed overnight. The reaction was cooled to 0 ℃, and $NaBH_4$ (1 g x 3, 79 mmol) was added in three times in each half an hour, and stirred overnight. The reaction mixture was concentrated under vacuum. The residue was redissolved in EtOAc (80 mL) and extracted with water. The combined organic layers were dried over anhydrous Na_2SO_4 and concentrated under vacuum to give a pale yellow liquid. The pale yellow oil was purified by column chromatography (SiO_2; Hexane/EtOAc 1:2) yielding a colorless oil (3.6 g, 65%). ^{1}H NMR (400 MHz, $CDCl_3$) δ 7.27 (d, *J* = 8.5 Hz, 4H), 6.94 (d, *J* = 8.5 Hz, 4H), 4.68 (d, *J* = 2.4 Hz, 4H), 3.73 (s, 4H), 2.51 (t, *J* = 2.5 Hz, 2H).

Preparation of Thread H·PF_6: **S8** (3.6 g, 12 mmol) was dissolved in 50 mL MeOH. Conc. HCl was added until the pH reach to 2. The MeOH was removed under vacuum. The residue was redissolved in 50 mL acetone, and saturated NH_4PF_6 solution was added until all solid was dissolved, and stirred for 30 minutes. The reaction mixture was then concentrated under vacuum to give white solid. The solid was filtered, yielding a white solid in quantitative yield. ^{1}H NMR (400 MHz, CD_3CN) δ 7.42 (d, J = 8.7 Hz, 1H), 7.04 (d, J = 8.8 Hz, 1H), 4.77 (d, J = 2.4 Hz, 1H), 4.16 (s, 1H), 2.84 (t, J = 2.4 Hz, 1H).

Synthesis of Dendron Stopper S12

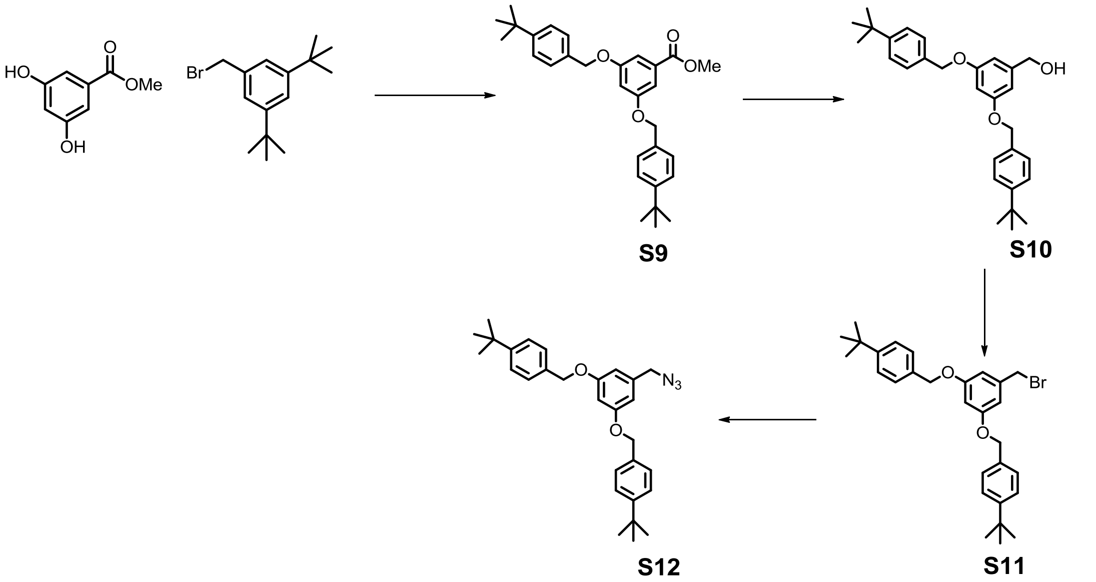

Supplementary Figure 3: Synthesis of Dendron Stopper.

S9 – S11 was synthesized according to literature with modifications.[3]

Preparation of **S9**: Methyl 3,5-dihydroxybenzoate (7 g, 40 mmol), 3,5-di-tert-butylbenzyl bromide (23 g, 80 mmol) and K_2CO_3 (22 g, 160 mmol) were dissolved in 150 mL acetone and refluxed overnight. The reaction mixture was then filtered to remove the K_2CO_3 before concentrated under vacuum. The residue was redissolved in $CHCl_3$ (100 mL) and extracted with water. The combined organic layers were dried over anhydrous $MgSO_4$ and concentrated under vacuum to give a white solid, which is pure enough without further purification. (16 g, 87 %) ^{1}H NMR (400 MHz, $CDCl_3$) δ 7.42 (d, J = 8.4 Hz, 4H), 7.37 (d, J = 8.4 Hz, 4H), 7.30 (d, J = 2.3 Hz, 2H), 6.81 (t, J = 2.3 Hz, 1H), 5.03 (s, 4H), 3.91 (s, 3H), 1.33 (s, 18H).

Preparation of **S10**: **S9** (10 g, 20 mmol) was dissolved in 100 mL THF at 0 ℃, $LiAlH_4$ (3 g, 80 mmol) was added slowly to the reaction mixture, and stirred overnight at room temperature. The excess $LiAlH_4$ was quenched with water at 0 ℃ in ice bath, filtered and concentrated under vacuum. The residue was redissolved in EtOAc (80 mL) and extracted with water. The combined organic layers were dried over anhydrous $MgSO_4$ and concentrated under vacuum to give a white solid which was pure enough without further purification (8 g, 92 %). ^{1}H NMR (400 MHz, $CDCl_3$) δ 7.42 (d, J = 8.5 Hz, 4H), 7.39 – 7.34 (m, 4H), 6.63 (d, J = 2.3 Hz, 2H), 6.56 (t, J = 2.3 Hz, 1H), 5.00 (s, 4H), 4.64 (d, J = 4.8 Hz, 2H), 1.33 (s, 18H).

Preparation of **S11**: **S10** (4 g, 9 mmol) was dissolved in 20 mL DCM, followed with the addition of PPh_3 (2.9 g, 11 mmol) and CBr_4 (3.7 g, 11 mmol) and stirred overnight. The solvent was removed under vacuum and purified by column chromatography directly (SiO_2; Hexane → Hexane/EtOAc 6 : 1) yielding a white solid (3.6 g, 80 %). ^{1}H NMR (400 MHz, $CDCl_3$) δ 7.42 (d, J = 8.4 Hz, 4H), 7.36 (d, J = 8.4 Hz, 4H), 6.64 (d, J = 2.3 Hz, 2H), 6.56 (t, J = 2.3 Hz, 1H), 4.99 (s, 4H), 4.43 (s, 2H), 1.33 (s, 18H).

Preparation of **S12**: **S11** (3.6 g, 7 mmol) was dissolved in 15 mL DMF, NaN_3 (1 g, 14 mmol) was added. The reaction mixture was stirred at 70 ℃ overnight. DMF was removed under vacuum, and the residue was redissolved in EtOAc and extracted with water, and brine (x2). The combined organic layers were dried over anhydrous $MgSO_4$ and concentrated under vacuum to give a pale yellow oil which solidify under vacuum (3 g, 94%). The product was pure enough without further purification. M.p. = 59–61 ℃. ^{1}H NMR (400 MHz, $CDCl_3$) δ 7.43 (d, J = 8.6 Hz, 4H), 7.38 (d, J = 8.4 Hz, 4H), 6.61 (t, J = 2.3 Hz, 1H), 6.57 (d, J = 2.2 Hz, 2H), 5.01 (s, 4H), 4.28 (s, 2H), 1.34 (s, 18H). ^{13}C NMR (101 MHz, $CDCl_3$) δ 160.35, 151.17, 137.59, 133.55, 127.59, 125.60, 107.06, 101.74, 70.06, 54.90, 34.65, 31.38. HRMS (ESI): $C_{29}H_{35}N_3O_2$ $[M]^+$: calcd 458.2802; found 458.2800.

Synthesis of Linker S14

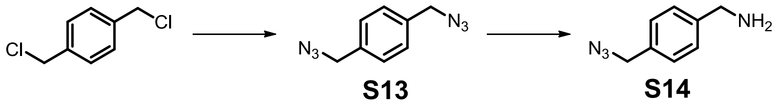

Supplementary Figure 4: Synthesis of Linker.

Linker was synthesized according to literature with modifications.[4]

Preparation of **S13**: α,α′-Dichloro-p-xylene (10 g, 50 mmol) was dissolved in 20 mL DMF, NaN_3 (9.2 g, 140 mmol) was added. The reaction mixture was stirred at 70 ℃ overnight. DMF was removed under vacuum, and the residue was redissolved in EtOAc and extracted with water, and brine (x2). The combined organic layers were dried over anhydrous $MgSO_4$ and concentrated under vacuum to give a pale yellow liquid which solidify to white solid under vacuum (8.6 g, 91 %). The product was pure enough without further purification. ^{1}H NMR (400 MHz, $CDCl_3$) δ 7.35 (s, 2H), 4.36 (s, 2H).

Preparation of **S14**: **S13** (6 g, 30 mmol) was dissolved in 20 mL diethyl ether, and 20 mL 2.5 M $HCl_{(aq)}$. The reaction mixture was cooled to 0 ℃. Triphenylphosphine (8.3

g, 30 mmol) was dissolved in 20 mL EtOAc, and was added dropwise to the reaction mixture. The reaction mixture stirred overnight at r.t. EtOAc and water added to the reaction mixture to separate two layers. The organic layer was discarded, and the water layer was washed with DCM (50 mL x 2). The water layer was basicfied to pH 11 by 1 M $NaOH_{(aq)}$, and was extracted with $CHCl_3$ (100 mL x 3). The combined organic layers were dried over anhydrous Na_2SO_4 and concentrated under vacuum to give a colorless liquid which solidify to white waxy solid in fringe (4.3 g, 88 %). The product was pure enough without further purification. ^{1}H NMR (400 MHz, $CDCl_3$) δ 7.34 (d, *J* = 8.0 Hz, 2H), 7.29 (d, *J* = 8.0 Hz, 2H), 4.32 (s, 2H), 3.89 (s, 2H).

Synthesis of G1 [2]Rotaxane Dendron-NHS

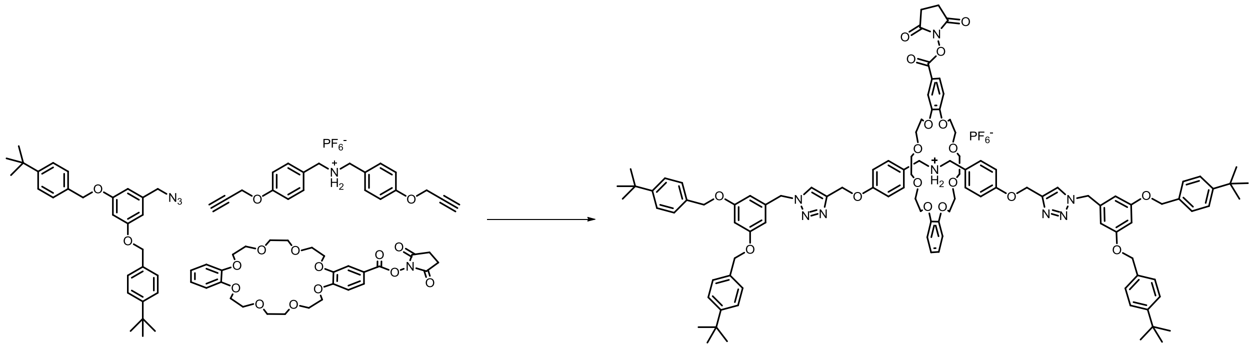

Supplementary Figure 5: Synthesis of G1 [2]Rotaxane Dendron-NHS.

Preparation of G1 [2]Rotaxane Dendron-NHS: Thread H·PF_6 (0.45 g, 1 mmol), DB24C8-OSu (0.60 g, 1 mmol), and **S12** (0.91 g, 2 mmol) were dissolved in 8 mL degassed DCM. The reaction mixture was stirred for 2 hours before the addition of $Cu(MeCN)_4PF_6$ (0.74 g, 2 mmol). The reaction was then stirred for 2 days at room temperature. The reaction mixture was diluted with 70 mL DCM, and extracted with 1.) 50 mL 2M $NaCN_{(aq)}$, 2.) 50 mL 1M $HCl_{(aq)}$, 3.) $NH_4PF_{6(aq)}$. The combined organic layers were dried over anhydrous $MgSO_4$ and concentrated under vacuum to give a yellow solid. The yellow solid was purified by column chromatography (SiO_2; $CHCl_3$/MeOH 40 : 1 → 30 : 1) yielding a white solid (1.6 g, 82 %). M.p. = 119–121 ℃. ^{1}H NMR (400 MHz, CD_3CN) δ 7.84 (s, 2H), 7.66 (dd, *J* = 8.6, 2.0 Hz, 1H), 7.41 (d, *J* = 8.4 Hz, 8H), 7.31 (d, *J* = 8.4 Hz, 8H), 7.23 (d, *J* = 8.7 Hz, 4H), 6.85 (d, *J* = 8.7 Hz, 1H), 6.81 – 6.77 (m, 2H), 6.74 (d, *J* = 8.7 Hz, 5H), 6.71 – 6.65 (m, 2H), 6.57 – 6.54 (m, 2H), 6.53 (d, *J* = 2.1 Hz, 4H), 5.44 (s, 4H), 5.01 (s, 4H), 4.98 (s, 8H), 4.56 (t, *J* = 6.7 Hz, 4H), 4.09 (br, 4H), 3.96 (dd, *J* = 14.4, 5.7 Hz, 4H), 3.82 – 3.76 (m, 4H), 3.72 – 3.69 (m, 2H), 3.66 (dd, *J* = 6.3, 3.1 Hz, 2H), 3.63 – 3.53 (m, 8H), 2.77 (s, 4H), 1.29 (s, 36H). ^{13}C NMR (101 MHz, CD_3CN) δ 171.32, 162.56, 161.23, 159.60, 154.23, 152.05, 148.67, 148.01, 139.06, 134.91, 131.77, 128.72, 126.42, 125.78, 125.40, 122.10, 117.96, 115.51, 114.05, 113.16, 113.06, 108.02, 102.52, 79.18, 71.68, 71.61, 71.55, 70.97, 70.77, 70.56, 69.46, 69.16, 68.63, 62.26, 54.42, 52.64, 35.22, 31.59, 26.47. HRMS (MALDI-TOF): $C_{107}H_{125}N_8O_{18}PF_6$ $[M–PF_6]^+$: calcd 1810.9138; found 1810.9119.

Synthesis of G1 [2]Rotaxane Dendron-N_3

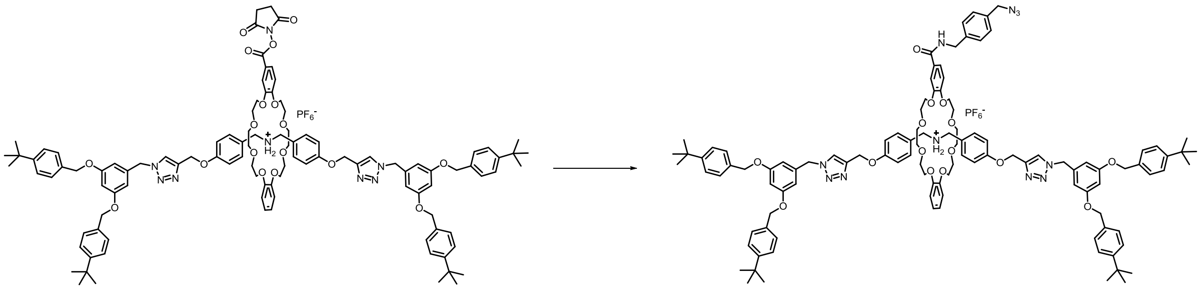

Supplementary Figure 6: Synthesis of G1 [2]Rotaxane Dendron-N_3.

Preparation of G1 [2]Rotaxane Dendron-N_3: G1 [2]Rotaxane Dendron-NHS (0.80 g, 0.40 mmol) was dissolved in 5 mL DCM, **S14** (0.13 g, 0.80 mmol) was added. The reaction mixture was stirred overnight at room temperature. The reaction mixture was diluted with 20 mL DCM and extracted with 1.) 20 mL 1 M $HCl_{(aq)}$, 2.) $NH_4PF_{6(aq)}$. The combined organic layers were dried over anhydrous $MgSO_4$ and concentrated under vacuum to give a yellow solid. The yellow solid was purified by column chromatography (SiO_2; DCM/MeOH 20 : 1) yielding a pale yellow solid (0.70 g, 87 %). M.p. = 117–119 °C. ^{1}H NMR (400 MHz, CD_3CN) δ 7.84 (s, 2H), 7.73 (t, *J* = 6.1 Hz, 1H), 7.44 – 7.39 (m, 8H), 7.35 – 7.28 (m, 12H), 7.25 – 7.21 (m, 6H), 6.83 – 6.76 (m, 3H), 6.75 – 6.69 (m, 5H), 6.57 – 6.51 (m, 6H), 5.43 (s, 4H), 4.97 (s, 12H), 4.56 (t, *J* = 6.8 Hz, 4H), 4.45 (d, *J* = 6.1 Hz, 2H), 4.28 (s, 2H), 4.06 – 3.95 (m, 8H), 3.82 – 3.73 (m, 4H), 3.71 – 3.65 (m, 4H), 3.55 – 3.62 (m, 8H), 1.29 (s, 36H). ^{13}C NMR (101 MHz, CD_3CN) δ 167.24, 161.36, 159.71, 152.18, 150.67, 148.56, 148.01, 144.48, 140.88, 139.09, 135.50, 135.05, 131.86, 130.76, 129.90, 129.50, 128.88, 128.78, 128.49, 126.50, 125.56, 125.03, 122.16, 121.32, 118.37, 115.63, 113.35, 112.59, 112.32, 108.21, 102.71, 71.93, 71.87, 71.61, 71.33, 71.28, 71.03, 70.93, 70.67, 69.18, 68.69, 62.27, 54.94, 54.53, 52.76, 43.75, 35.28, 31.62. HRMS (MALDI-TOF): $C_{111}H_{130}N_{11}O_{15}PF_6$ $[M–PF_6]^+$: calcd 1857.9774; found 1857.9743.

Synthesis of G1 [2]Rotaxane Dendron-Acetylene

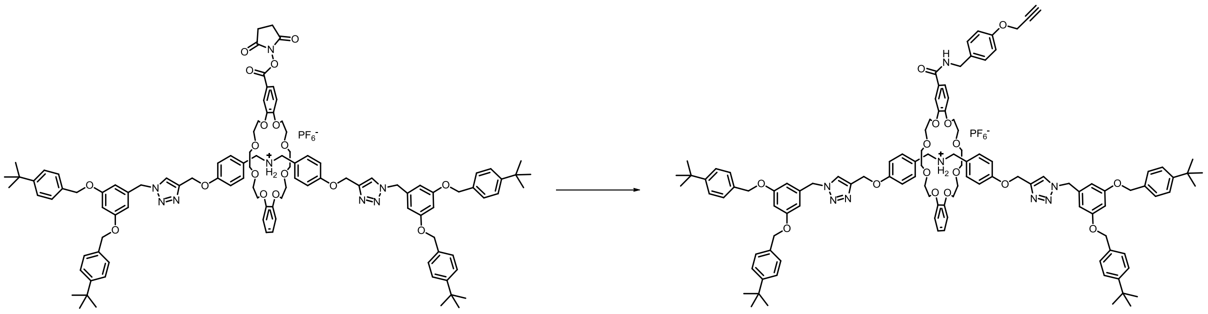

Supplementary Figure 7: Synthesis of G1 [2]Rotaxane Dendron-Acetylene.

Preparation of G1 [2]Rotaxane Dendron-Acetylene: G1 [2]Rotaxane Dendron-NHS (0.80 g, 0.40 mmol) was dissolved in 5 mL DCM, **S6** (0.13 g, 0.80 mmol) was added. The reaction mixture was stirred overnight at room temperature. The reaction mixture was diluted with 20 mL DCM and extracted with 1.) 20 mL 1M $HCl_{(aq)}$, 2.) $NH_4PF_{6(aq)}$. The combined organic layers were dried over anhydrous $MgSO_4$ and concentrated under vacuum to give a yellow solid. The pale red solid was purified by column chromatography (SiO_2; DCM/MeOH 20 : 1) yielding a pale orange solid (0.71 g, 88 %). M.p. = 110–112 °C. ^{1}H NMR (400 MHz, CD_3CN) δ 7.84 (s, 2H), 7.63 (t, *J* = 6.1 Hz, 1H), 7.41 (d, *J* = 8.3 Hz, 8H), 7.31 (d, *J* = 8.3 Hz, 10H), 7.22 (t, *J* = 9.0 Hz, 8H), 6.86 – 6.78 (m, 5H), 6.73 (d, *J* = 8.6 Hz, 4H), 6.56 – 6.52 (m, 6H), 5.44 (s, 4H), 4.97 (s, 12H), 4.65 (d, *J* = 2.5 Hz, 2H), 4.56 (t, *J* = 6.8 Hz, 4H), 4.38 (d, *J* = 5.9 Hz, 2H), 4.06 – 3.97 (m, 8H), 3.81 – 3.77 (m, 2H), 3.77 – 3.74 (m, 2H), 3.71 – 3.66 (m, 4H), 3.62 – 3.55 (m, 8H), 2.76 (t, *J* = 2.4 Hz, 1H), 1.29 (s, 36H). ^{13}C NMR (101 MHz, CD_3CN) δ 167.04, 161.30, 159.64, 157.51, 152.12, 150.56, 148.50, 147.92, 144.43, 139.04, 134.99, 133.69, 131.81, 129.85, 128.74, 128.52, 128.52, 126.45, 125.51, 125.00, 122.11, 121.25, 115.66, 115.57, 113.30, 112.52, 112.23, 108.15, 102.65, 79.88, 76.78, 71.89, 71.82, 71.56, 71.25, 70.99, 70.90, 70.62, 69.13, 68.98, 68.69, 68.64, 62.23, 56.51, 54.51, 52.72, 43.45, 35.26, 31.60. HRMS (MALDI-TOF): $C_{113}H_{131}N_8O_{16}PF_6$ $[M–PF_6]^+$: calcd 1856.9710; found 1856.9712.

Synthesis of G1 [3]Rotaxane Dendrimer

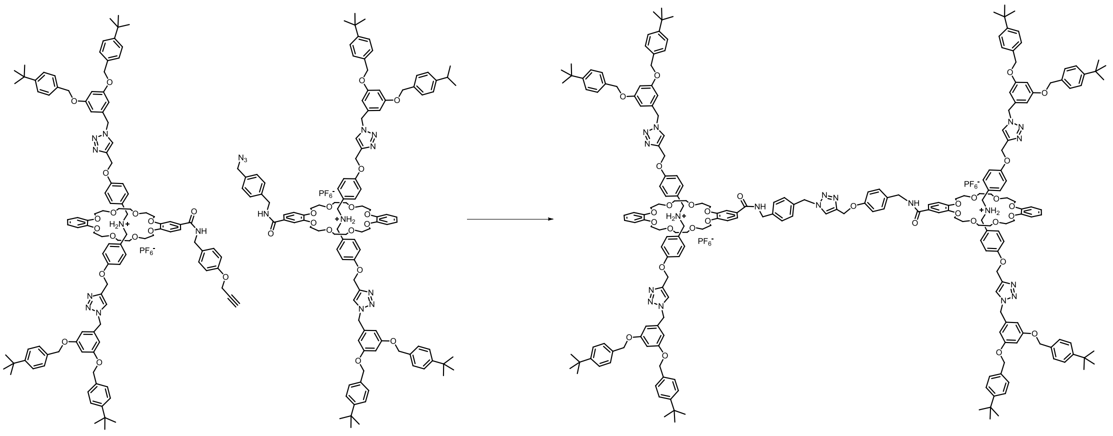

Supplementary Figure 8: Synthesis of G1 [3]Rotaxane Dendrimer.

Preparation of G1 [3]Rotaxane Dendrimer: G1 [2]Rotaxane Dendron-N_3 (0.50 g, 0.25 mmol), and G1 [2]Rotaxane Dendron-Acetylene (0.50 g, 0.25 mmol) were dissolved in 5 mL degassed DCM. $Cu(MeCN)_4PF_6$ (0.10 g, 0.25 mmol) and DIPEA (0.1 mL, 0.50 mmol) were added. The reaction was stirred for 2 days at room temperature. The reaction mixture was diluted with 50 mL DCM, and extracted with 1.) 50 mL 2M $NaCN_{(aq)}$, 2.) 50 mL 1M $HCl_{(aq)}$, 3.) $NH_4PF_{6(aq)}$. The combined organic layers were dried over anhydrous $MgSO_4$ and concentrated under vacuum to give a yellow solid. The yellow solid was purified by column chromatography (SiO_2; DCM/MeOH 30 : 1 → 20 : 1) yielding a pale yellow solid (0.83 g, 83 %). M.p. = 138–140 ℃. ^1H NMR (400 MHz, CD_3CN) δ 7.82 (s, 2H), 7.81 (s, 2H), 7.77 (t, *J* = 6.0 Hz, 1H), 7.70 (s, 1H), 7.66 (t, *J* = 6.0 Hz, 1H), 7.39 (d, *J* = 8.3 Hz, 16H), 7.29 (d, *J* = 8.3 Hz, 20H), 7.20 (t, *J* = 7.6 Hz, 12H), 7.12 (dd, *J* = 17.5, 8.3 Hz, 4H), 6.79 (dt, *J* = 6.3, 3.3 Hz, 6H), 6.73 – 6.68 (m, 8H), 6.66 (dd, *J* = 8.5, 2.4 Hz, 2H), 6.56 – 6.49 (m, 12H), 5.45 (s, 2H), 5.42 – 5.38 (m, 8H), 5.00 (s, 2H), 4.94 (s, 24H), 4.55 (t, *J* = 6.7 Hz, 8H), 4.40 (d, *J* = 6.0 Hz, 2H), 4.34 (d, *J* = 6.0 Hz, 2H), 4.05 – 3.93 (m, 16H), 3.80 – 3.72 (m, 8H), 3.67 – 3.53 (m, 24H), 1.27 (s, 72H). ^{13}C NMR (101 MHz, CD_3CN) δ 167.18, 167.05, 161.25, 159.58, 158.19, 152.07, 150.52, 150.49, 148.45, 147.84, 144.75, 144.36, 144.34, 141.01, 138.99, 135.36, 134.92, 133.19, 131.76, 129.79, 128.96, 128.91, 128.72, 128.40, 128.27, 126.42, 125.46, 125.03, 124.76, 122.08, 121.27, 115.64, 115.50, 113.23, 112.46, 112.17, 108.10, 102.57, 71.88, 71.79, 71.50, 71.27, 71.21, 70.91, 70.83, 70.57, 69.06, 68.88, 68.60, 62.37, 62.13, 54.46, 54.15, 52.66, 43.65, 43.43, 35.23, 31.59. HRMS (ESI): $C_{224}H_{261}N_{19}O_{31}P_2F_{12}$ $[M–2PF_6]^{2+}$: calcd 1857.4788; found 1857.4723.

Synthesis of G2 [4]Rotaxane Dendron-NHS

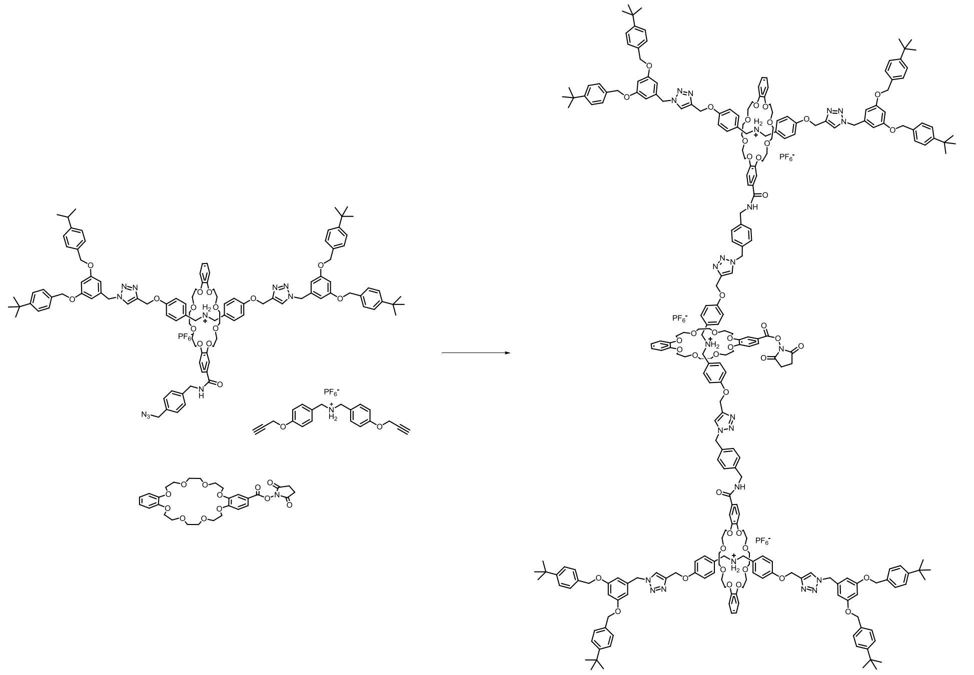

Supplementary Figure 9: Synthesis of G2 [4]Rotaxane Dendron-NHS.

Preparation of G2 [4]Rotaxane Dendron-NHS: Thread H·PF_6 (0.113 g, 0.25 mmol), DB24C8-OSu (0.221 g, 0.38 mmol), and G1 [2]Rotaxane Dendron-N_3 (1.00 g, 0.50 mmol) were dissolved in 7 mL degassed DCM. The reaction mixture was stirred for 2 hours before the addition of $Cu(MeCN)_4PF_6$ (0.10 g, 0.26 mmol). The reaction was then stirred for 4 days at room temperature. The reaction mixture was diluted with 70 mL DCM, and extracted with 1.) 50 mL 2 M $NaCN_{(aq)}$, 2.) 50 mL 1 M $HCl_{(aq)}$, 3.) $NH_4PF_{6(aq)}$. The combined organic layers were dried over anhydrous $MgSO_4$ and concentrated under vacuum to give a yellow solid. The yellow solid was purified by column chromatography (SiO_2; EtOAc → Acetone → Acetone with NH_4PF_6 (0.30 gL^{-1})) yielding a white solid (0.85 g, 67 %). M.p. = 164–166 ℃. ^{1}H NMR (400 MHz, CD_3CN) δ 7.82 (s, 4H), 7.77 (s, 2H), 7.76 – 7.71 (m, 2H), 7.39 (d, *J* = 8.4 Hz, 16H), 7.29 (d, *J* = 8.4 Hz, 20H), 7.26 – 7.21 (m, 14H), 7.20 – 7.14 (m, 8H), 6.82 – 6.75 (m, 11H), 6.73 – 6.64 (m, 16H), 6.55 – 6.50 (m, 12H), 5.41 (d, *J* = 6.8 Hz, 12H), 4.95 (s, 28H), 4.54 (q, *J* = 7.0 Hz, 12H), 4.41 (d, *J* = 6.1 Hz, 4H), 4.06 – 3.90 (m, 24H), 3.80 – 3.72 (m, 12H), 3.69 – 3.51 (m, 36H), 2.76 (s, 4H), 1.27 (s, 72H). ^{13}C NMR (101 MHz, CD_3CN) δ 167.26, 167.15, 161.31, 159.64, 157.51, 152.15, 150.61, 150.55, 148.51, 147.92, 147.89, 144.41, 141.16, 139.05, 135.33, 134.98, 133.67, 131.81, 131.79, 129.80, 129.15, 128.93, 128.76, 128.33, 126.47, 125.52, 125.49, 125.07, 124.95, 122.13, 121.31, 115.67, 115.56, 115.54, 113.30, 112.52, 112.22, 108.16, 102.64, 79.92, 76.90, 71.92, 71.84, 71.55, 71.28, 70.88, 70.62, 69.12, 68.94, 68.64, 62.19, 56.53, 54.49, 54.26, 52.70, 43.65, 43.45, 35.25, 31.61. HRMS (ESI):

$C_{271}H_{315}N_{24}O_{44}P_3F_{18}$ [M–3PF6]$^{3+}$: calcd 1537.4456; found 1537.4434.

Synthesis of G2 [4]Rotaxane Dendron-N$_3$

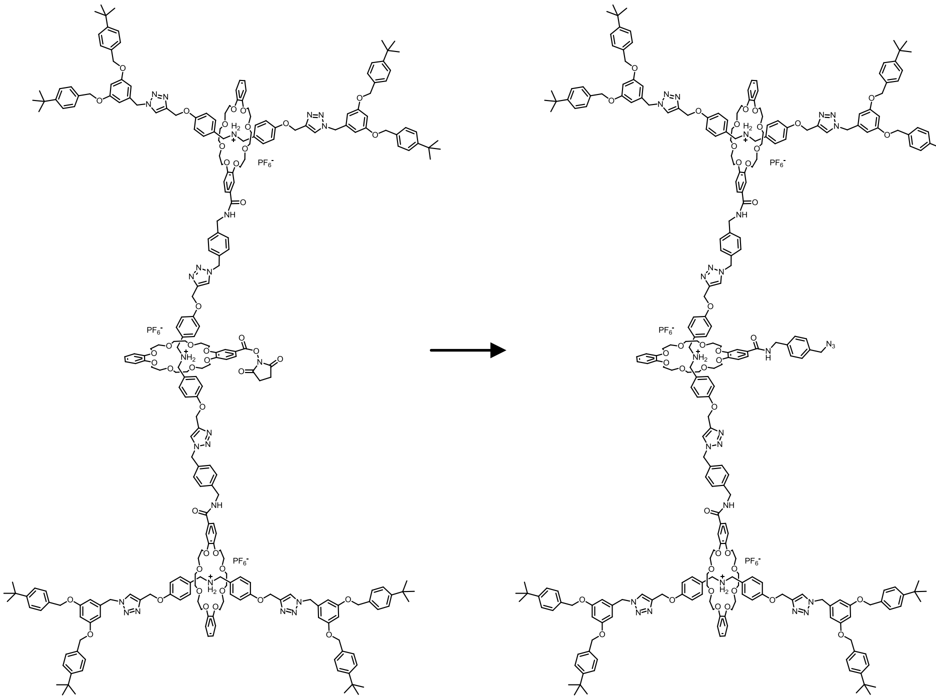

Supplementary Figure 10: Synthesis of G2 [4]Rotaxane Dendron-N$_3$.

Preparation of G2 [4]Rotaxane Dendron-N$_3$: G2 [4]Rotaxane Dendron-NHS (0.40 g, 0.08 mmol) was dissolved in 5 mL DCM, **S14** (0.05 g, 0.30 mmol) was added. The reaction mixture was stirred overnight at room temperature. The reaction mixture was diluted with 20 mL DCM and extracted with 1.) 20 mL 1M $HCl_{(aq)}$, 2.) $NH_4PF_{6(aq)}$. The combined organic layers were dried over anhydrous $MgSO_4$ and concentrated under vacuum to give a yellow solid. The yellow solid was purified by column chromatography (SiO_2; DCM/MeOH 20 : 1 → 10 : 1) yielding a pale yellow solid (0.31 g, 76 %). M.p. = 170–173 ℃. ^{1}H NMR (400 MHz, CD_3CN) δ 7.81 (d, J = 4.2 Hz, 4H), 7.76 (s, 3H), 7.74 (s, 1H), 7.39 (d, J = 8.3 Hz, 16H), 7.29 (d, J = 8.3 Hz, 22H), 7.25 – 7.18 (m, 16H), 7.19 – 7.11 (m, 8H), 6.79 (t, J = 5.4 Hz, 8H), 6.73 – 6.64 (m, 16H), 6.55 – 6.50 (m, 12H), 5.40 (s, 12H), 4.98 – 4.89 (m, 28H), 4.55 (t, J = 6.1 Hz, 12H), 4.44 – 4.38 (m, 6H), 4.25 (s, 2H), 4.06 – 3.91 (m, 24H), 3.80 – 3.72 (m, 12H), 3.68 – 3.51 (m, 36H), 1.27 (s, 72H). HRMS (ESI): $C_{275}H_{320}N_{27}O_{41}P_3F_{18}$ [M–3PF$_6$]$^{3+}$: calcd 1553.1334; found 1553.13489.

Synthesis of G2 [4]Rotaxane Dendron-Acetylene

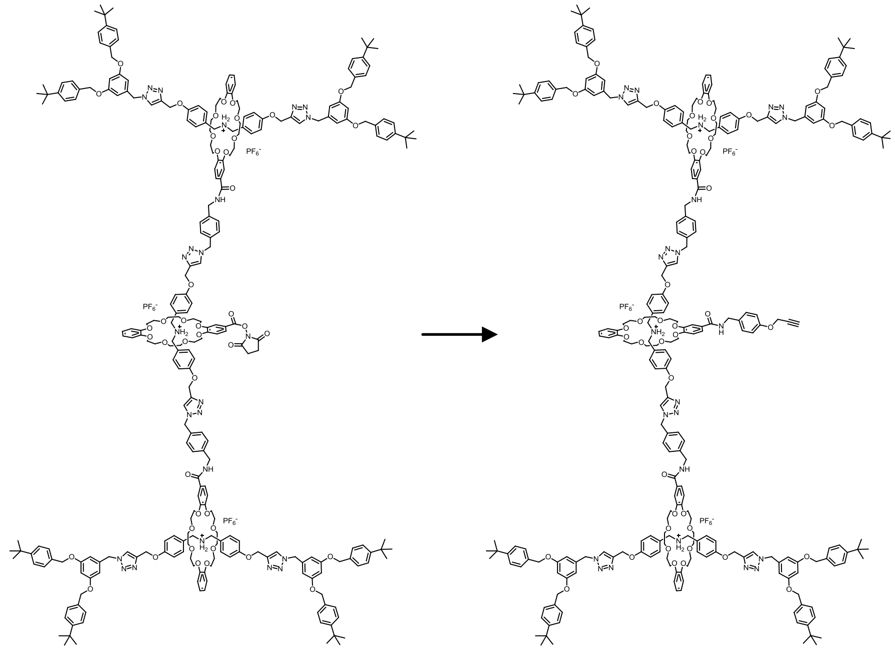

Supplementary Figure 11: Synthesis of G2 [4]Rotaxane Dendron-Acetylene.

Preparation of G2 [4]Rotaxane Dendron-Acetylene: G2 [4]Rotaxane Dendron-NHS (0.40 g, 0.08 mmol) was dissolved in 5 mL DCM, **S6** (0.05 g, 0.30 mmol) was added. The reaction mixture was stirred overnight at room temperature. The reaction mixture was diluted with 20 mL DCM and extracted with 1.) 20 mL 1M $HCl_{(aq)}$, 2.) $NH_4PF_{6(aq)}$. The combined organic layers were dried over anhydrous $MgSO_4$ and concentrated under vacuum to give an orange solid. The orange solid was purified by column chromatography (SiO_2; DCM/MeOH 20 : 1 → 10 : 1) yielding a pale orange solid (0.30 g, 74 %). M.p. = 145–147 °C. ^{1}H NMR (400 MHz, CD_3CN) δ 7.82 (s, 4H), 7.77 (d, *J* = 4.4 Hz, 4H), 7.74 (s, 1H), 7.39 (d, *J* = 8.4 Hz, 16H), 7.29 (d, *J* = 8.4 Hz, 22H), 7.25 – 7.15 (m, 24H), 6.81 – 6.77 (m, 8H), 6.73 – 6.63 (m, 16H), 6.54 – 6.51 (m, 12H), 5.40 (s, 12H), 4.94 (d, *J* = 3.8 Hz, 26H), 4.91 (s, 2H), 4.63 (d, *J* = 2.4 Hz, 2H), 4.55 (t, *J* = 6.8 Hz, 12H), 4.40 (d, *J* = 6.0 Hz, 4H), 4.36 (d, *J* = 5.9 Hz, 2H), 4.04 – 3.91 (m, 24H), 3.80 – 3.71 (m, 12H), 3.66 – 3.53 (m, 36H), 2.76 (t, *J* = 2.4 Hz, 1H), 1.27 (s, 72H). ^{13}C NMR (101 MHz, CD_3CN) δ 165.87, 165.76, 159.93, 158.26, 156.12, 150.76, 149.22, 149.16, 147.12, 146.53, 146.50, 143.03, 139.77, 137.66, 133.94, 133.60, 132.28, 130.43, 130.40, 128.41, 127.77, 127.55, 127.38, 126.94, 125.09, 124.14, 124.10, 123.68, 123.56, 120.74, 119.92, 114.28, 114.17, 114.15, 111.91, 111.13, 110.84, 106.77, 101.25, 78.54, 75.51, 70.53, 70.46, 70.16, 69.89, 69.50, 69.23, 67.73, 67.55, 67.25, 60.80, 55.14, 53.10, 52.87, 51.31, 42.27, 42.06, 33.86, 30.22. HRMS (ESI): $C_{277}H_{321}N_{24}O_{42}P_3F_{18}$ $[M–3PF_6]^{3+}$: calcd 1552.7979; found 1552.79846.

Synthesis of G2 [7]Rotaxane Dendrimer

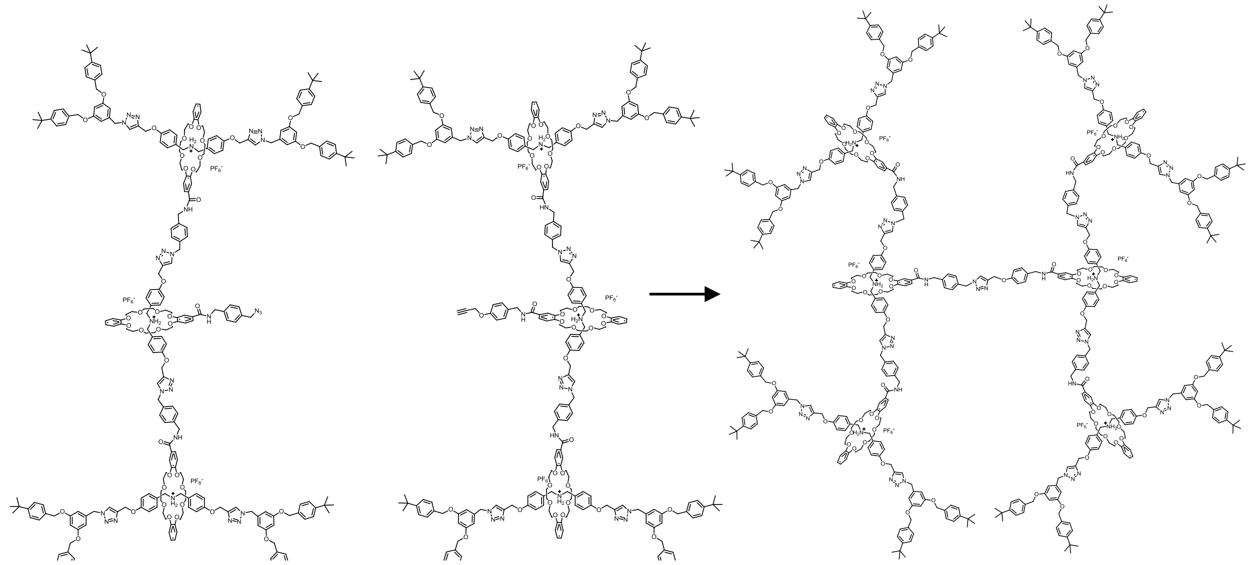

Supplementary Figure 12: Synthesis of G2 [7]Rotaxane Dendrimer.

Preparation of G2 [7]Rotaxane Dendrimer: G2 [4]Rotaxane Dendron-N_3 (0.30 g, 0.059 mmol), and G2 [4]Rotaxane Dendron-Acetylene (0.30 g, 0.059 mmol) were dissolved in 5 mL degassed DCM. $Cu(MeCN)_4PF_6$ (0.05 g, 0.13 mmol) and DIPEA (0.05 mL, 0.25 mmol) were added. The reaction was stirred for 3 days at room temperature. The reaction mixture was diluted with 30 mL DCM, and extracted with 1.) 20 mL 2 M $NaCN_{(aq)}$, 2.) 20 mL 1 M $HCl_{(aq)}$, 3.) $NH_4PF_{6(aq)}$. The combined organic layers were dried over anhydrous $MgSO_4$ and concentrated under vacuum to give a yellow solid. The yellow solid was purified by column chromatography (SiO_2; DCM/MeOH 20 : 1 → 10 : 1) yielding a pale yellow solid (0.51 g, 84 %). M.p. = 167–170 ℃. ^{1}H NMR (400 MHz, CD_3CN) δ 7.86 – 7.80 (m, 12H), 7.76 (d, *J* = 11.7 Hz, 4H), 7.73 (s, 1H), 7.41 – 7.36 (m, 32H), 7.32 – 7.26 (m, 46H), 7.20 (q, *J* = 8.2 Hz, 42H), 7.12 (dd, *J* = 8.2, 3.3 Hz, 8H), 6.78 (dt, *J* = 7.8, 3.6 Hz, 16H), 6.72 – 6.61 (m, 32H), 6.54 – 6.49 (m, 24H), 5.39 (d, *J* = 8.5 Hz, 26H), 4.98 – 4.86 (m, 58H), 4.54 (dd, *J* = 13.7, 5.4 Hz, 24H), 4.43 – 4.32 (m, 12H), 3.79 – 3.71 (m, 22H), 3.68 – 3.47 (m, 72H), 1.26 (d, *J* = 7.2 Hz, 144H). HRMS (ESI): $C_{552}H_{641}N_{51}O_{83}P_6F_{36}$ $[M–6PF_6]^{6+}$: calcd 1552.9657; found 1552.7983.

Synthesis of G3 [8]Rotaxane Dendron-NHS

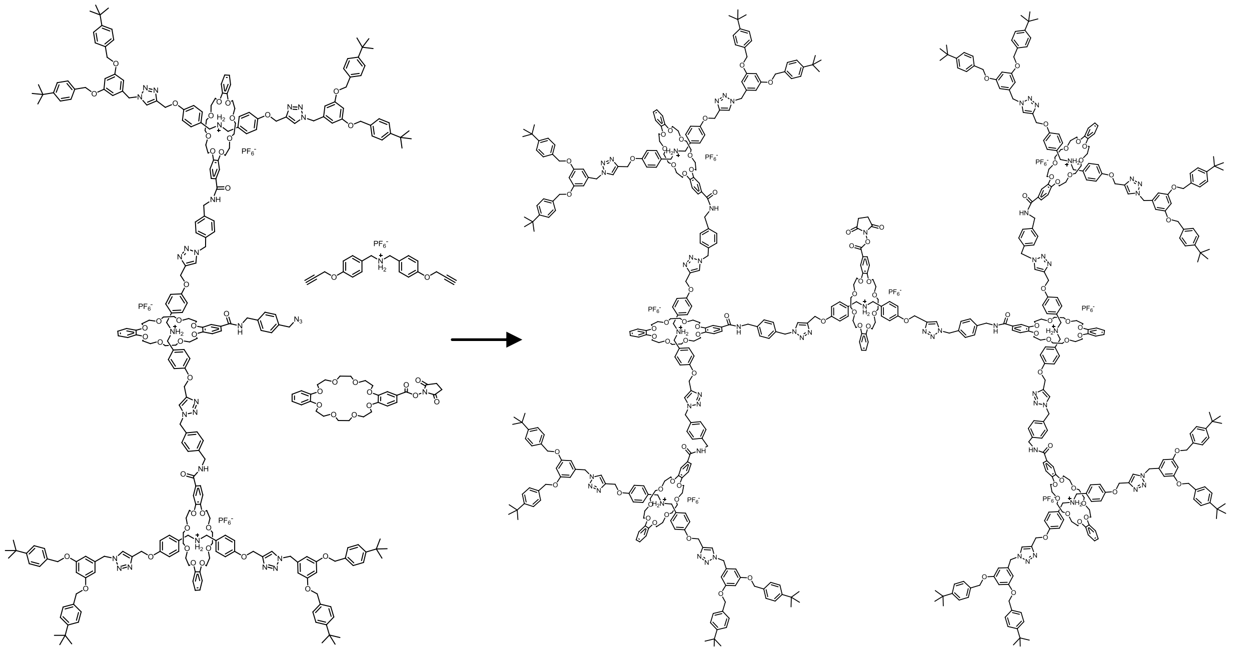

Supplementary Figure 13: Synthesis of G3 [8]Rotaxane Dendron-NHS.

Preparation of G3 [8]Rotaxane Dendron-NHS: Thread H·PF_6 (0.044 g, 0.09 mmol), DB24C8-OSu (0.15 g, 0.25 mmol), and G2 [4]Rotaxane Dendron-N_3 (1.00 g, 0.19 mmol) were dissolved in 8 mL degassed DCM. The reaction mixture was stirred for 2 hours before the addition of $Cu(MeCN)_4PF_6$ (0.10 g, 0.26 mmol). The reaction was then stirred for 6 days at room temperature. The reaction mixture was diluted with 70 mL DCM, and extracted with 1.) 50 mL 2 M $NaCN_{(aq)}$, 2.) 50 mL 1 M $HCl_{(aq)}$, 3.) $NH_4PF_{6(aq)}$. The combined organic layers were dried over anhydrous $MgSO_4$ and concentrated under vacuum to give a yellow solid. The yellow solid was purified by column chromatography (SiO_2; DCM/EtOAc 1 : 1 → Acetone → Acetone with NH_4PF_6 (0.30 gL^{-1})) yielding a pale yellow solid (0.41 g, 41 %). M.p. = 173–175 ℃. ^{1}H NMR (400 MHz, CD_3CN) δ 7.82 (s, 10H), 7.79 (s, 2H), 7.74 (s, 4H), 7.38 (d, *J* = 8.1 Hz, 34H), 7.28 (d, *J* = 8.4 Hz, 40H), 7.20 (dd, *J* = 9.8, 6.9 Hz, 41H), 7.13 (d, *J* = 8.1 Hz, 12H), 6.80 – 6.62 (m, 64H), 6.51 (t, *J* = 2.8 Hz, 24H), 5.38 (d, *J* = 4.5 Hz, 28H), 4.96 – 4.86 (m, 60H), 4.59 – 4.48 (m, 28H), 4.39 (d, *J* = 6.1 Hz, 12H), 4.04 – 3.89 (m, 54H), 3.79 – 3.70 (m, 28H), 3.67 – 3.49 (m, 86H), 2.74 (s, 4H), 1.26 (s, 144H). ^{13}C NMR (101 MHz, CD_3CN) δ 171.41, 167.28, 161.31, 159.64, 152.14, 150.62, 148.50, 147.92, 144.41, 144.36, 141.15, 141.06, 139.04, 135.30, 134.97, 131.81, 129.14, 128.93, 128.90, 128.87, 128.76, 128.30, 126.47, 125.52, 125.10, 124.98, 122.13, 121.36, 115.55, 113.29, 112.53, 112.21, 108.16, 102.63, 71.92, 71.84, 71.55, 71.27, 70.91, 70.62, 69.11, 68.93, 68.63, 62.17, 55.39, 54.48, 54.23, 52.69, 43.67, 35.25, 31.62, 26.52. HRMS (ESI): $C_{599}H_{695}N_{56}O_{96}P_7F_{42}$ $[M–7PF_6]^{7+}$: calcd

1459.3105; found 1459.3068.

Synthesis of G3 [8]Rotaxane Dendron-N_3

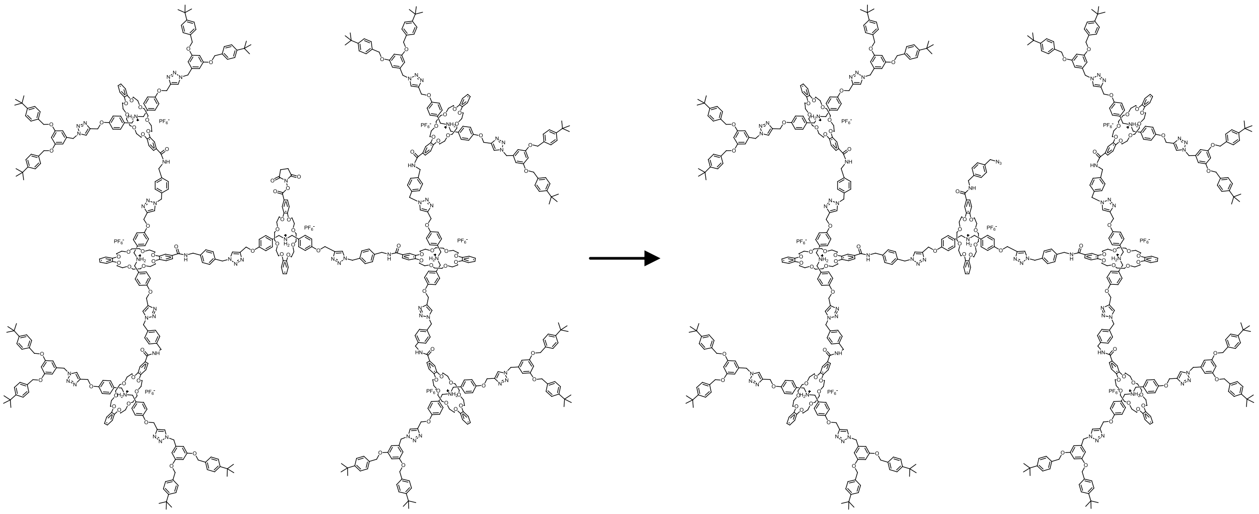

Supplementary Figure 14: Synthesis of G3 [8]Rotaxane Dendron-N_3.

Preparation of G3 [8]Rotaxane Dendron-N_3: G3 [8]Rotaxane Dendron-NHS (0.40 g, 0.036 mmol) was dissolved in 5 mL DCM, **S14** (0.05 g, 0.30 mmol) was added. The reaction mixture was stirred overnight at room temperature. The reaction mixture was diluted with 20 mL DCM and extracted with 1.) 20 mL 1M $HCl_{(aq)}$, 2.) $NH_4PF_{6(aq)}$. The combined organic layers were dried over anhydrous $MgSO_4$ and concentrated under vacuum to give a yellow solid. The yellow solid was purified by column chromatography (SiO_2; DCM/MeOH 20 : 1 → 10 : 1) yielding a pale yellow solid (0.31 g, 77 %). M.p. = 163–166 ℃. ^{1}H NMR (400 MHz, CD_3CN) δ 7.82 (s, 10H), 7.77 (s, 2H), 7.74 (s, 4H), 7.38 (d, *J* = 8.3 Hz, 34H), 7.28 (d, *J* = 8.3 Hz, 41H), 7.19 (dt, *J* = 8.3, 4.1 Hz, 44H), 7.13 (d, *J* = 7.9 Hz, 12H), 6.79 – 6.62 (m, 64H), 6.51 (t, *J* = 2.9 Hz, 24H), 5.38 (d, *J* = 4.9 Hz, 28H), 4.95 – 4.86 (m, 60H), 4.53 (br, 28H), 4.38 (d, *J* = 6.4 Hz, 14H), 4.22 (s, 2H), 4.03 – 3.89 (m, 54H), 3.78 – 3.69 (m, 28H), 3.65 – 3.49 (m, 86H), 1.26 (s, 144H). ^{13}C NMR (101 MHz, CD_3CN) δ 167.28, 161.32, 159.66, 152.15, 150.63, 148.51, 147.93, 144.42, 144.38, 141.17, 139.05, 135.30, 134.98, 131.82, 129.14, 128.94, 128.88, 128.76, 128.34, 126.47, 125.52, 125.09, 124.97, 122.14, 121.36, 115.57, 113.31, 112.53, 112.24, 108.17, 102.64, 71.94, 71.85, 71.55, 71.31, 70.90, 70.63, 69.12, 68.94, 68.65, 62.20, 55.39, 54.49, 54.24, 52.71, 43.67, 35.26, 31.62. HRMS (ESI): $C_{603}H_{700}N_{59}O_{93}P_7F_{42}$ $[M–7PF_6]^{7+}$: calcd 1466.0338; found 1465.8906.

Synthesis of G3 [8]Rotaxane Dendron-Acetylene

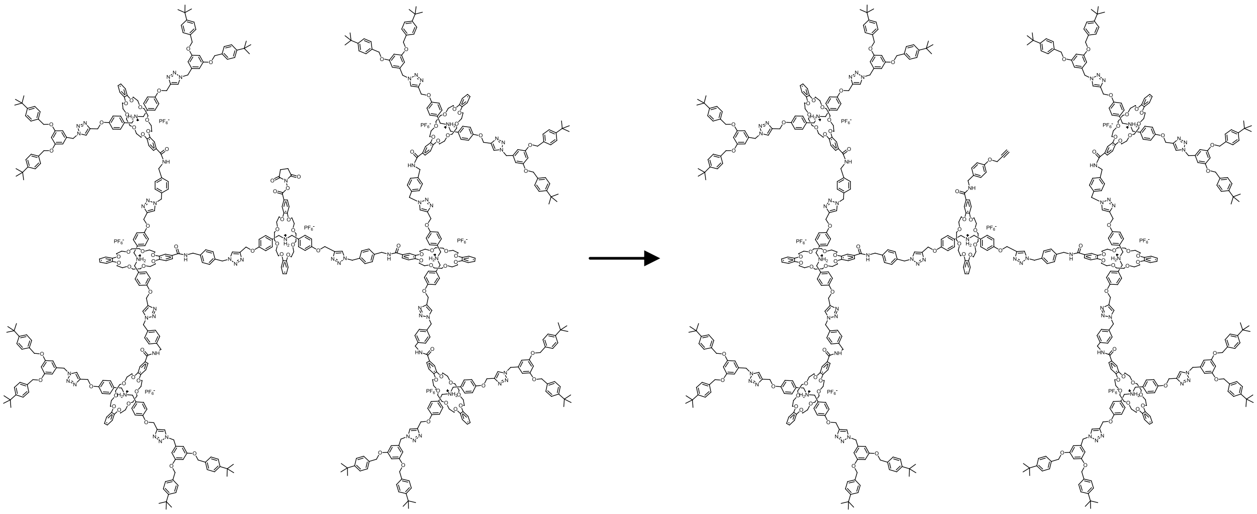

Supplementary Figure 15: Synthesis of G3 [8]Rotaxane Dendron-Acetylene.

Preparation of G3 [8]Rotaxane Dendron-N_3: G3 [8]Rotaxane Dendron-Acetylene (0.40 g, 0.036 mmol) was dissolved in 5 mL DCM, **S6** (0.05 g, 0.30 mmol) was added. The reaction mixture was stirred overnight at room temperature. The reaction mixture was diluted with 20 mL DCM and extracted with 1.) 20 mL 1M $HCl_{(aq)}$, 2.) $NH_4PF_{6(aq)}$. The combined organic layers were dried over anhydrous $MgSO_4$ and concentrated under vacuum to give an orange solid. The yellow solid was purified by column chromatography (SiO_2; DCM/MeOH 20 : 1 → 10 : 1) yielding a pale orange solid (0.28 g, 69 %). M.p. = 160–162 ℃. ^{1}H NMR (400 MHz, CD_3CN) δ 7.82 (s, 10H), 7.77 (s, 2H), 7.74 (s, 4H), 7.38 (d, *J* = 8.3 Hz, 34H), 7.28 (d, *J* = 8.3 Hz, 41H), 7.19 (dt, *J* = 8.3, 4.1 Hz, 44H), 7.13 (d, *J* = 7.9 Hz, 12H), 6.79 – 6.62 (m, 64H), 6.51 (t, *J* = 2.9 Hz, 24H), 5.38 (d, *J* = 4.9 Hz, 28H), 4.95 – 4.86 (m, 60H), 4.53 (br, 28H), 4.38 (d, *J* = 6.4 Hz, 14H), 4.22 (s, 2H), 4.03 – 3.89 (m, 54H), 3.78 – 3.69 (m, 28H), 3.65 – 3.49 (m, 86H), 1.26 (s, 144H). ^{13}C NMR (101 MHz, CD_3CN) δ 167.29, 161.31, 159.64, 152.14, 150.62, 148.50, 147.91, 147.88, 144.41, 144.36, 141.14, 139.04, 135.29, 134.97, 131.81, 129.79, 129.13, 128.93, 128.87, 128.76, 128.30, 126.47, 125.51, 125.48, 125.10, 124.98, 122.13, 121.35, 115.65, 115.55, 115.52, 113.29, 112.52, 112.21, 108.15, 102.63, 79.94, 76.98, 71.92, 71.84, 71.54, 71.27, 70.90, 70.62, 69.11, 68.92, 68.62, 62.17, 56.53, 55.39, 54.48, 54.24, 52.69, 43.68, 35.25, 31.62. HRMS (ESI): $C_{605}H_{701}N_{56}O_{94}P_7F_{42}$ $[M–7PF_6]^{7+}$: calcd 1465.8900; found 1465.7452.

Synthesis of G3 [15]Rotaxane Dendrimer

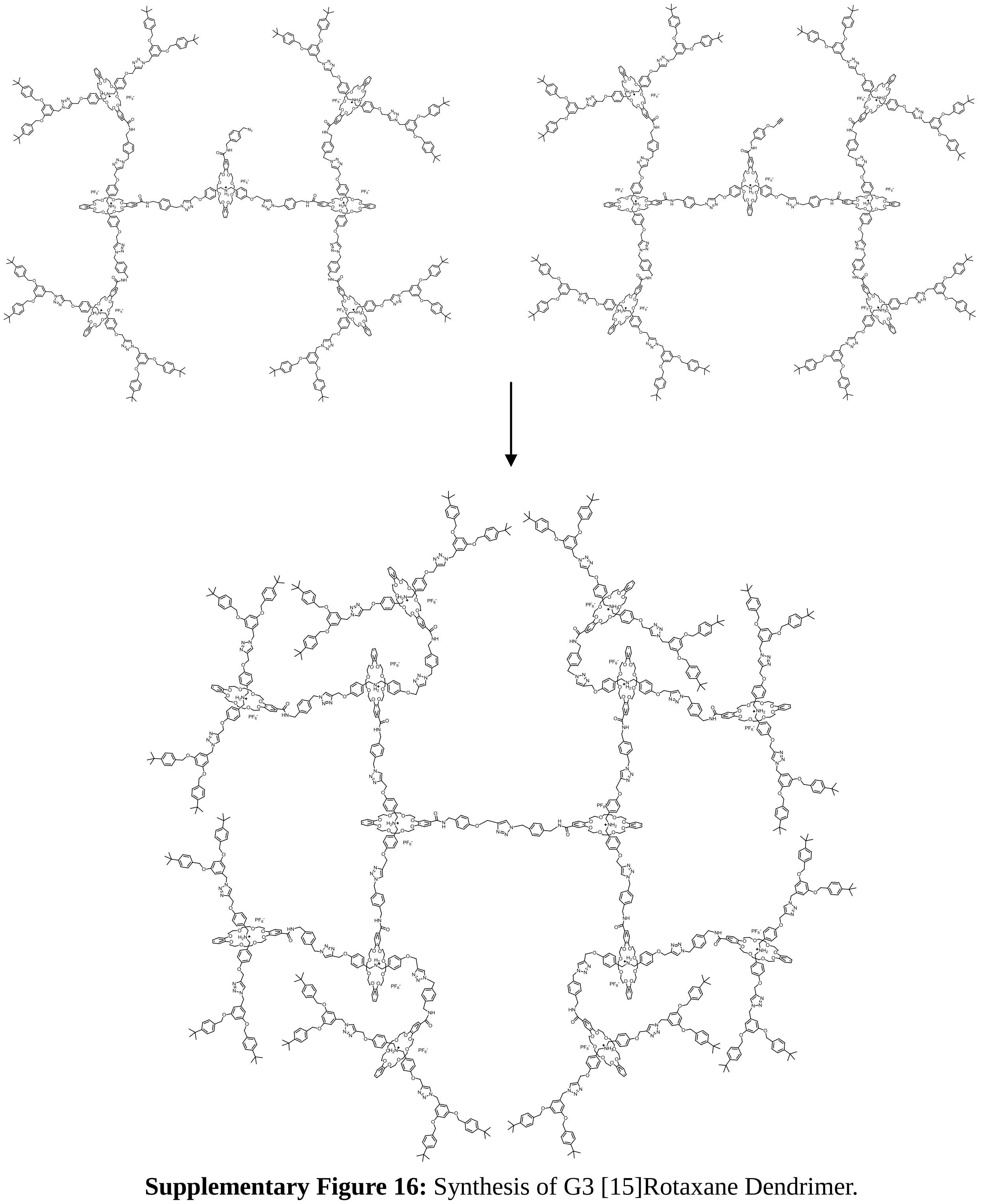

Supplementary Figure 16: Synthesis of G3 [15]Rotaxane Dendrimer.

Preparation of G3 [15]Rotaxane Dendrimer: G3 [8]Rotaxane Dendron-N_3 (0.30 g, 0.027 mmol), and G3 [8]Rotaxane Dendron-Acetylene (0.30 g, 0.027 mmol) were dissolved in 5 mL degassed DCM. $Cu(MeCN)_4PF_6$ (0.03 g, 0.08 mmol) and DIPEA (0.02 mL, 0.12 mmol) were added. The reaction was stirred for 3 days at room

temperature. The reaction mixture was diluted with 30 mL DCM, and extracted with 1.) 20 mL 2 M $NaCN_{(aq)}$, 2.) 20 mL 1 M $HCl_{(aq)}$, 3.) $NH_4PF_{6(aq)}$. The combined organic layers were dried over anhydrous $MgSO_4$ and concentrated under vacuum to give a yellow solid. The yellow solid was purified by column chromatography (SiO_2; DCM/MeOH 20 : 1 → 10 : 1) yielding a pale yellow solid (0.43 g, 71 %). M.p. = 179–181 ℃. ^{1}H NMR (400 MHz, CD_3CN) δ 7.82 (d, J = 1.9 Hz, 20H), 7.74 (d, J = 3.9 Hz, 13H), 7.40 – 7.34 (m, 68H), 7.31 – 7.25 (m, 82H), 7.19 (d, J = 8.6 Hz, 88H), 7.10 (d, J = 8.9 Hz, 24H), 6.80 – 6.61 (m, 128H), 6.54 – 6.47 (m, 48H), 5.37 (t, J = 7.3 Hz, 56H), 4.97 – 4.82 (m, 124H), 4.52 (br, 56H), 4.37 (br, 28H), 4.04 – 3.86 (m, 112H), 3.80 – 3.68 (m, 56H), 3.65 – 3.47 (m, 168H), 1.25 (d, J = 6.0 Hz, 288H). ^{13}C NMR (101 MHz, CD_3CN) δ 167.38, 161.30, 159.63, 152.09, 150.69, 150.65, 148.45, 147.93, 144.49, 144.39, 144.34, 141.08, 141.02, 139.01, 135.25, 134.93, 132.19, 131.79, 129.12, 129.05, 128.92, 128.86, 128.75, 128.22, 128.18, 126.45, 125.47, 125.13, 125.02, 122.13, 121.43, 116.01, 115.53, 113.26, 112.53, 112.18, 108.15, 102.62, 71.85, 71.54, 71.23, 70.90, 70.61, 69.08, 68.89, 68.61, 62.16, 55.39, 54.47, 54.21, 52.67, 43.72, 35.24, 31.66. HRMS (ESI): $C_{1208}H_{1401}N_{115}O_{187}P_{14}F_{84}$ $[M{-}13PF_6]^{13+}$: calcd 1589.8018; found 1588.96254.

Synthesis of G4 [16]Rotaxane Dendrion-NHS

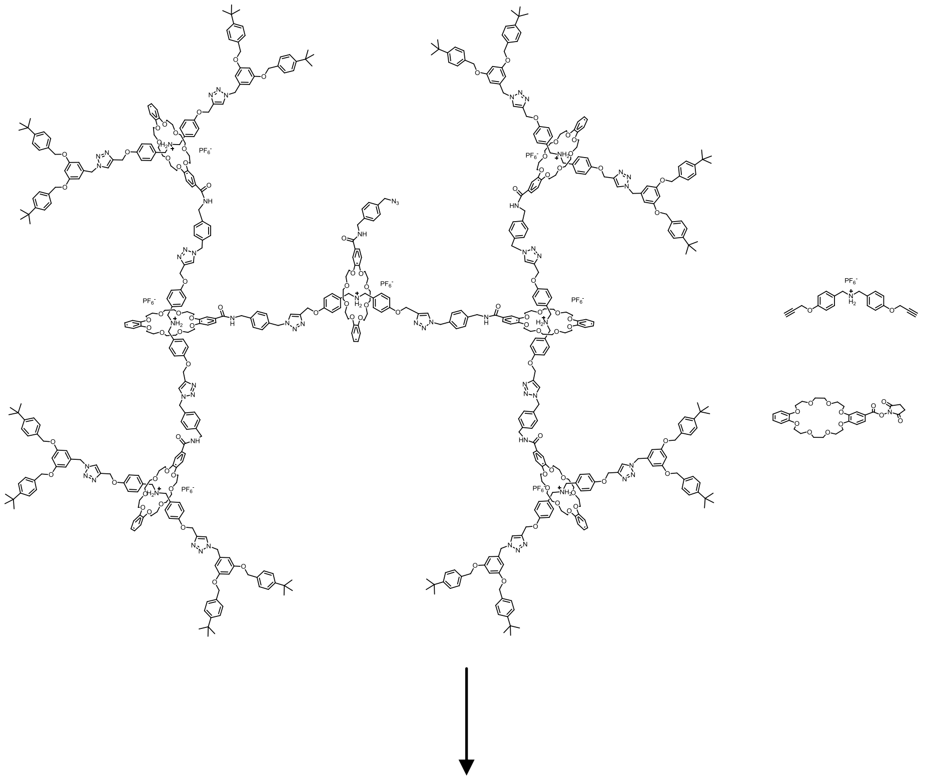

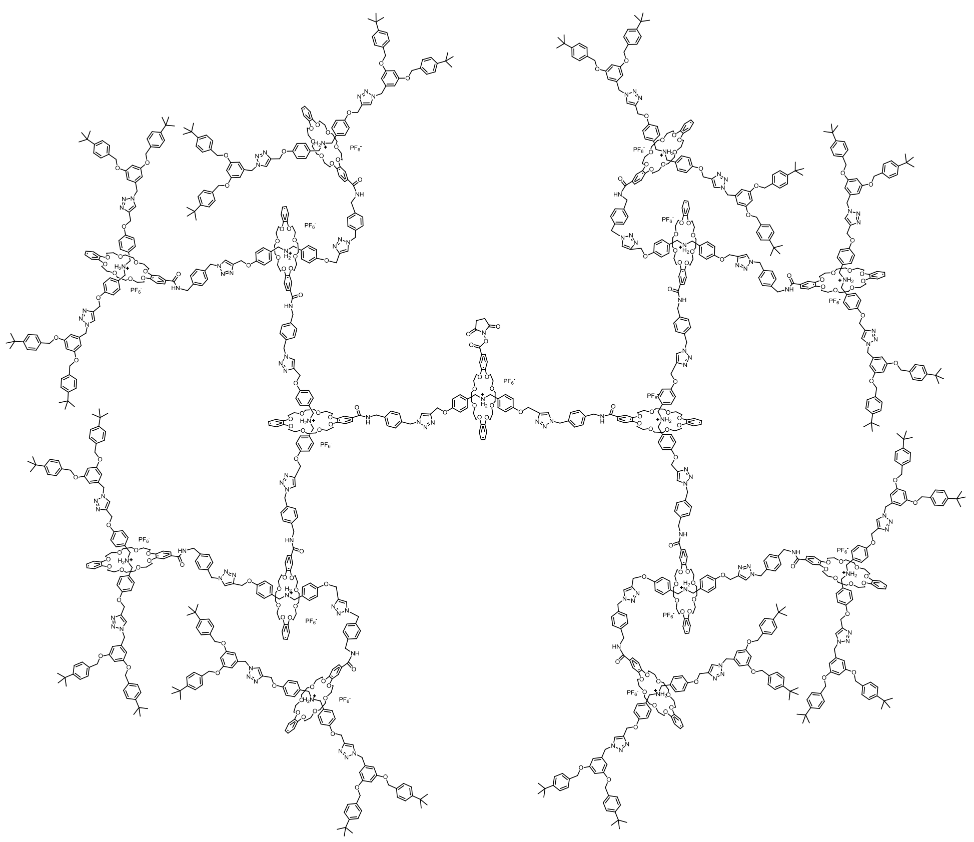

Supplementary Figure 17: Synthesis of G4 [16]Rotaxane Dendron-NHS.

Preparation of G4 [16]Rotaxane Dendron-NHS: Thread H·PF_6 (0.014 g, 0.031 mmol), DB24C8-OSu (0.080 g, 0.13 mmol), and G3 [8]Rotaxane Dendron-N_3 (0.70 g, 0.062 mmol) were dissolved in 8 mL degassed DCM. The reaction mixture was stirred for 2 hours before the addition of $Cu(MeCN)_4PF_6$ (0.10 g, 0.26 mmol). The reaction was then stirred for 6 days at room temperature. The reaction mixture was diluted with 70 mL DCM, and extracted with 1.) 50 mL 2M $NaCN_{(aq)}$, 2.) 50 mL 1 M $HCl_{(aq)}$, 3.) $NH_4PF_{6(aq)}$. The combined organic layers were dried over anhydrous $MgSO_4$ and concentrated under vacuum to give a yellow solid. The yellow solid was purified by column chromatography (SiO_2; DCM/EtOAc 1 : 1 → Acetone → Acetone with NH_4PF_6 (0.30 gL^{-1})) yielding a pale yellow solid (0.30 g, 41 %). M.p. = 178–180 ℃. ^1H NMR (400 MHz, CD_3CN) δ 7.82 (d, *J* = 2.1 Hz, 22H), 7.77 – 7.73 (m, 15H), 7.39 – 7.36 (m, 68H), 7.29 – 7.26 (m, 84H), 7.22 – 7.17 (m, 88H), 7.13 – 7.07 (m, 25H), 6.79 – 6.64 (m, 132H), 6.51 (d, *J* = 3.2 Hz, 48H), 5.39 – 5.34 (m, 58H), 4.93 – 4.86 (m, 128H), 4.53 (br, 60H), 4.37 (br, 31H), 4.03 – 3.89 (m, 120H), 3.76 – 3.72 (m, 64H), 3.64 – 3.51 (m, 176H), 2.72 (s, 4H), 1.25 (d, J = 5.0 Hz, 289H). ^{13}C NMR (101 MHz, CD_3CN) δ 166.03, 165.96, 159.92, 158.24, 150.74, 149.25, 147.09, 146.53, 146.50, 143.01, 142.96, 139.71, 139.66, 137.64, 133.89, 133.57, 130.42, 130.39, 127.74, 127.66, 127.52, 127.37, 126.86, 126.81, 125.07, 124.12, 123.72, 123.61,

120.74, 120.00, 114.15, 111.89, 111.14, 110.79, 106.76, 101.23, 70.53, 70.45, 70.15, 69.87, 69.51, 69.22, 67.71, 67.52, 67.23, 60.78, 54.00, 53.09, 52.83, 51.30, 50.51, 42.30, 33.85, 30.24. (two peaks are missing) HRMS (ESI): $C_{1255}H_{1455}N_{120}O_{200}P_{15}F_{90}$ $[M–13PF_6]^{13+}$: calcd 1669.9046; found 1668.98011.

Synthesis of model compounds of rotaxane dendrimer

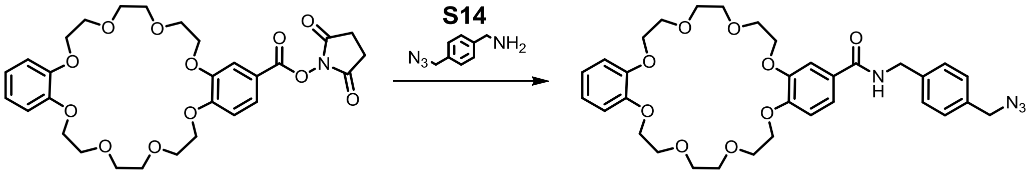

DB24C8-OSu S15

Supplementary Figure 18: Synthesis of model compound **S15.**

Preparation of model compound **S15**: DB24C8-OSu (0.80 g, 1.35 mmol) was dissolved in 4 mL DCM, and **S14** (0.33 g, 2.03 mmol) was added dropwise to the reaction mixture. The reaction was stirred overnight at room temperature. The reaction mixture was diluted with 15 mL DCM, and extracted with 10 mL 1 M $HCl_{(aq)}$. The combined organic layers were dried over anhydrous $MgSO_4$ and concentrated under vacuum to give a pale yellow solid. The crude pale yellow solid was recrystallized in hot EtOH yielding a white solid (0.82 g, 95 %). M.p. = 106–108 ℃. ^{1}H NMR (400 MHz, $CDCl_3$) δ 7.42 (d, *J* = 2.1 Hz, 1H), 7.36 (d, *J* = 8.1 Hz, 2H), 7.31 – 7.26 (m, 4H), 6.87 (d, *J* = 3.1 Hz, 4H), 6.47 (t, *J* = 5.8 Hz, 1H), 4.62 (d, *J* = 5.7 Hz, 2H), 4.32 (s, 2H), 4.20 – 4.13 (m, 8H), 3.93 – 3.89 (m, 8H), 3.82 (d, J = 5.3 Hz, 8H). ^{13}C NMR (101 MHz, $CDCl_3$) δ 166.90, 151.77, 148.87, 148.68, 138.65, 134.68, 128.65, 128.36, 127.10, 121.43, 121.41, 119.94, 113.99, 113.00, 112.39, 71.43, 71.29, 71.25, 69.92, 69.80, 69.69, 69.51, 69.36, 69.27, 54.47, 43.73. HRMS (MALDI-TOF): $C_{33}H_{40}N_4O_9$ $[M+Na]^+$: calcd 659.2689; found 659.2664.

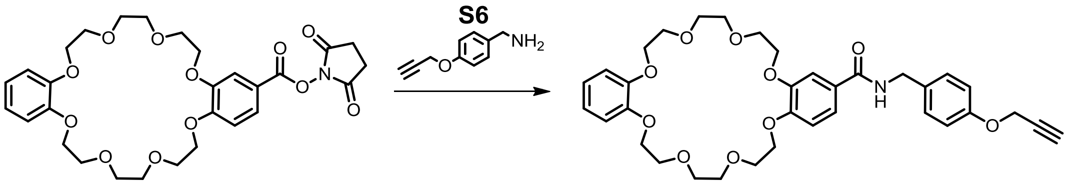

DB24C8-OSu S16

Supplementary Figure 19: Synthesis of model compound **S16.**

Preparation of model compound **S16**: DB24C8-OSu (0.80 g, 1.35 mmol) was dissolved in 4 mL DCM, and **S6** (0.32 g, 2.03 mmol) was added dropwise to the reaction mixture. The reaction was stirred overnight at room temperature. The reaction

mixture was diluted with 15 mL DCM, and extracted with 10 mL 1 M $HCl_{(aq)}$. The combined organic layers were dried over anhydrous $MgSO_4$ and concentrated under vacuum to give a yellow solid. The crude yellow solid was recrystallized in hot EtOH yielding a pale yellow solid (0.80 g, 93 %). M.p. = 124–127 ℃. ^{1}H NMR (400 MHz, $CDCl_3$) δ 7.40 (d, J = 2.1 Hz, 1H), 7.31 – 7.24 (m, 4H), 6.95 (d, J = 8.7 Hz, 2H), 6.87 (d, J = 3.0 Hz, 4H), 6.37 (t, J = 5.7 Hz, 1H), 4.68 (d, J = 2.4 Hz, 2H), 4.55 (d, J = 5.5 Hz, 2H), 4.19 – 4.12 (m, 8H), 3.91 (dd, J = 5.8, 2.9 Hz, 8H), 3.82 (d, J = 5.3 Hz, 8H), 2.52 (t, J = 2.4 Hz, 1H). ^{13}C NMR (101 MHz, $CDCl_3$) δ 166.81, 156.99, 151.68, 148.88, 148.64, 131.42, 129.31, 127.23, 121.43, 121.40, 119.90, 115.16, 113.99, 112.95, 112.36, 99.98, 78.48, 75.64, 71.42, 71.29, 69.93, 69.80, 69.70, 69.49, 69.36, 69.28, 55.85, 43.59. HRMS (MALDI-TOF): $C_{35}H_{41}NO_{10}$ $[M+Na]^+$: calcd 658.2633; found 658.2652.

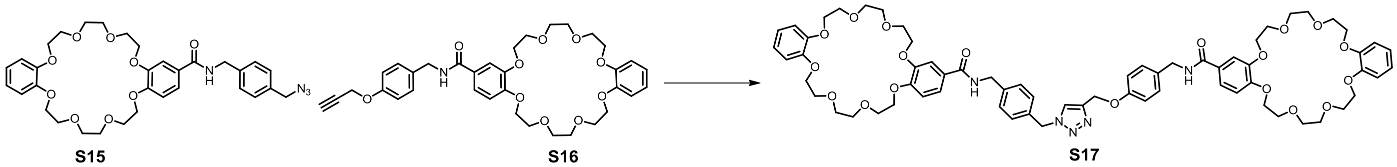

Supplementary Figure 20: Synthesis of model compound **S17.**

Preparation of model compound **S17**: **S15** (0.3 g, 0.47 mmol) and **S16** (0.3 g, 0.47 mmol) were dissolved in 5 mL DCM, $Cu(MeCN)_4PF_6$ (0.17 g, 0.47 mmol) and DIPEA (0.16 mL, 0.94 mmol) were added. The reaction was stirred 24 hours at room temperature. The reaction mixture was diluted with 20 mL DCM, and extracted with 1.) 20 mL 2 M $NaCN_{(aq)}$, 2.) 20 mL 1 M $HCl_{(aq)}$. The combined organic layers were dried over anhydrous $MgSO_4$ and concentrated under vacuum to give a pale yellow solid. The pale yellow solid was purified by column chromatography (SiO_2; DCM/MeOH 10 : 1 → 5 : 1) yielding a white glassy solid (0.4 g, 67%). M.p. = 60–63 ℃. ^{1}H NMR (400 MHz, CD_3CN) δ 7.80 (s, 1H), 7.56 (br, 1H), 7.47 (br, 1H), 7.42 – 7.37 (m, 4H), 7.33 – 7.29 (m, 2H), 7.26 – 7.18 (m, 4H), 6.96 – 6.87 (m, 12H), 5.50 (s, 2H), 5.10 (s, 2H), 4.48 (d, J = 6.0 Hz, 2H), 4.42 (d, J = 5.9 Hz, 2H), 4.17 – 4.12 (m, 8H), 4.09 – 4.11 (m, 8H), 3.76 – 3.81 (m, 16H), 3.65 – 3.68 (m, 16H). ^{13}C NMR (101 MHz, CD_3CN) δ 165.88, 165.77, 156.82, 150.85, 148.36, 148.31, 147.77, 143.42, 139.70, 134.14, 131.90, 128.38, 127.60, 127.57, 127.00, 126.85, 123.42, 120.99, 120.95, 120.23, 120.20, 114.44, 113.70, 113.59, 112.20, 112.15, 70.11, 70.08, 70.04, 69.02, 68.98, 68.88, 68.76, 68.48, 68.38, 68.33, 68.27, 61.06, 52.80, 42.25, 42.02. HRMS (MALDI-TOF): $C_{68}H_{81}N_5O_{19}$ $[M+Na]^+$: calcd 1294.5429; found 1294.5405.

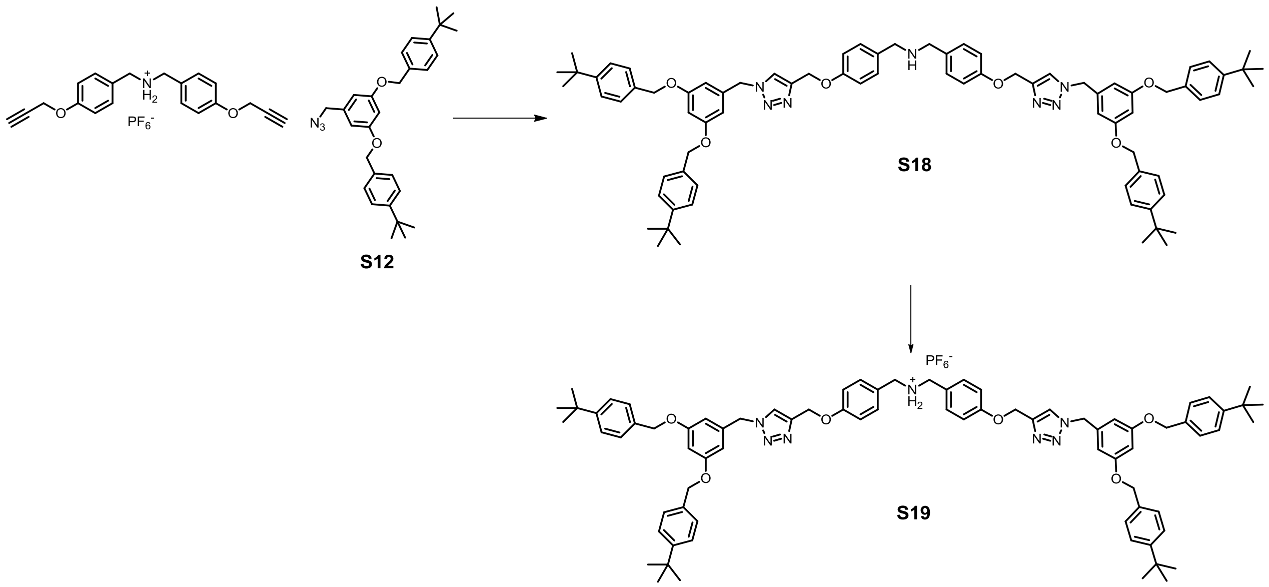

Supplementary Figure 21: Synthesis of model compound **S18** and **S19.**

Preparation of model compound **S18**: Thread H·PF_6 (0.2 g, 0.44 mmol), **S12** (0.4 g, 0.89 mmol) were dissolved in 7 mL DCM, $Cu(MeCN)_4PF_6$ and DIPEA were added. The reaction was stirred 24 hours at room temperature. The reaction mixture was diluted with 20 mL DCM, and extracted with 1.) 20 mL 2 M $NaCN_{(aq)}$, 2.) 20 mL 1 M $NaOH_{(aq)}$. The combined organic layers were dried over anhydrous $MgSO_4$ and concentrated under vacuum to give a yellow solid. The yellow solid was purified by column chromatography (SiO_2; EtOAc) yielding a pale yellow solid (0.43 g, 80%). M.p. = 68–70 ℃. ^1H NMR (400 MHz, CD_3CN) δ 7.82 (s, 2H), 7.41 (d, J = 8.4 Hz, 8H), 7.31 (d, J = 8.3 Hz, 8H), 7.22 (d, J = 8.6 Hz, 4H), 6.93 (d, J = 8.7 Hz, 4H), 6.53 (t, J = 2.3 Hz, 2H), 6.49 (d, J = 2.2 Hz, 4H), 5.43 (s, 4H), 5.12 (s, 4H), 4.96 (s, 8H), 3.60 (s, 4H), 1.29 (s, 36H). ^{13}C NMR (101 MHz, CD_3CN) δ 159.91, 156.83, 150.73, 143.55, 137.79, 133.65, 133.23, 128.91, 127.41, 125.08, 123.48, 114.18, 106.55, 101.30, 69.21, 61.12, 53.03, 51.57, 33.86, 30.21. HRMS (MALDI-TOF): $C_{78}H_{89}N_7O_6$ $[M+Na]^+$: calcd 1242.6775; found 1242.6728.

Preparation of model compound **S19**: **S18** (0.1 g, 0.82 mmol) was dissolved in 10 mL MeOH, and conc. HCl was added until the pH reach 2. MeOH was then removed under vacuum to give a yellow solid. The residue was redissolved in 10 mL DCM, 10 mL saturated $NH_4PF_{6(aq)}$ was added and stirred for 30 minutes. The organic layers were dried over anhydrous $MgSO_4$ and concentrated under vacuum to give a yellow solid (80 mg, 71%). M.p. = 63–65 ℃ ^1H NMR (400 MHz, CD_3CN) δ 7.87 (s, 2H), 7.42 (d, J = 8.5 Hz, 8H), 7.37 – 7.31 (m, 12H), 7.05 (d, J = 8.7 Hz, 4H), 6.55 (t, J = 2.3 Hz, 2H), 6.51 (d, J = 2.2 Hz, 4H), 5.45 (s, 4H), 5.16 (s, 4H), 4.98 (s, 8H), 4.05 (s,

4H), 1.30 (s, 36H). ^{13}C NMR (101 MHz, CD_3CN) δ 159.94, 158.82, 150.78, 143.09, 137.73, 133.65, 131.24, 127.41, 125.10, 123.68, 123.44, 114.74, 106.66, 101.28, 69.24, 61.16, 53.09, 50.50, 33.87, 30.20.

Preparation of Neutral G1 – 3 Rotaxane Dendrimers

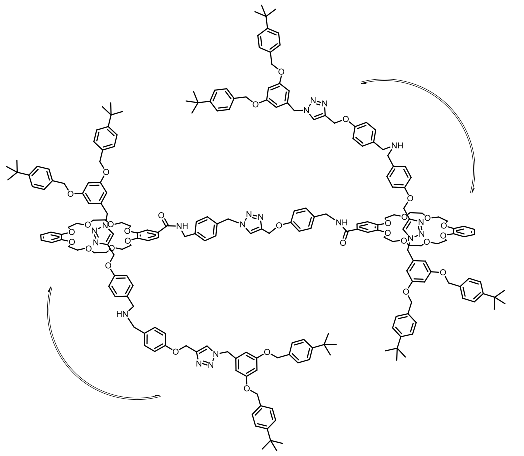

Supplementary Figure 22: Neutral G1 [3]rotaxane dendrimer.

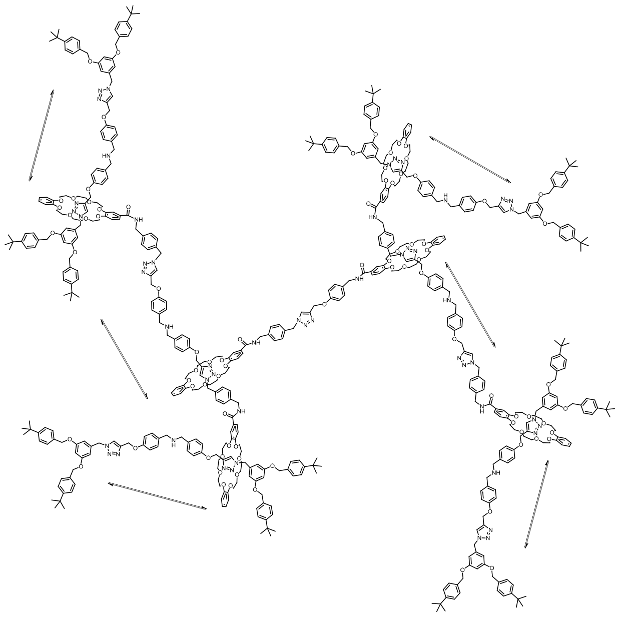

Supplementary Figure 23: Neutral G2 [7]rotaxane dendrimer.

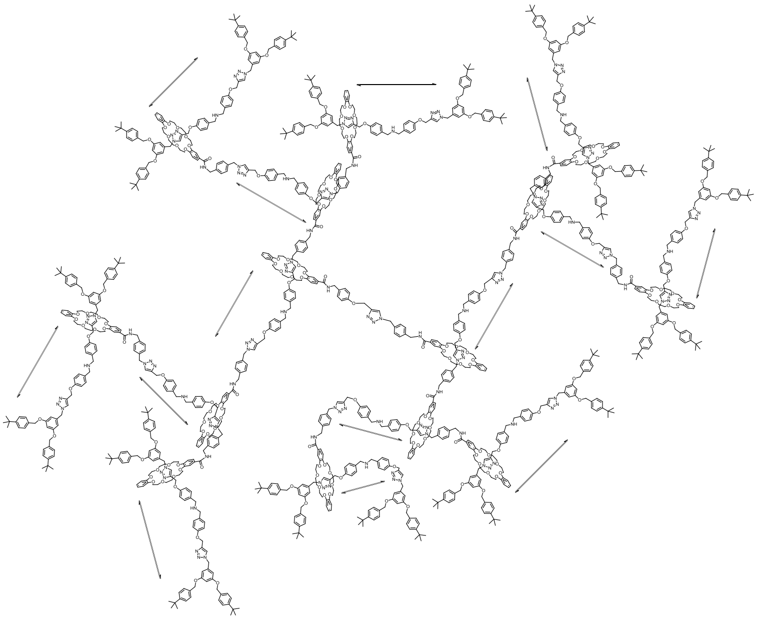

Supplementary Figure 24: Neutral G3 [15]rotaxane dendrimer.

General procedure:G(n) rotaxane dendrimers were dissolved in 1 mL CD_3CN, BEMP resin (2-tert-butylimino-2-diethylamino-1,3-dimethylperhydro-1,3,2-diazaphosphorine, polymer-bound (Sigma-Aldrich)) was added and stirred overnight at room temperature. DCM was added to redissolved the deprotonated neutral rotaxane dendrimer, BEMP resin was filtered, and the combined organic layers were concentrated under vacuum to afford the deprotonated G(n) rotaxane dendrimer as a yellow solid in quantitative yield.

Previous synthetic approach towards G2 [7]rotaxane dendrimer

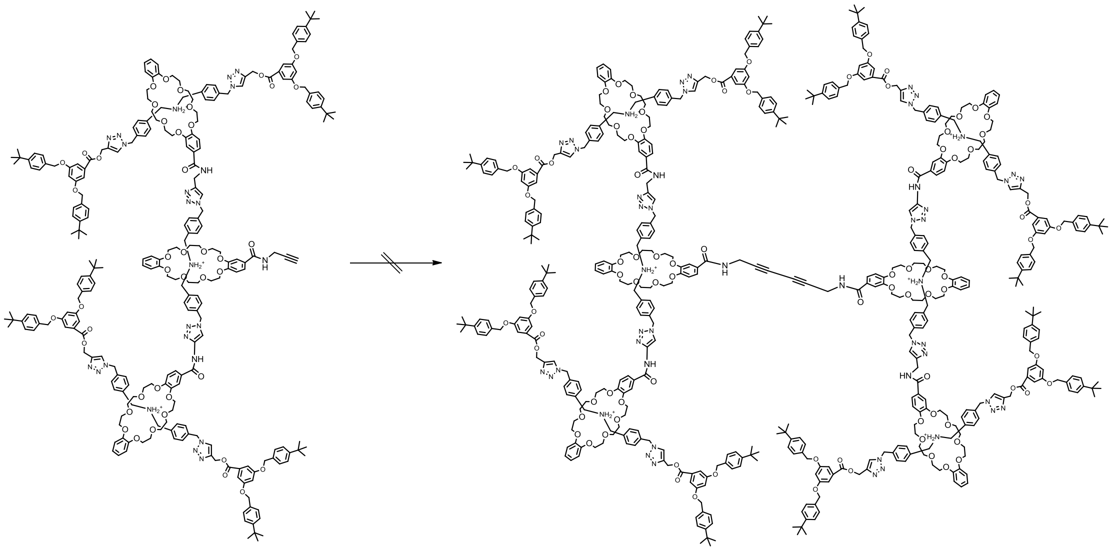

Supplementary Figure 25: Attempt synthesis of G2 [7]Rotaxane Dendrimer through Glaser-Hay's acetylene homo-coupling.[5] Conditions: $Cu(MeCN)_4PF_6$, TMEDA, O_2,

THF, 24h.

Stacked ^{1}H NMR spectra of Rotaxane Dendrons and Rotaxane Dendrimers

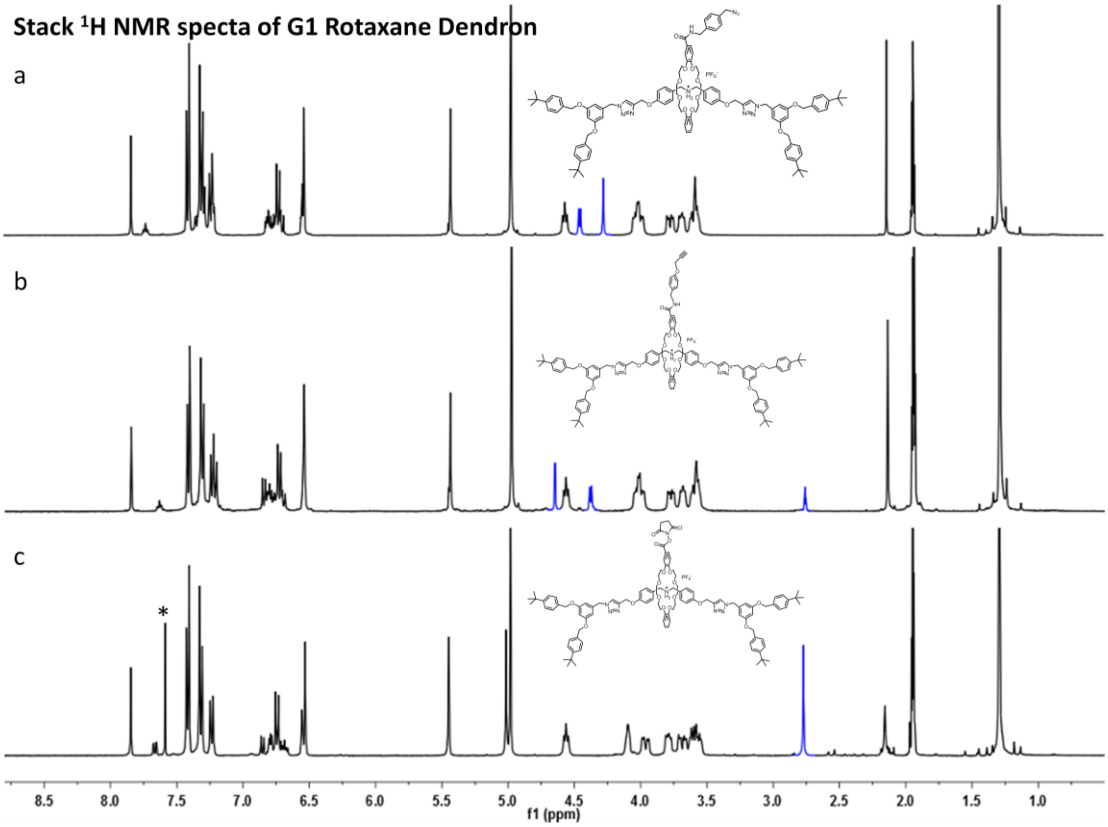

Supplementary Figure 26: Stacked ^{1}H NMR spectra (400 MHz, CD_3CN) of a) G1 [2]rotaxane dendron azide, b) G1 [2]rotaxane dendron acetylene, and c) G1 [2]rotaxane dendron NHS. The peaks highlight as blue corresponding to the characteristic peaks. Asterisk: solvent residue signal.

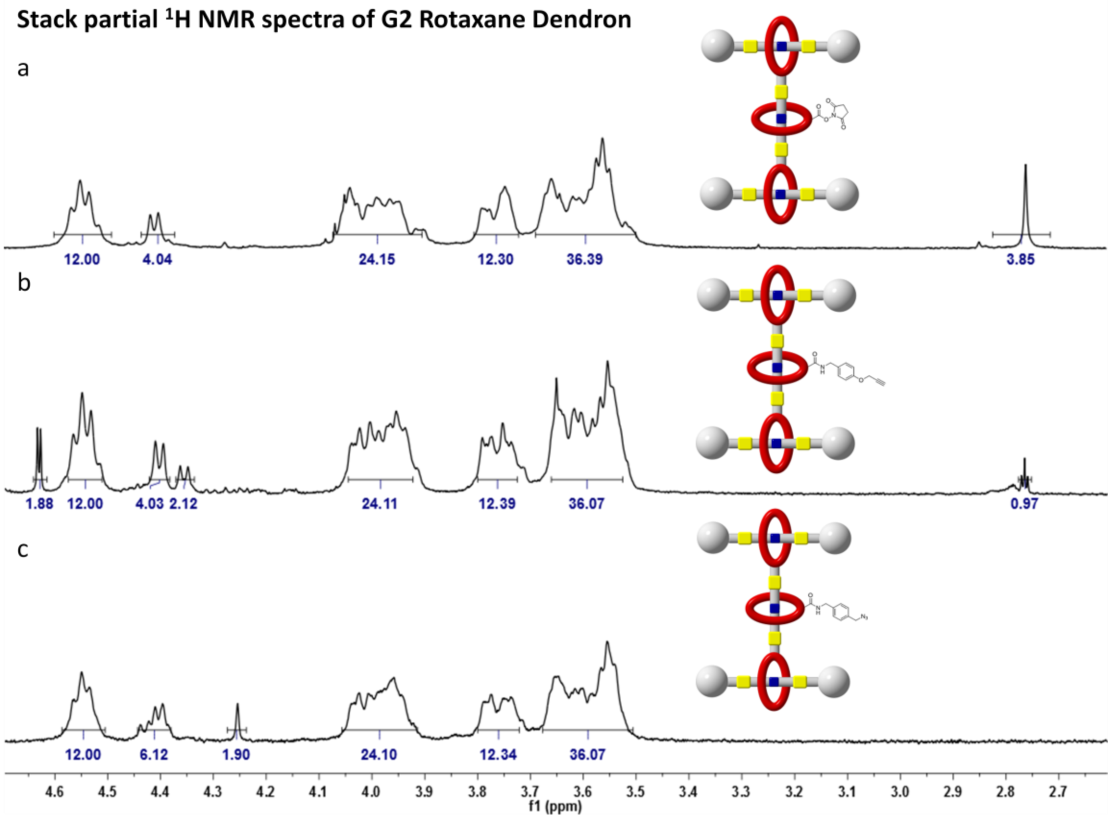

Supplementary Figure 27: Stacked partial ^{1}H NMR spectra (400 MHz, CD_3CN) of a) G2 [4]rotaxane dendron NHS, b) G2 [4]rotaxane dendron acetylene, and c) G2 [4]rotaxane dendron azide.

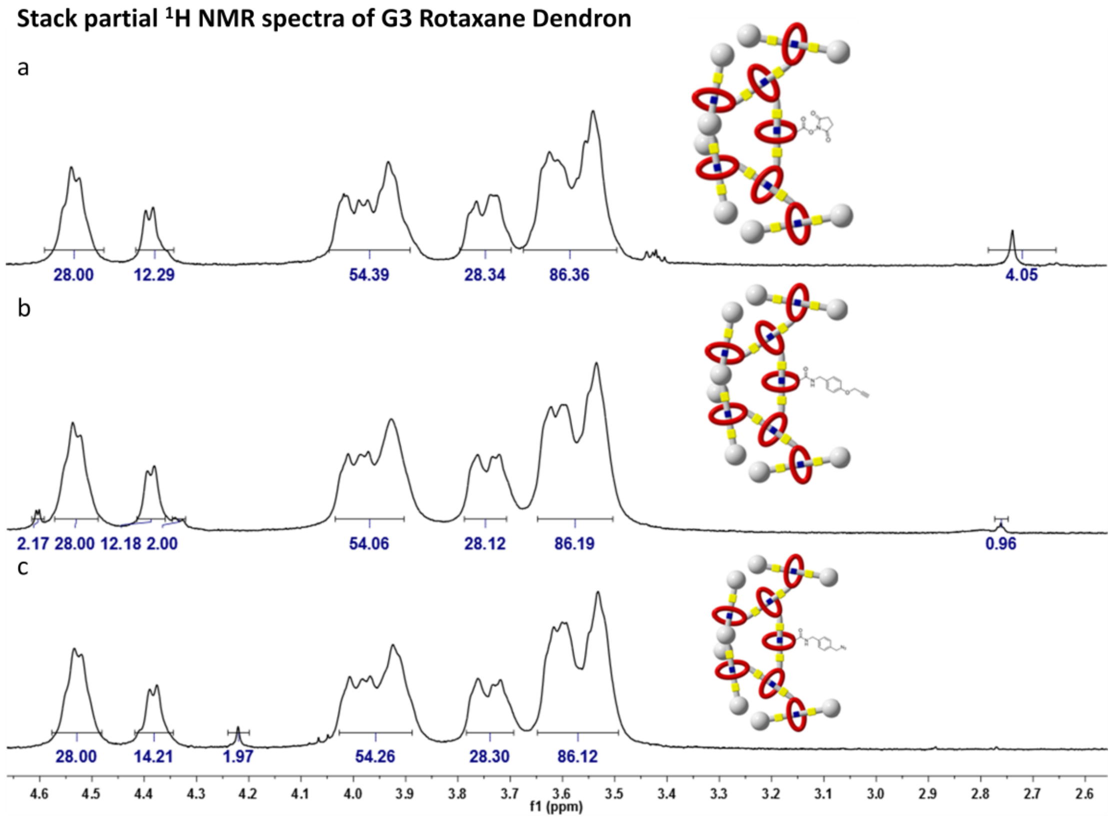

Supplementary Figure 28: Stacked partial ^{1}H NMR spectra (400 MHz, CD_3CN) of a) G3 [8]rotaxane dendron NHS, b) G3 [8]rotaxane dendron acetylene, and c) G3 [8]rotaxane dendron azide.

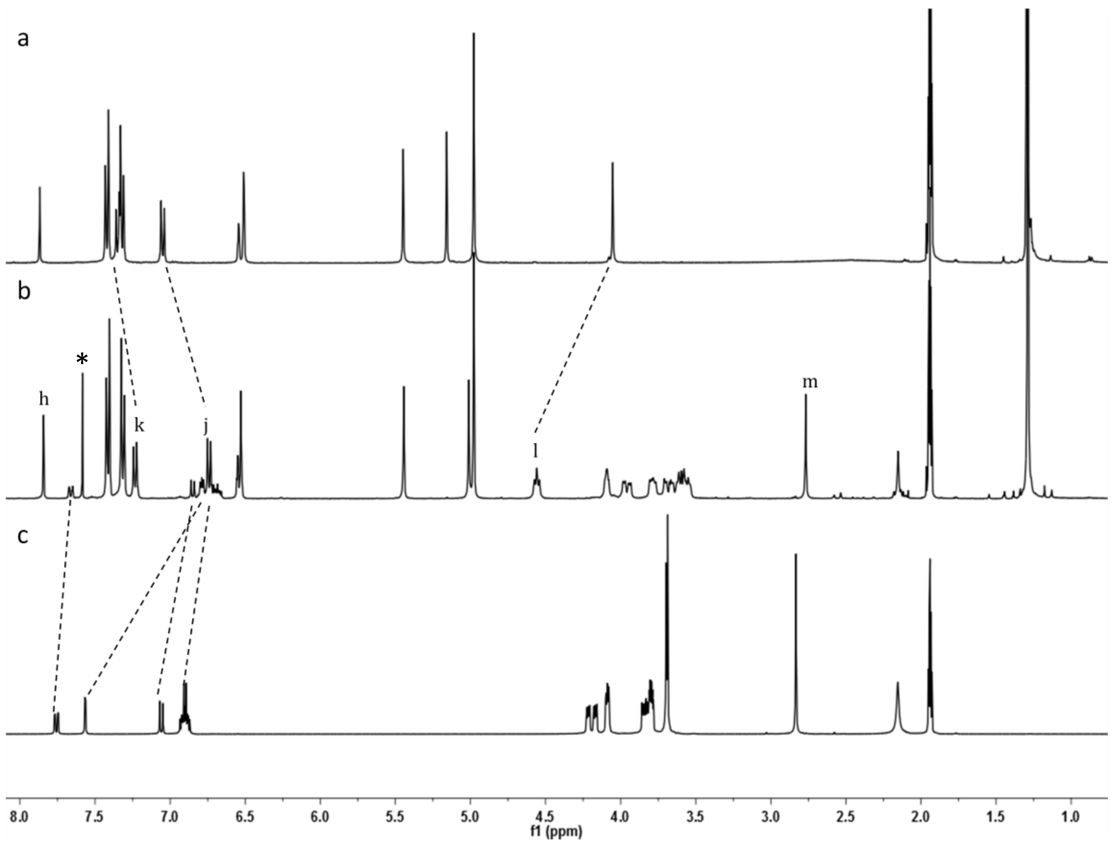

Supplementary Figure 29: Stacked partial ^{1}H NMR spectra (400 MHz, CD_3CN) of a) **S19**, b) G1 [2]rotaxane dendron-NHS, and c) DB24C8-NHS. Asterisk: solvent residue signal.

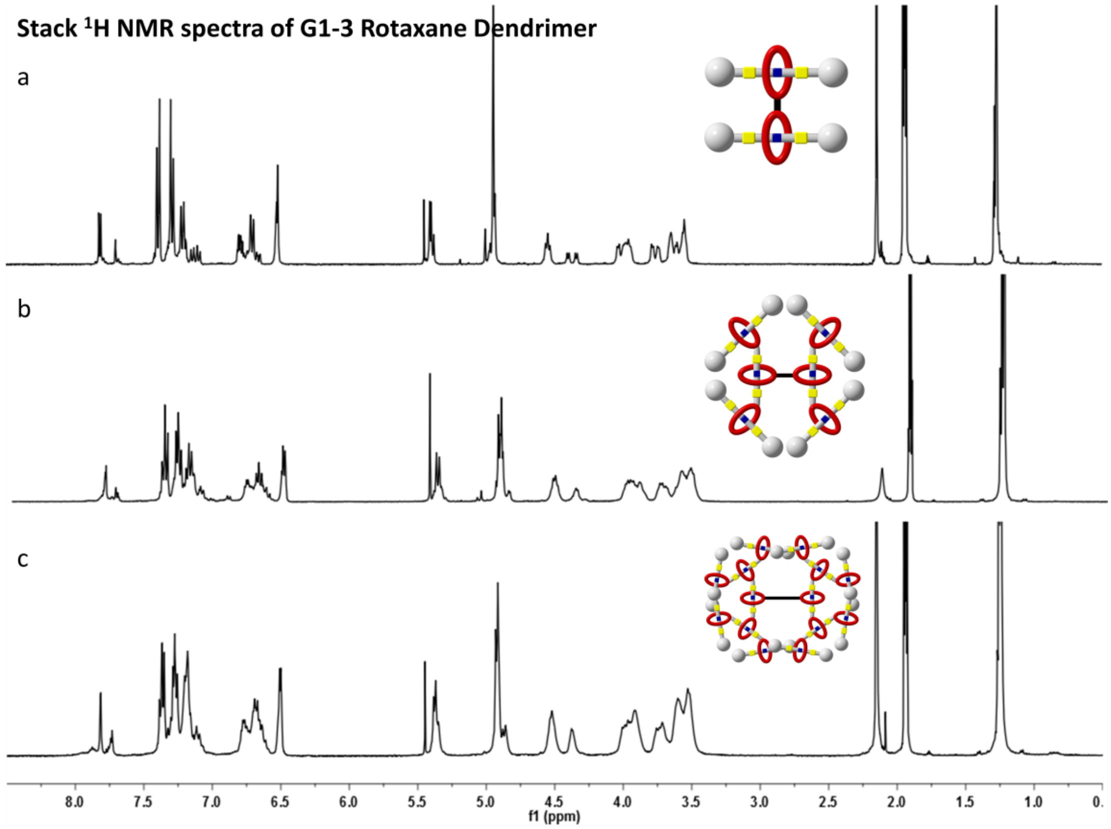

Supplementary Figure 30: Stacked ^{1}H NMR spectra (400 MHz, CD_3CN) of a) G1 [3]rotaxane dendrimer, b) G3 [7]rotaxane dendrimer, and c) G3 [15]rotaxane dendrimer.

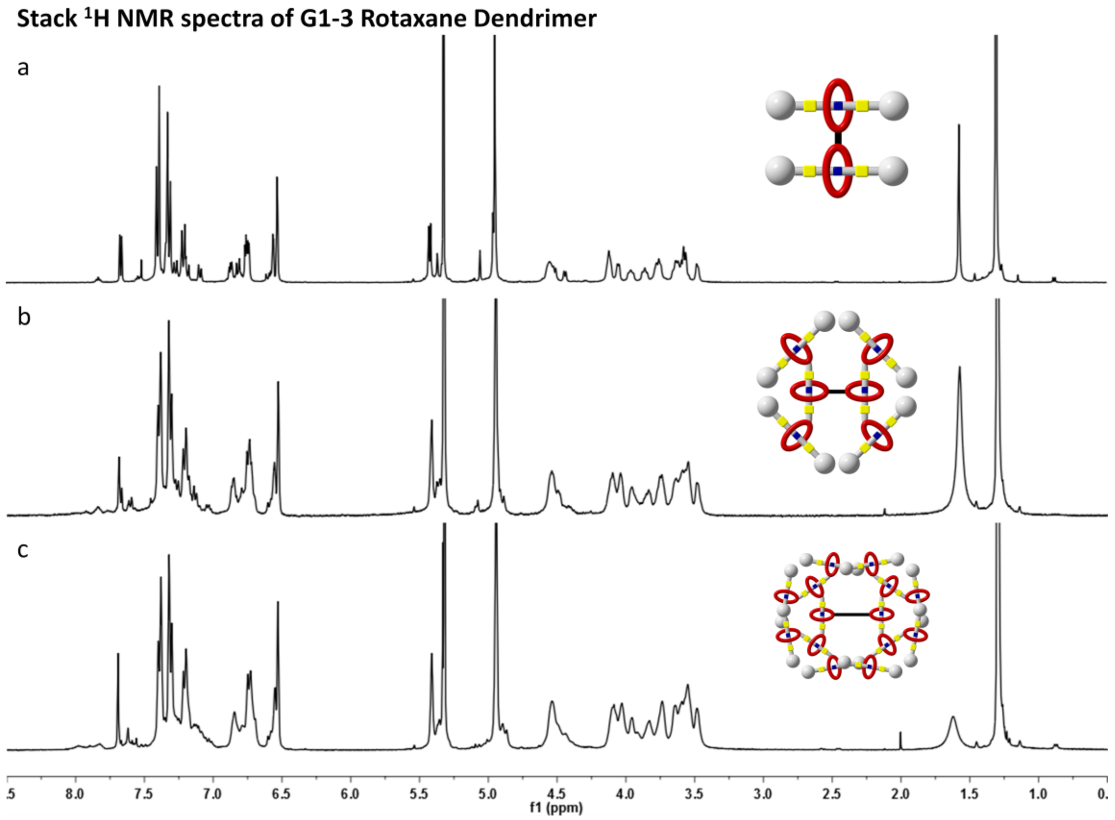

Supplementary Figure 31: Stacked ^{1}H NMR spectra (400 MHz, CD_2Cl_2) of a) G1 [3]rotaxane dendrimer, b) G3 [7]rotaxane dendrimer, and c) G3 [15]rotaxane dendrimer.

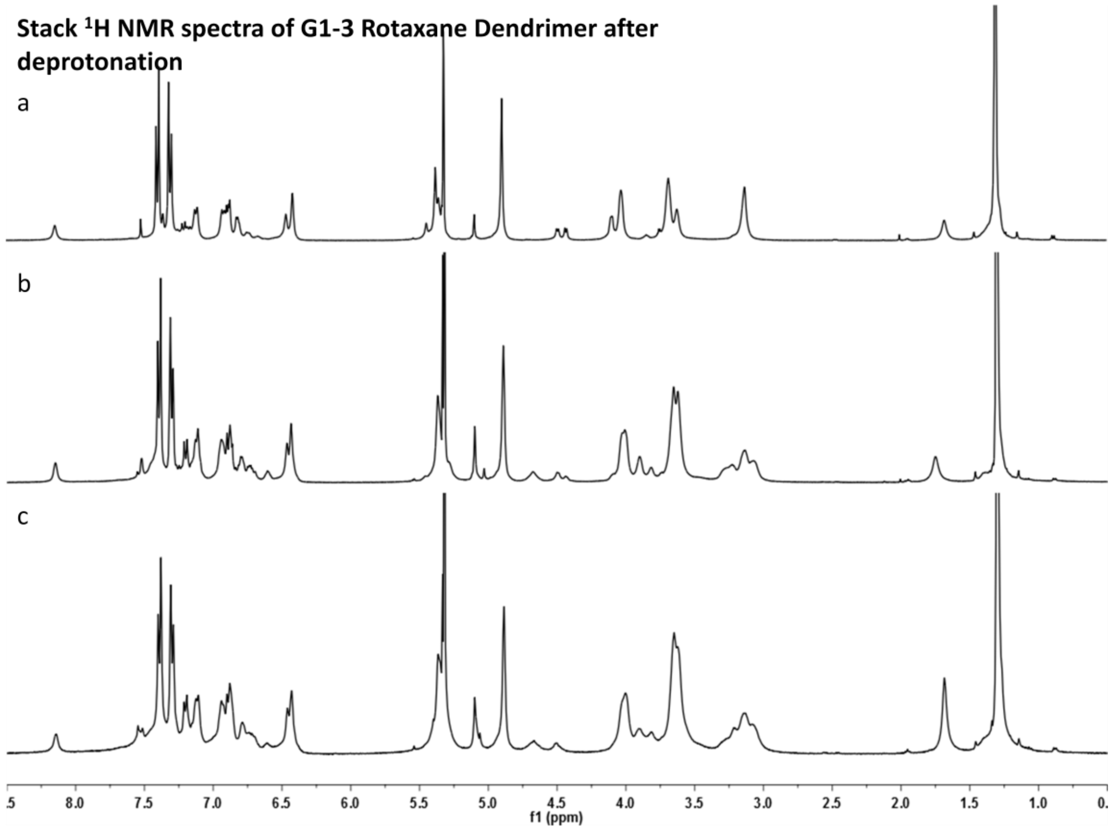

Supplementary Figure 32: Stacked ^{1}H NMR spectra (400 MHz, CD_2Cl_2) of a) Neutral G1 [3]rotaxane dendrimer, b) Neutral G2 [7]rotaxane dendrimer, and c) Neutral G3 [15]rotaxane.

Diffusion-ordered spectroscopy (DOSY) 2D NMR

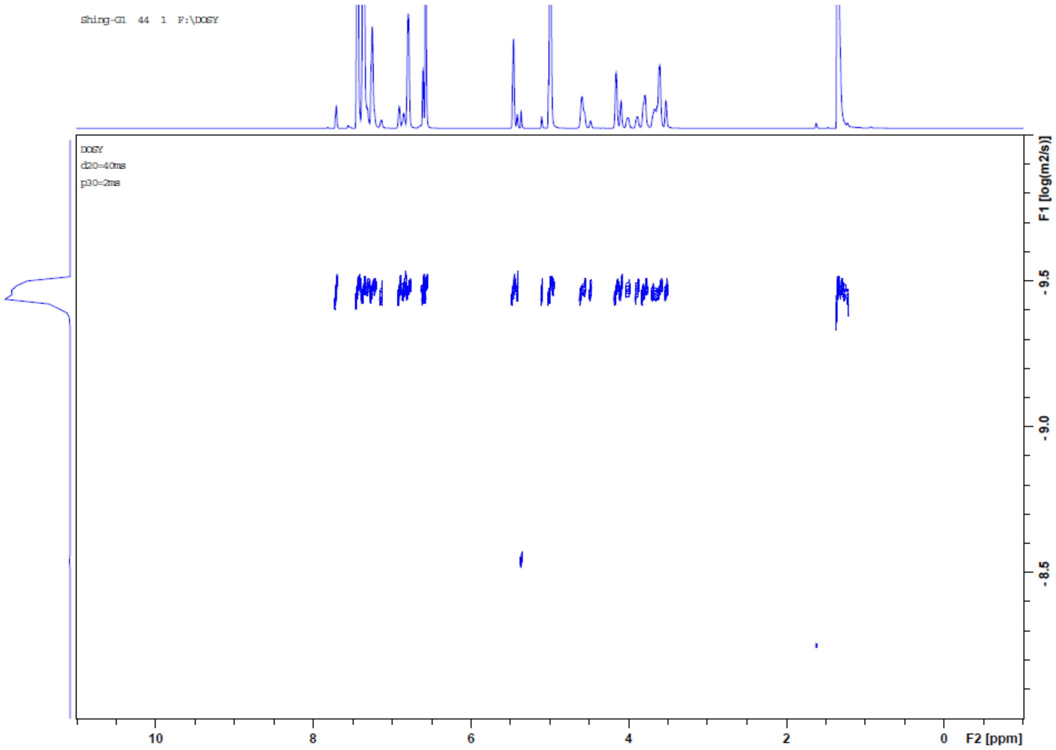

Supplementary Figure 33: DOSY NMR spectrum (500 MHz, CD_2Cl_2, 298 K) of G1 [3]rotaxane dendrimer.

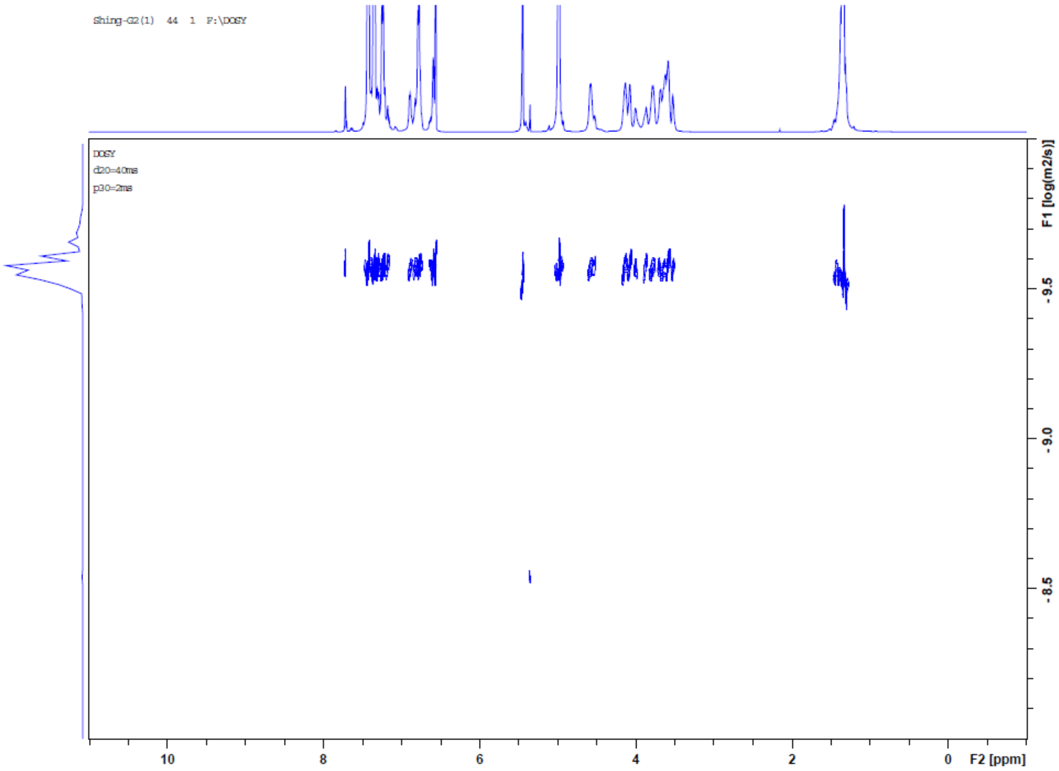

Supplementary Figure 34: DOSY NMR spectrum (500 MHz, CD_2Cl_2, 298 K) of G2 [7]rotaxane dendrimer.

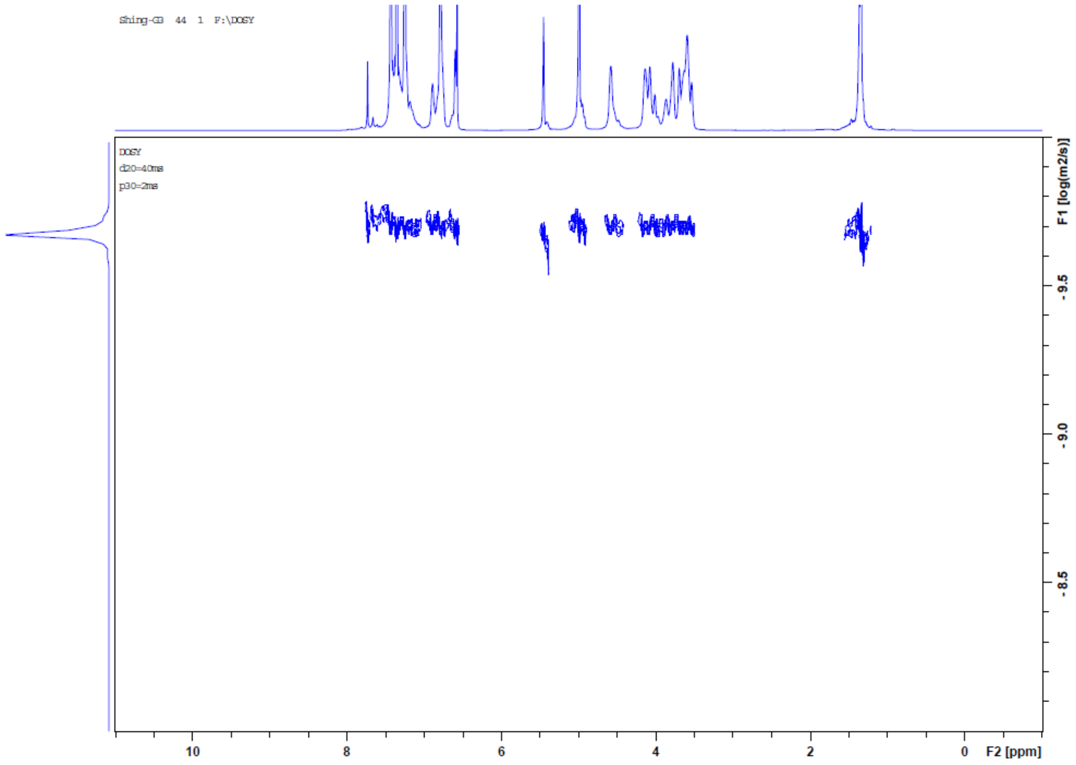

Supplementary Figure 35: DOSY NMR spectrum (500 MHz, CD_2Cl_2, 298 K) of G3

[15]rotaxane dendrimer.

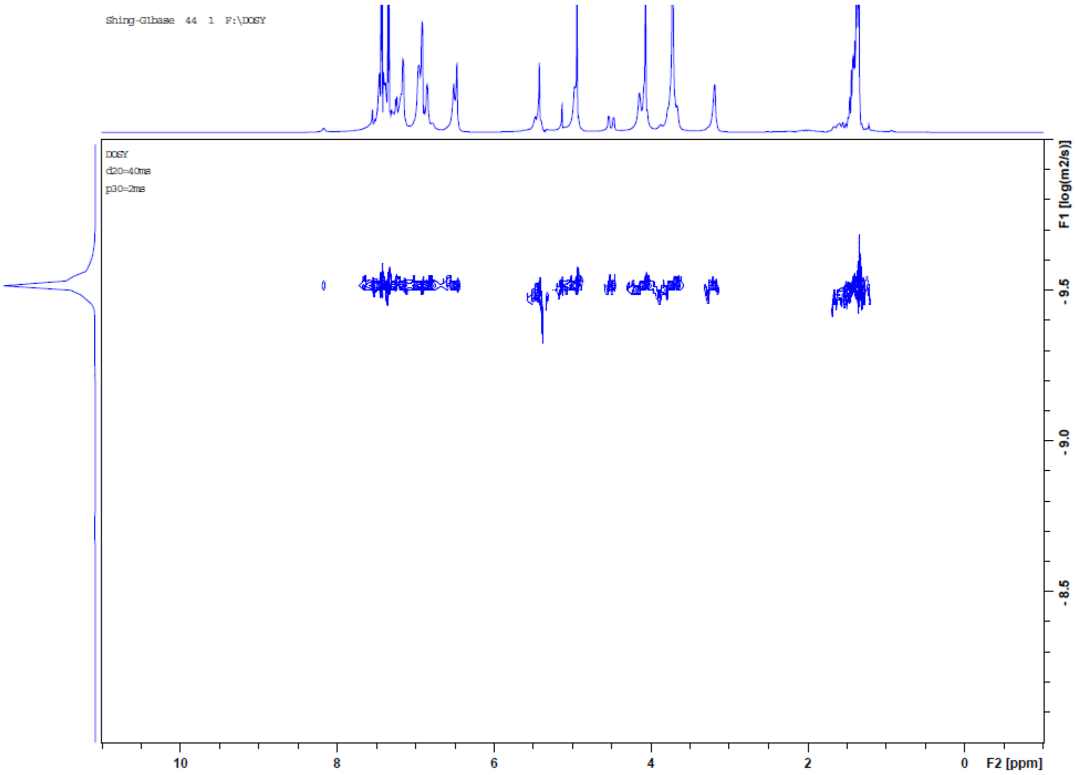

Supplementary Figure 36: DOSY NMR spectrum (500 MHz, CD_2Cl_2, 298 K) of Neutral G1 [3]rotaxane dendrimer.

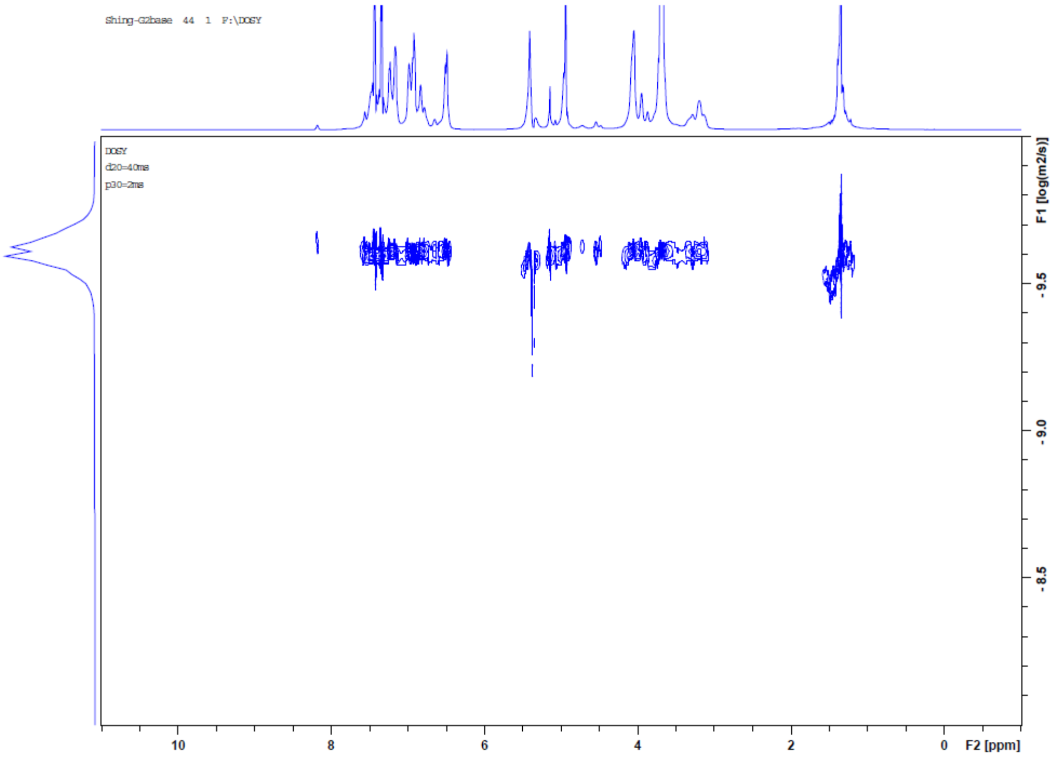

Supplementary Figure 37: DOSY NMR spectrum (500 MHz, CD_2Cl_2, 298 K) of Neutral G2 [7]rotaxane dendrimer.

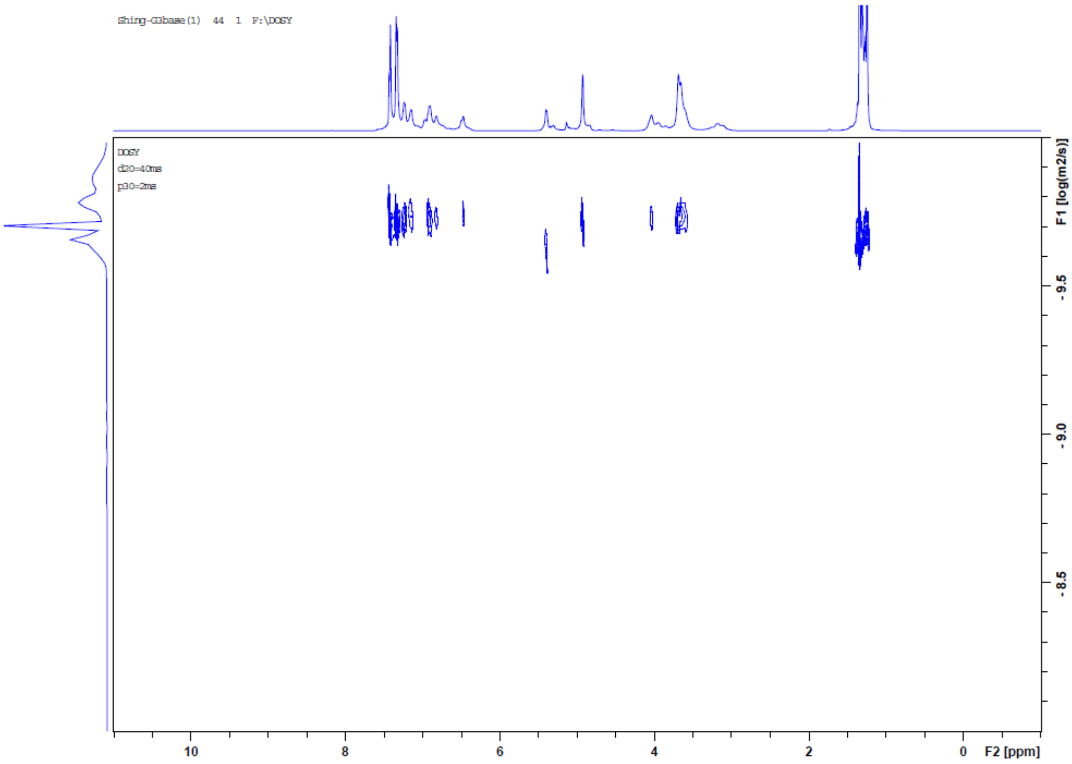

Supplementary Figure 38: DOSY NMR spectrum (500 MHz, CD_2Cl_2, 298 K) of Neutral G3 [15]rotaxane dendrimer.

Supplementary Table 1 The summary of ionic and neutral G1–3 rotaxane dendrimers diffusion coefficient and radius/diameter calculated from Stokes-Einstein equation.

G(n) rotaxane dendrimer	Diffusion coefficient (m^2/s)	Radius (nm)	Diameter (nm)
**G1**	3.42×10^{-10}	0.78	1.56
**G2**	2.69×10^{-10}	0.99	1.98
**G3**	1.98×10^{-10}	1.35	2.64
**Neutral G1**	3.05×10^{-10}	0.87	1.74
**Neutral G2**	2.44×10^{-10}	1.09	2.18
**Neutral G3**	1.79×10^{-10}	1.49	2.98

Nuclear Overhauser Effect Spectroscopy (NOESY) 2D NMR

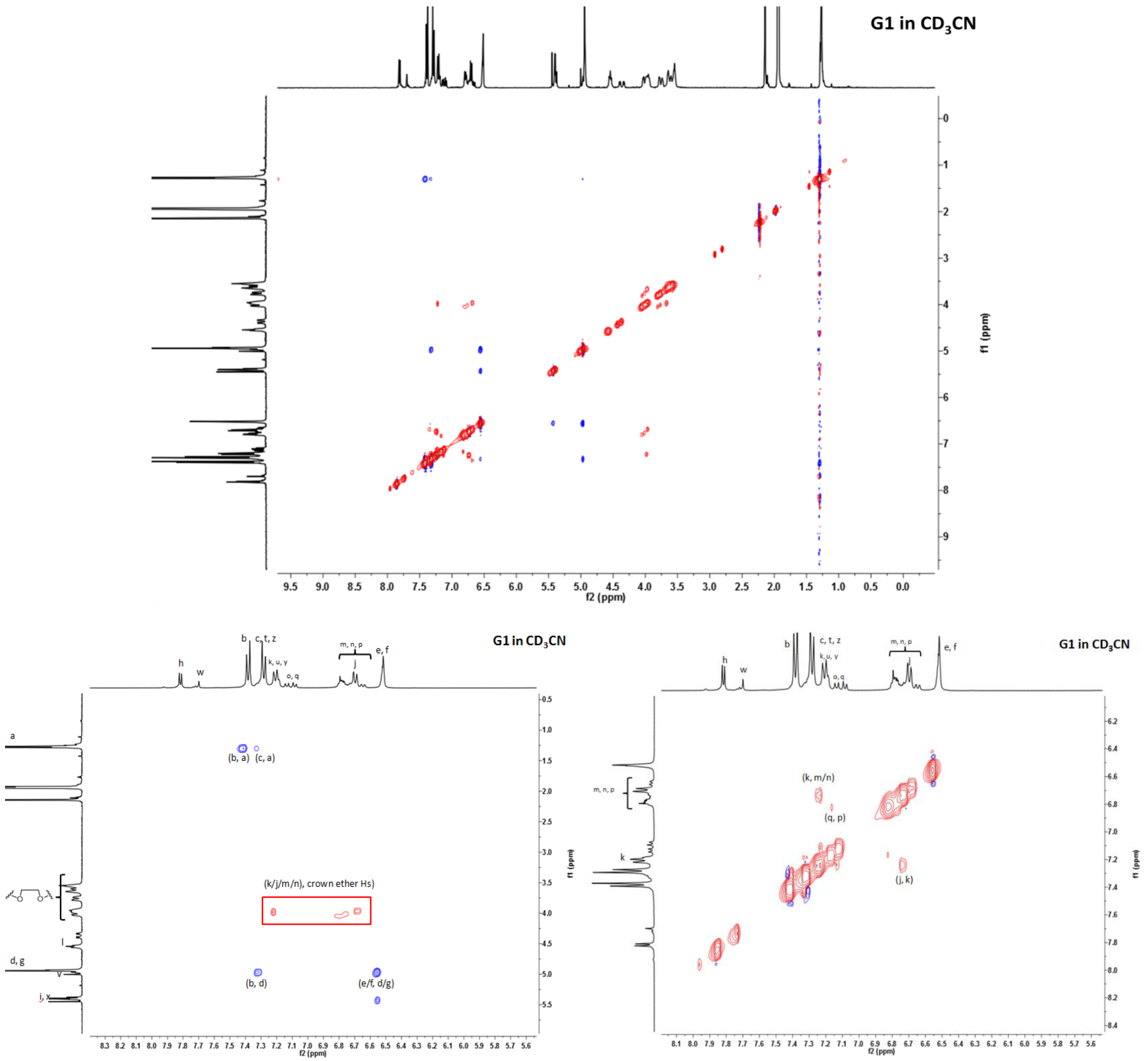

Supplementary Figure 39: NOESY NMR spectrum (400 MHz, CD_3CN, 298 K) of G1 [3]rotaxane dendrimer.

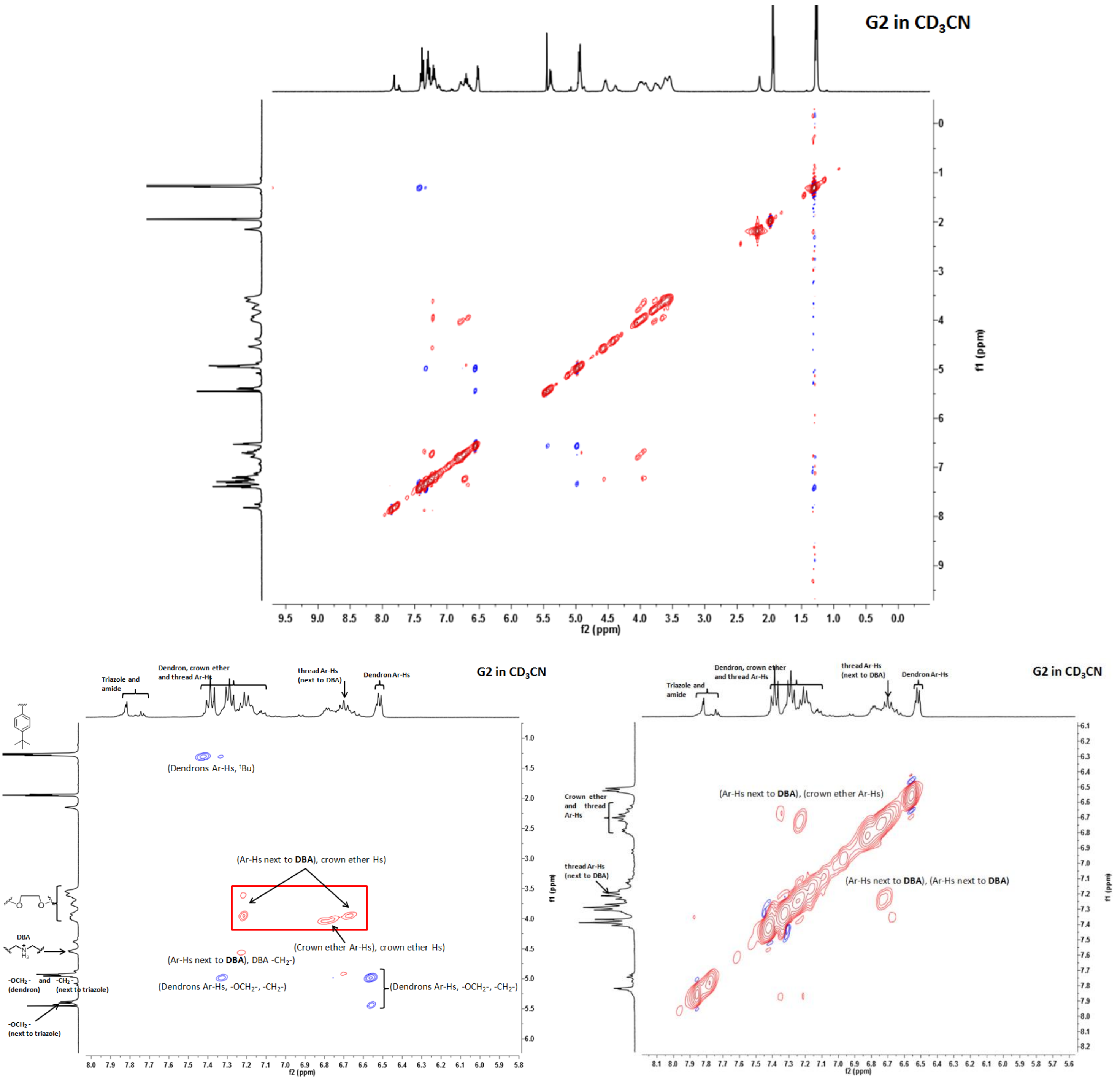

Supplementary Figure 40: NOESY NMR spectrum (400 MHz, CD_3CN, 298 K) of G2 [7]rotaxane dendrimer.

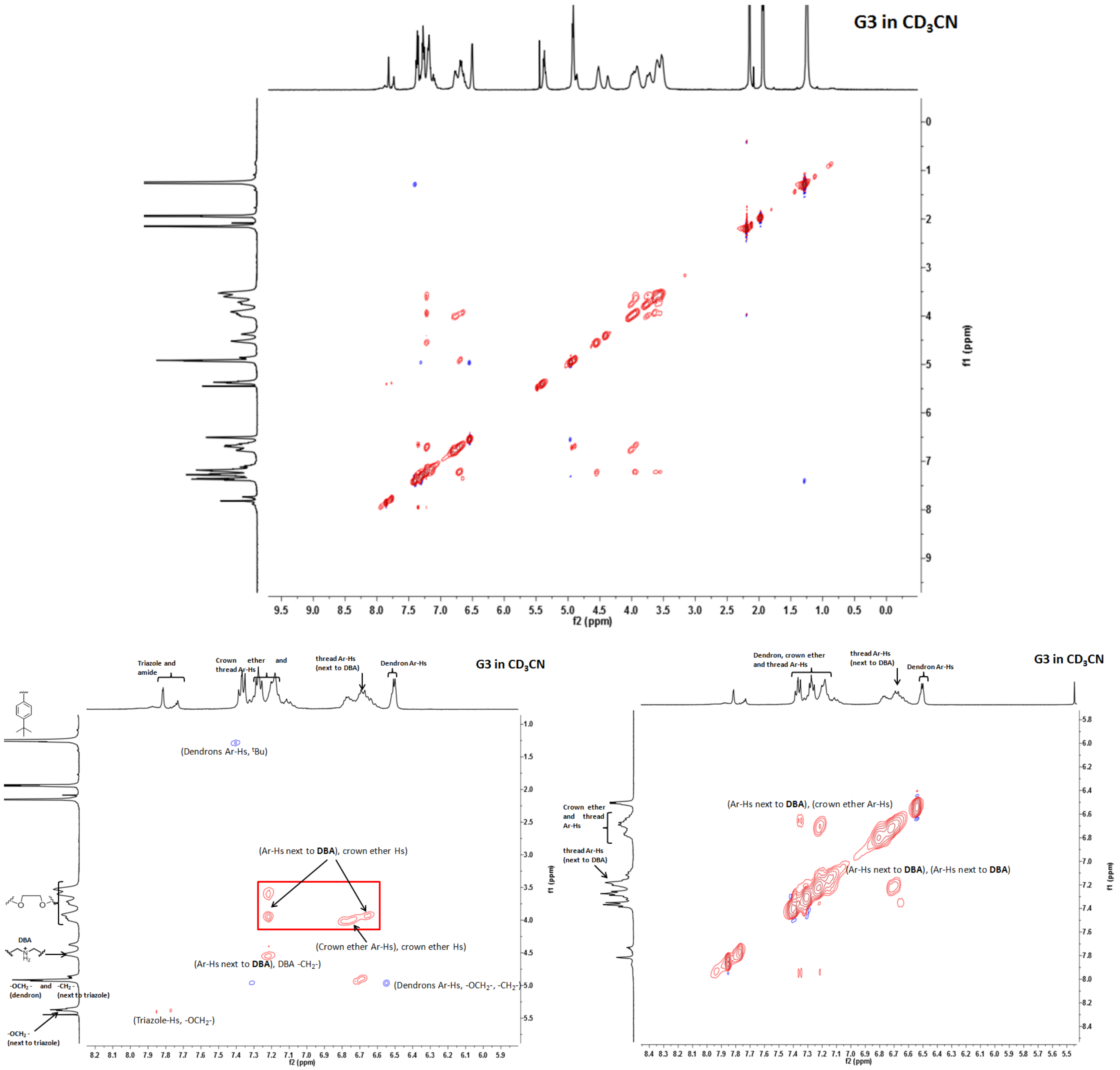

Supplementary Figure 41: NOESY NMR spectrum (400 MHz, CD_3CN, 298 K) of G3 [15]rotaxane dendrimer.

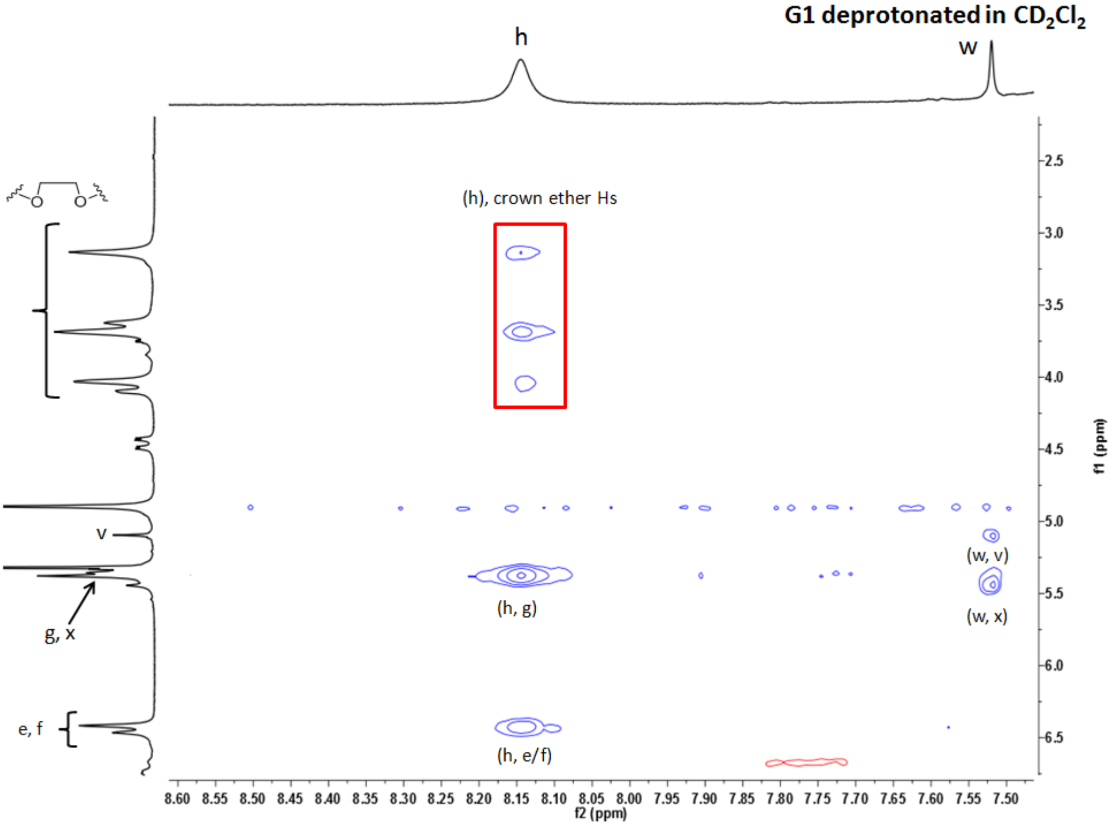

Supplementary Figure 42: Partial NOESY NMR spectrum (400 MHz, CD_2Cl_2 298 K) of Neutral G1 [3]rotaxane dendrimer.

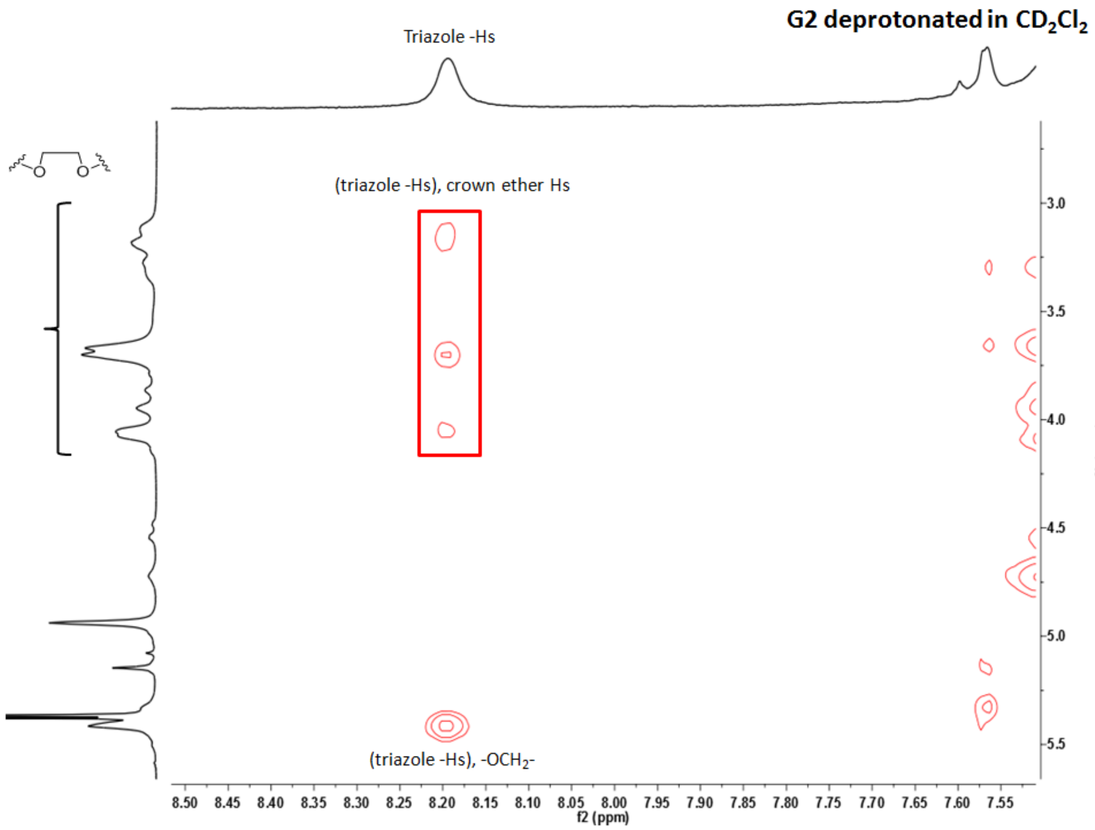

Supplementary Figure 43: Partial NOESY NMR spectrum (400 MHz, CD_2Cl_2, 298 K) of Neutral G2 [7]rotaxane dendrimer.

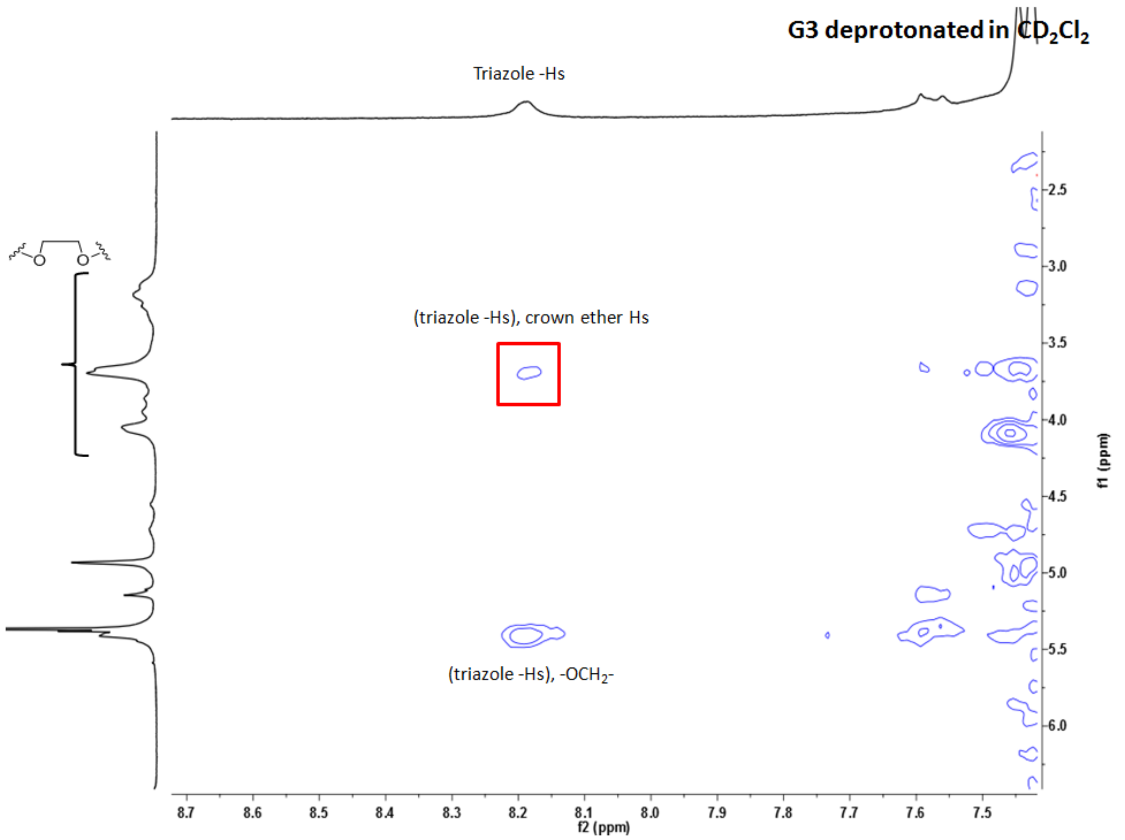

Supplementary Figure 44: Partial NOESY NMR spectrum (400 MHz, CD_2Cl_2, 298 K) of Neutral G3 [15]rotaxane dendrimer.

Computational Study

We employ the first-principles computational tool DFTB+[6] (which adopts the density functional based tight binding approach[7]) to calculate molecular structures and compare them with the experimental observations of molecular size. The Lennard-Jones dispersion model[8] implemented in the DFTB+ code is used to describe the intermolecular interactions. Due to the molecular complexity and near-infinite possibilities for optimizing their structures, we present partially optimized results here. It can be expected that further optimization would fold the resulting molecular structures more tightly than presented here.

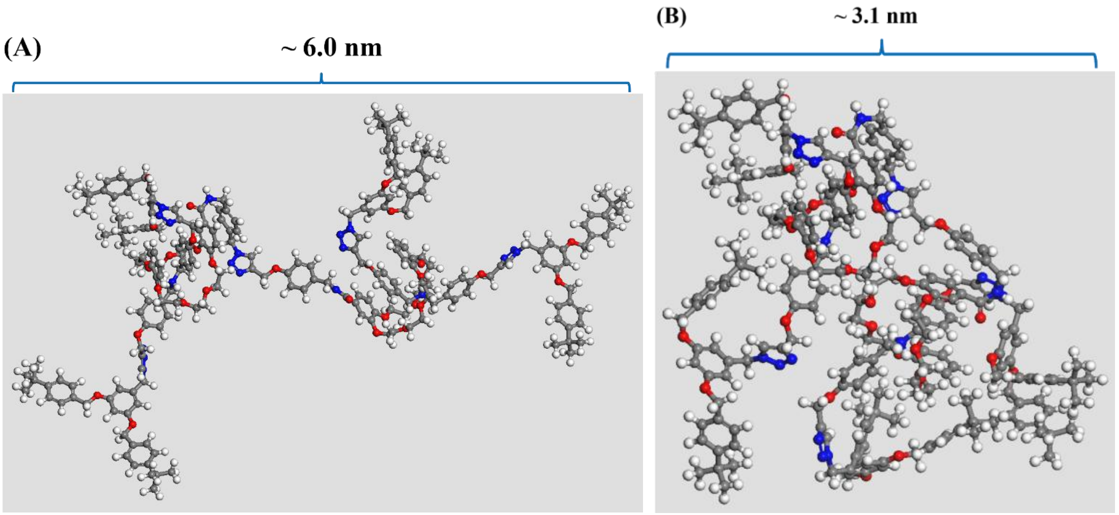

Supplementary Figure 45: Illustration of calculated structures of G1 T3B-RD and their sizes: a) fully extended; b) partially folded.

We may take as an example the structure of G1 T3B-RD (Supplementary Figure 45): the maximum size of the fully extended molecule is ~6.0 nm, while the partially-folded G1 T3B-RD has a diameter of ~3.1 nm. This partially-folded calculated diameter is larger than the experimental result, indicating that the actual structure of G1 T3B-RD is likely tightly folded into a 3-D globular structure in solution.

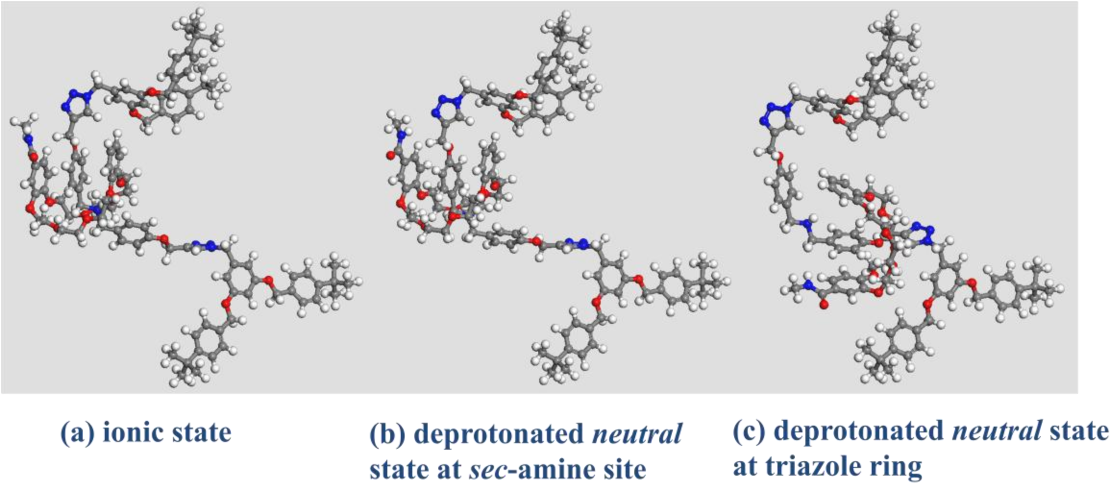

Supplementary Figure 46: Optimized truncated structures of [2]rotaxane in different states: a) ionic state; b) deprotonated neutral state at the *sec*-amine site; c) deprotonated neutral state at the triazole ring.

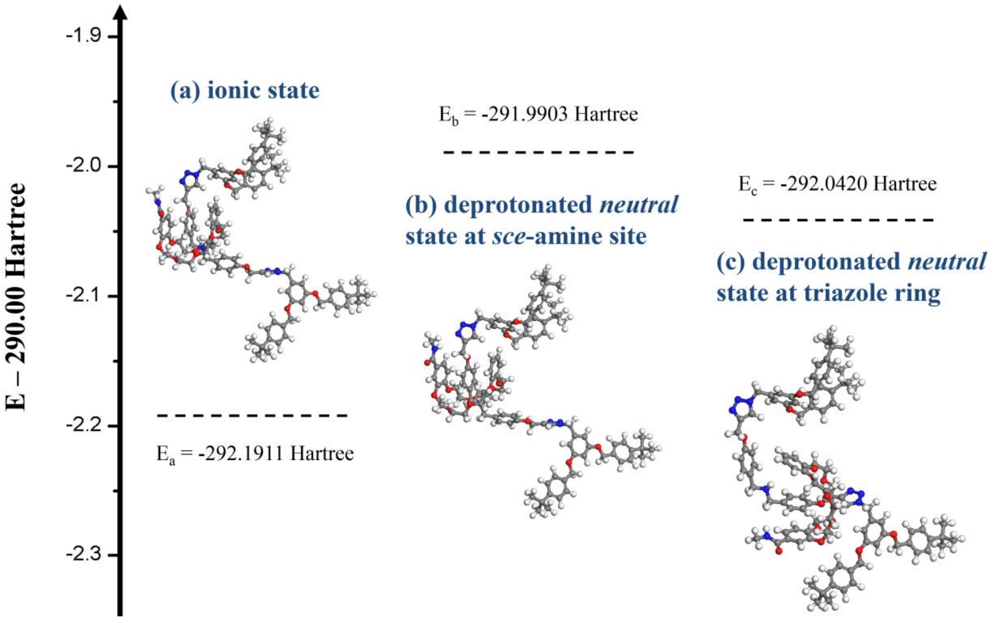

Supplementary Figure 47: Energy diagram of three optimized truncated structures of [2]rotaxane in different states.

To give support for the ability of triazole to bind with DB24C8, a simplified truncated [2]rotaxane model (based on our T3B-RD design and retaining the relevant segments) was used for computational study. In the three different states shown in Supplmentry Figure 47, the calculated total energies are, respectively: a) -292.1911 Hartree in an ionic state, b) -291.9903 Hartree in a deprotonated neutral state at the sec-amine site, and c) -292.0420 Hartree in a deprotonated neutral state at the triazole ring. In the

ionic state, the geometry optimization result showed that the DB24C8 robustly binds to the DBA, with the lowest total energy. Conversely, in the deprotonated neutral state, the DB24C8 tends to bind with the triazole ring, with lower total energy compared with the sec-amine site, which is consistent with the T3B-RDs ^{1}H NMR studies.

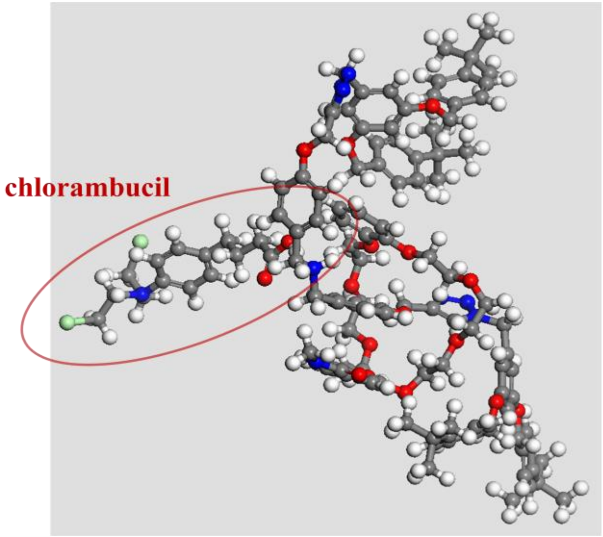

Supplementary Figure 48: Optimized truncated structures of neutral [2]rotaxane in the presence of a chlorambucil molecule (highlighted with the red ellipse) which binds to the DBA and expels the macrocycle to the triazole.

We also conducted a computation in the presence of the guest chlorambucil molecule. The geometry optimization shows that the chlorambucil will bind with the DBA robustly and the macrocycle is thus expelled to the triazole site (Supplementary Figure 48). The electrostatic interaction between the DBA and carboxylate (chlorambucil) is stronger than the intra-molecular interaction (hydrogen bonding) with DB24C8. As a consequence, the existence of the chlorambucil prohibits the shuttling of the macrocycle between two triazole rings.

Dynamic Light Scattering (DLS)

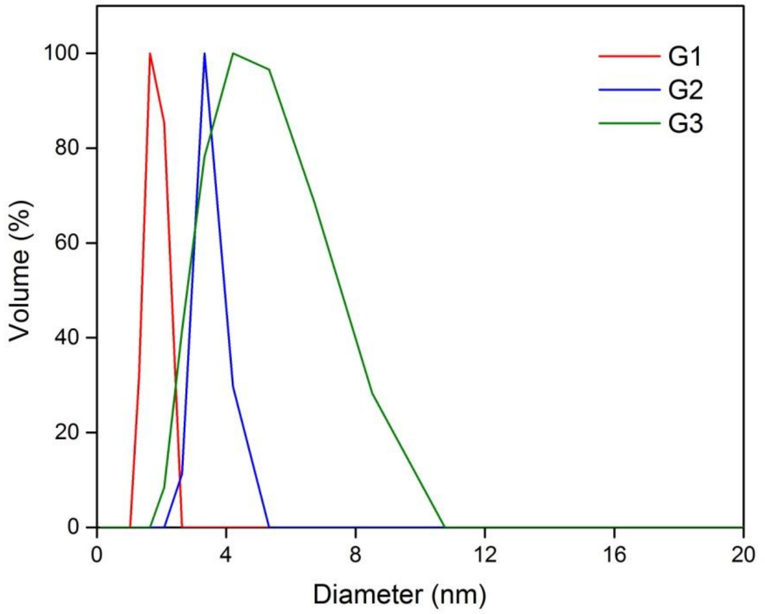

Supplementary Figure 49: Dynamic light scattering (DLS) diagram of G1–G3 in CH_2Cl_2.

Supplementary Table 2: The hydrodynamic diameter measurement of G1–G3 from DLS.

G(n) rotaxane dendrimer	Diameter (nm)
**G1**	1.80 ± 0.14
**G2**	3.50 ± 0.21
**G3**	4.57 ± 0.81

Atomic Force Microscopy (AFM)

General procedure of preparing AFM samples: 0.001% w/w dendrimer in THF solution was prepared and that the dendrimer molecules were deposited on the mica surface through a spin coating. Tapping mode AFM was performed.

Other surface such as highly oriented pyrolytic graphite (HOPG) cannot give satisfactory result, due to the hydrophobic surface make the dendrimer-dendrimer interaction stronger than dendrimer-surface interaction.[10]

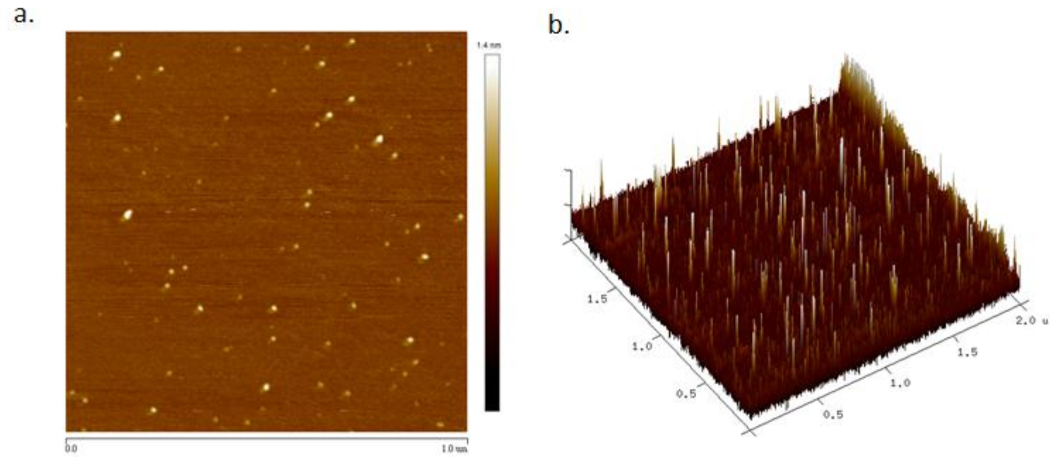

Supplementary Figure 50: a) AFM image of G1 [3]rotaxane dendrimers on mica surface (1 μm x 1 μm) and b) 3-D surface plot of G1 [3]rotaxane dendrimers (2 μm x 2 μm).

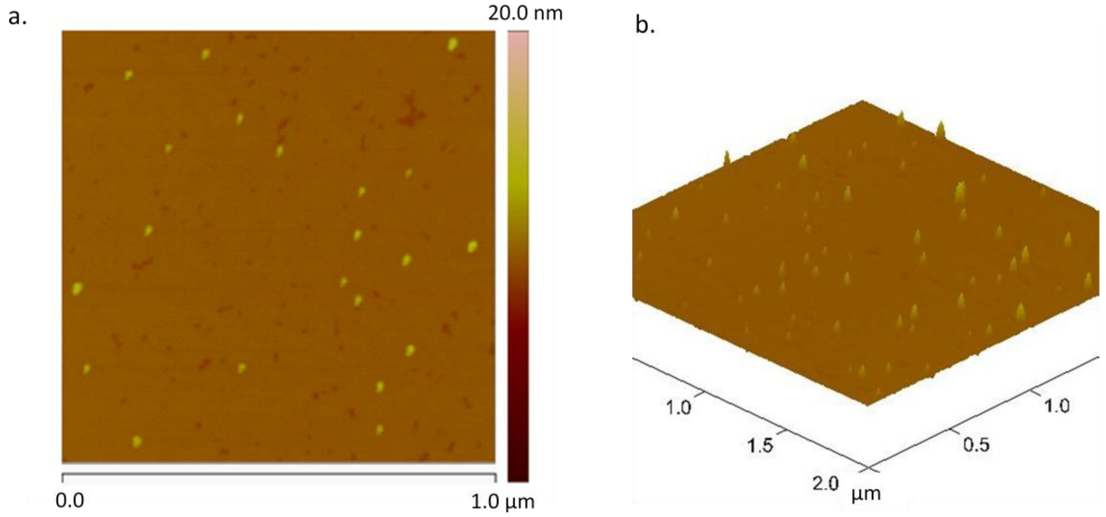

Supplementary Figure 51: a) AFM image of G2 [7]rotaxane dendrimers on mica surface (1 μm x 1 μm) and b) 3-D surface plot of G2 [7]rotaxane dendrimers (2 μm x 2 μm).

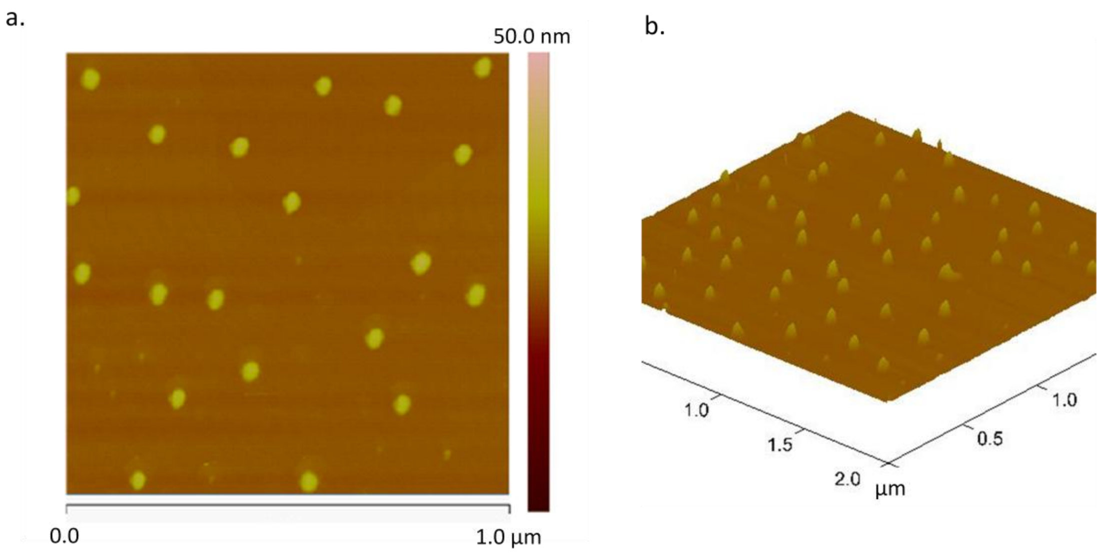

Supplementary Figure 52: a) AFM image of G3 [15]rotaxane dendrimers on mica surface (1 µm x 1 µm) and b) 3-D surface plot of G3 [15]rotaxane dendrimers (2 µm x 2 µm).

Supplementary Table 3: The average height measurement of G1–G3 rotaxane dendrimers from AFM images on mica surface.

G(n) rotaxane dendrimer	Height (nm)
**G1**	1.15 ± 0.05
**G2**	3.94 ± 0.16
**G3**	10.96 ± 0.14

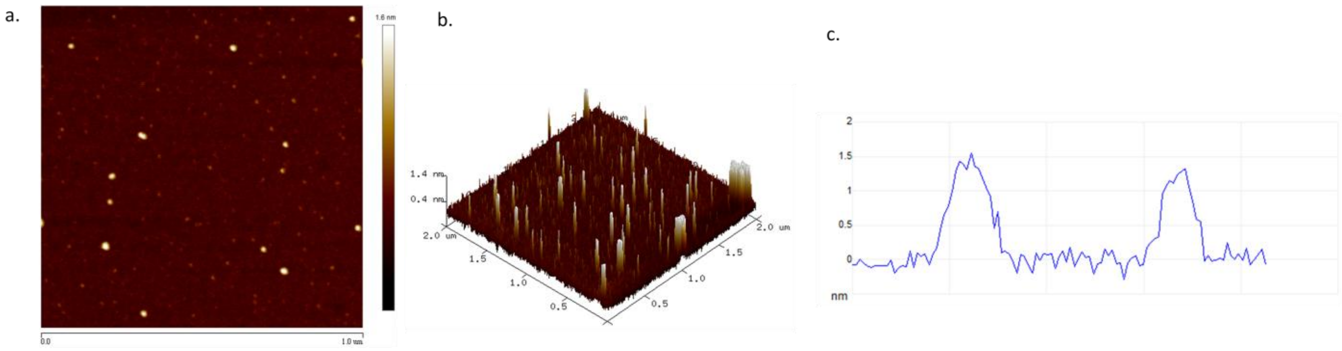

Supplementary Figure 53: a) AFM image of neutral G1 [3]rotaxane dendrimers on mica surface (1 µm x 1 µm), b) 3-D surface plot of G1 [3]rotaxane dendrimers (2 µm x 2 µm) and c) height profile of neutral G1 [3]rotaxane dendrimers.

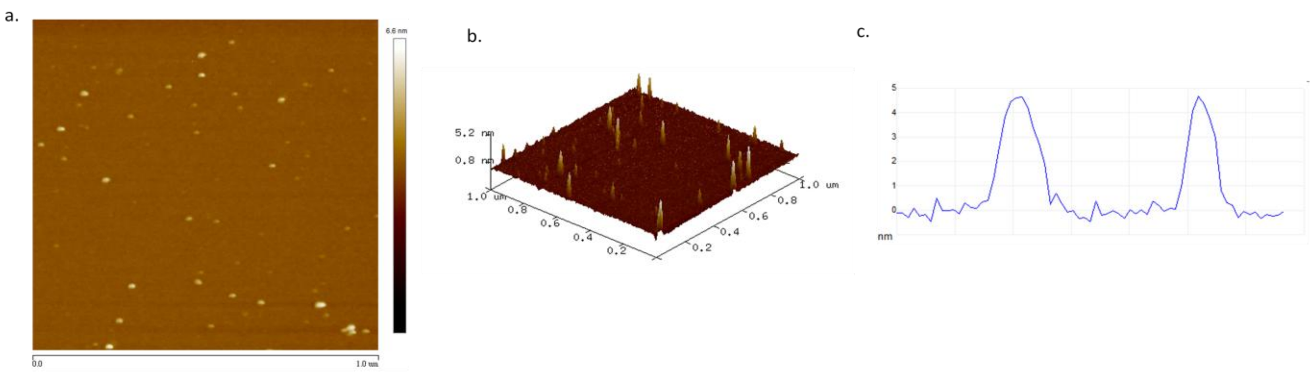

Supplementary Figure 54: a) AFM image of neutral G2 [7]rotaxane dendrimers on mica surface (1 µm x 1 µm), b) 3-D surface plot of G2 [7]rotaxane dendrimers (1 µm x 1 µm) and c) height profile of neutral G2 [7]rotaxane dendrimers.

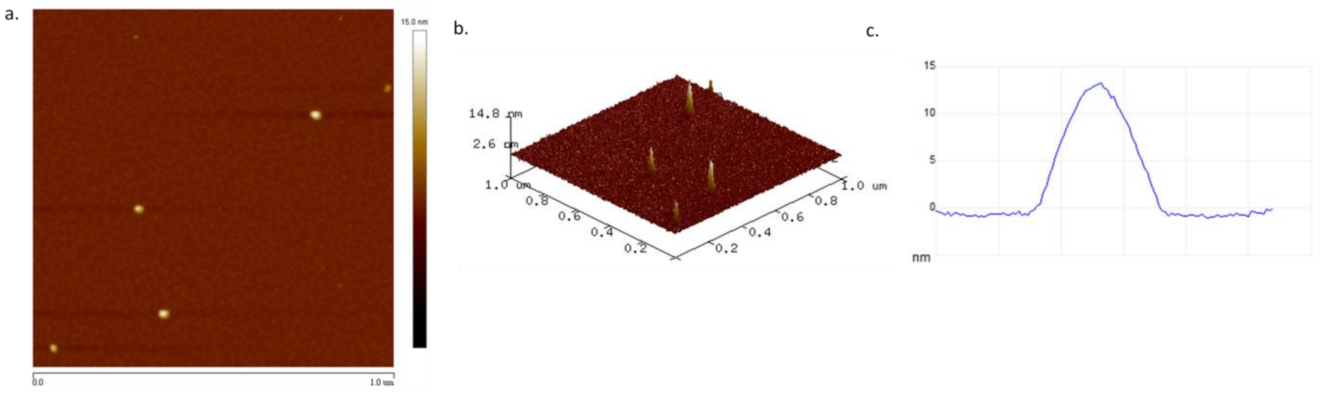

Supplementary Figure 55: a) AFM image of neutral G3 [15]rotaxane dendrimers on mica surface (1 µm x 1 µm), b) 3-D surface plot of G3 [15]rotaxane dendrimers (2 µm x 2 µm) and c) height profile of neutral G3 [15]rotaxane dendrimers.

Supplementary Table 4: The average height measurement of G1–G3 rotaxane dendrimers from AFM images on mica surface.

G(n) rotaxane dendrimer	Height (nm)
**Neutral G1**	1.38 ± 0.06
**Neutral G2**	4.81 ± 0.17
**Neutral G3**	13.25 ± 0.44

Stacked ^{1}H NMR spectra of Rotaxane Dendrimers and Neutral Rotaxane Dendrimers

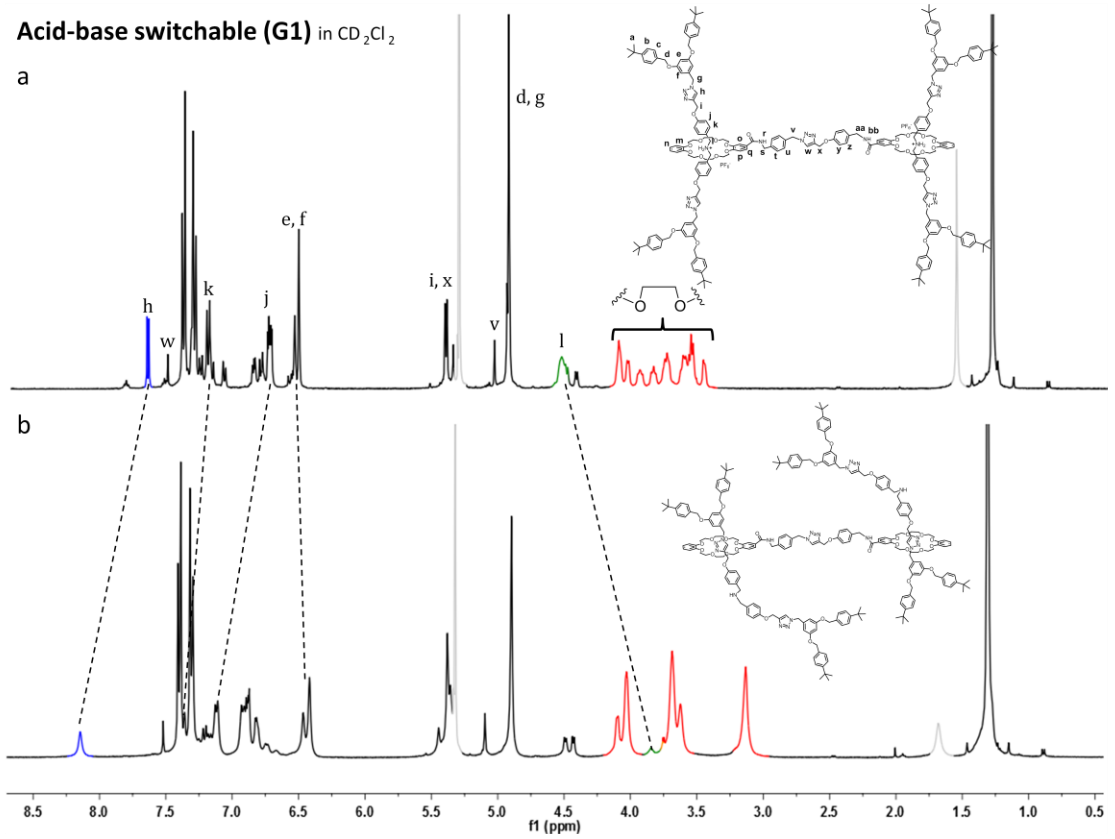

Supplementary Figure 56: Stacked ^{1}H NMR spectra (400 MHz, CD_2Cl_2) of a) G1 [3]rotaxane dendrimer and b) Neutral G1 [3]rotaxane dendrimer.

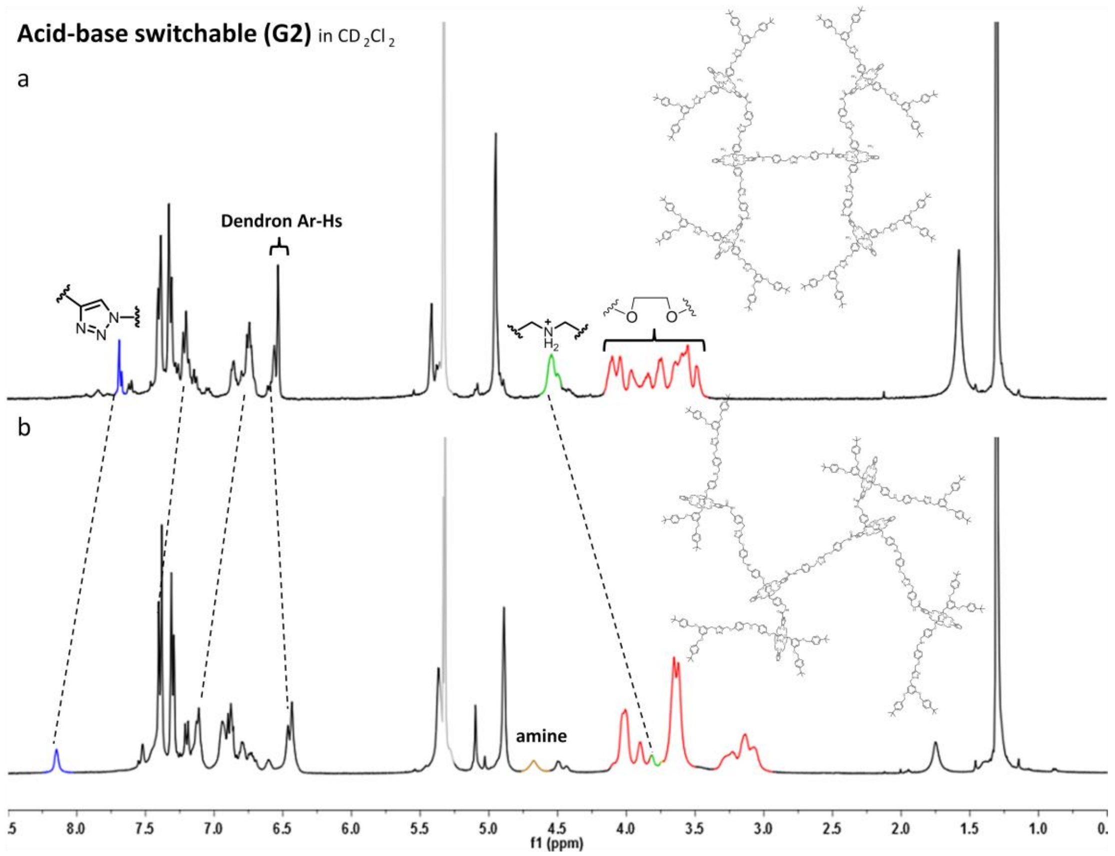

Supplementary Figure 57: Stacked ^{1}H NMR spectra (400 MHz, CD_2Cl_2) of a) G2 [7]rotaxane dendrimer and b) Neutral G2 [7]rotaxane dendrimer.

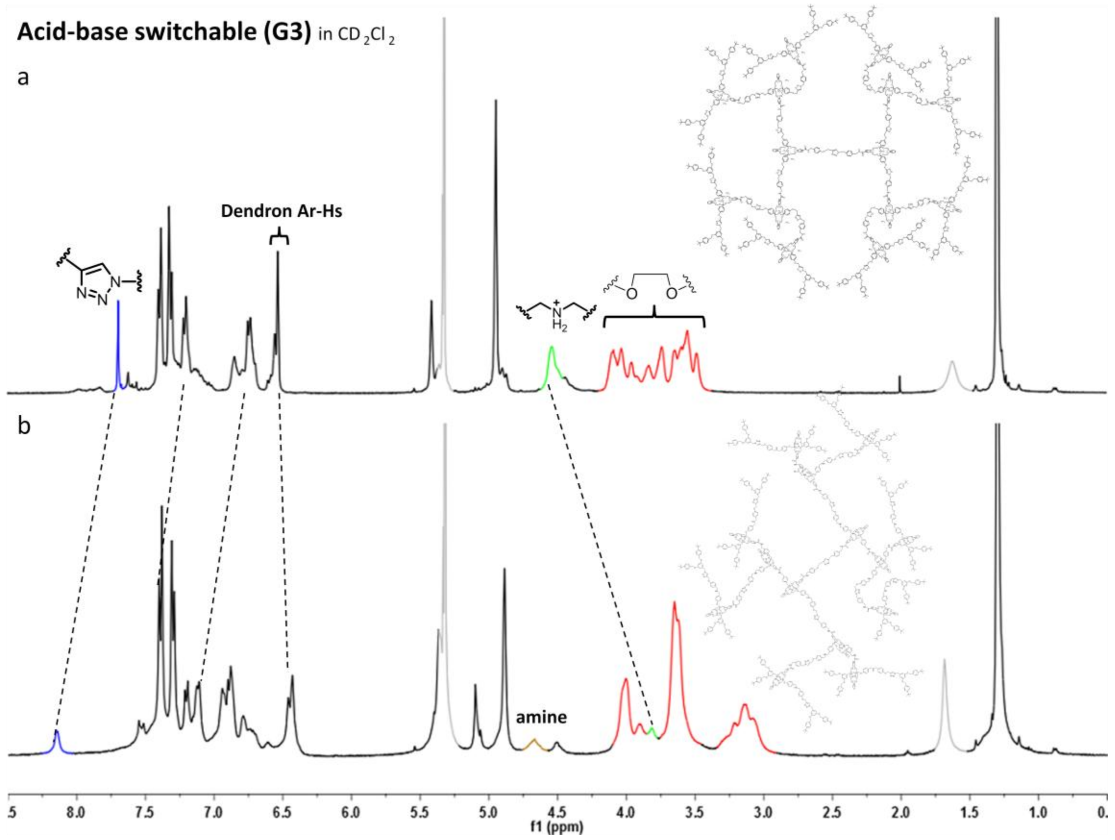

Supplementary Figure 58: Stacked ^{1}H NMR spectra (400 MHz, CD_2Cl_2) of a) G3 [15]rotaxane dendrimer and b) Neutral G15 [15]rotaxane dendrimer.

Acid-Base Switching Cycles of Rotaxane Dendrimer

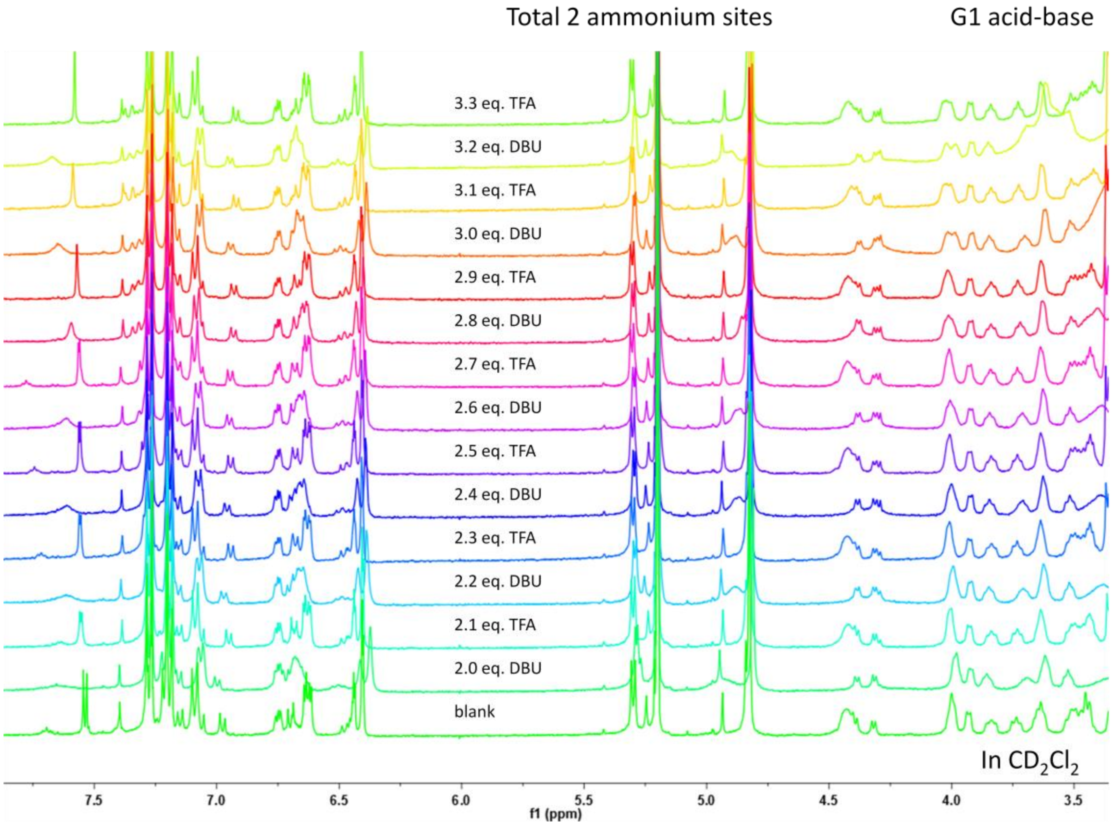

Supplementary Figure 59: Stacked ^{1}H NMR spectra (400 MHz, CD_2Cl_2) G1 [2]rotaxane dendrimer after alternatively addition of DBU and TFA with 7 cycles (Concentration: 1 mM).

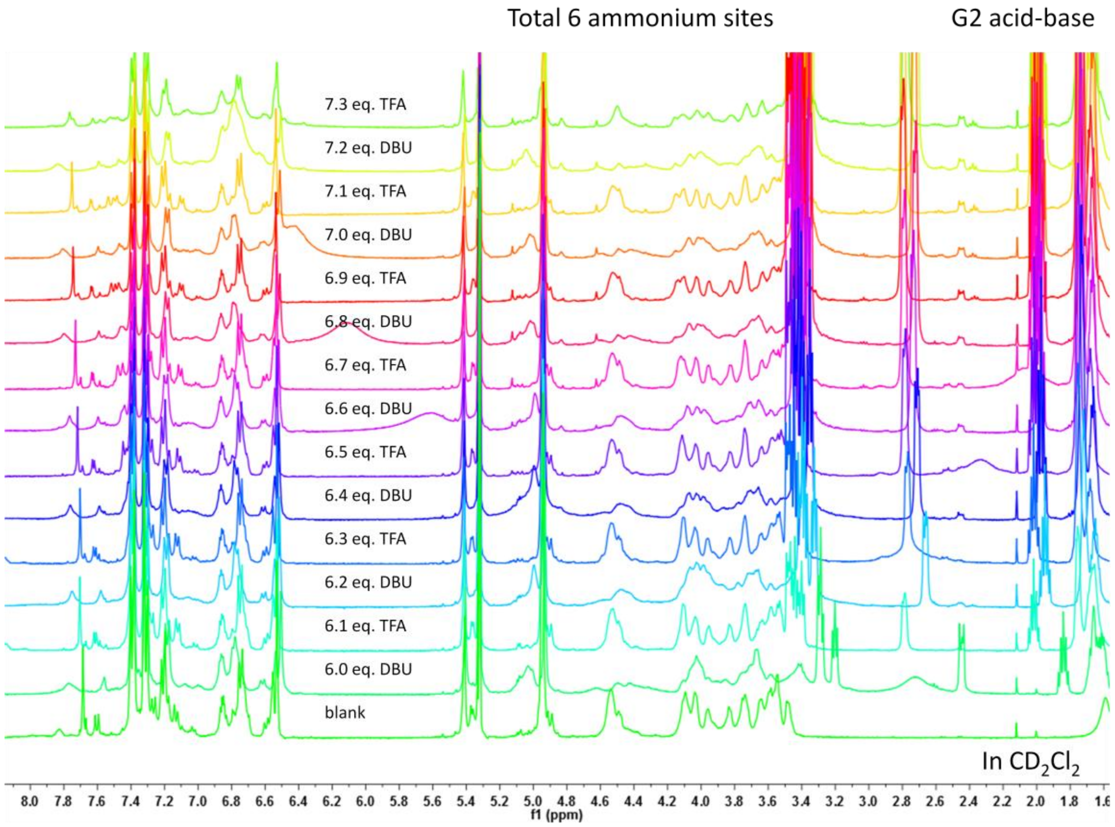

Supplementary Figure 60: Stacked ^{1}H NMR spectra (400 MHz, CD_2Cl_2) G2 [7]rotaxane dendrimer after alternatively addition of DBU and TFA with 7 cycles (Concentration: 1 mM).

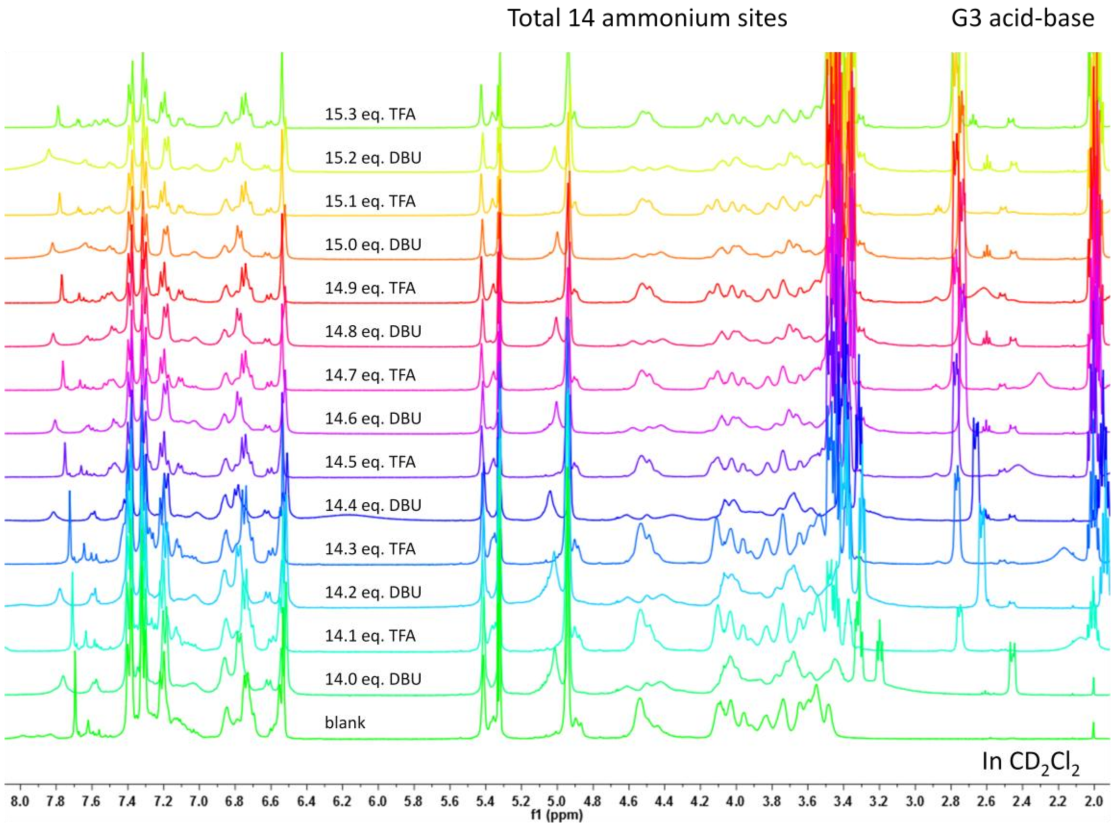

Supplementary Figure 61: Stacked ^{1}H NMR spectra (400 MHz, CD_2Cl_2) G3 [15]rotaxane dendrimer after alternatively addition of DBU and TFA with 7 cycles (Concentration: 1 mM).

Influence of Acid (TFA) in the Switching Process

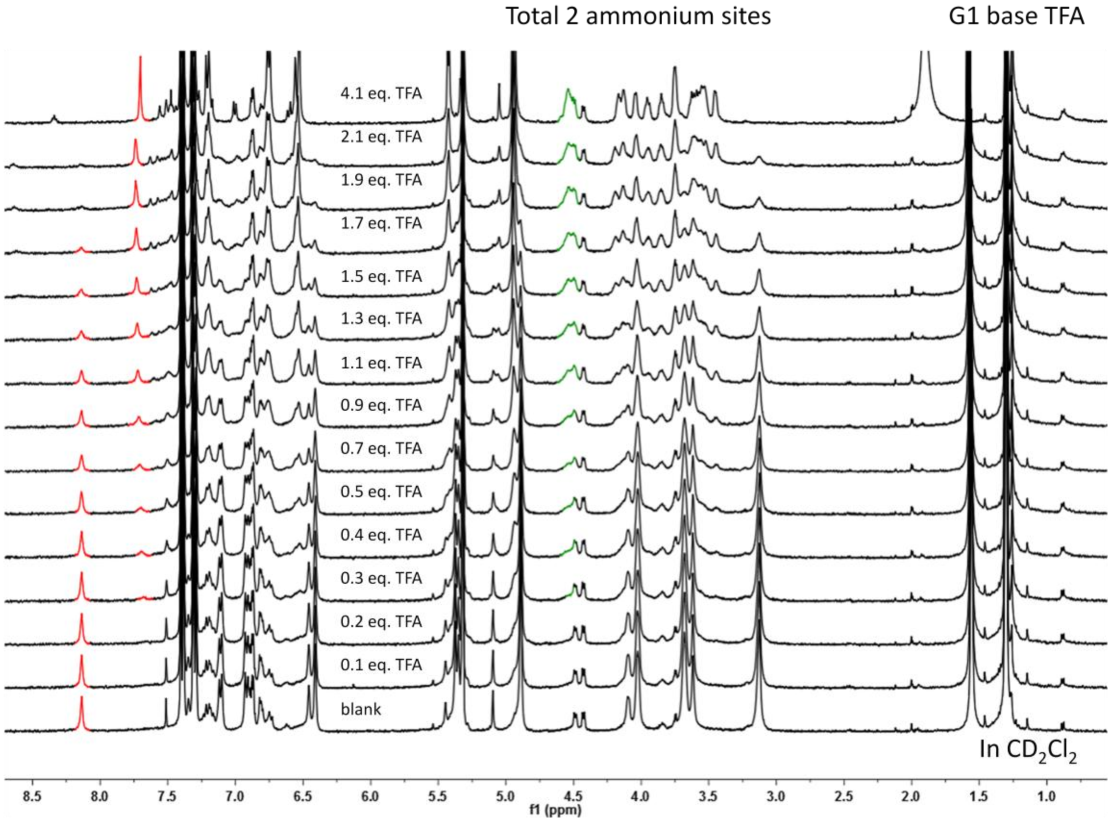

Supplementary Figure 62: Stacked ^{1}H NMR spectra (400 MHz, CD_2Cl_2) *Neutral* G1 [3]rotaxane dendrimer after cumulative addition of TFA (Concentration: 1 mM). Red color represents the triazole, and green color represents the protons adjacent to **DBA**.

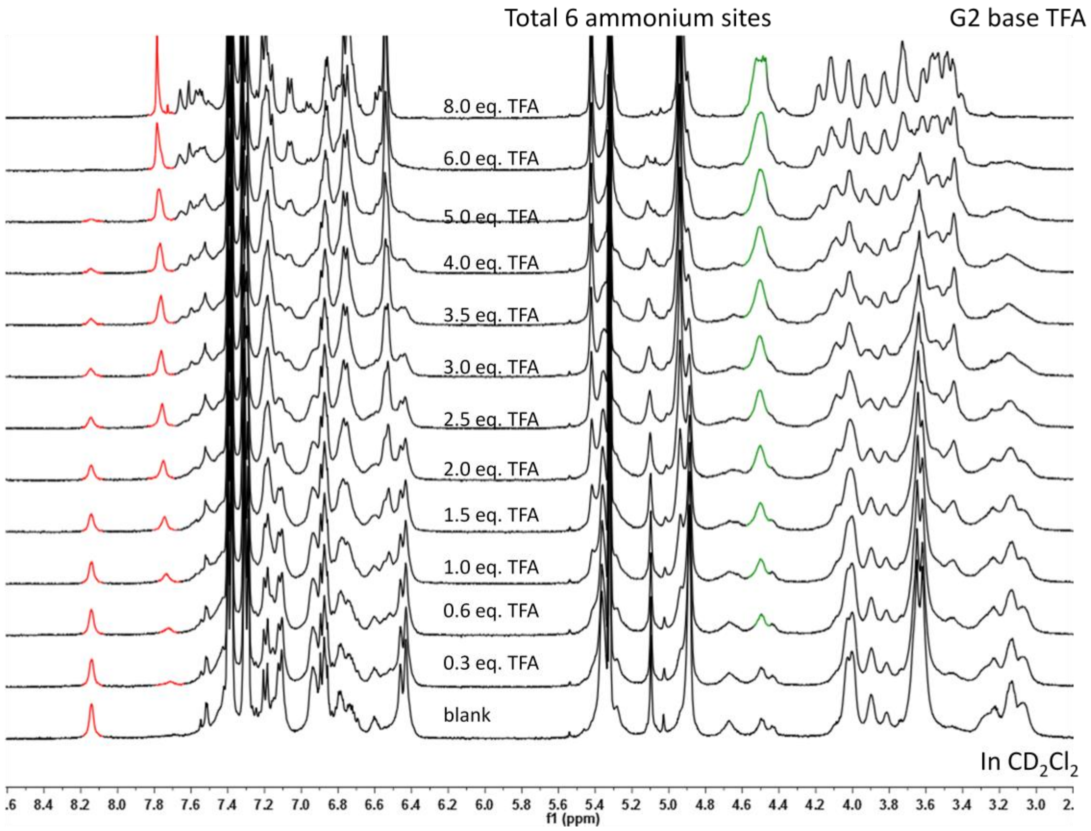

Supplementary Figure 63: Stacked ^{1}H NMR spectra (400 MHz, CD_2Cl_2) Neutral G2 [7]rotaxane dendrimer after cumulative addition of TFA (Concentration: 1 mM). Red color represents the triazole, and green color represents the protons adjacent to DBA.

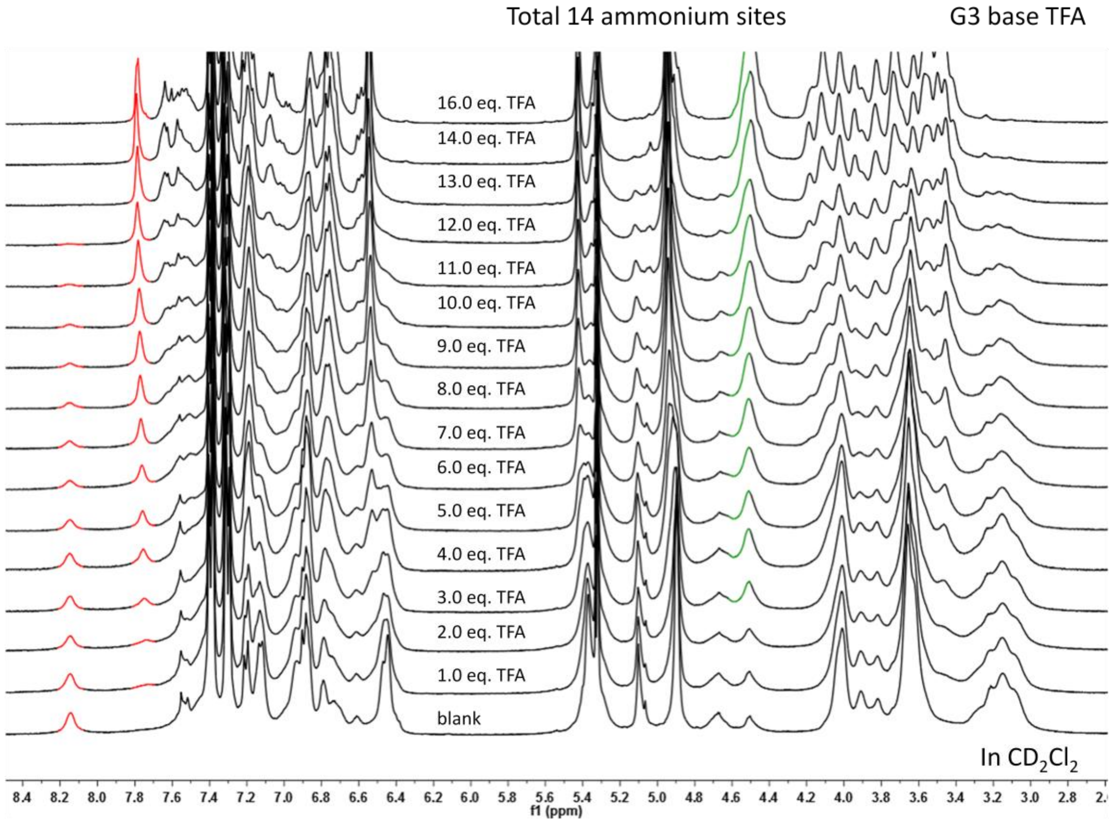

Supplementary Figure 64: Stacked ^{1}H NMR spectra (400 MHz, CD_2Cl_2) Neutral G3 [15]rotaxane dendrimer after cumulative addition of TFA (Concentration: 1 mM). Red color represents the triazole, and green color represents the protons adjacent to DBA.

Influence of Base (DBU) in the Switching Process

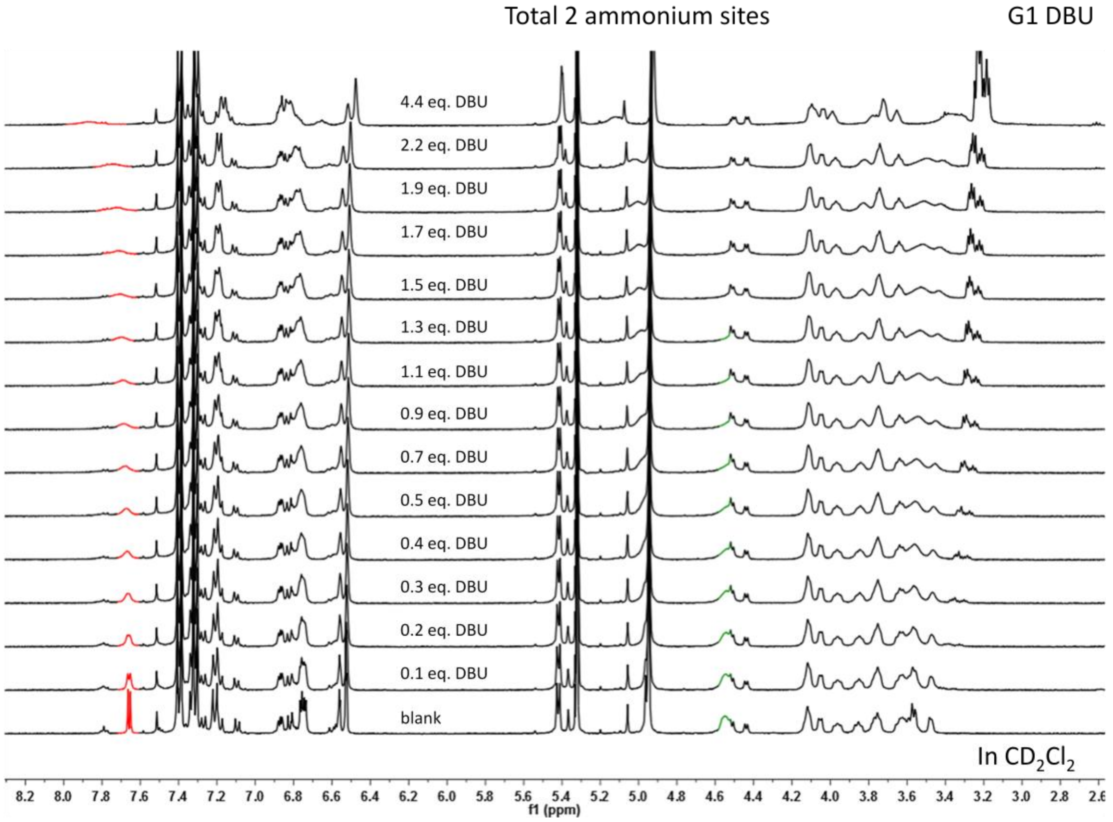

Supplementary Figure 65: Stacked ^{1}H NMR spectra (400 MHz, CD_2Cl_2) G1 [3]rotaxane dendrimer after cumulative addition of DBU (Concentration: 1 mM). Red color represents the triazole, and green color represents the protons adjacent to DBA.

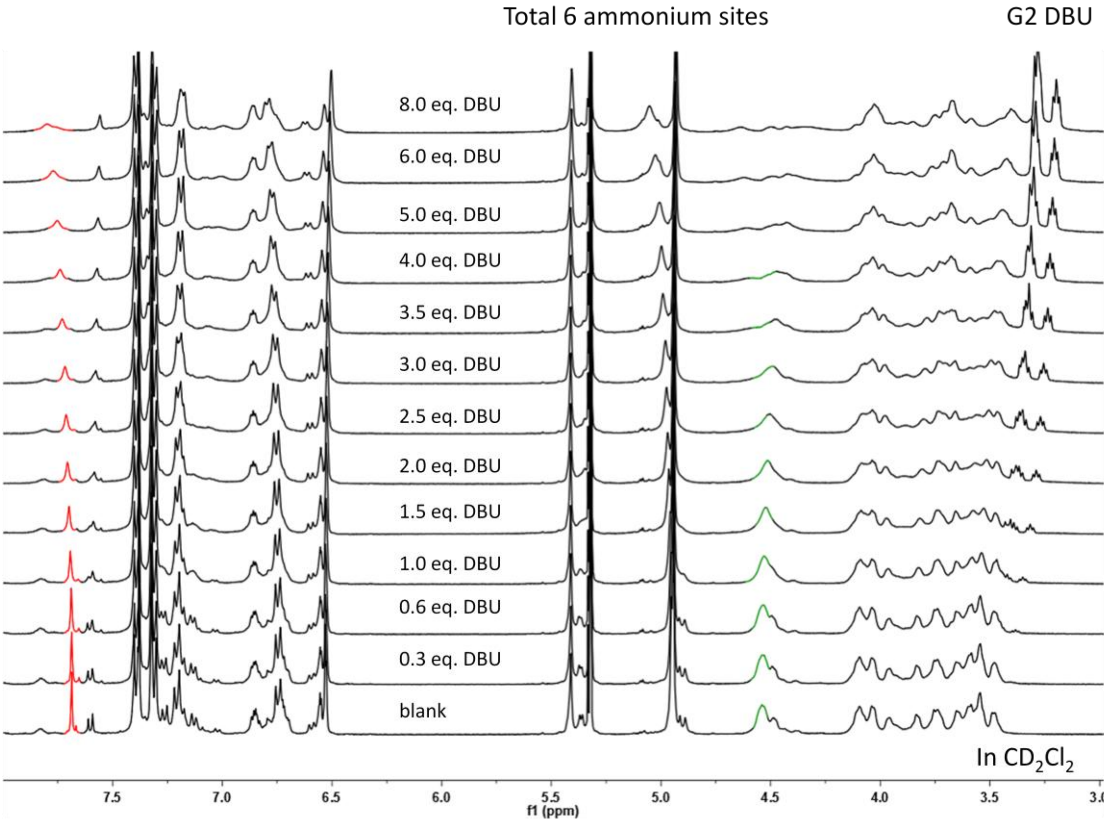

Supplementary Figure 66: Stacked ^{1}H NMR spectra (400 MHz, CD_2Cl_2) G2 [7]rotaxane dendrimer after cumulative addition of DBU (Concentration: 1 mM). Red color represents the triazole, and green color represents the protons adjacent to DBA.

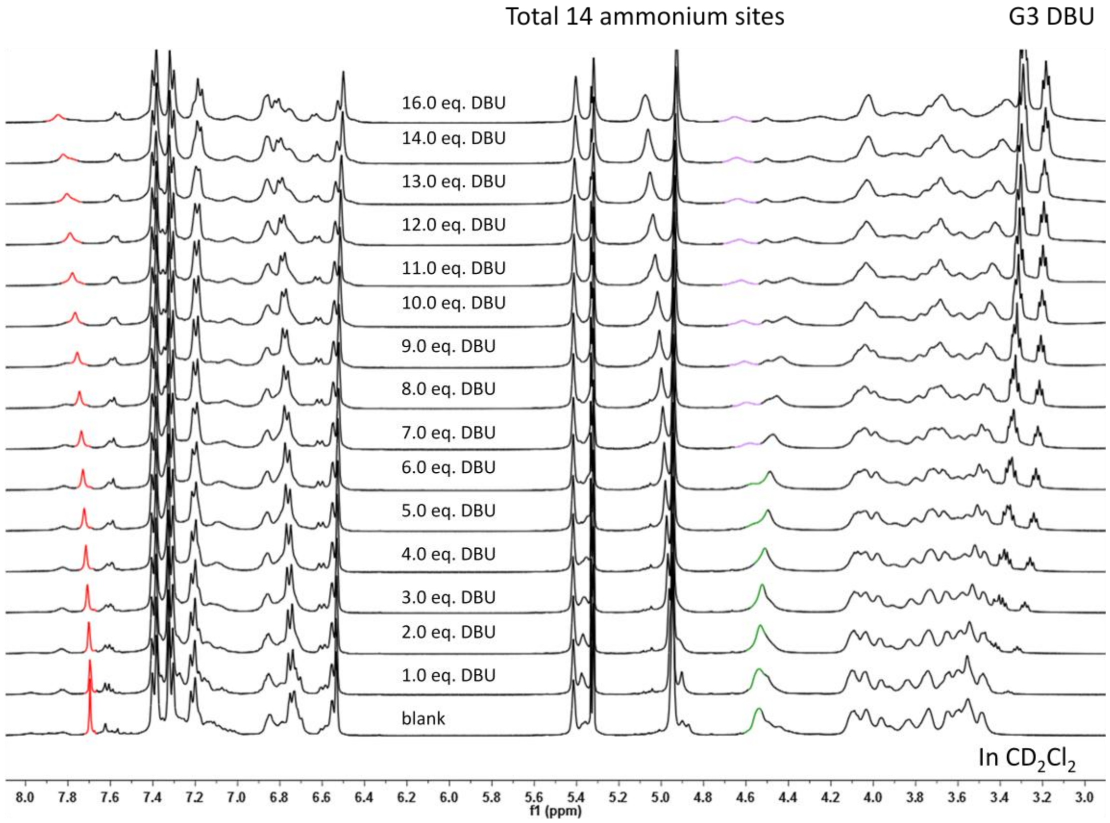

Supplementary Figure 67: Stacked ^{1}H NMR spectra (400 MHz, CD_2Cl_2) G3 [15]rotaxane dendrimer after cumulative addition of DBU (Concentration: 1 mM). Red color represents the triazole, green color represents the protons adjacent to DBA and purple color represents the amine.

Stepwise cumulative addition of acid/base was studied in the switching processes. When TFA was added gradually to all neutral G1–G3 T3B-RDs, ratiometric peaks of triazole (free and bound) were found (Supplementary Figures 62–64). This ratiometric peak corresponding to an incomplete protonation of all DBA sites, part of all DB24C8 molecules were shifted back to DBA, and some remained at the triazoles. Once excess TFA was added, all macrocycles shuttled back to DBA. When DBU was added stepwise to the G1–G3 T3B-RDs, triazole peaks broadened gradually and shifted downfield, while the DBA protons shifted upfield gradually. This phenomenon was similar to the addition of TFA to neutral G1–G3 rotaxane dendrimers, suggesting the shuttling of macrocycles in the rotaxane dendrimer was depending on the amount of acid/base added into the system.

Titration Experiment with Guest Molecules

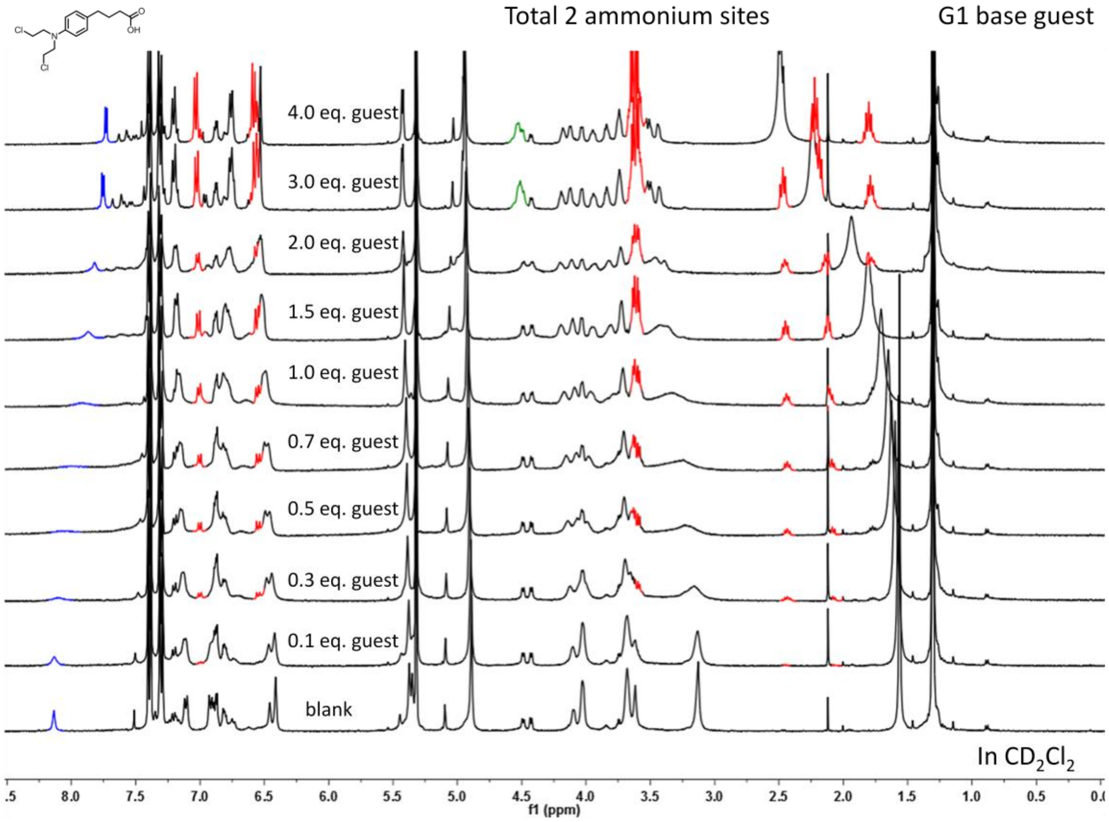

Supplementary Figure 68: Stacked ^{1}H NMR spectra (400 MHz, CD_2Cl_2) Neutral G1 [3]rotaxane dendrimer after cumulative addition of chlorambucil (Concentration: 1 mM). Red color represents the guest molecules, green color represents the protons adjacent to DBA, blue color represents the triazole protons.

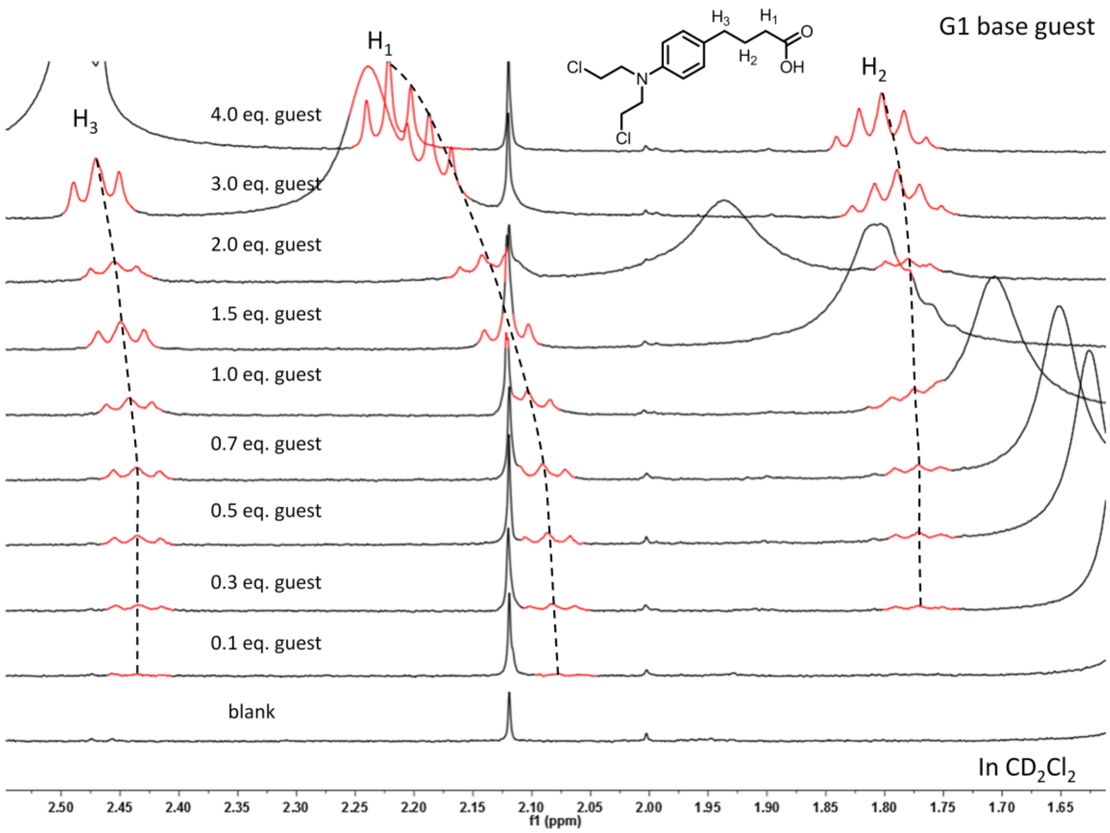

Supplementary Figure 69: Partial stacked ^{1}H NMR spectra (400 MHz, CD_2Cl_2) Neutral G1 [3]rotaxane dendrimer after cumulative addition of chlorambucil (Concentration: 1 mM). Red color represents the guest molecules.

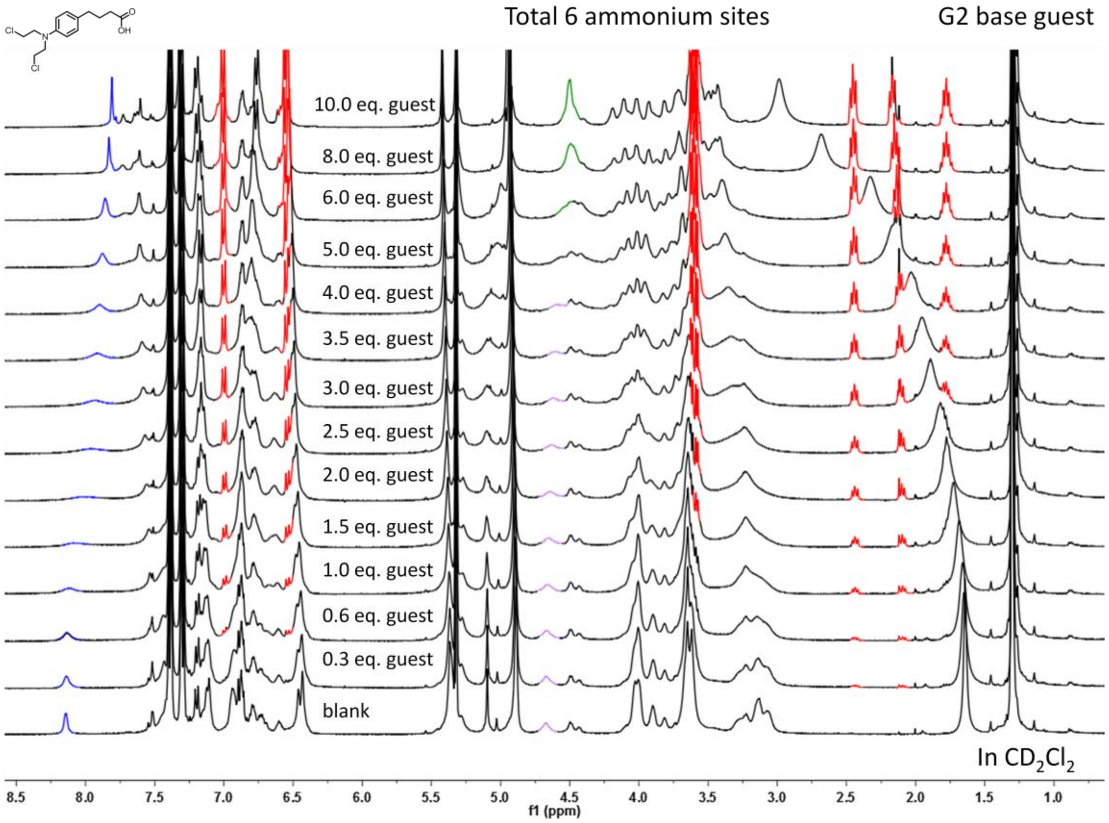

Supplementary Figure 70: Stacked ^{1}H NMR spectra (400 MHz, CD_2Cl_2) Neutral G2 [7]rotaxane dendrimer after cumulative addition of chlorambucil (Concentration: 1 mM). Red color represents the guest molecules, green color represents the protons adjacent to DBA, blue color represents the triazole protons and purple color represents the amine proton.

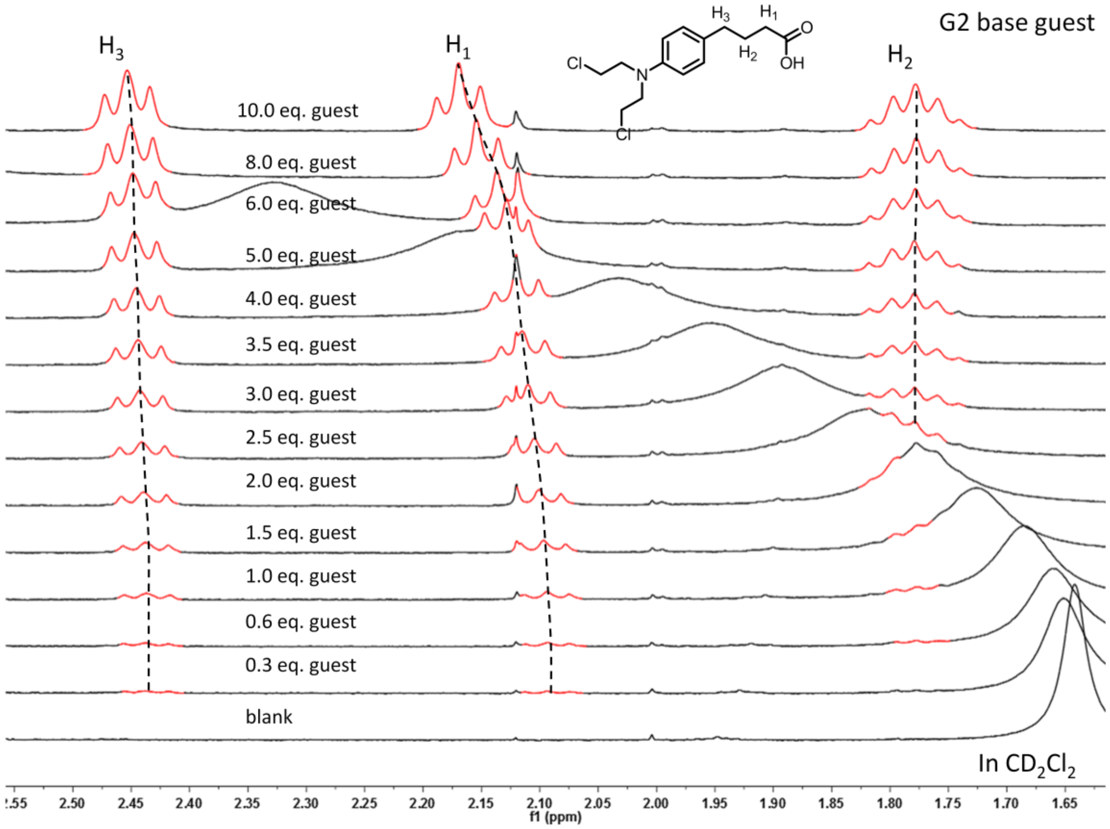

Supplementary Figure 71: Partial stacked ^{1}H NMR spectra (400 MHz, CD_2Cl_2) Neutral G2 [7]rotaxane dendrimer after cumulative addition of chlorambucil (Concentration: 1 mM). Red color represents the guest molecules.

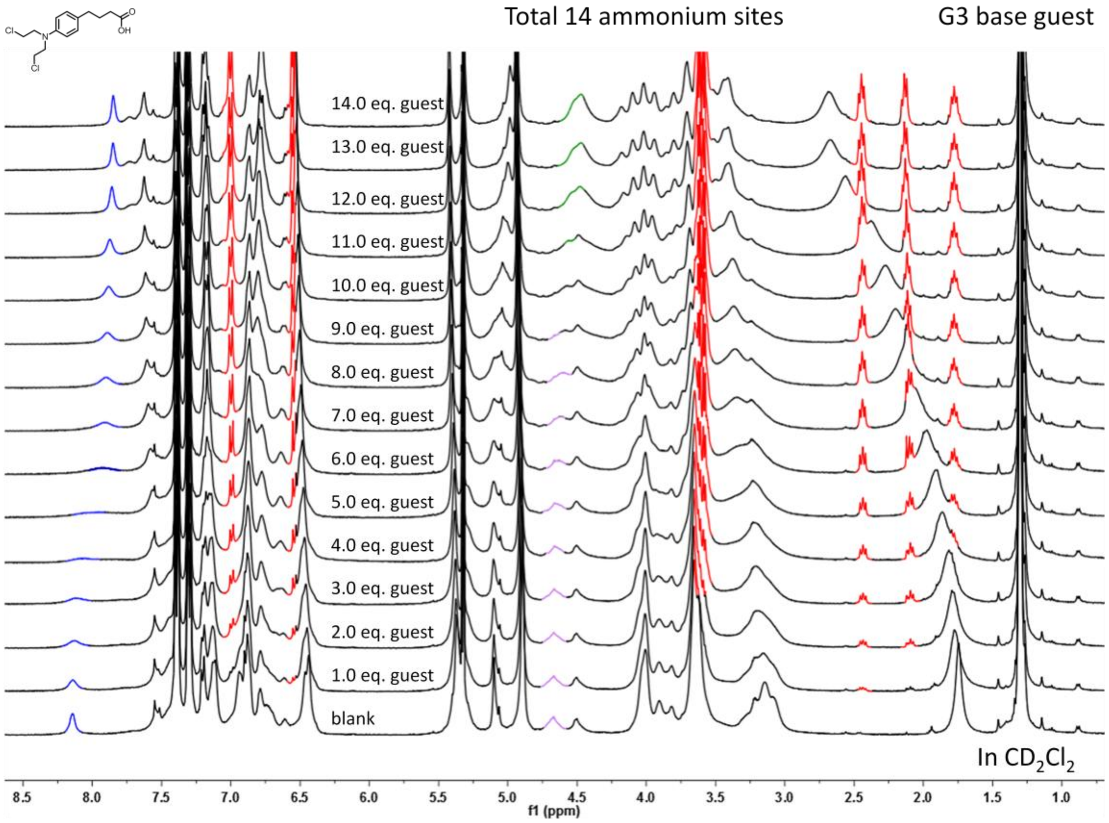

Supplementary Figure 72: Stacked ^{1}H NMR spectra (400 MHz, CD_2Cl_2) Neutral G3 [15]rotaxane dendrimer after cumulative addition of chlorambucil (Concentration: 1 mM). Red color represents the guest molecules, green color represents the protons adjacent to DBA, blue color represents the triazole protons and purple color represents the amine proton.

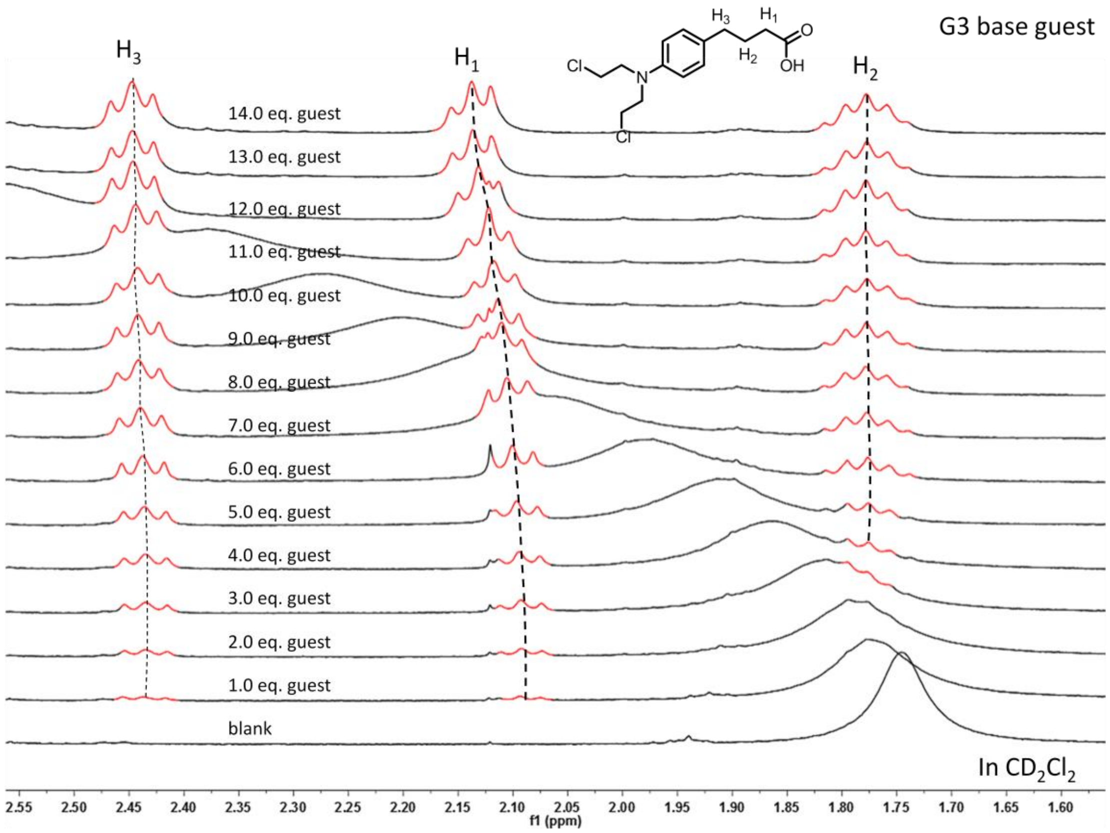

Supplementary Figure 73: Partial stacked ^{1}H NMR spectra (400 MHz, CD_2Cl_2) Neutral G3 [15]rotaxane dendrimer after cumulative addition of chlorambucil (Concentration: 1 mM). Red color represents the guest molecules.

In G1 dendrimer's NMR titration (Supplementary Figure 64), when titrating up to 2.0 equivalents of guest molecules, the unique hydrogen-bonded DBA proton signals are still not restored (green color), meaning that the macrocycle is still located at the triazole, while the DBA interacts with the carboxylate. Once excess acid (guest) was added, the carboxylate reprotonated to carboxylic acid and the macrocycle moved from triazoles back to the original site. Therefore, G1 could bind with two guest molecules by observing from NMR titration. In the case of G2 dendrimer's NMR titration (Supplementary Figure 70), we could observe the unique DB24C8-DBA binding signals after the addition of 4.0 equivalents of guest molecules, therefore one G2 dendrimer was able to bind with 4 guest molecules. In G3 dendrimer's NMR titration (Supplementary Figure 72), similar peak shifts were used for the determination of guest molecules binding to the rotaxane dendrimers, and 8 guest molecules were bound. In conclusion, guest substrates can be bound to the amine sites at the outer layer of the dendrimers.

When excess guest (acid) was titrated to the rotaxane dendrimers solution, the excess

acid will protonate the carboxylate (bind) to carboxylic acid (free), and the macrocycle will shuttle back to the DBA sites, as proved by ^{1}H NMR, in comparison to the titration with TFA (Supplementary Figure 62–64).

The protons shifted of the guest molecule (chlorambucil) was due to the electrostatic interaction of the carboxylate with DBA.[9]

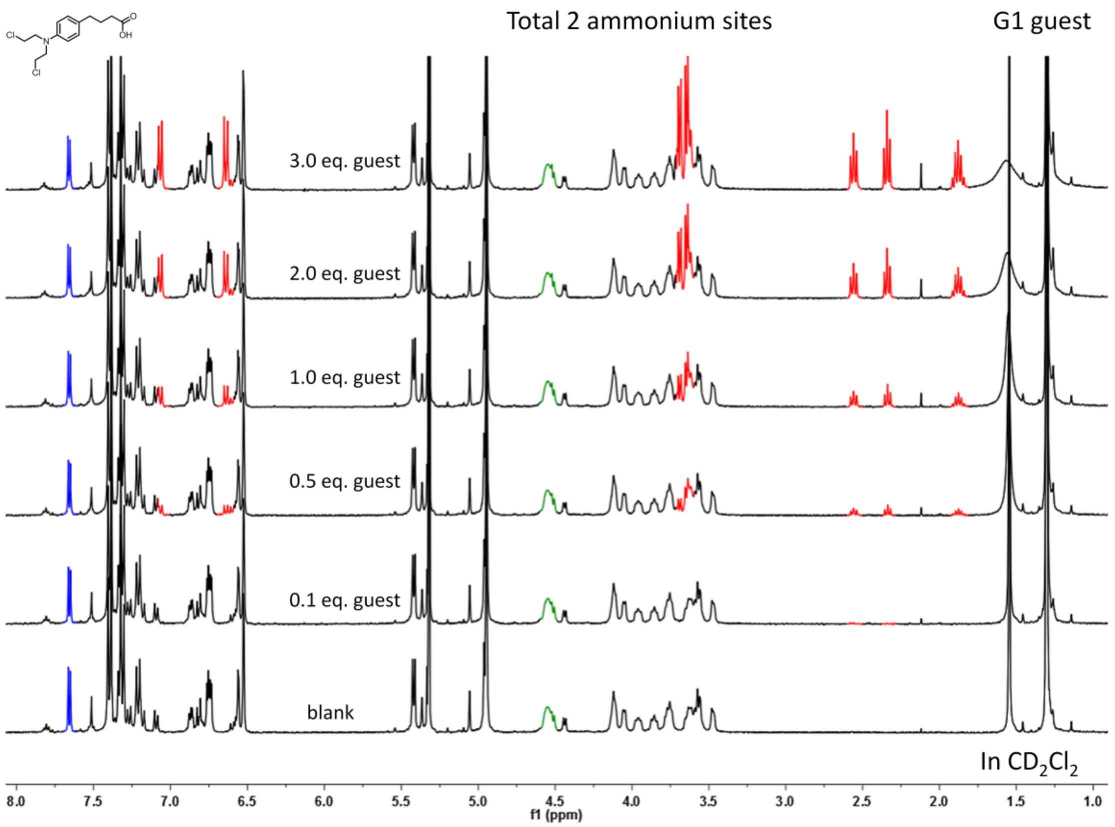

Supplementary Figure 74: Stacked ^{1}H NMR spectra (400 MHz, CD_2Cl_2) G1 [3]rotaxane dendrimer after cumulative addition of chlorambucil (Concentration: 1 mM). Red color represents the guest molecules, green color represents the protons adjacent to DBA, blue color represents the triazole protons.

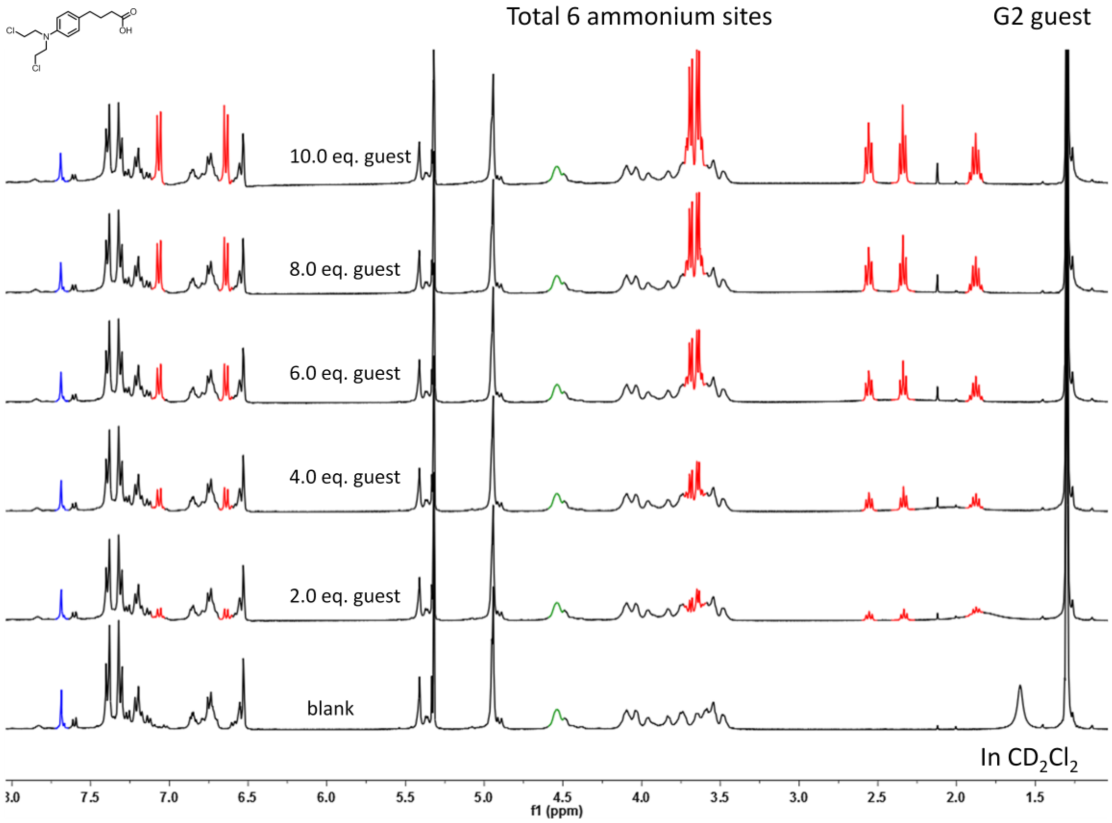

Supplementary Figure 75: Stacked ^{1}H NMR spectra (400 MHz, CD_2Cl_2) G2 [7]rotaxane dendrimer after cumulative addition of chlorambucil (Concentration: 1 mM). Red color represents the guest molecules, green color represents the protons adjacent to DBA, blue color represents the triazole protons.

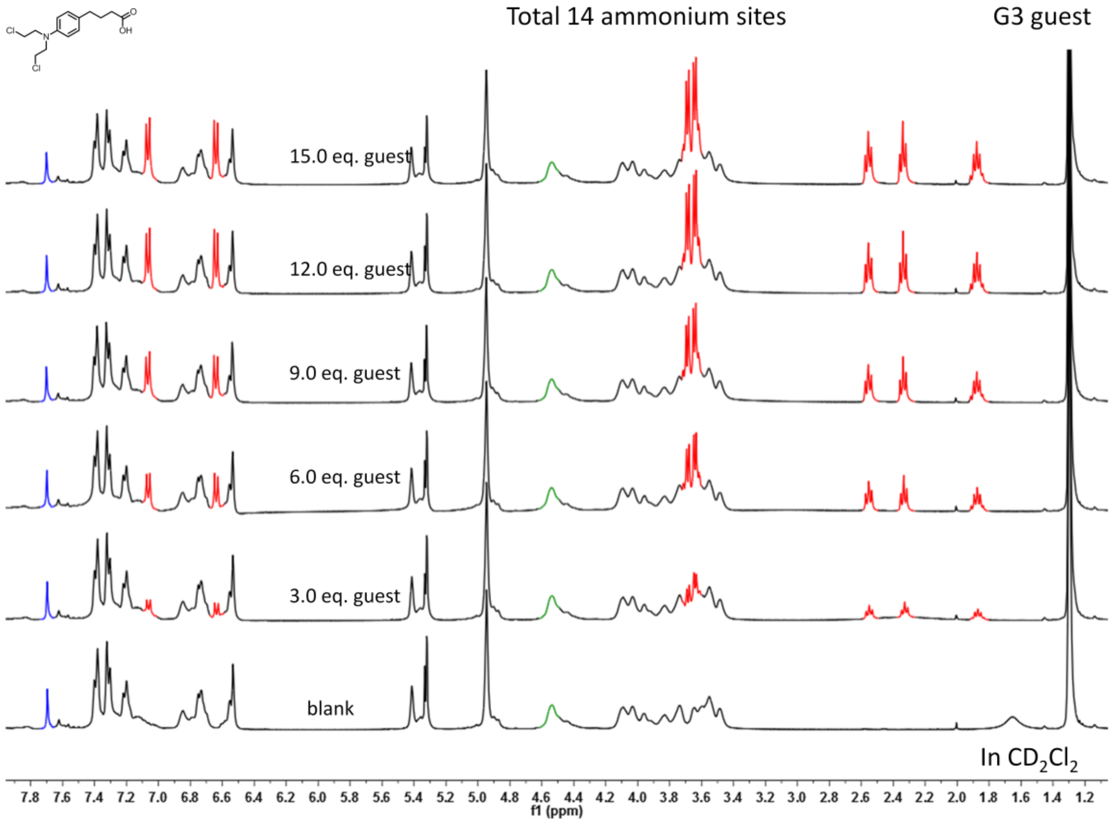

Supplementary Figure 76: Stacked ^{1}H NMR spectra (400 MHz, CD_2Cl_2) G3 [15]rotaxane dendrimer after cumulative addition of chlorambucil (Concentration: 1 mM). Red color represents the guest molecules, green color represents the protons adjacent to DBA, blue color represents the triazole protons.

Thread was used as a model for the study, and once guest (chlorambucil) was added, the proton adjacent to DBA shifted downfield slightly, due to the protonation and the ionic interaction with the guest molecules. On the other hand, when titrating the guest molecules to the ionic G1–G3 rotaxane dendrimers, no any chemical shift was observed in both dendrimer and guest molecules, indicating no any interaction between the two components.

Binding Constant (K_a) Determination

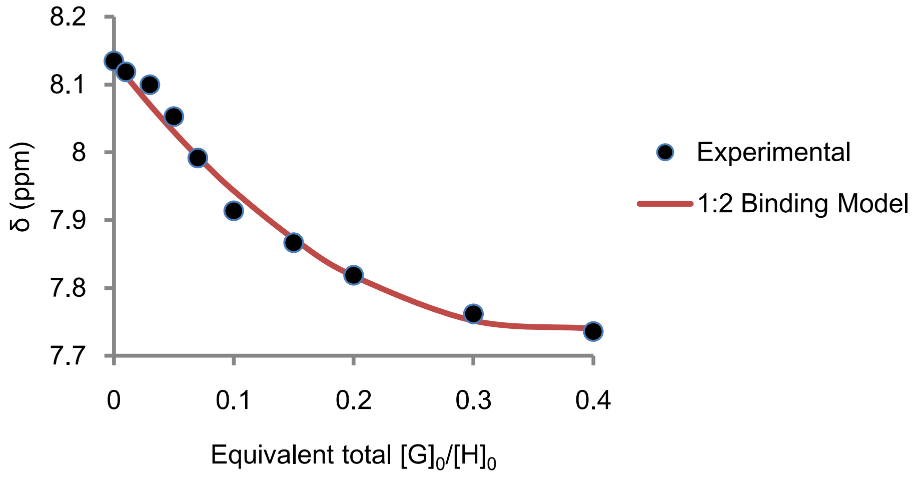

Supplementary Figure 77: The chemical shift of triazole in G1 rotaxane dendrimer with a 1:2 binding model.

Binding constant (K_a) was calculated by monitoring the chemical shift of triazole protons in G1 rotaxane dendrimer.

The experimental chemical shift was in agreement with the calculated 1:2 binding model, and the binding constant (K_a) was calculated to be 5.2 x 10^5 M^{-1} with online software BindFit v0.5 demonstrated by Throdarson *el al.* [11]

NMR Spectra of Selected Compounds

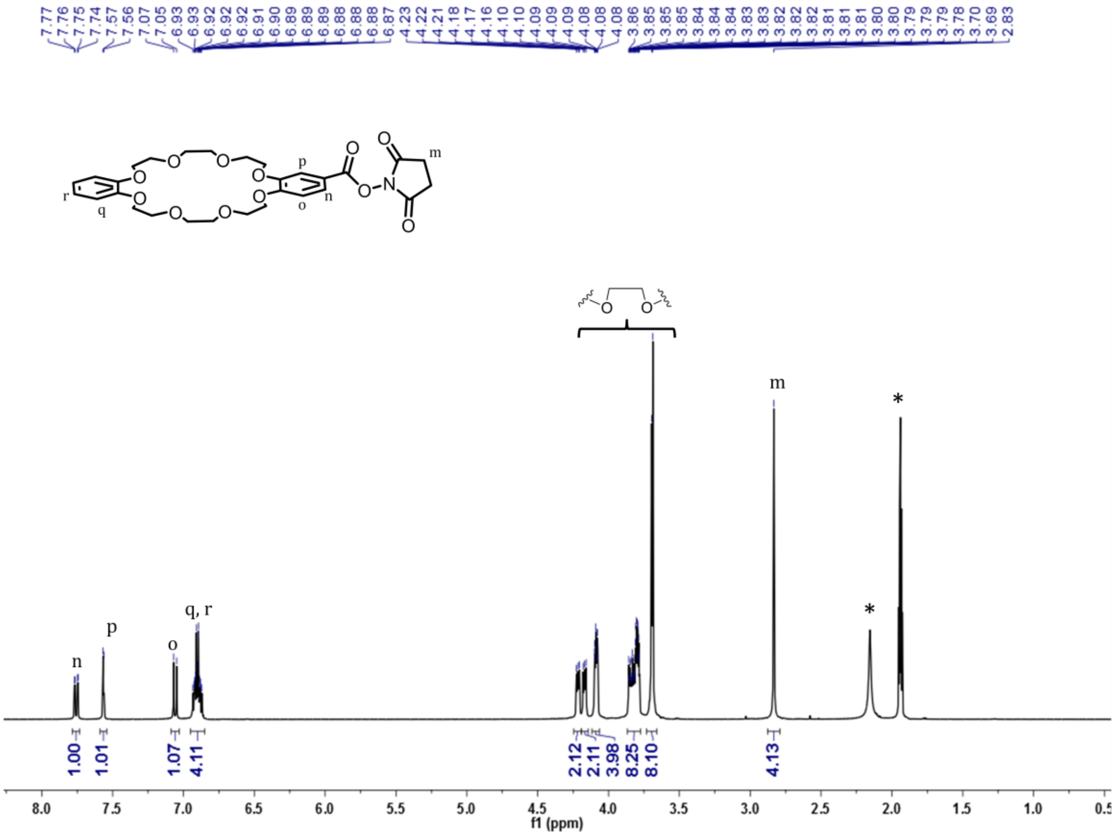

Supplementary Figure 78: ^{1}H NMR spectrum (400 MHz, CD_3CN) of DB24C8-OSu (Asterisk: solvent residual signal).

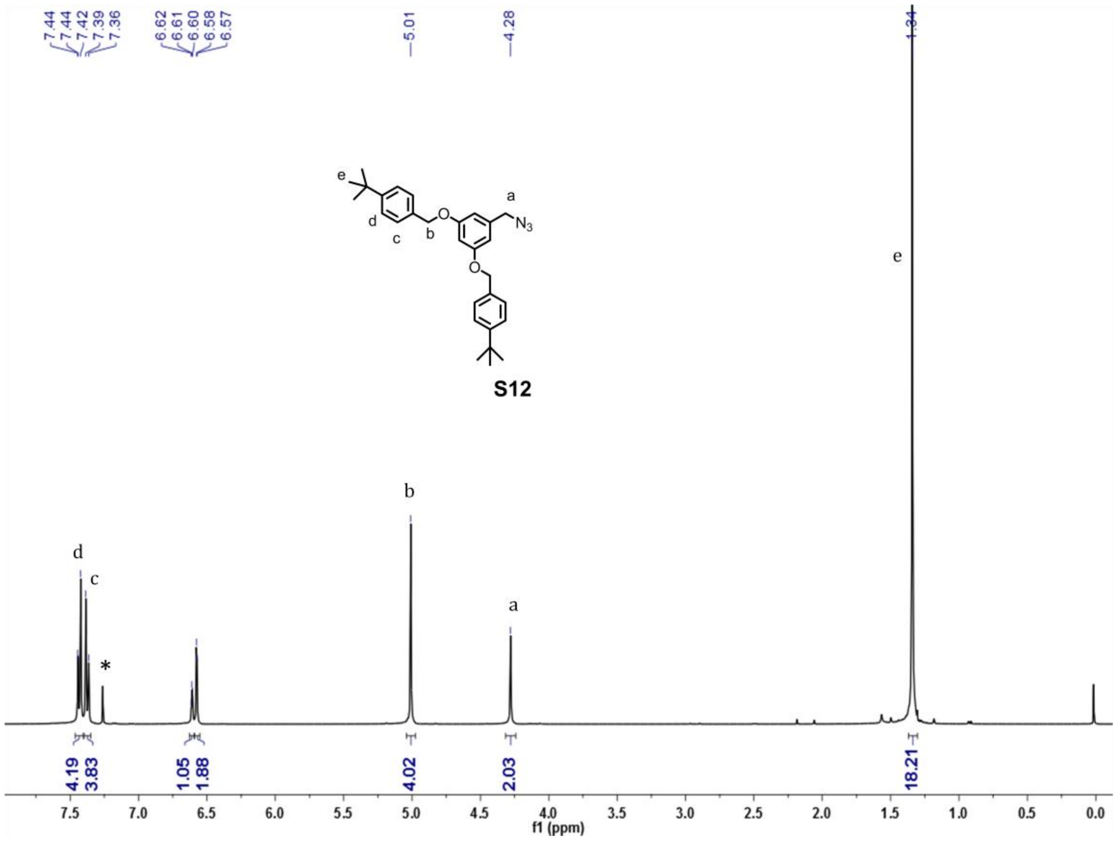

Supplementary Figure 79: ^{1}H NMR spectrum (400 MHz, $CDCl_3$) of **S12** (Asterisk: solvent residual signal).

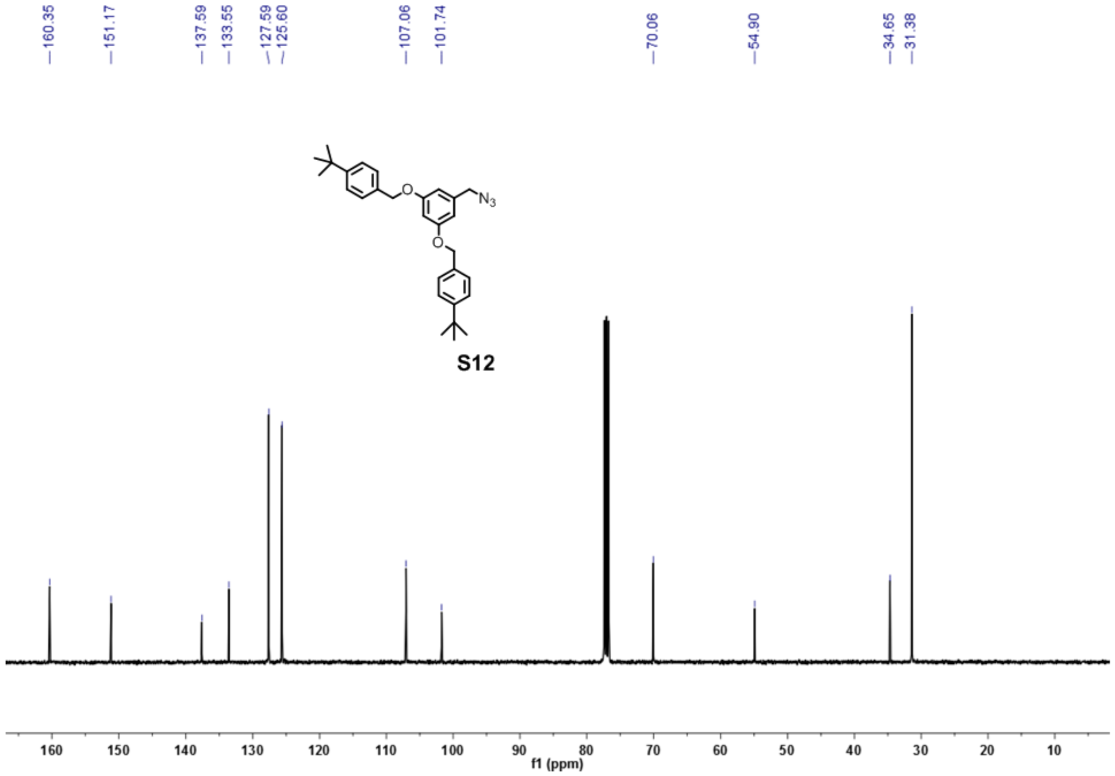

Supplementary Figure 80: ^{13}C NMR spectrum (101 MHz, $CDCl_3$) of **S12**.

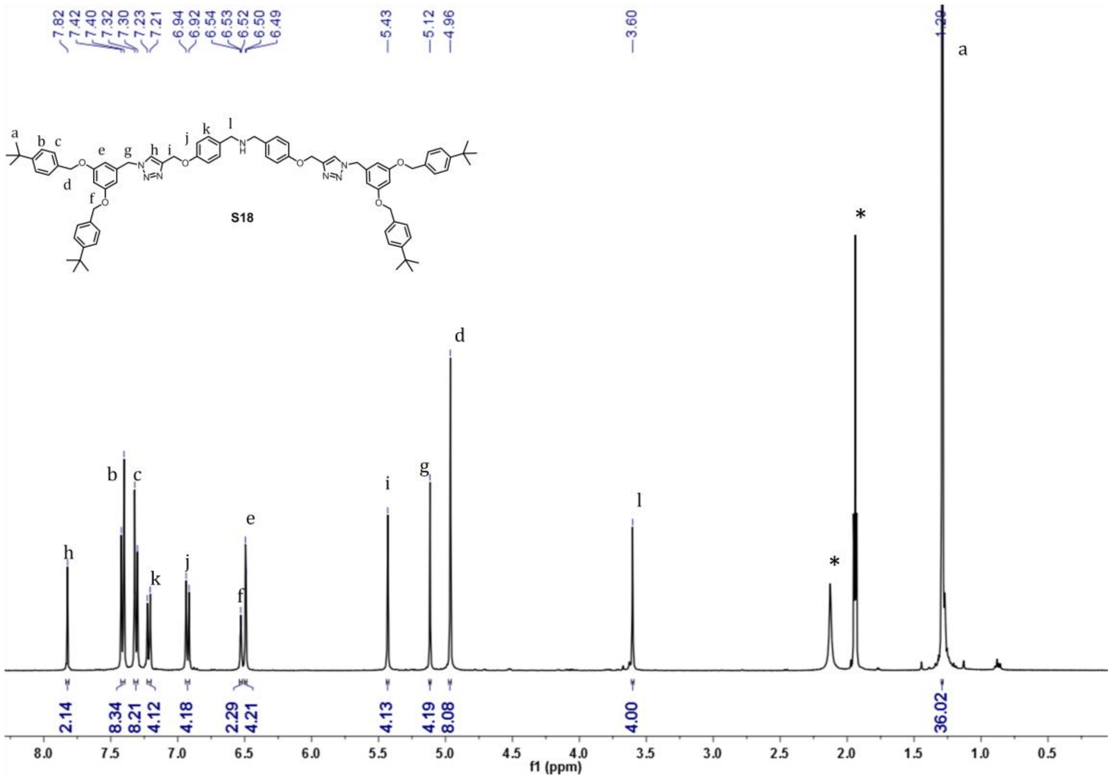

Supplementary Figure 81: ^{1}H NMR spectrum (400 MHz, CD_3CN) of **S18** (Asterisk: solvent residual signal).

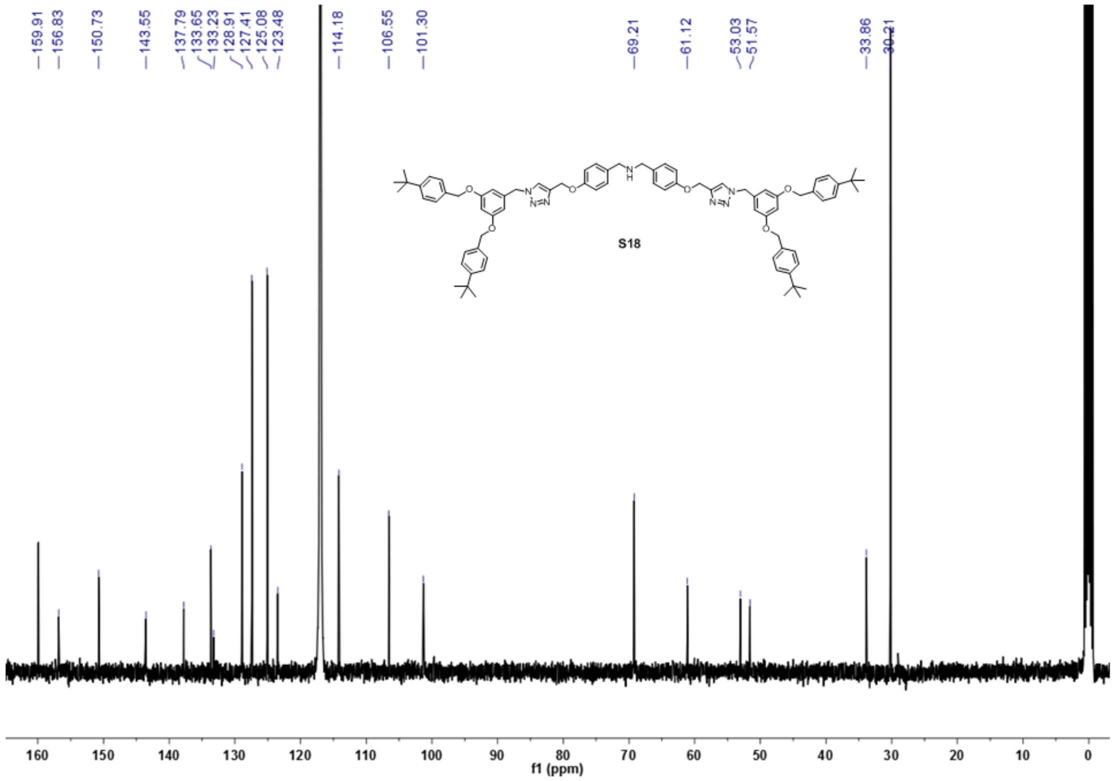

Supplementary Figure 82: ^{13}C NMR spectrum (101 MHz, $CDCl_3$) of **S18**.

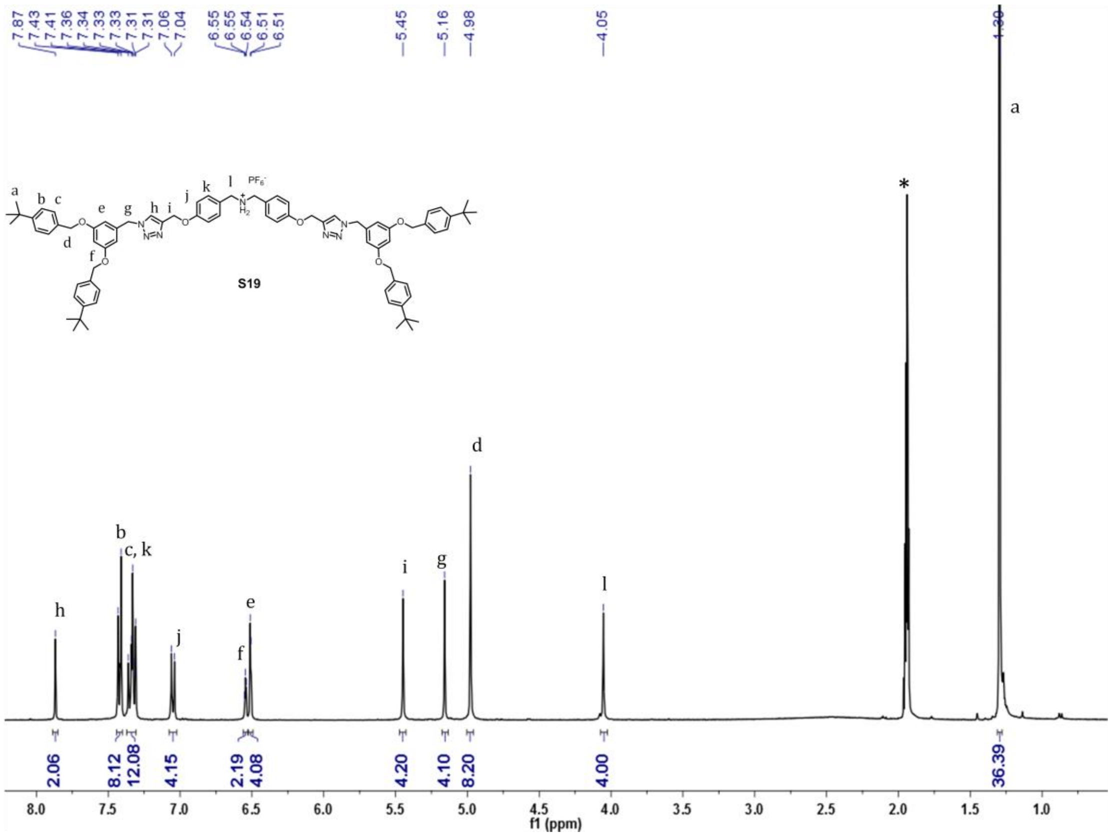

Supplementary Figure 83: ^{1}H NMR spectrum (400 MHz, CD_3CN) of **S19** (Asterisk:

solvent residual signal).

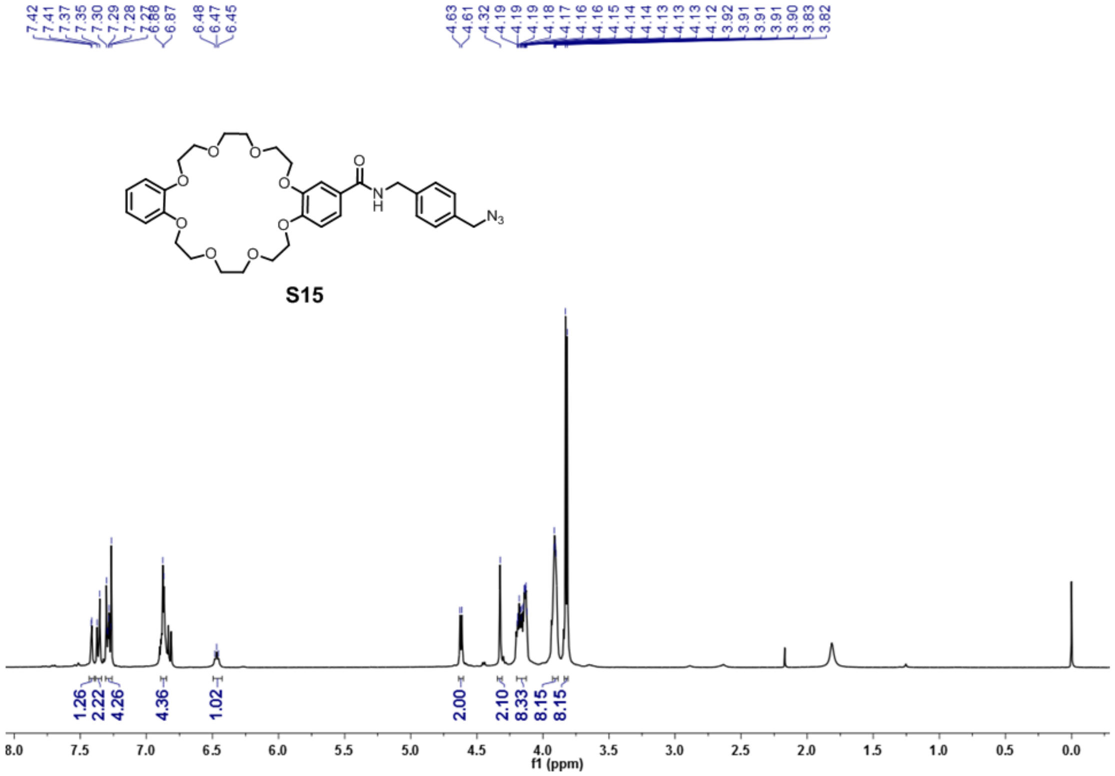

Supplementary Figure 84: ^{1}H NMR spectrum (400 MHz, $CDCl_3$) of **S15**.

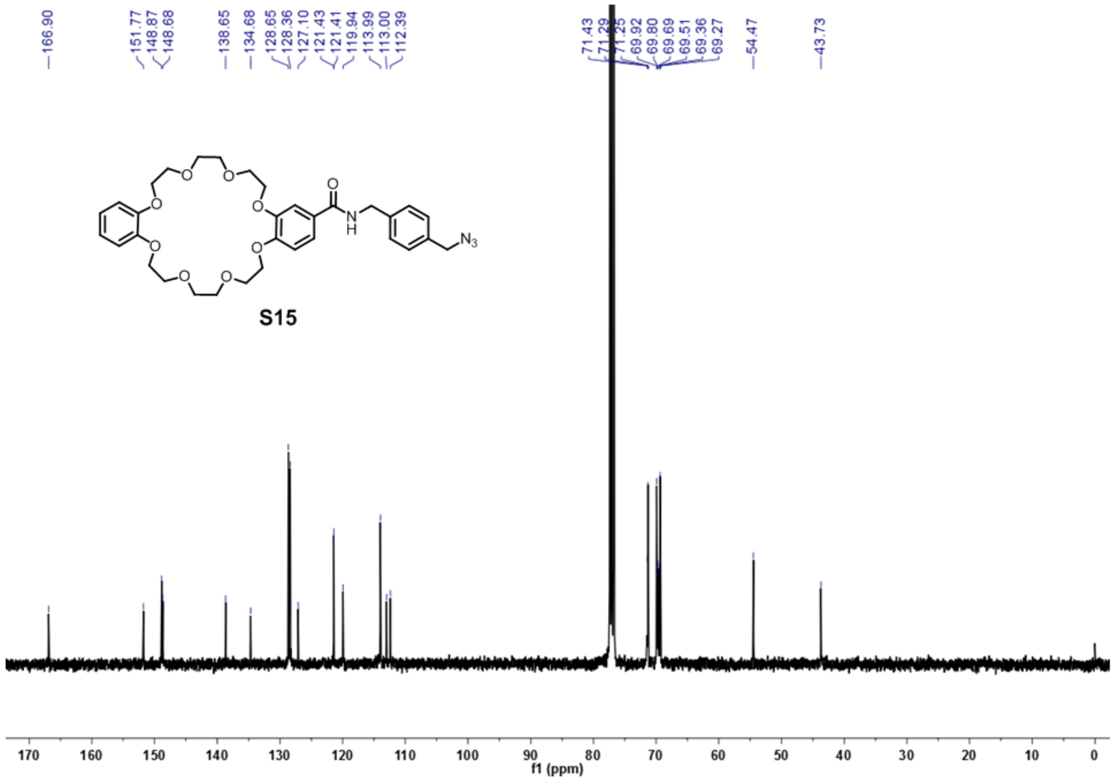

Supplementary Figure 85: ^{13}C NMR spectrum (101 MHz, $CDCl_3$) of **S15**.

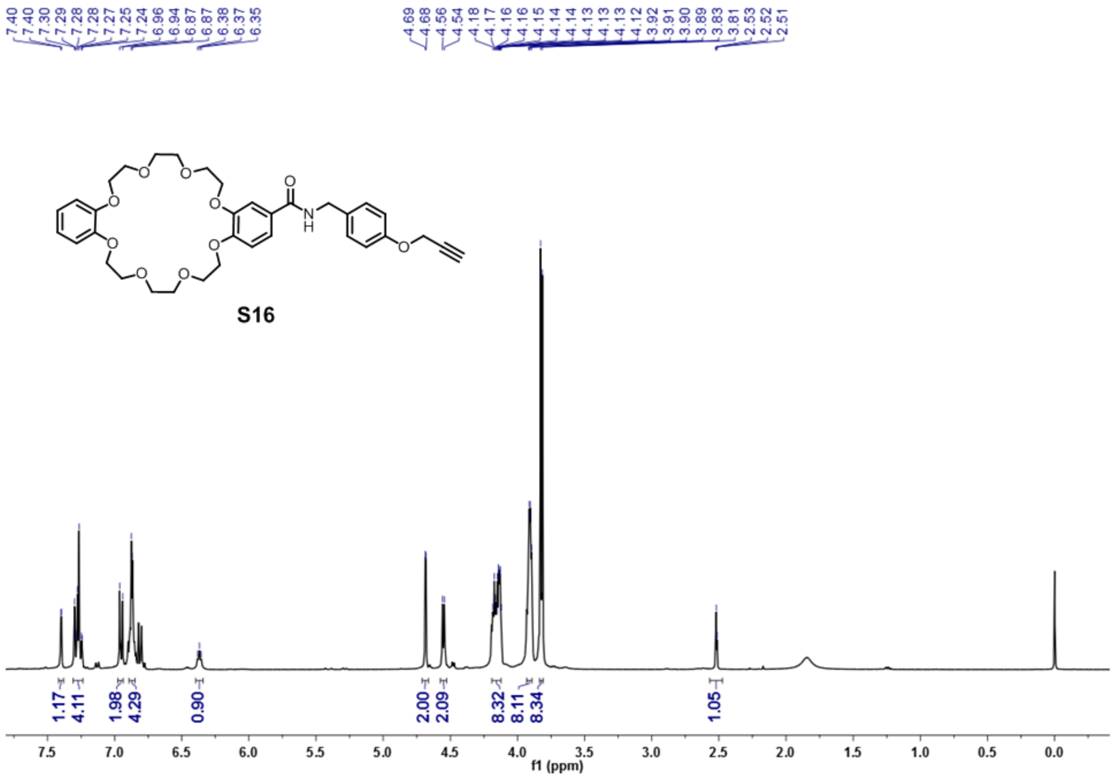

Supplementary Figure 86: ^{1}H NMR spectrum (400 MHz, $CDCl_3$) of **S16**.

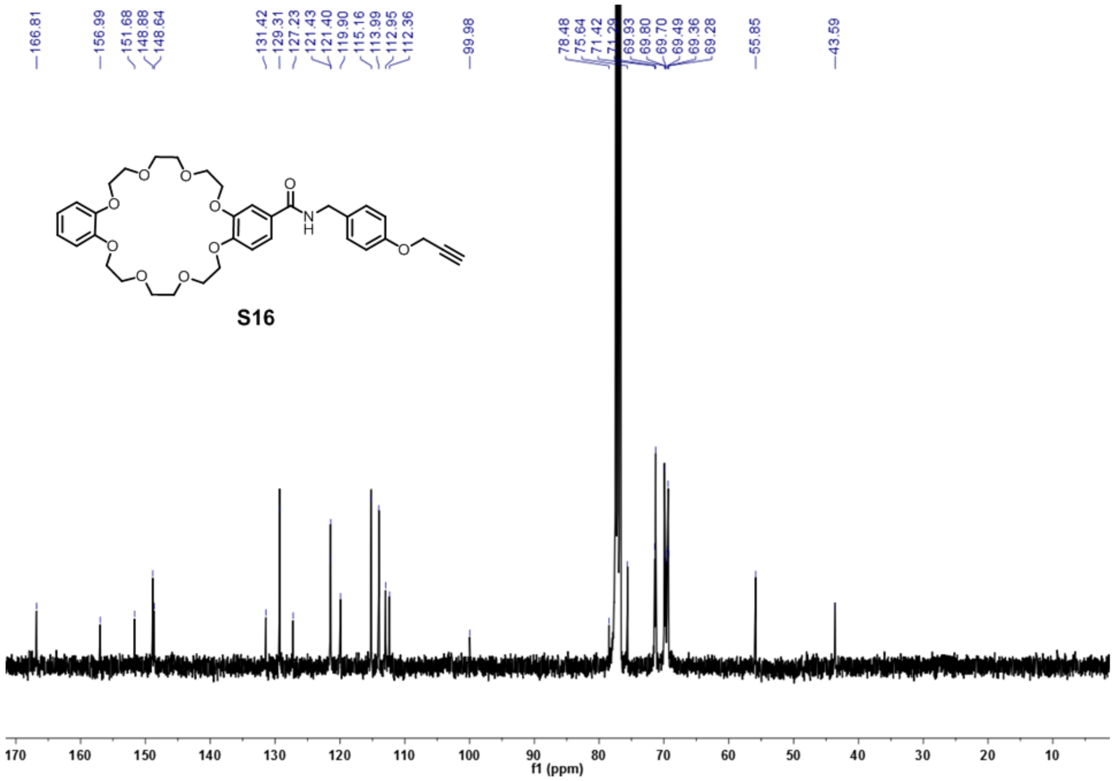

Supplementary Figure 87: ^{13}C NMR spectrum (101 MHz, $CDCl_3$) of **S16**.

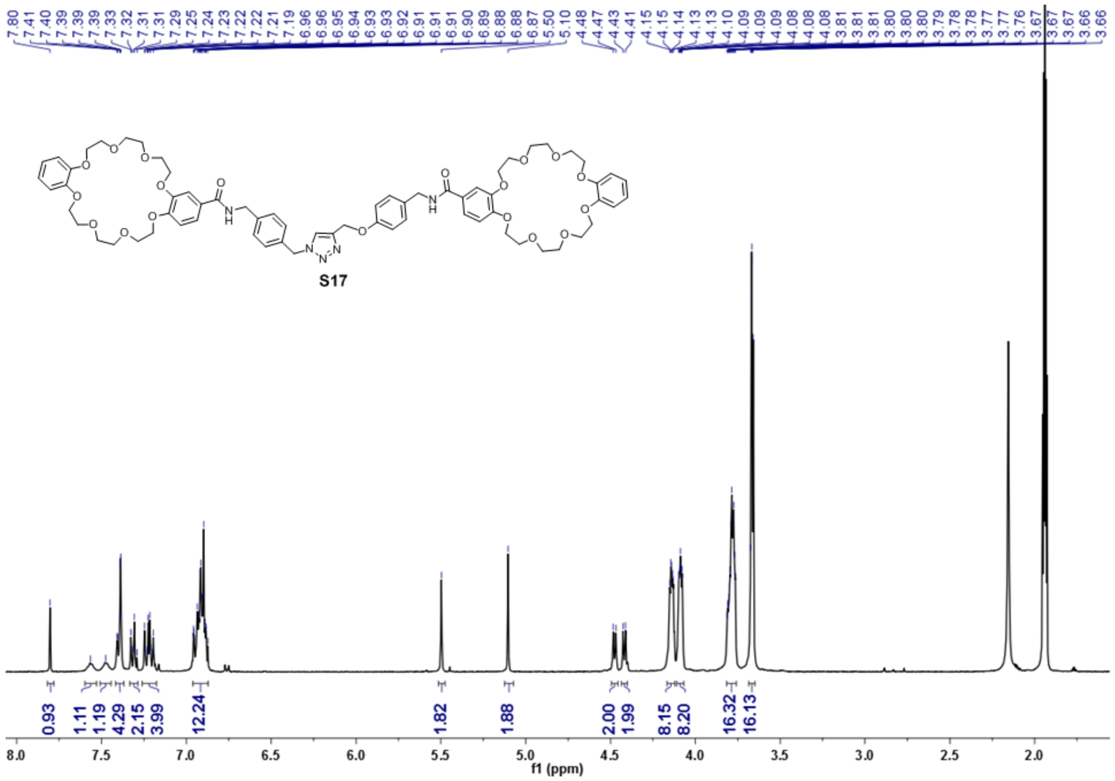

Supplementary Figure 88: ^{1}H NMR spectrum (400 MHz, CD_3CN) of **S17**.

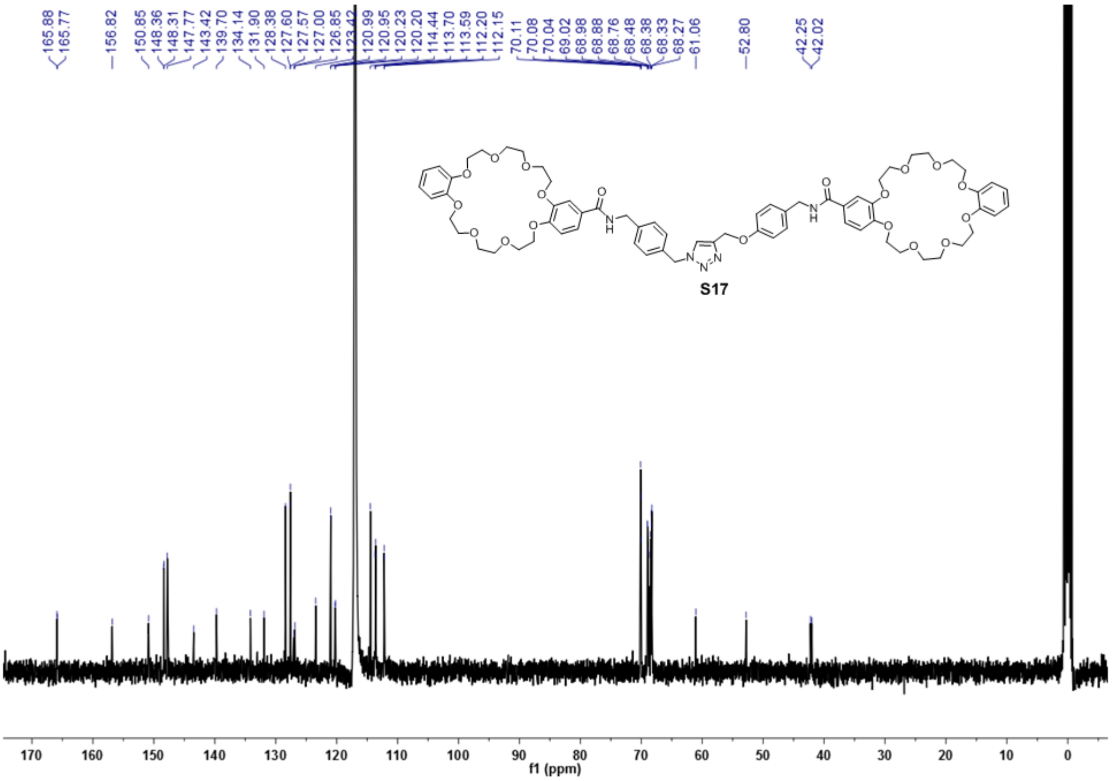

Supplementary Figure 89: ^{13}C NMR spectrum (101 MHz, CD_3CN) of **S17**.

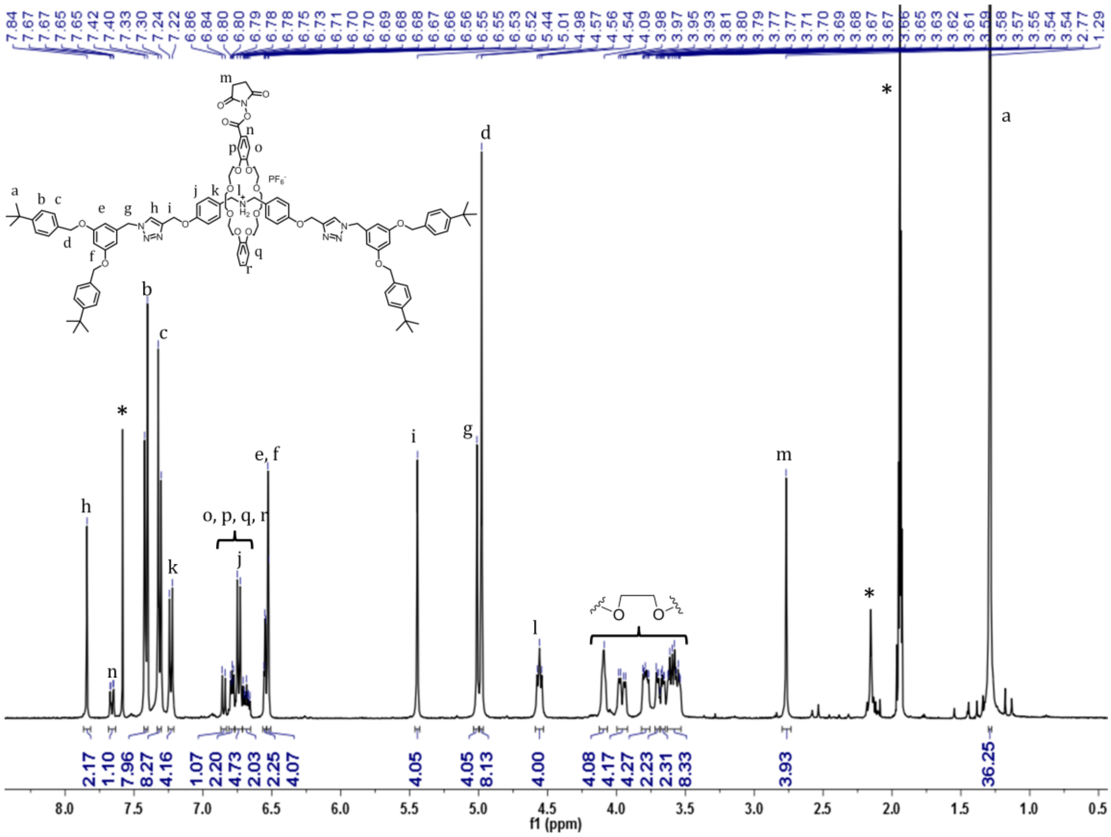

Supplementary Figure 90: ^{1}H NMR spectrum (400 MHz, CD_3CN) of G1

[2]Rotaxane Dendron-NHS (Asterisk: solvent residual signal).

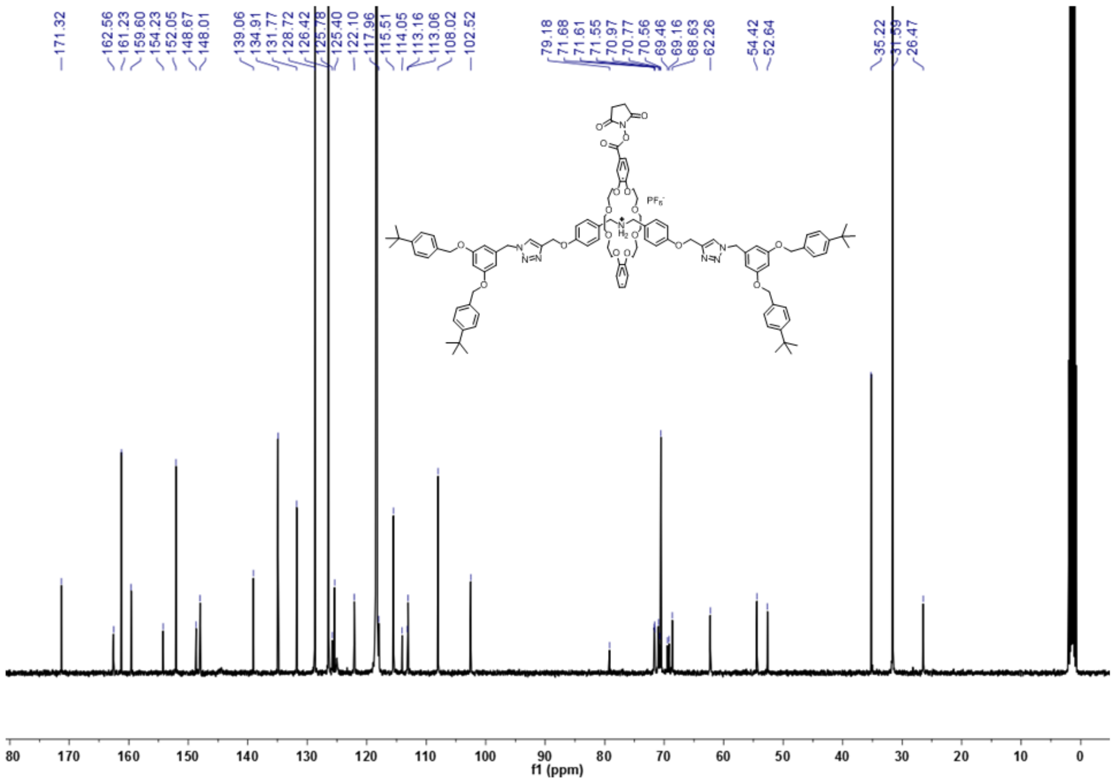

Supplementary Figure 91: ^{13}C NMR spectrum (400 MHz, CD_3CN) of G1 [2]Rotaxane Dendron-NHS.

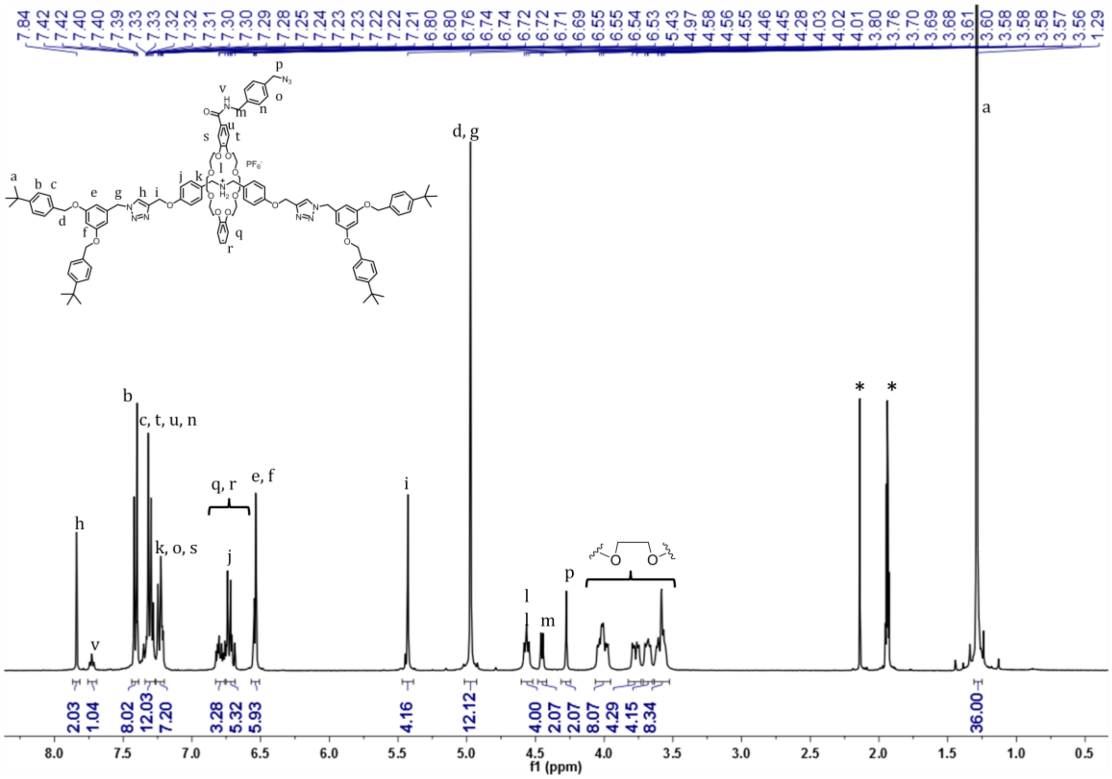

Supplementary Figure 92: ^{1}H NMR spectrum (400 MHz, CD_3CN) of G1 [2]Rotaxane Dendron-Azide (Asterisk: solvent residual signal).

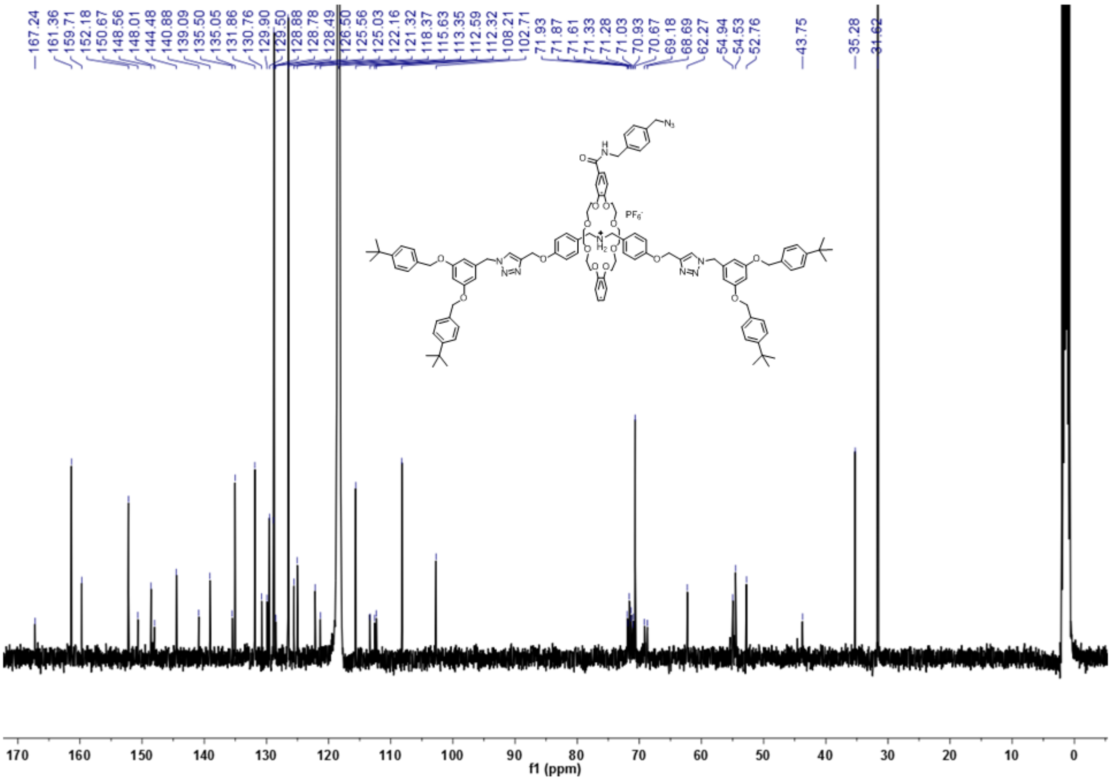

Supplementary Figure 93: ^{13}C NMR spectrum (101 MHz, CD_3CN) of G1

[2]Rotaxane Dendron-Azide.

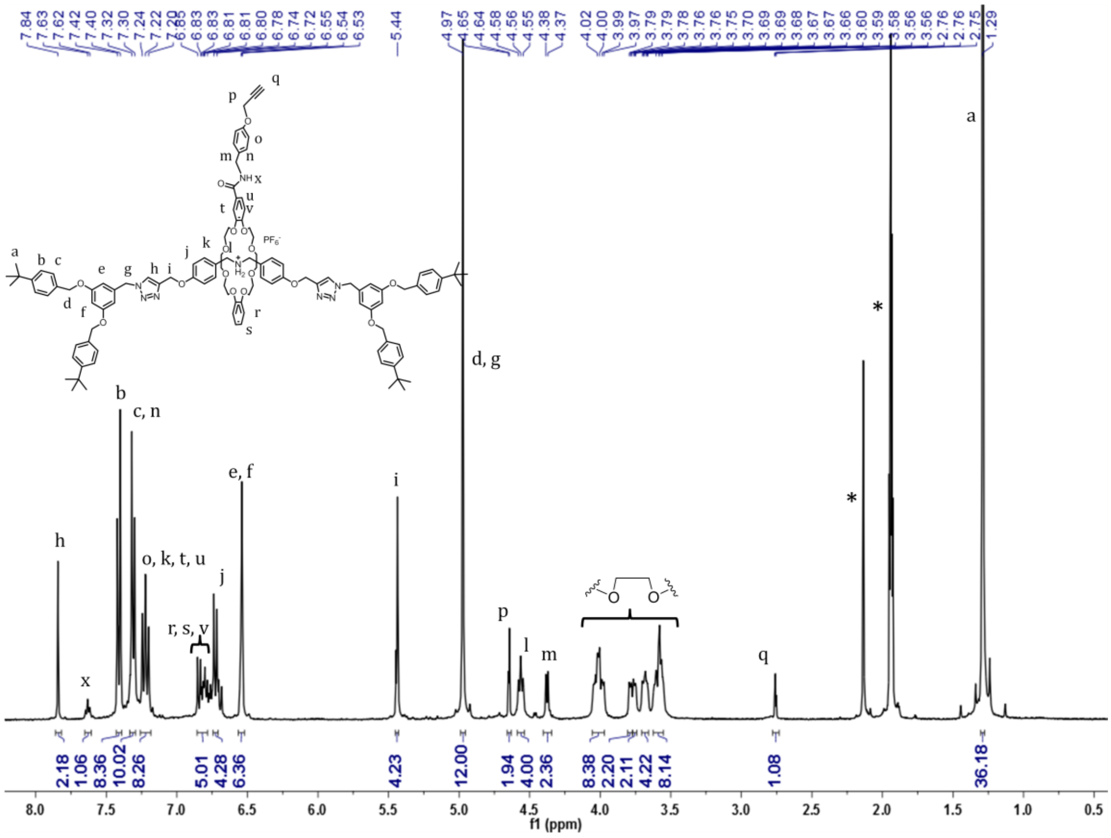

Supplementary Figure 94: ^{1}H NMR spectrum (400 MHz, CD_3CN) of G1 [2]Rotaxane Dendron-Acetylene (Asterisk: solvent residual signal).

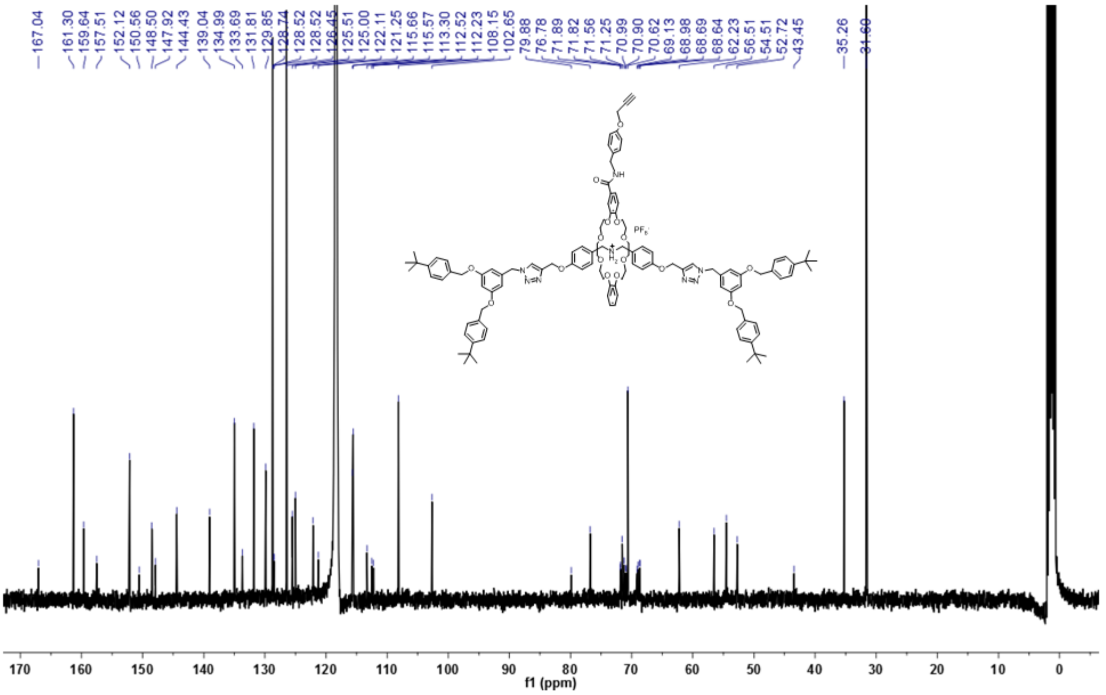

Supplementary Figure 95: ^{13}C NMR spectrum (101 MHz, CD_3CN) of G1 [2]Rotaxane Dendron-Acetylene.

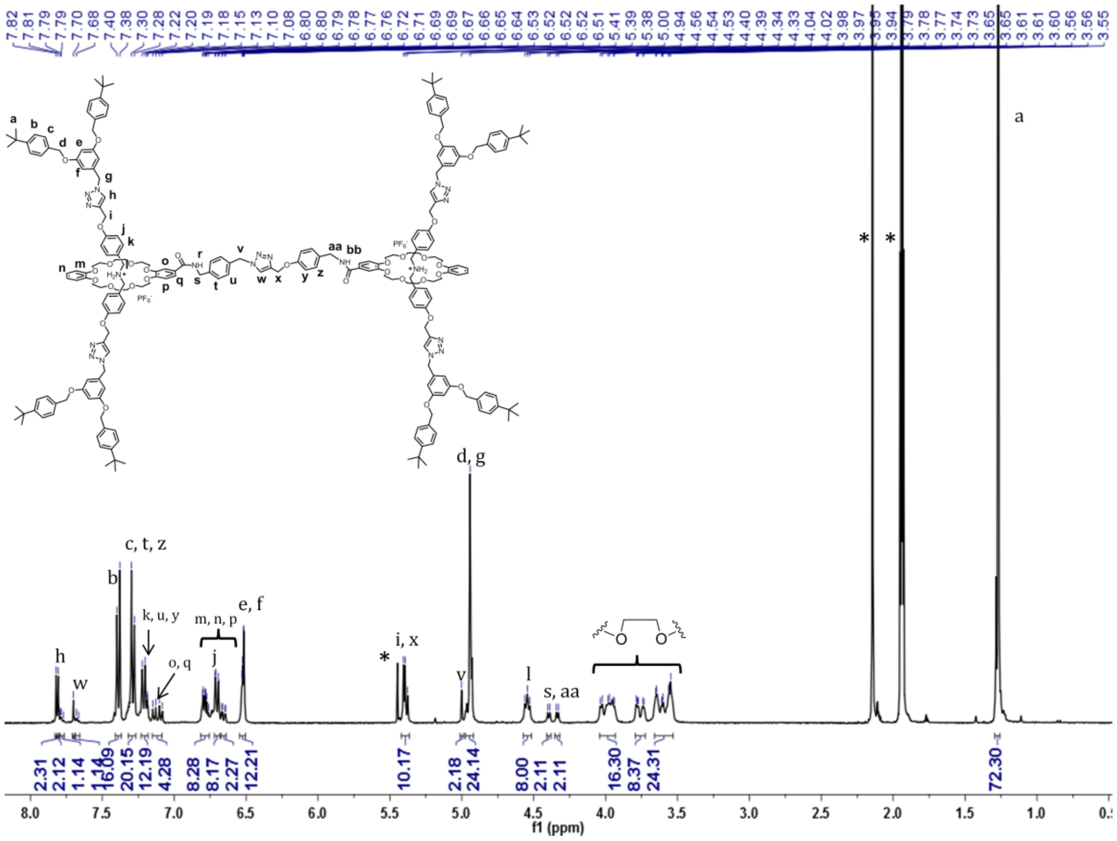

Supplementary Figure 96: ^{1}H NMR spectrum (400 MHz, CD_3CN) of G1 [3]Rotaxane Dendrimer (Asterisk: solvent residual signal).

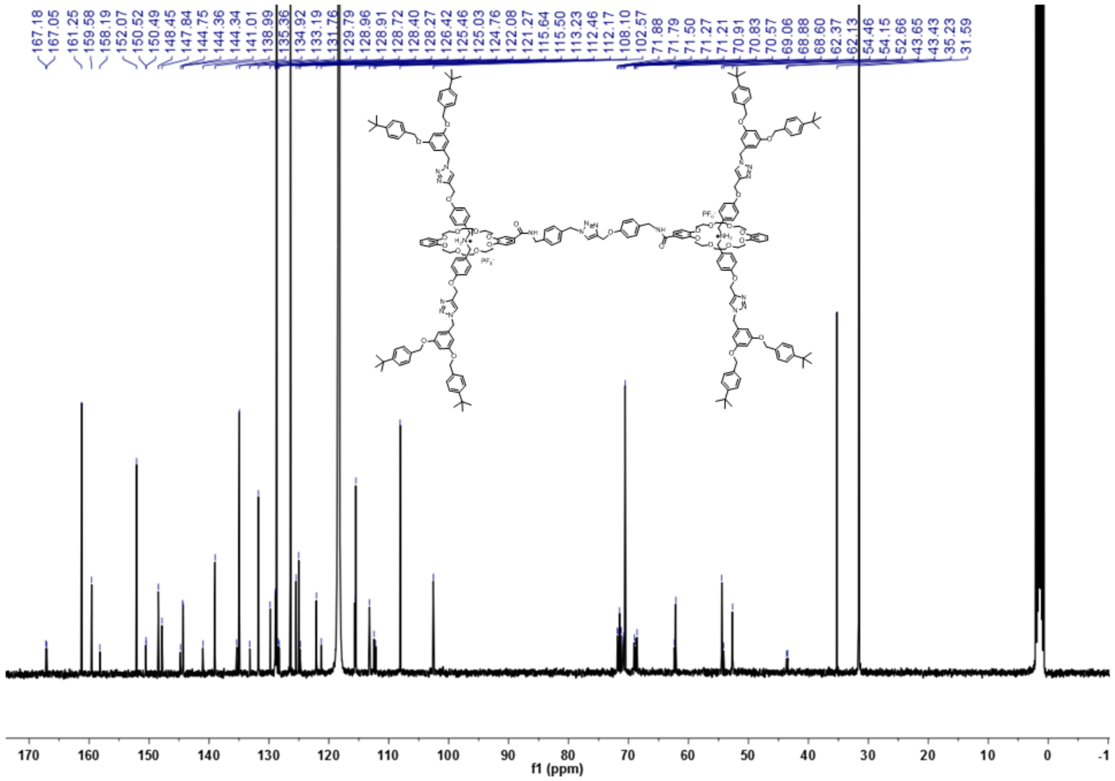

Supplementary Figure 97: ^{13}C NMR spectrum (101 MHz, CD_3CN) of G1 [3]Rotaxane Dendrimer.

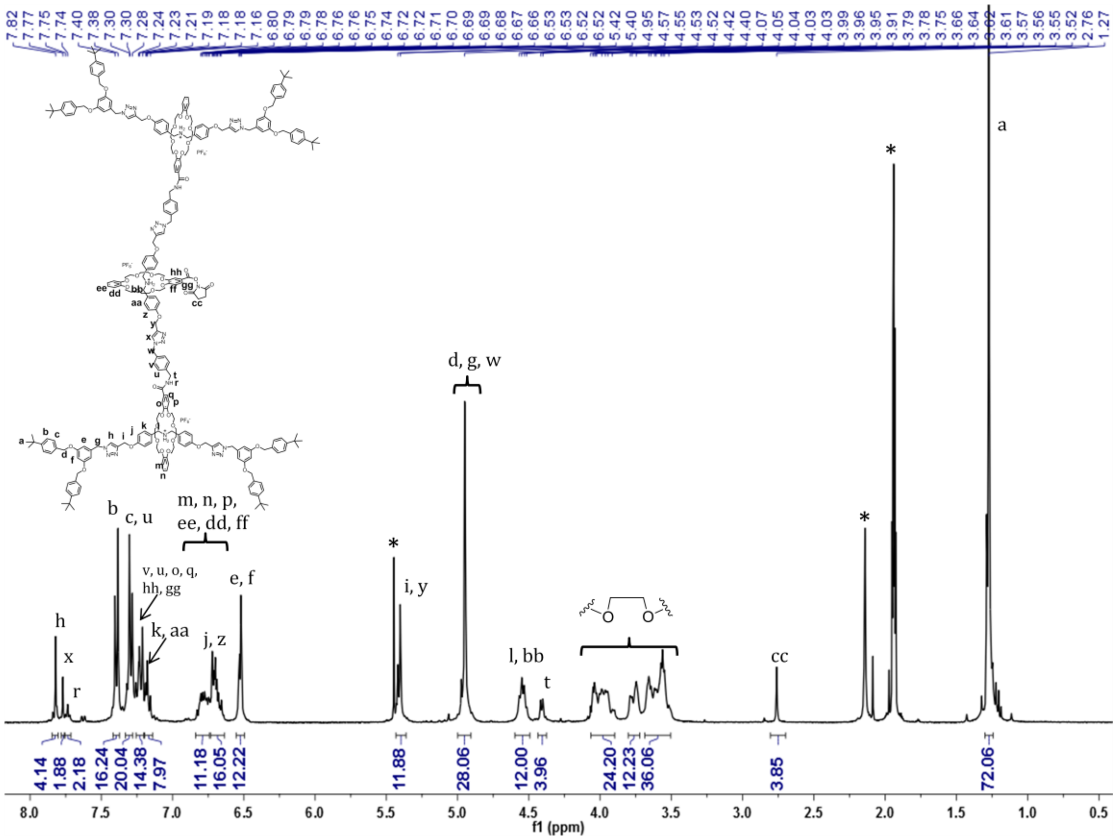

Supplementary Figure 98: ^{1}H NMR spectrum (400 MHz, CD_3CN) of G2 [4]Rotaxane Dendron-NHS (Asterisk: solvent residual signal).

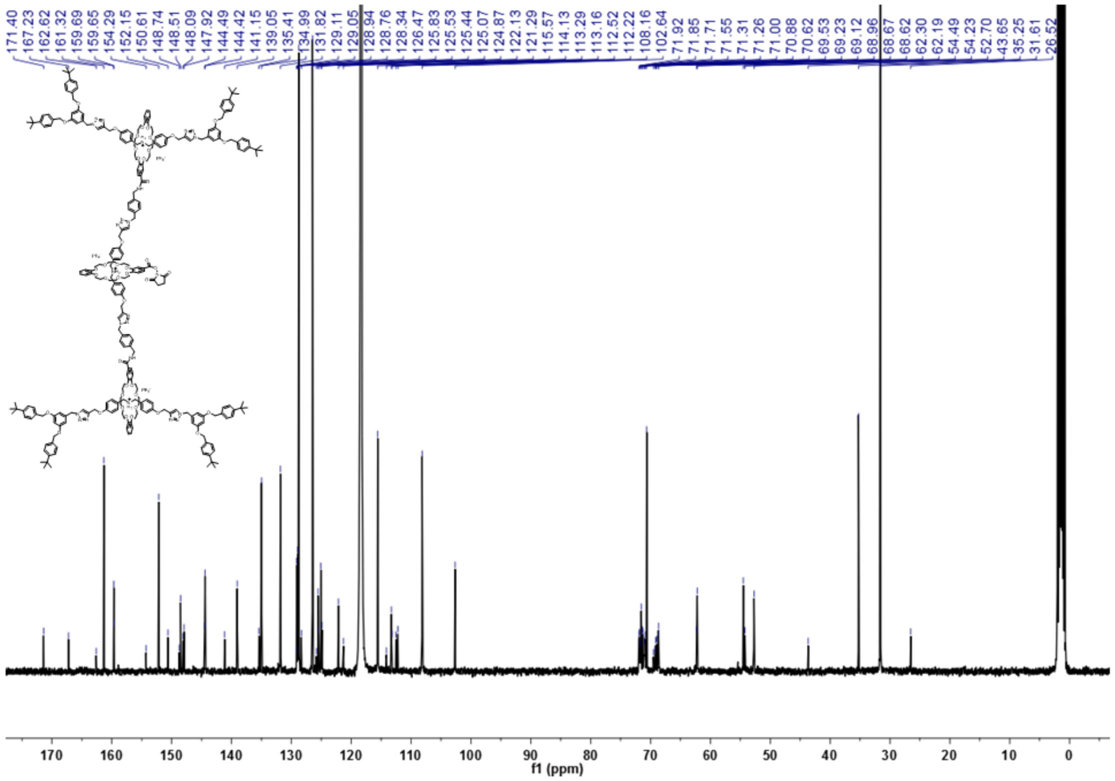

Supplementary Figure 99: ^{13}C NMR spectrum (101 MHz, CD_3CN) of G2 [4]Rotaxane Dendron-NHS.

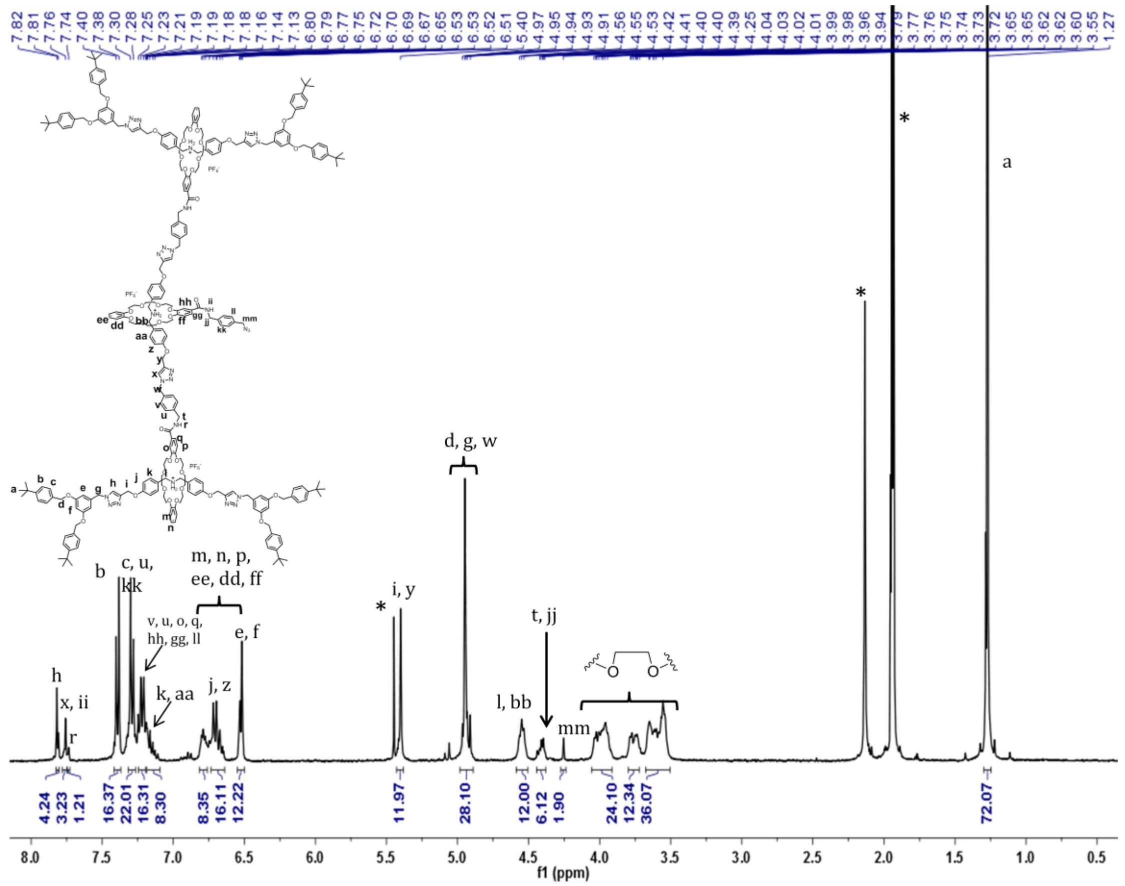

Supplementary Figure 100: ^{1}H NMR spectrum (400 MHz, CD_3CN) of G2 [4]Rotaxane Dendron-Azide (Asterisk: solvent residual signal).

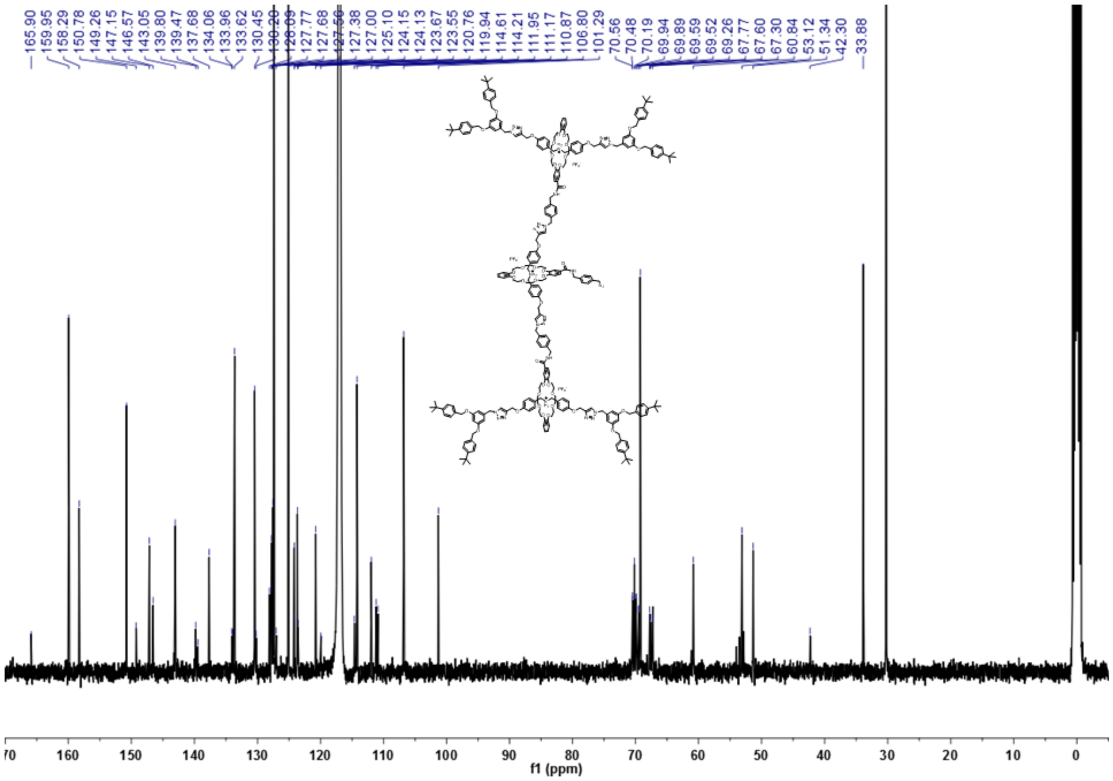

Supplementary Figure 101: ^{13}C NMR spectrum (101 MHz, CD_3CN) of G2 [4]Rotaxane Dendron-Azide.

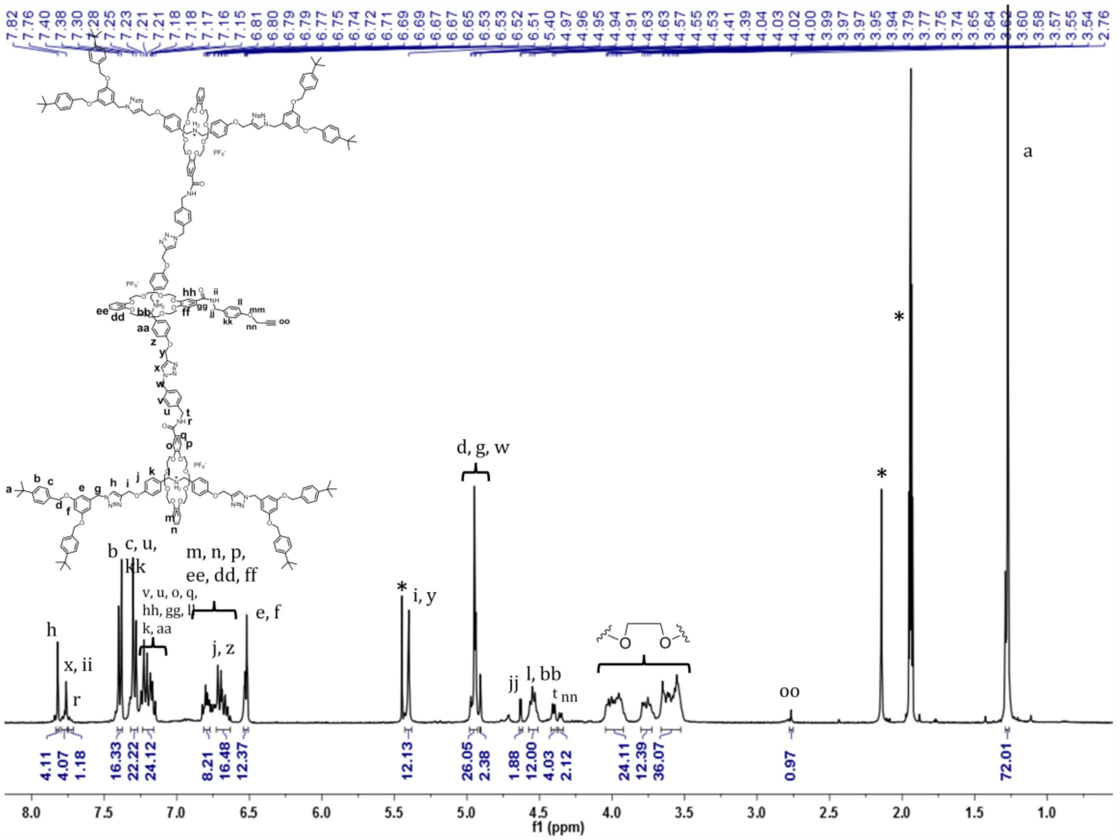

Supplementary Figure 102: ^{1}H NMR spectrum (400 MHz, CD_3CN) of G2 [4]Rotaxane Dendron-Acetylene (Asterisk: solvent residual signal).

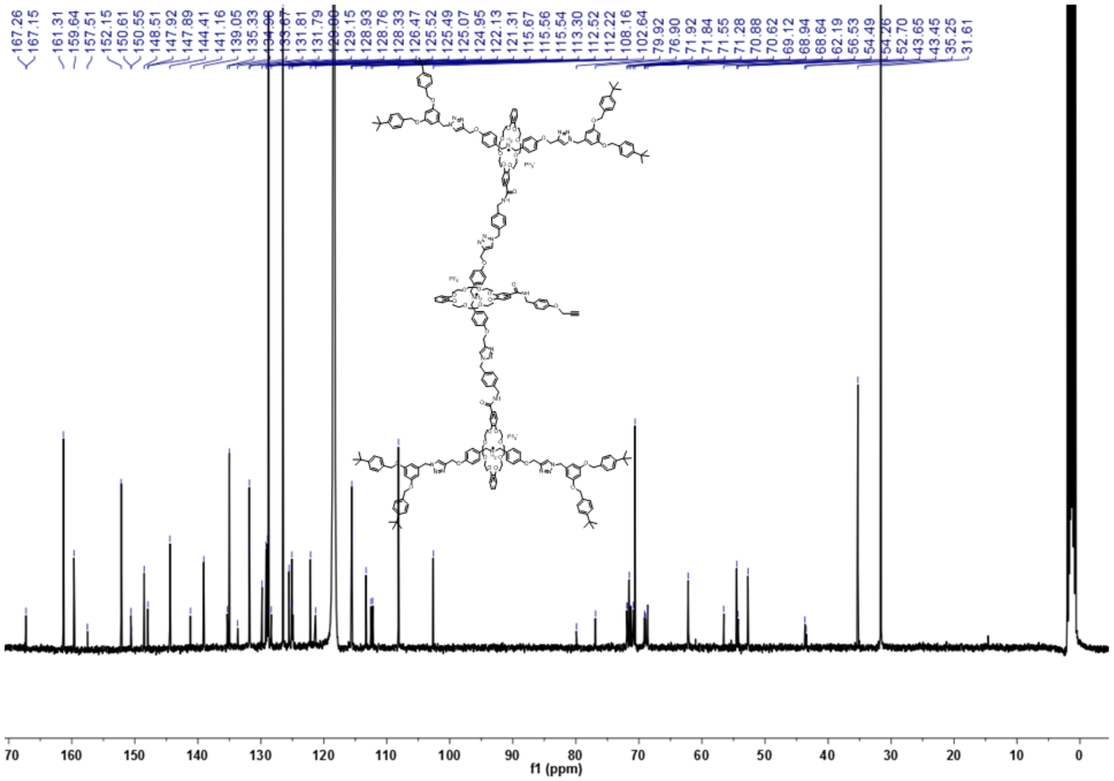

Supplementary Figure 103: ^{13}C NMR spectrum (101 MHz, CD_3CN) of G2 [4]Rotaxane Dendron-Acetylene.

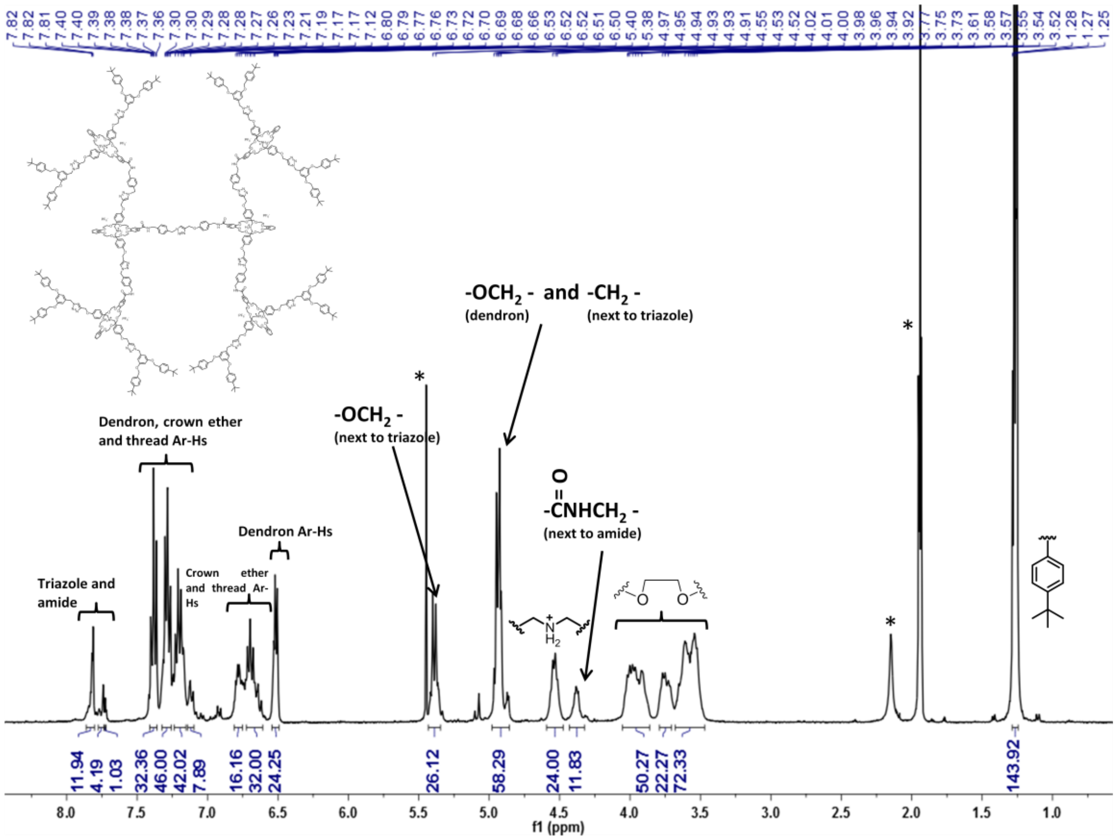

Supplementary Figure 104: ^{1}H NMR spectrum (400 MHz, CD_3CN) of G2 [7]Rotaxane Dendrimer (Asterisk: solvent residual signal).

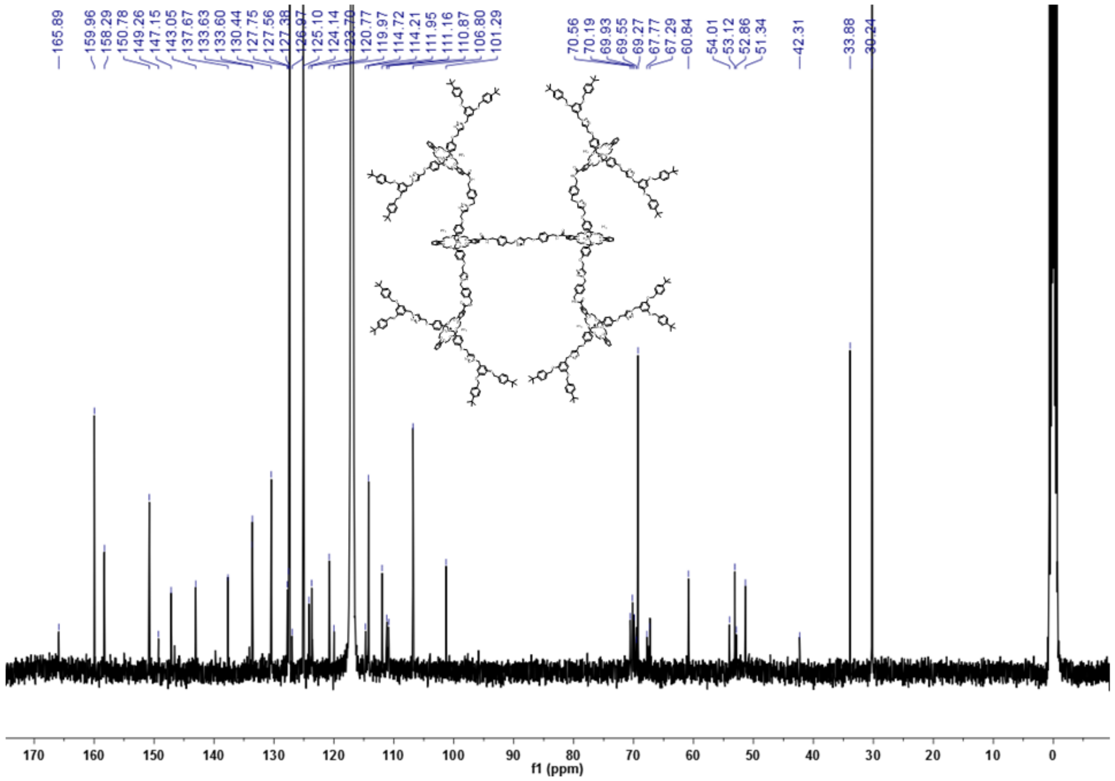

Supplementary Figure 105: ^{13}C NMR spectrum (101 MHz, CD_3CN) of G2 [7]Rotaxane Dendrimer.

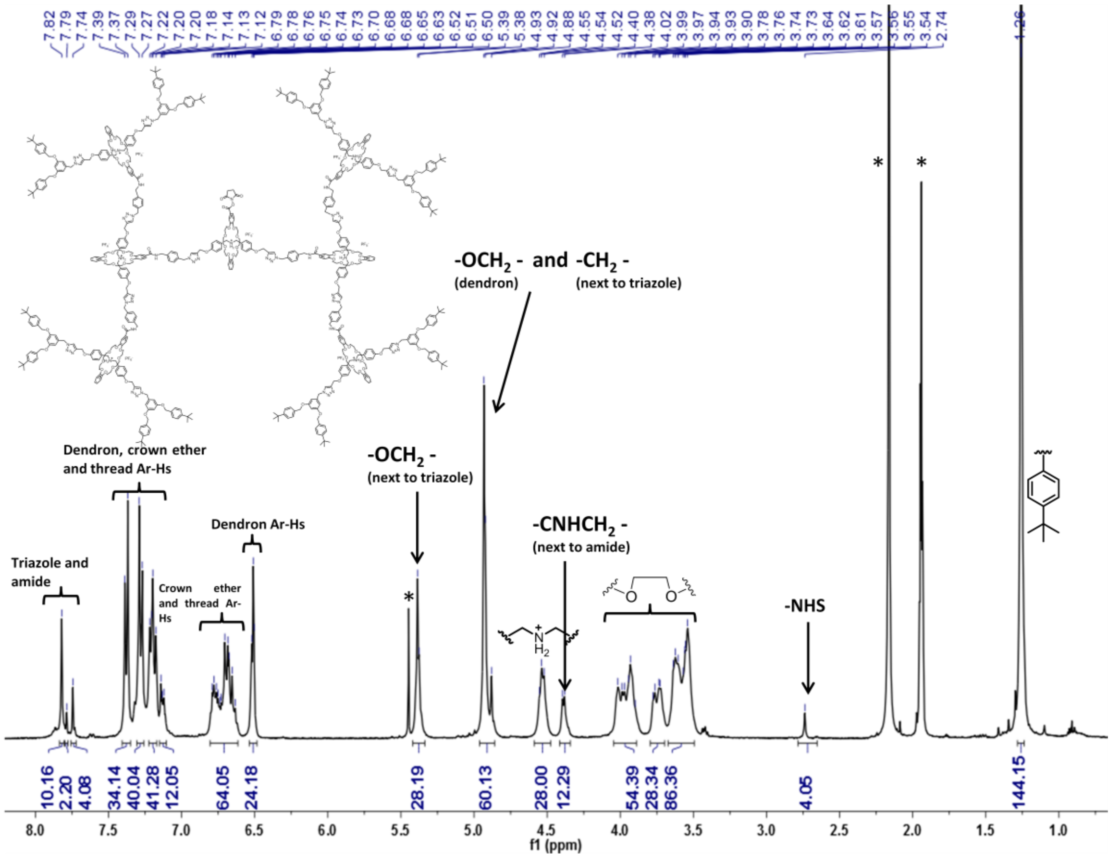

Supplementary Figure 106: ^{1}H NMR spectrum (400 MHz, CD_3CN) of G3 [8]Rotaxane Dendron-NHS (Asterisk: solvent residual signal).

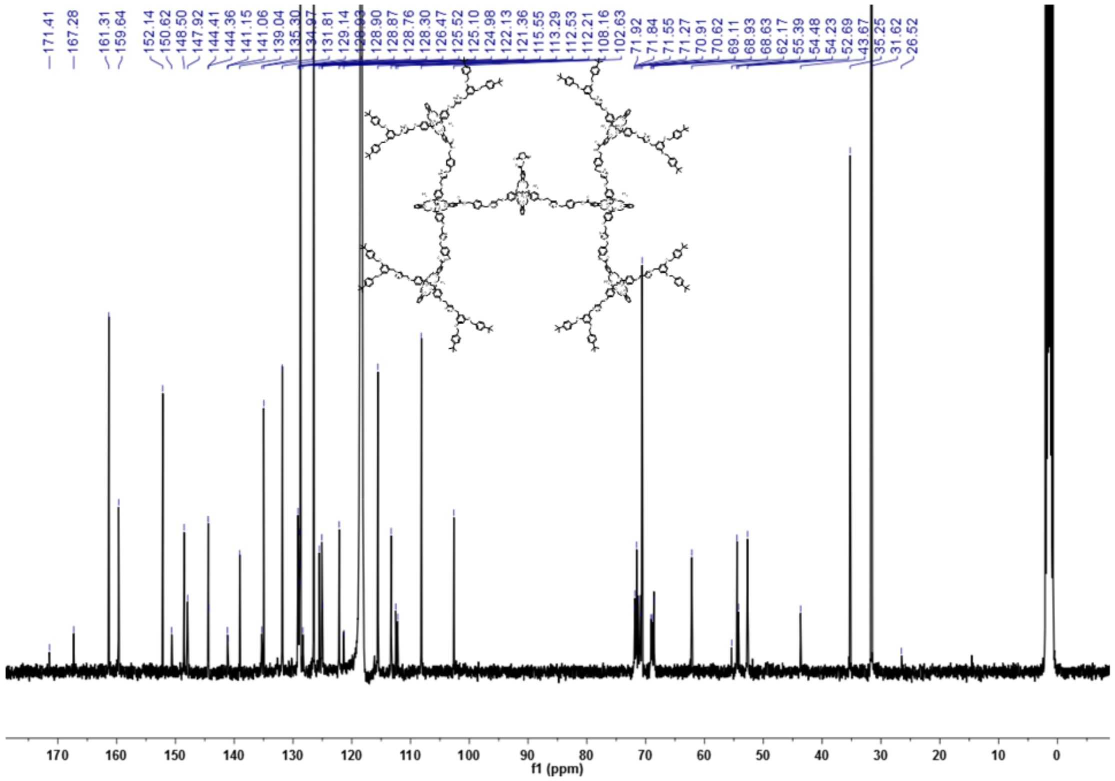

Supplementary Figure 107: ^{13}C NMR spectrum (101 MHz, CD_3CN) of G3 [8]Rotaxane Dendron-NHS (Asterisk: solvent residual signal).

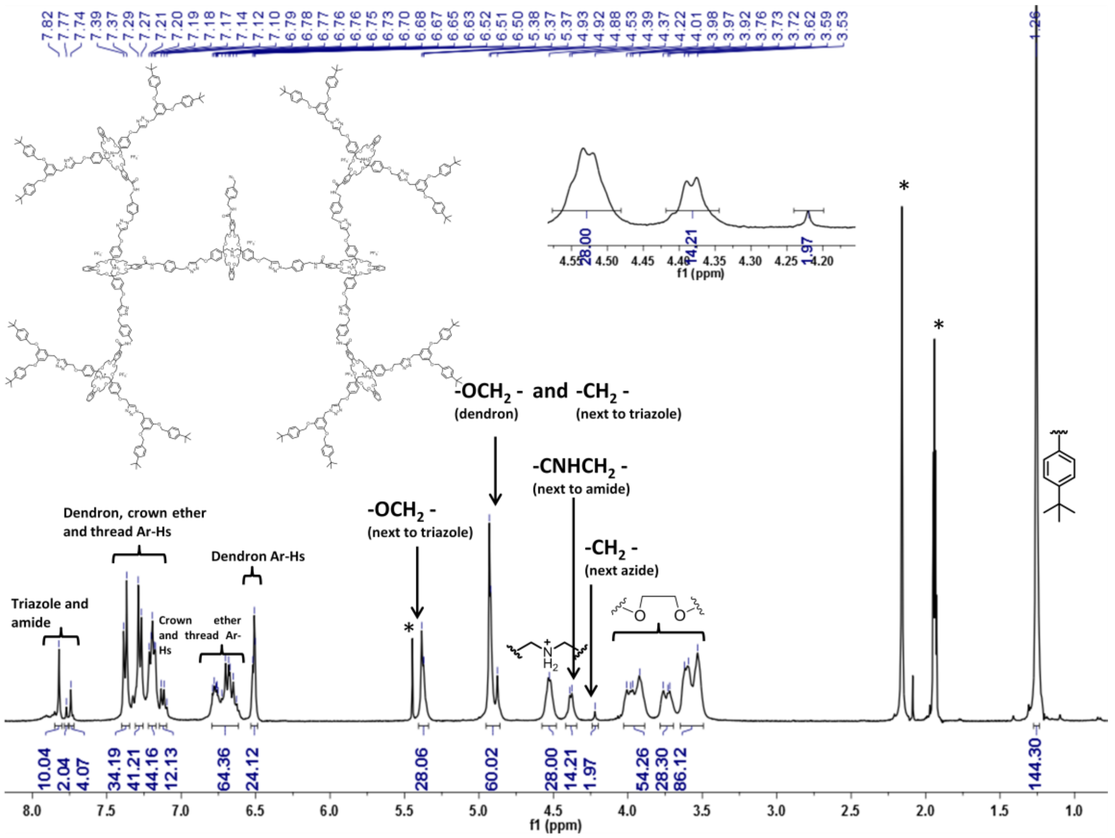

Supplementary Figure 108: ^{1}H NMR spectrum (400 MHz, CD_3CN) of G3 [8]Rotaxane Dendron-Azide (Asterisk: solvent residual signal). Inset is the enlarged region from 4.10 – 4.60 ppm.

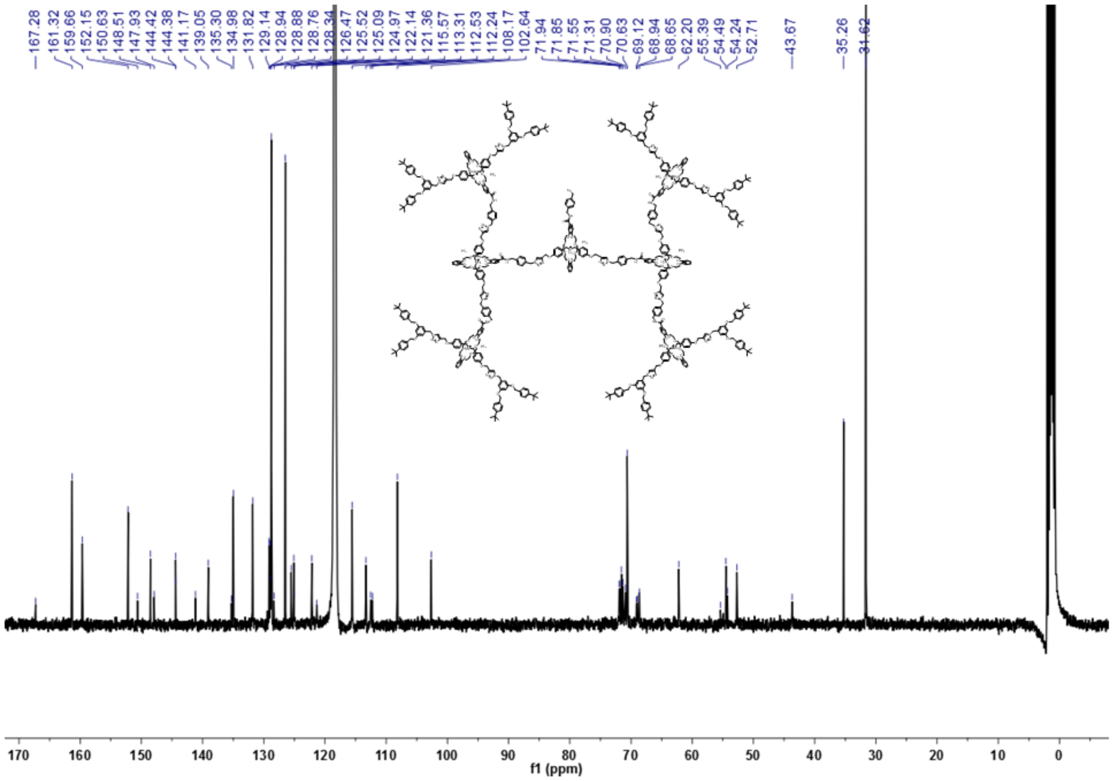

Supplementary Figure 109: ^{13}C NMR spectrum (101 MHz, CD_3CN) of G3 [8]Rotaxane Dendron-Azide.

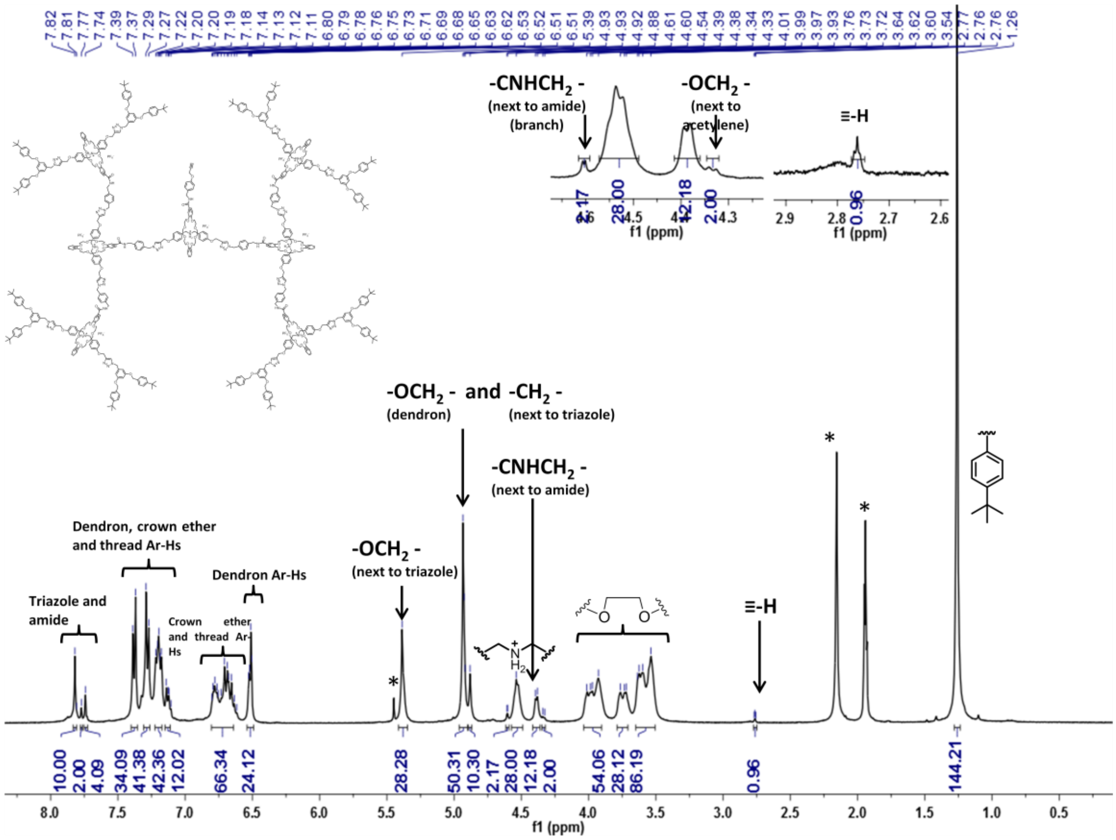

Supplementary Figure 110: ^{1}H NMR spectrum (400 MHz, CD_3CN) of G3 [8]Rotaxane Dendron-Acetylene (Asterisk: solvent residual signal). Insert is the enlarged region from 2.6 – 2.9 ppm and 4.2 – 4.7 ppm.

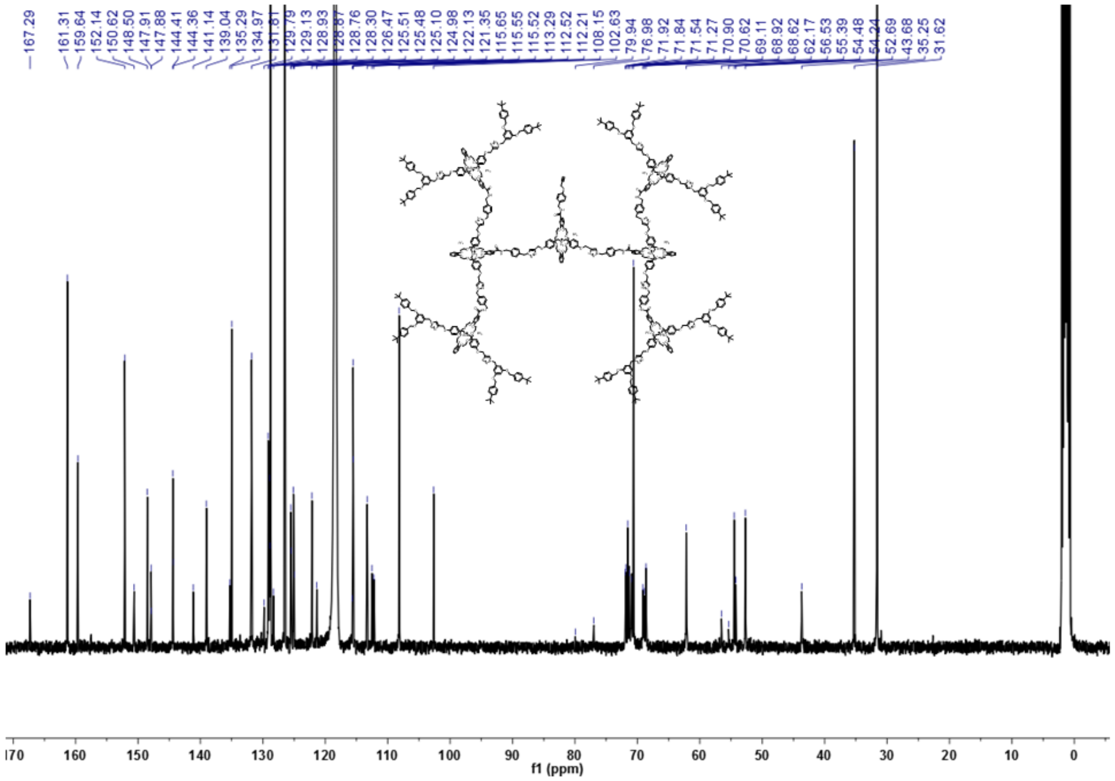

Supplementary Figure 111: ^{13}C NMR spectrum (101 MHz, CD_3CN) of G3 [8]Rotaxane Dendron-Acetylene.

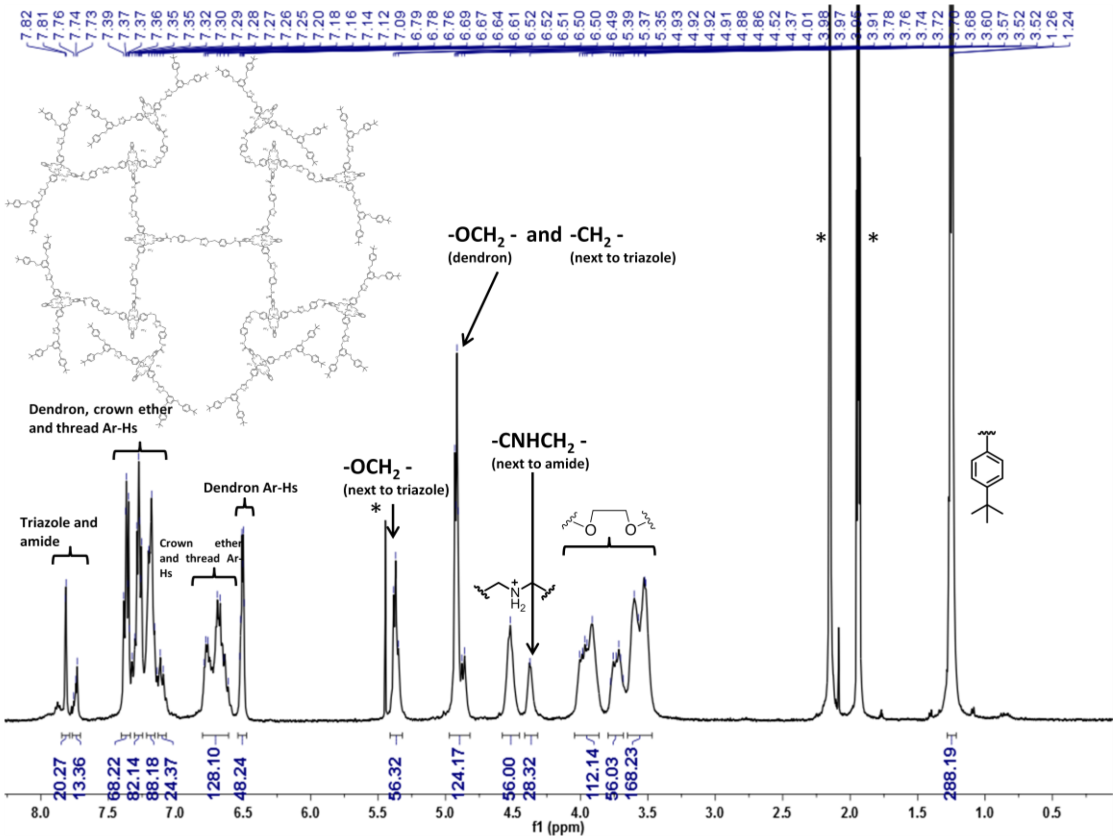

Supplementary Figure 112: ^{1}H NMR spectrum (400 MHz, CD_3CN) of G3 [15]Rotaxane Dendrimer (Asterisk: solvent residual signal).

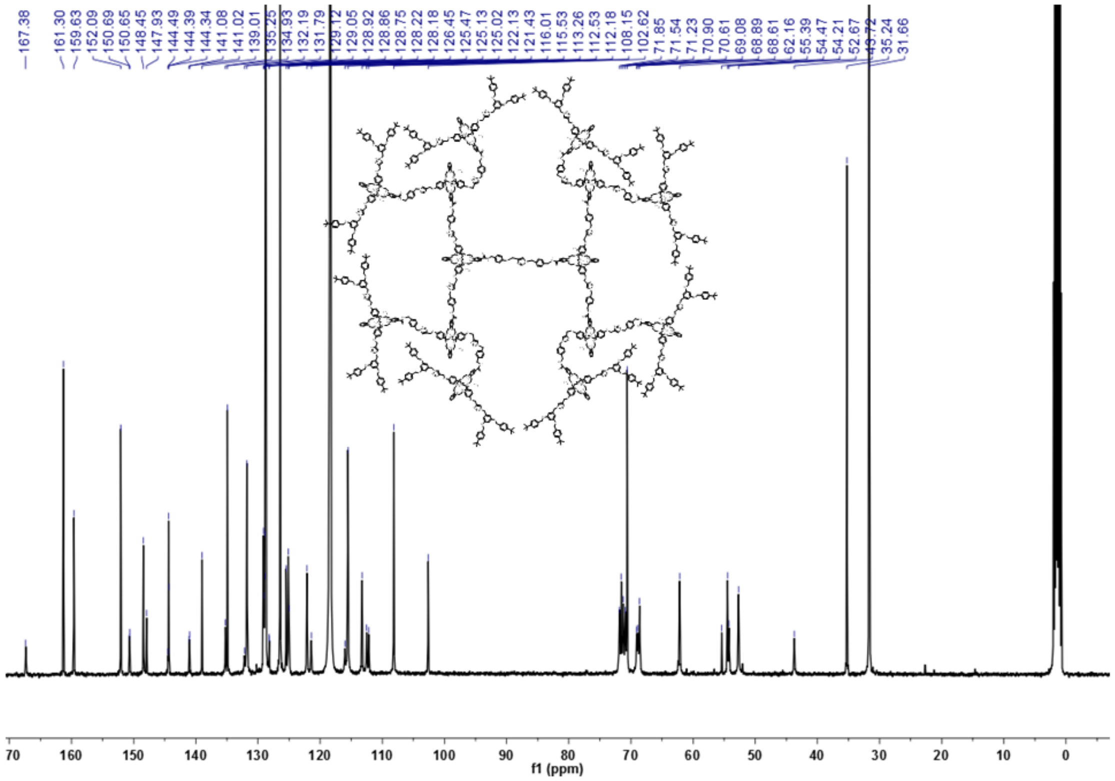

Supplementary Figure 113: ^{13}C NMR spectrum (101 MHz, CD_3CN) of G3 [15]Rotaxane Dendrimer.

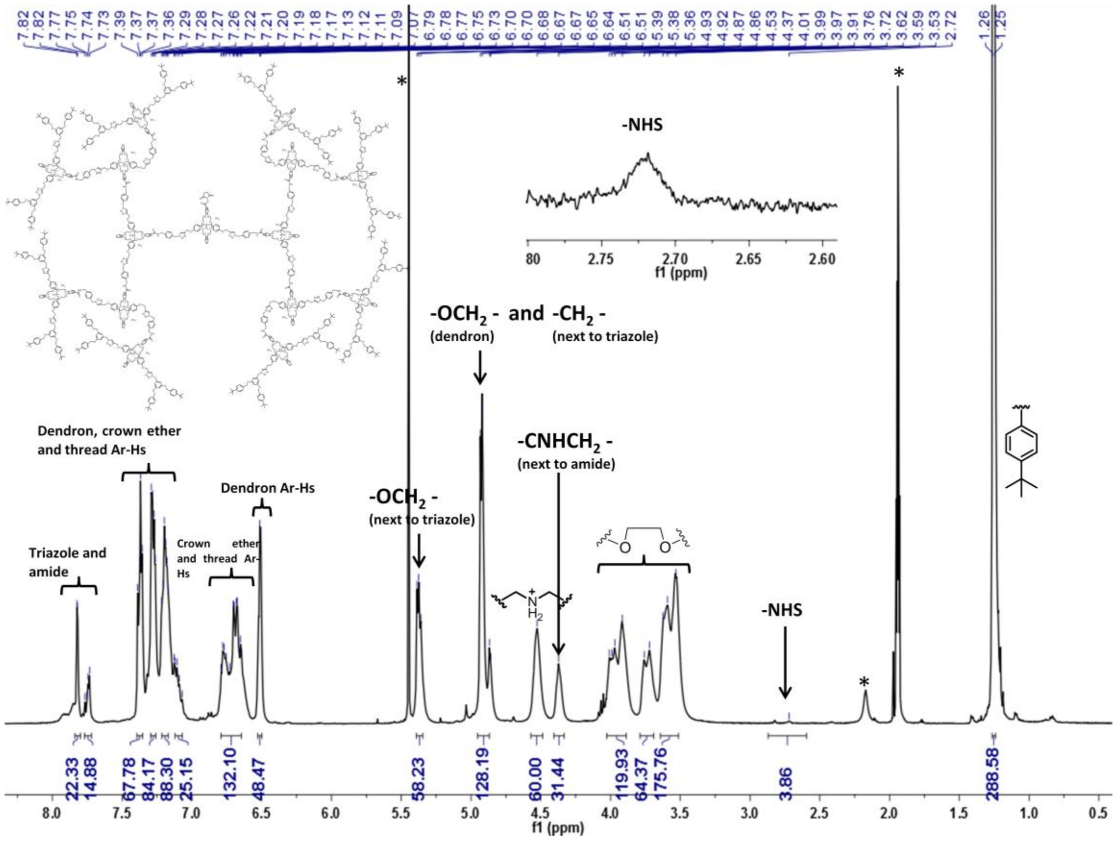

Supplementary Figure 114: ^{1}H NMR spectrum (400 MHz, CD_3CN) of G4 [16]Rotaxane Dendron. Insert is the enlarged region from 2.58 – 2.8 ppm (Asterisk: solvent residual signal).

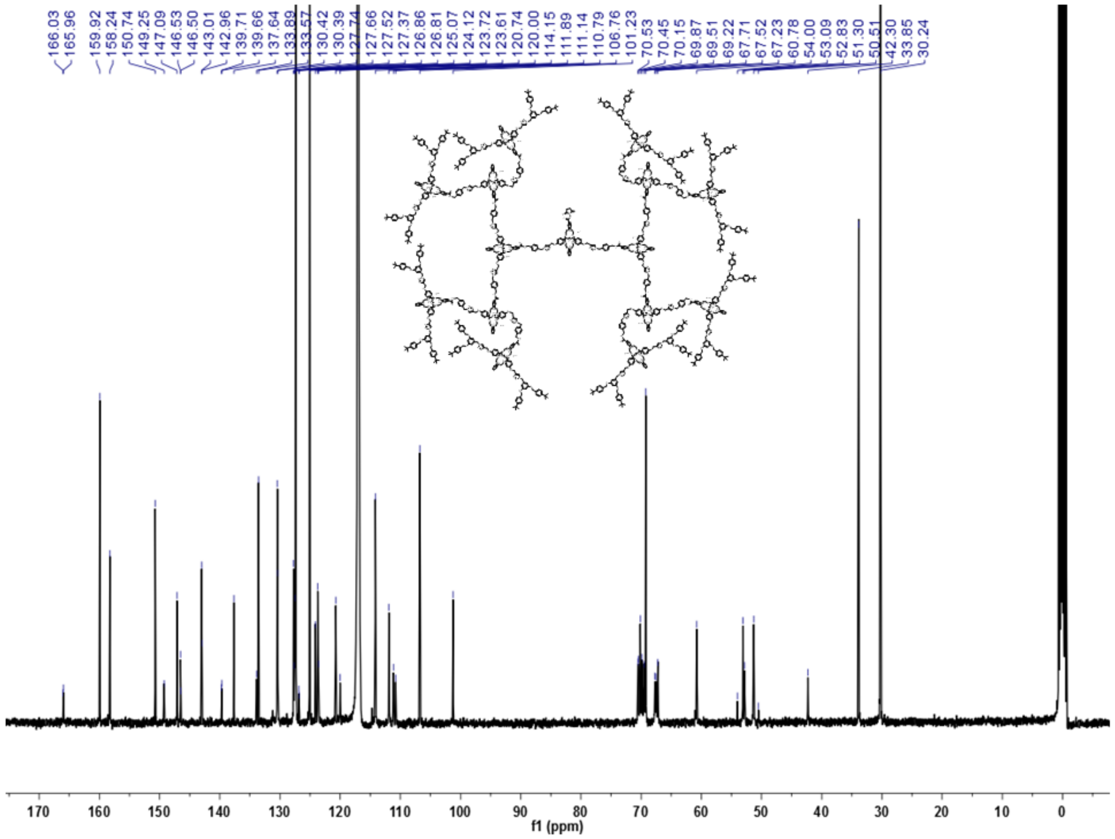

Supplementary Figure 115: ^{13}C NMR spectrum (101 MHz, CD_3CN) of G4 [16]Rotaxane Dendron.

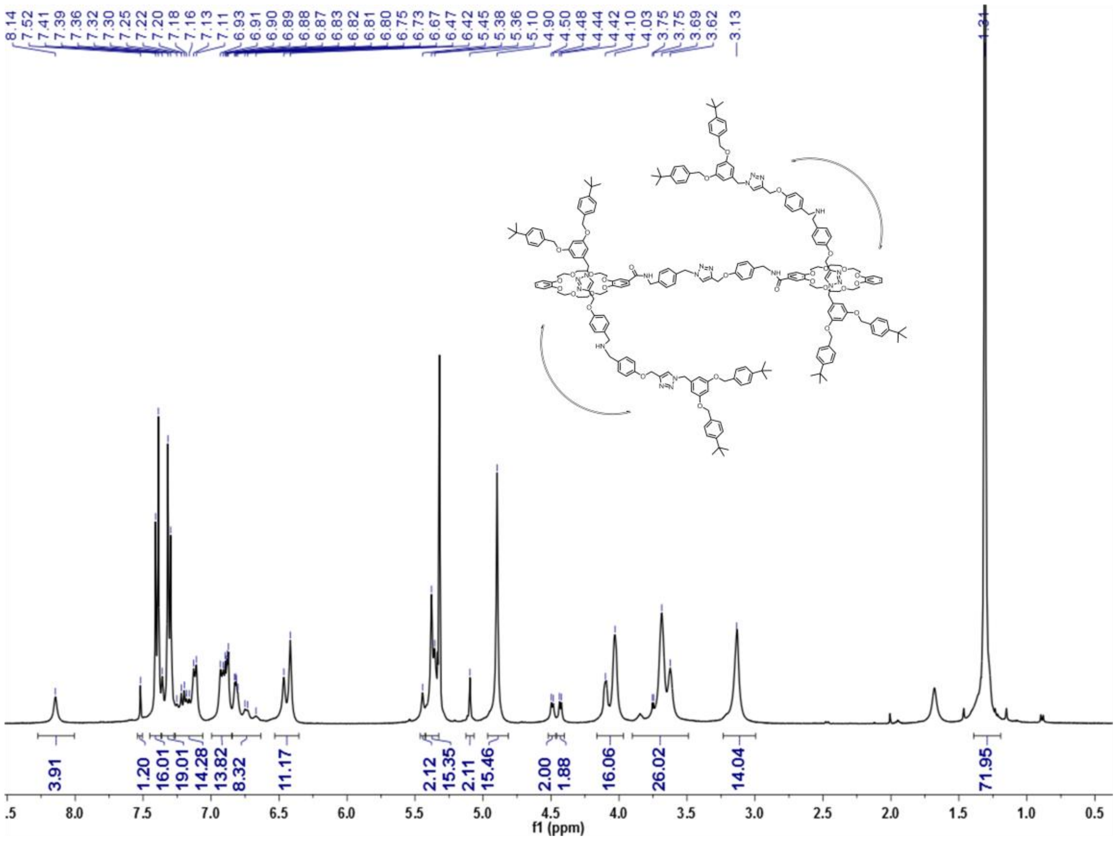

Supplementary Figure 116: ^{1}H NMR spectrum (400 MHz, CD_2Cl_2) of Neutral G1 [3]Rotaxane Dendrimer.

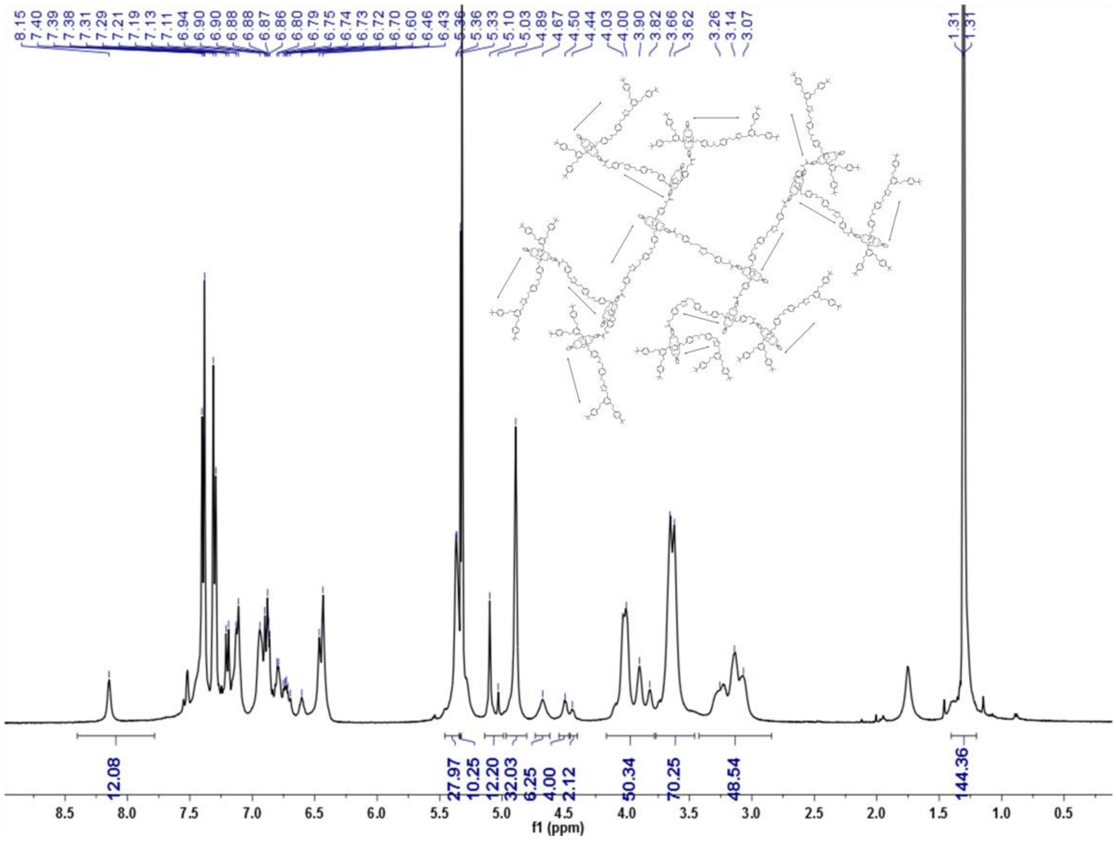

Supplementary Figure 117: ^{1}H NMR spectrum (400 MHz, CD_2Cl_2) of Neutral G2 [7]Rotaxane Dendrimer.

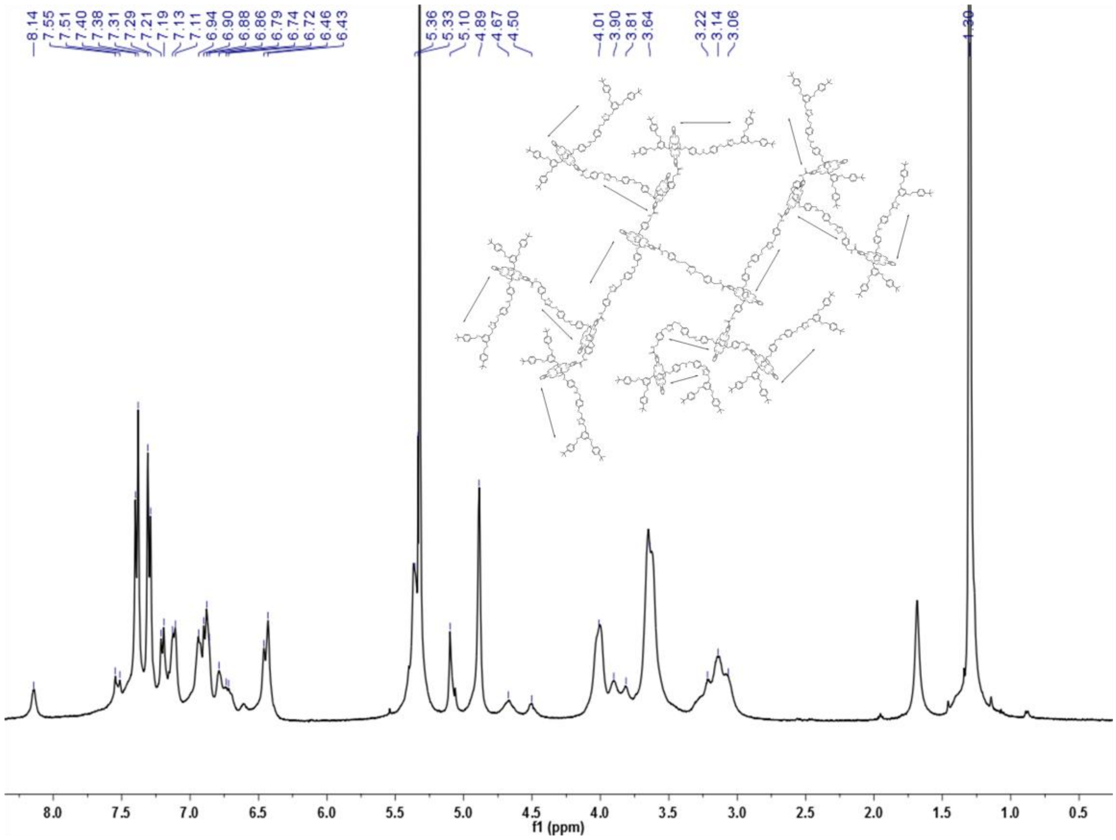

Supplementary Figure 118: ^{1}H NMR spectrum (400 MHz, CD_2Cl_2) of Neutral G3 [15]Rotaxane Dendrimer.

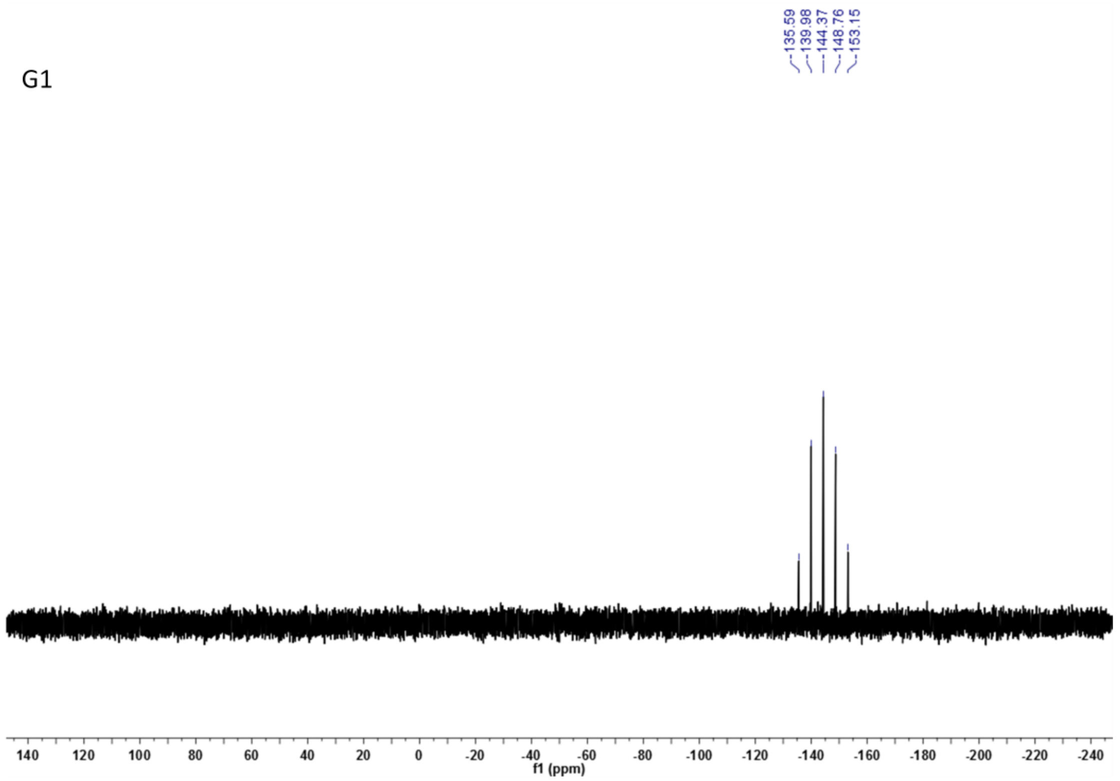

Supplementary Figure 119: ^{31}P NMR spectrum (162 MHz, CD_2Cl_2) of G1 [3]Rotaxane Dendrimer.

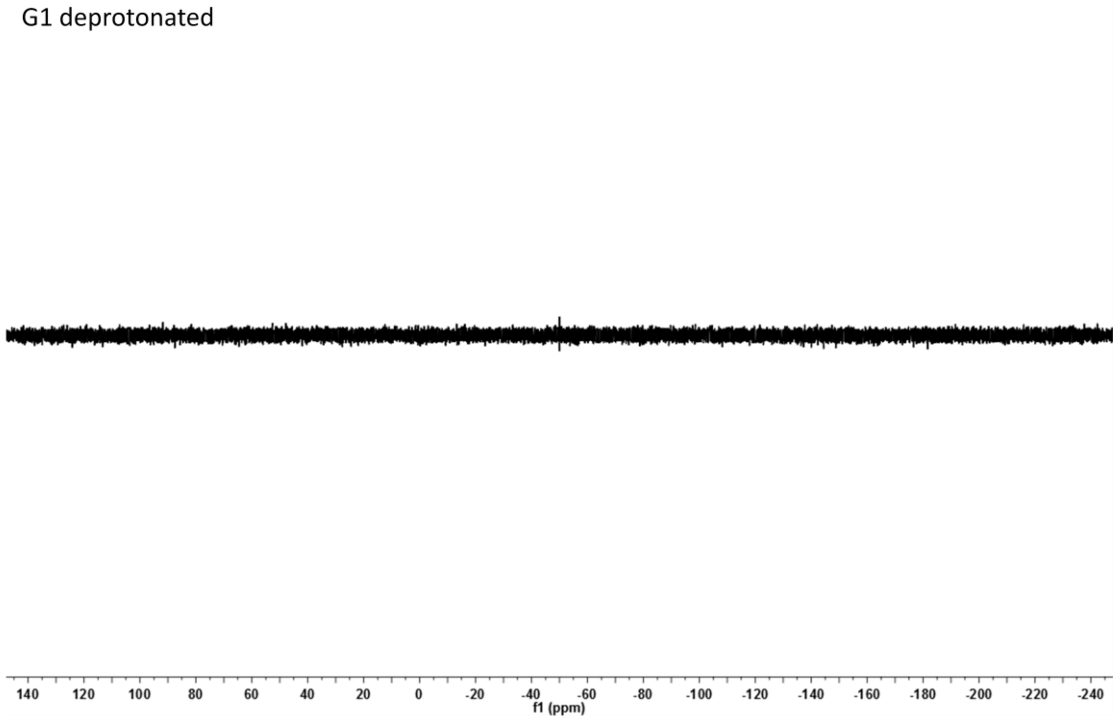

Supplementary Figure 120: ^{31}P NMR spectrum (162 MHz, CD_2Cl_2) of Neutral G1

[3]Rotaxane Dendrimer.

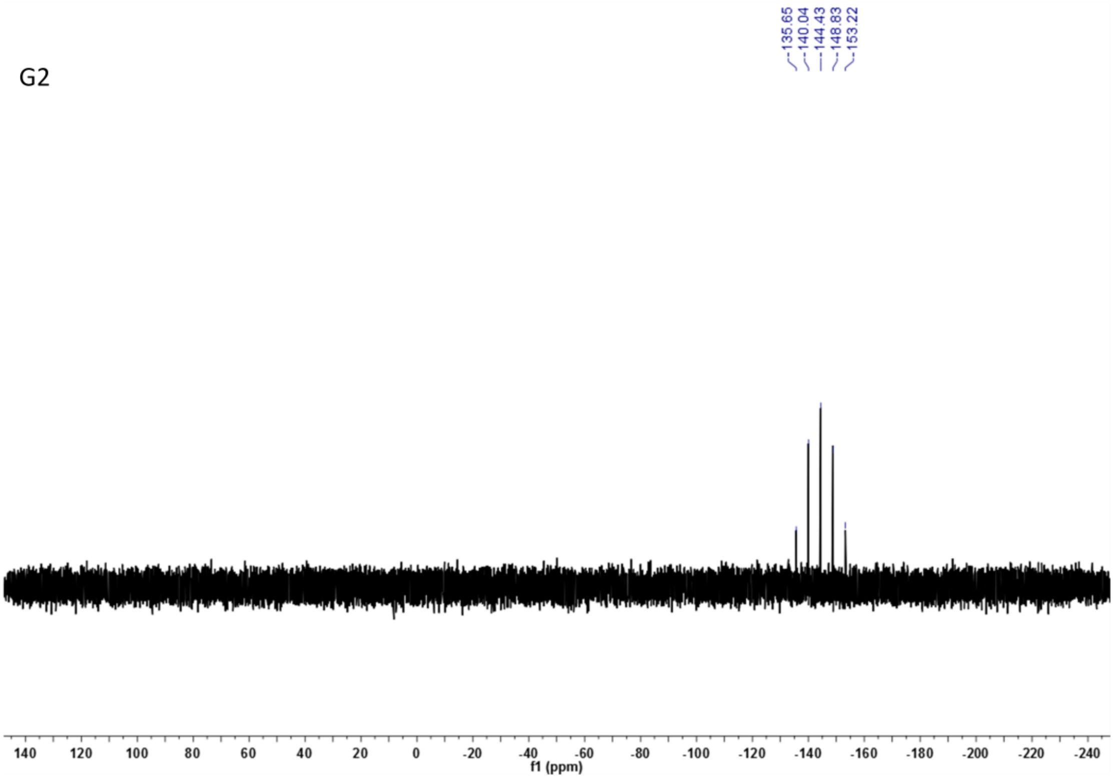

Supplementary Figure 121: ^{31}P NMR spectrum (162 MHz, CD_2Cl_2) of G2 [7]Rotaxane Dendrimer.

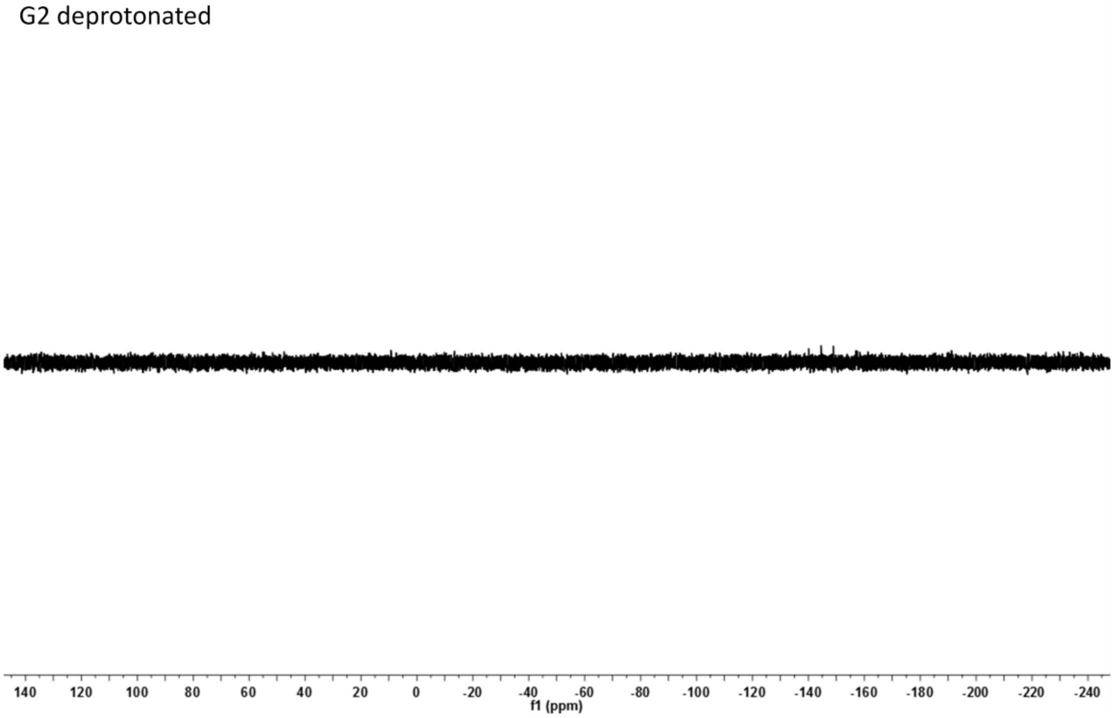

Supplementary Figure 122: ^{31}P NMR spectrum (162 MHz, CD_2Cl_2) of Neutral G2 [7]Rotaxane Dendrimer.

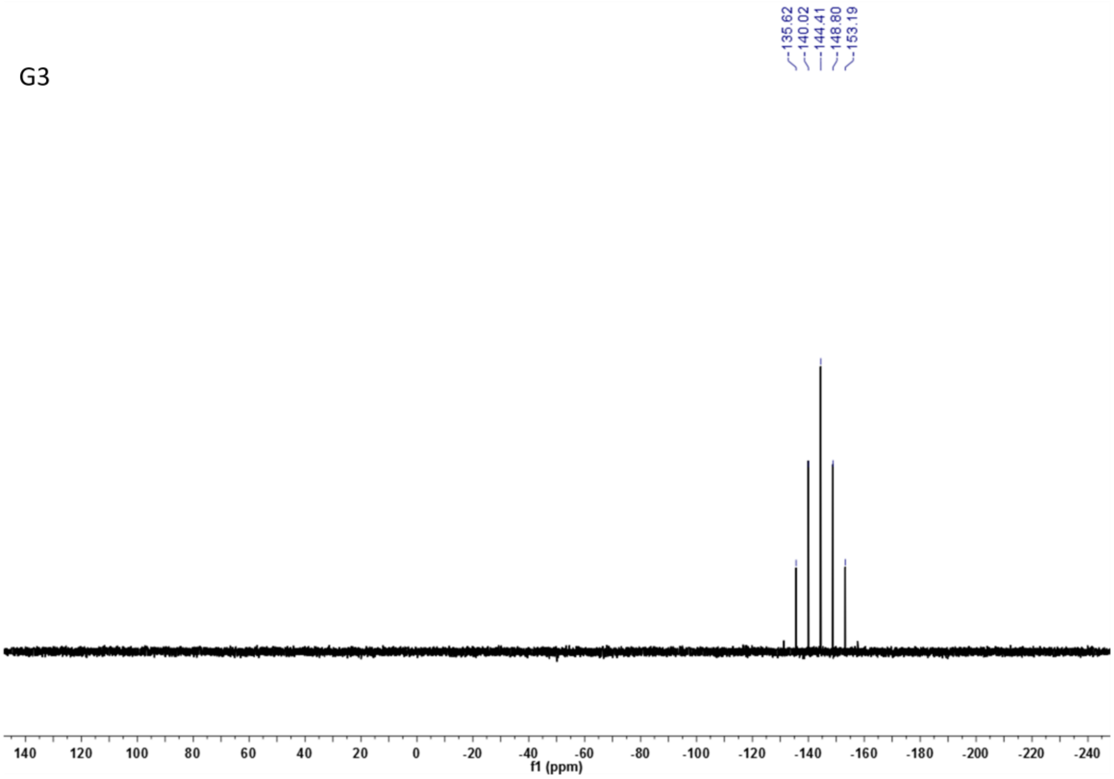

Supplementary Figure 123: ^{31}P NMR spectrum (162 MHz, CD_2Cl_2) of G3 [15]Rotaxane Dendrimer.

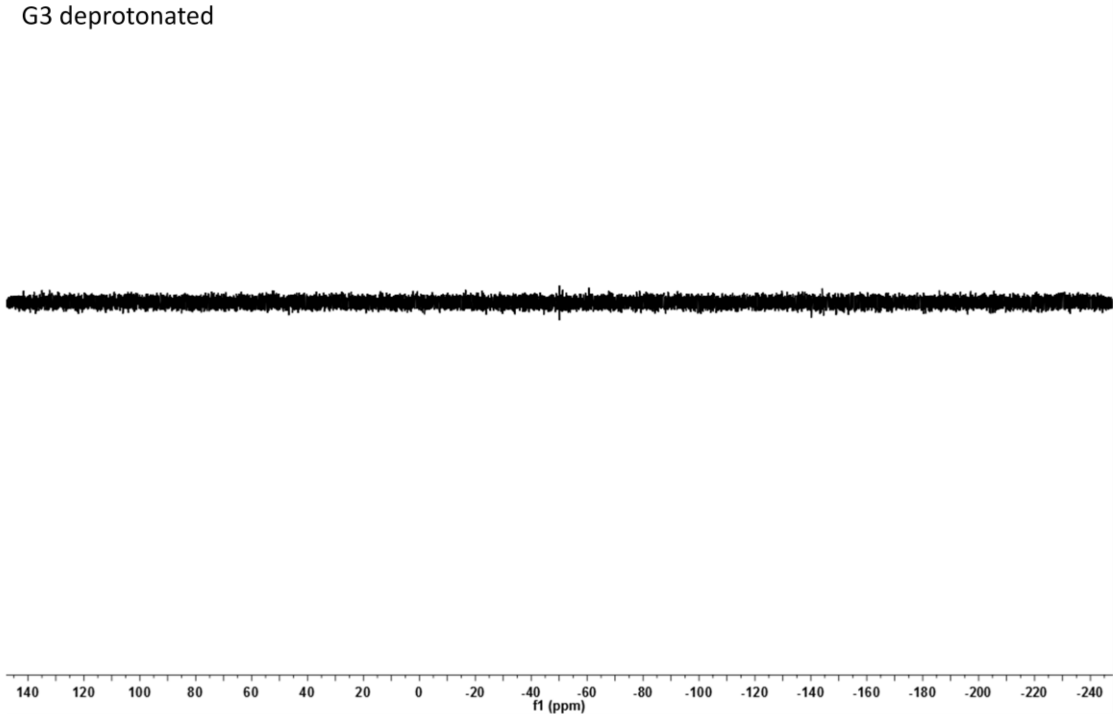

Supplementary Figure 124: ^{31}P NMR spectrum (162 MHz, CD_2Cl_2) of Neutral G3 [15]Rotaxane Dendrimer.

Mass Spectra of Selected Compounds

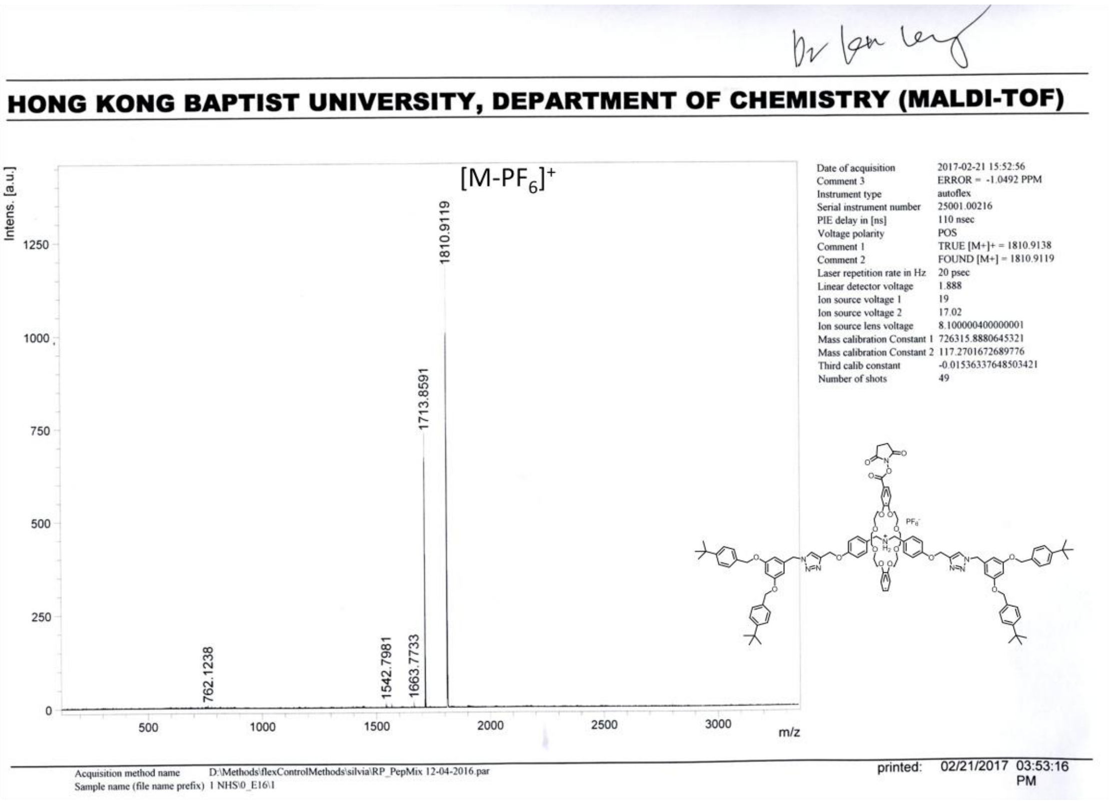

Supplementary Figure 125: HRMS MALDI-TOF of G1 [2]Rotaxane Dendron-NHS.

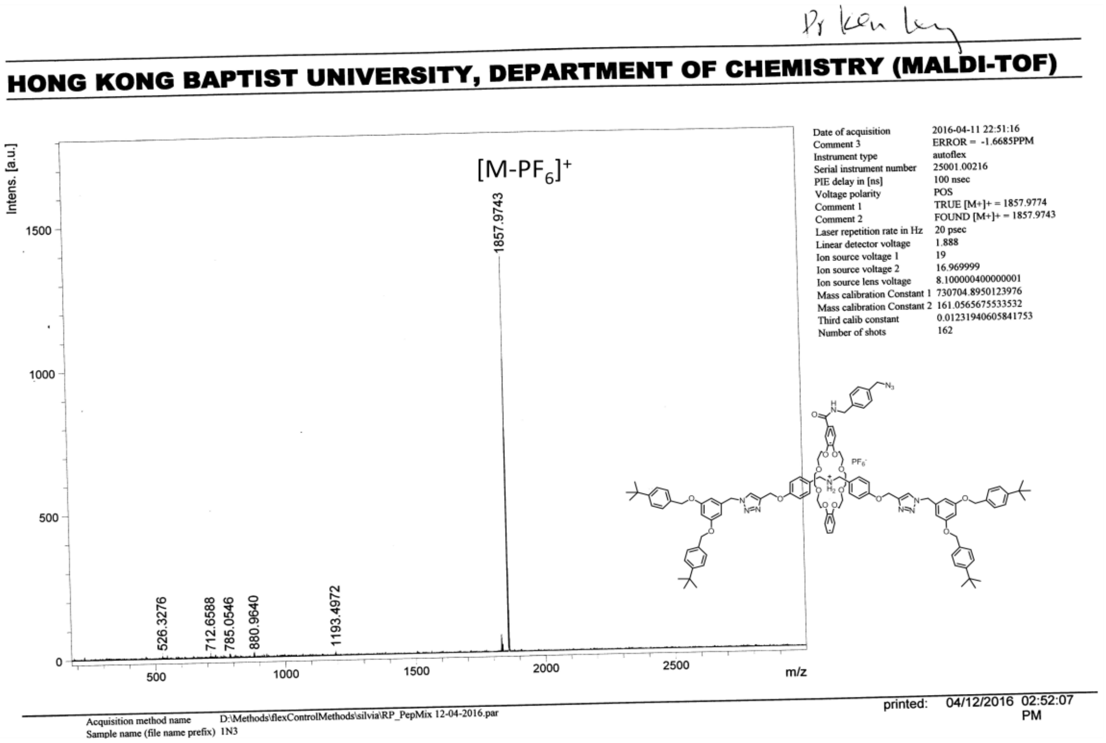

Supplementary Figure 126: HRMS MALDI-TOF of G1 [2]Rotaxane Dendron-Azide.

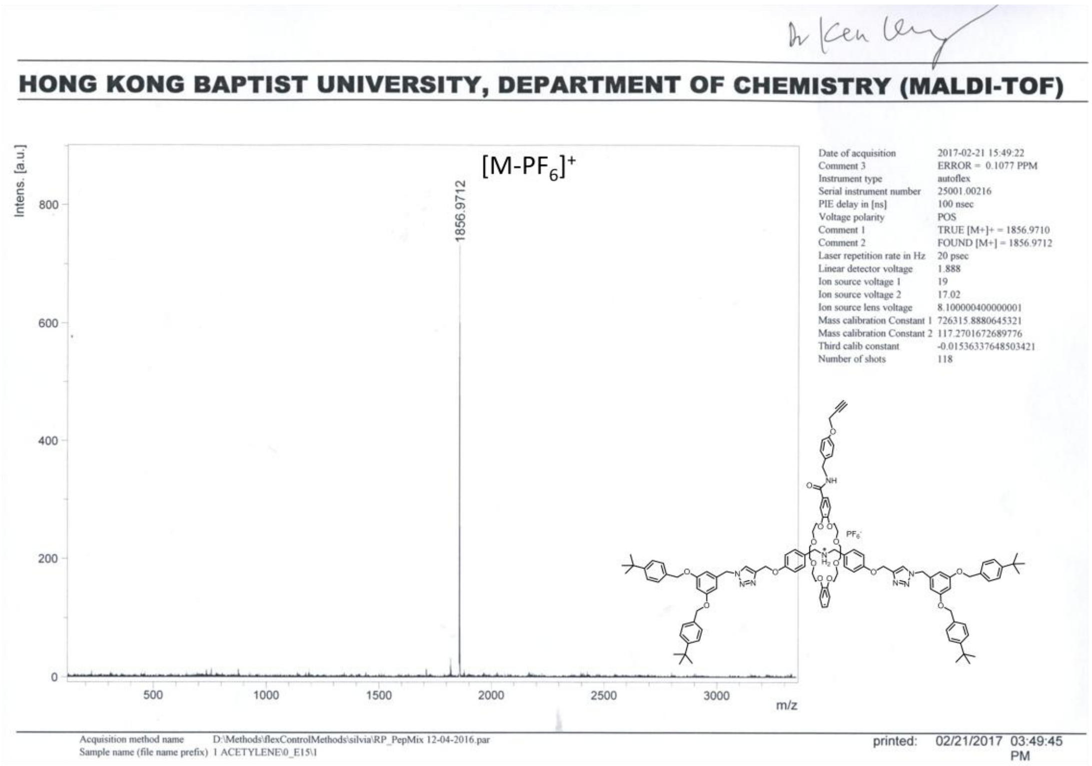

Supplementary Figure 127: HRMS MALDI-TOF of G1 [2]Rotaxane Dendron-Acetylene.

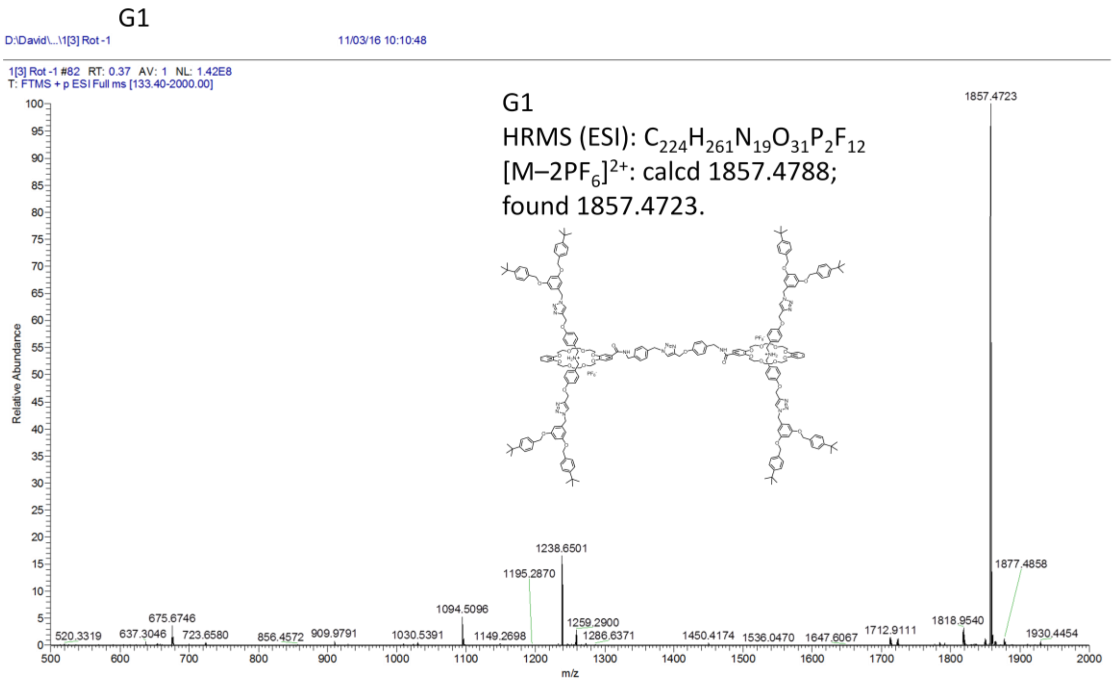

Supplementary Figure 128: HRMS ESI of G1 [3]Rotaxane Dendrimer.

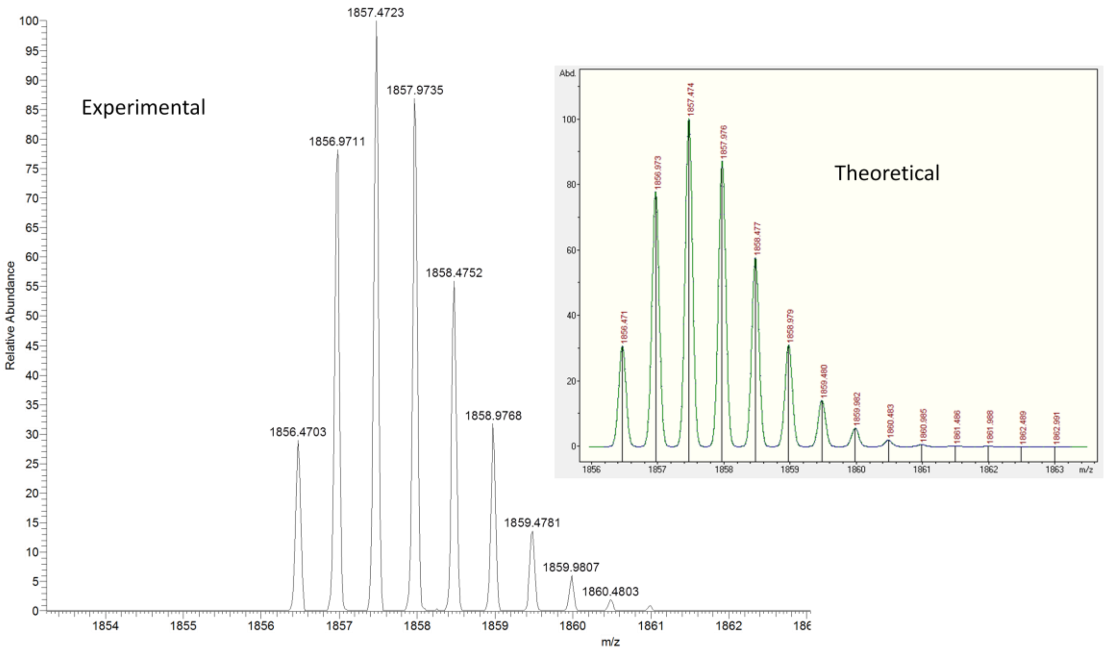

Supplementary Figure 129: Expanded HRMS ESI of G1 [3]Rotaxane Dendrimer of $[M–2PF_6]^{2+}$ ion.

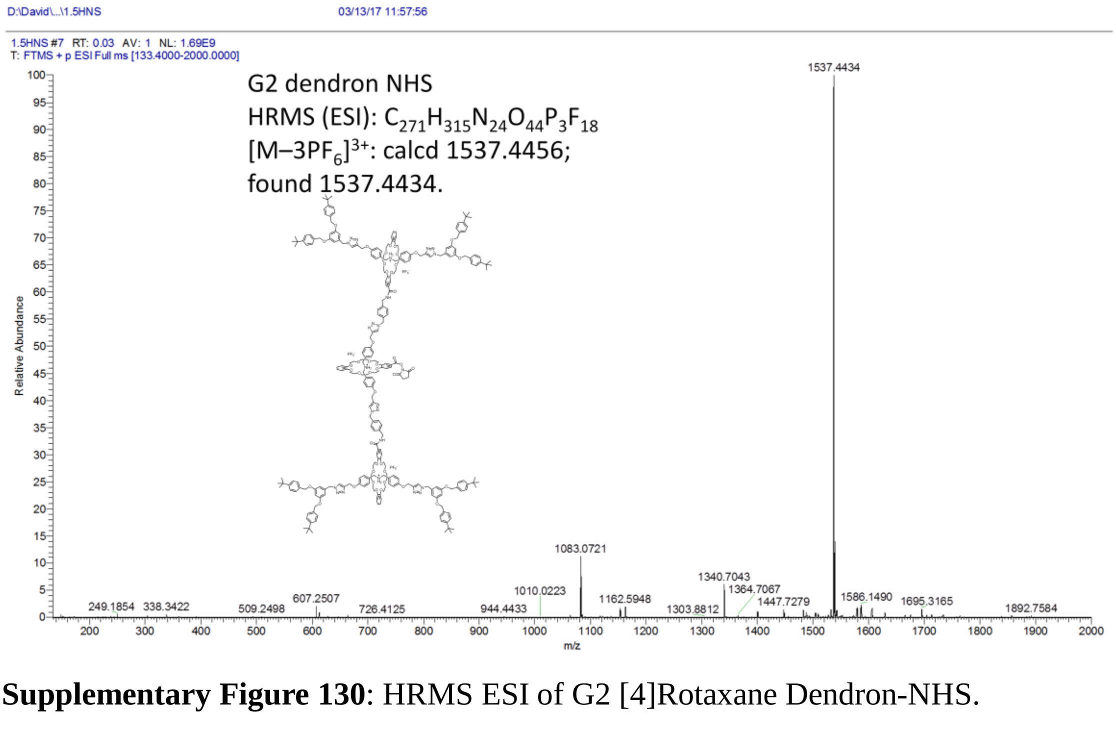

Supplementary Figure 130: HRMS ESI of G2 [4]Rotaxane Dendron-NHS.

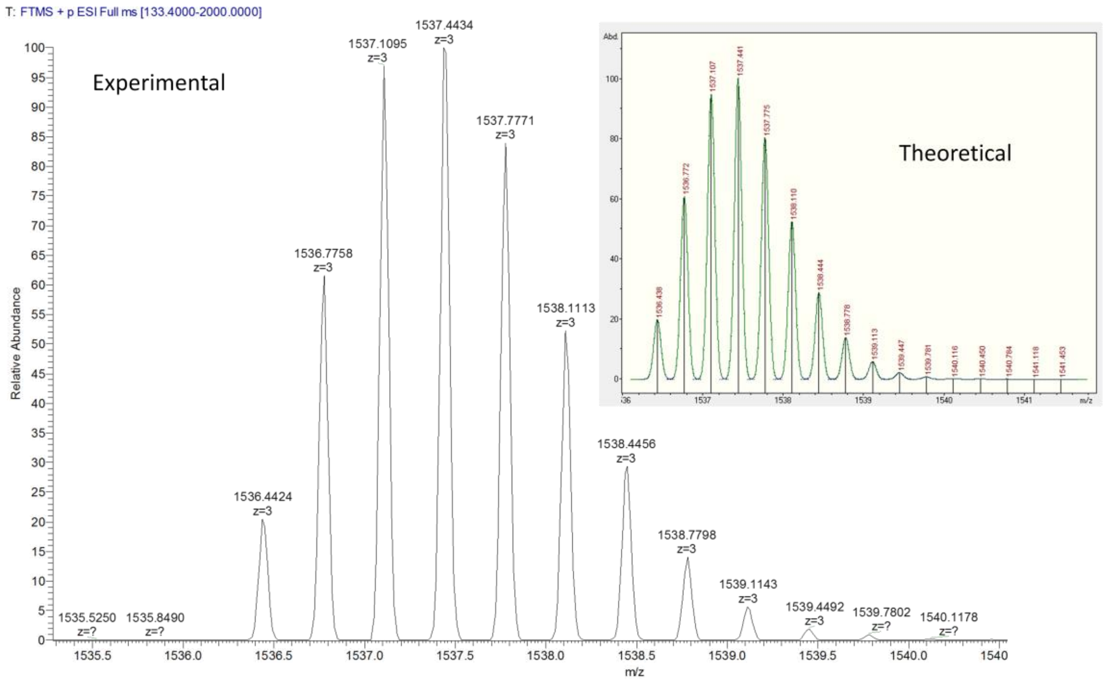

Supplementary Figure 131: Expanded HRMS ESI of G2 [4]Rotaxane Dendron-NHS of $[M{-}3PF_6]^{3+}$ ion.

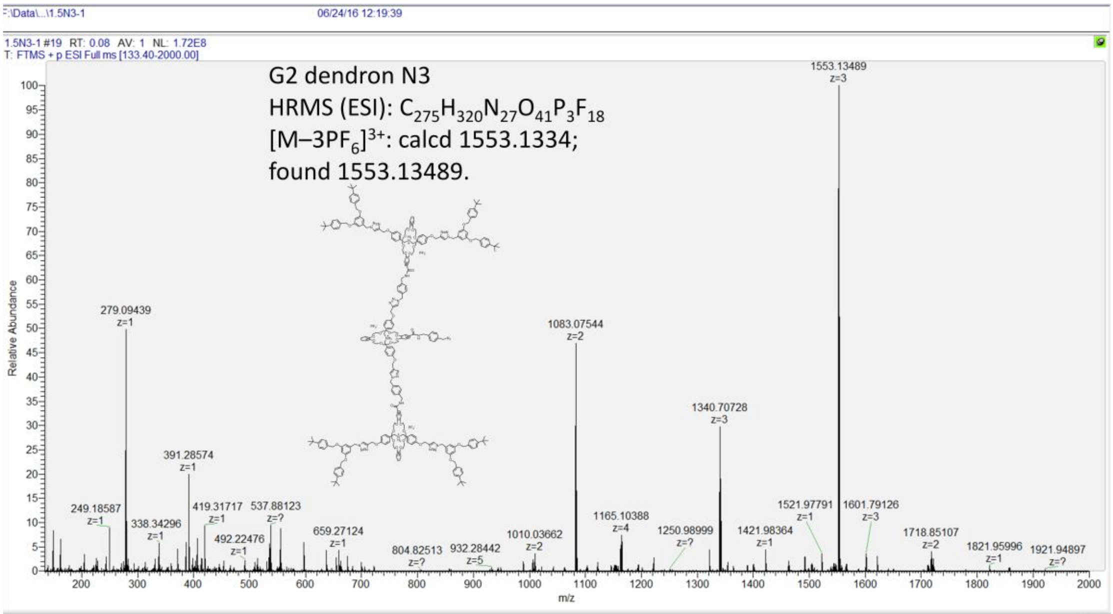

Supplementary Figure 132: HRMS ESI of G2 [4]Rotaxane Dendron-Azide.

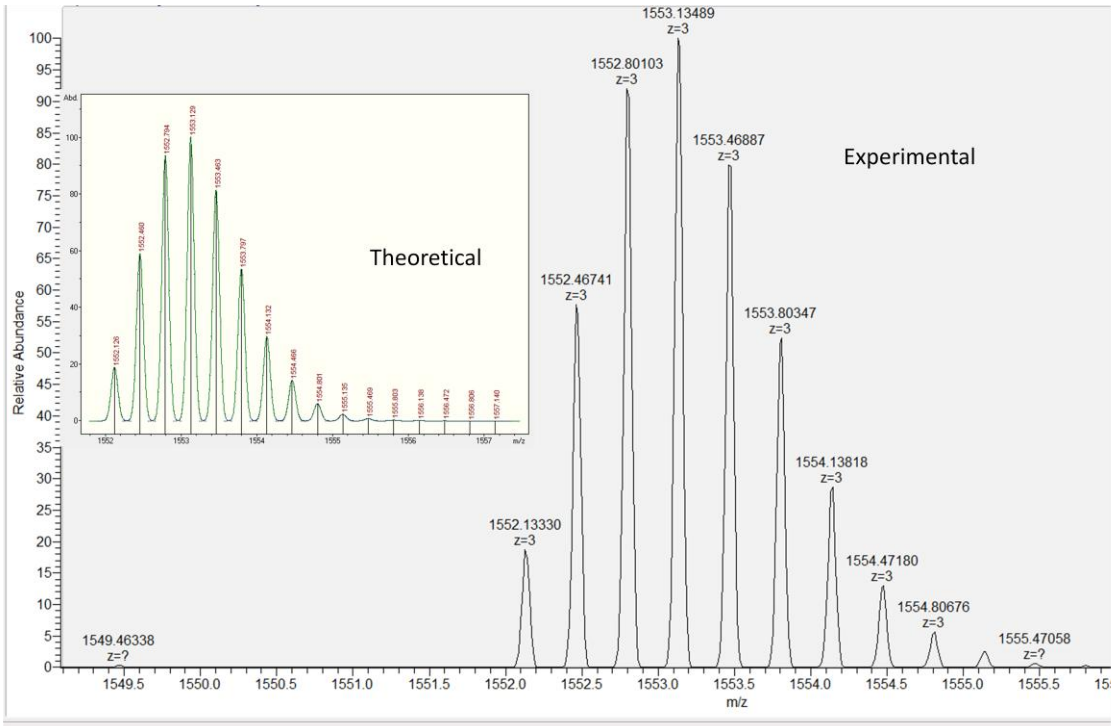

Supplementary Figure 133: Expanded HRMS ESI of G2 [4]Rotaxane Dendron-Azide of $[M–3PF_6]^{3+}$ ion.

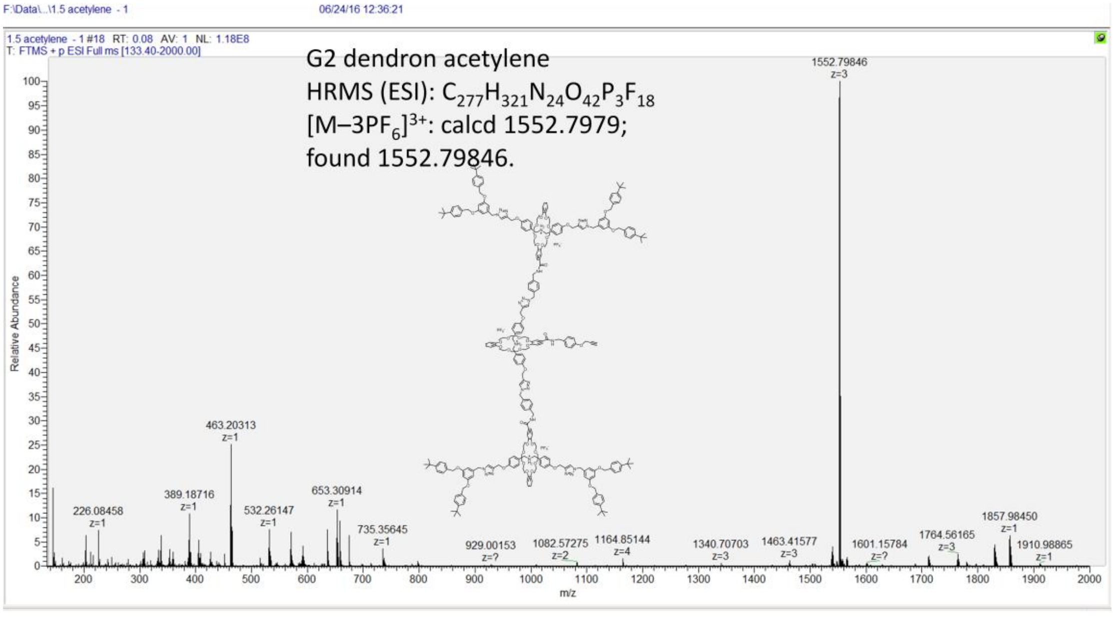

Supplementary Figure 134: HRMS ESI of G2 [4]Rotaxane Dendron-Acetylene.

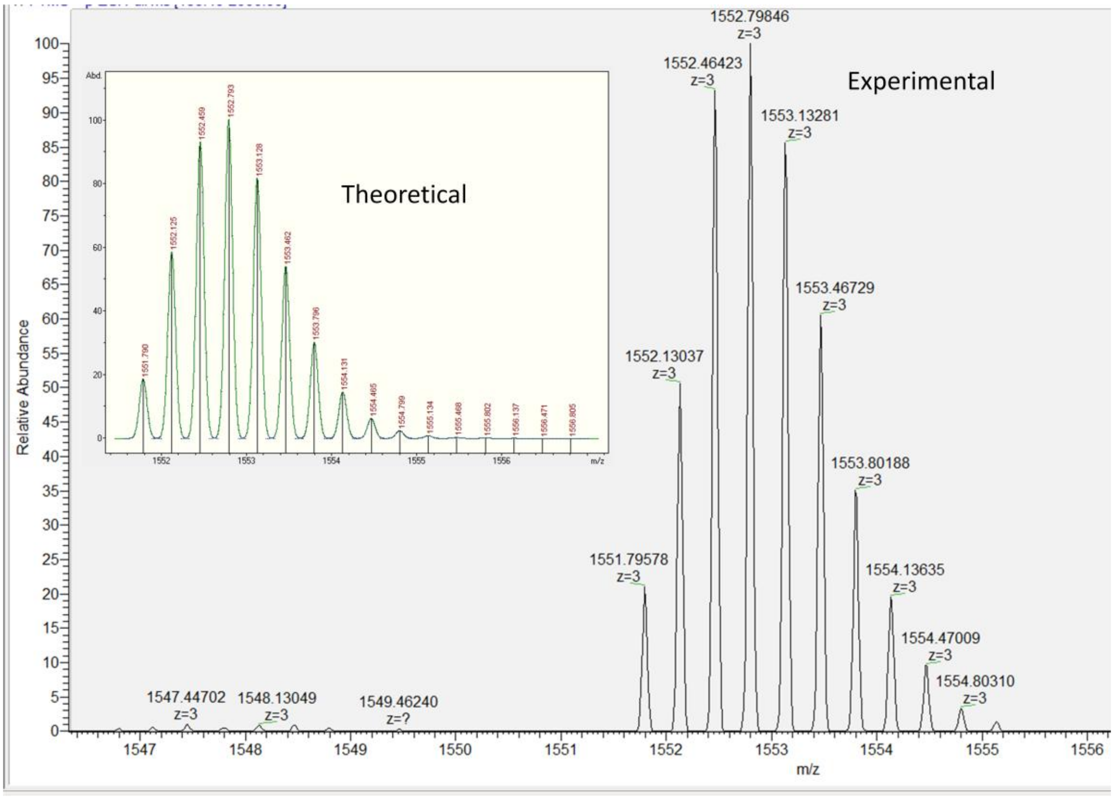

Supplementary Figure 135: Expanded HRMS ESI of G2 [4]Rotaxane Dendron-Acetylene of $[M{-}3PF_6]^{3+}$ ion.

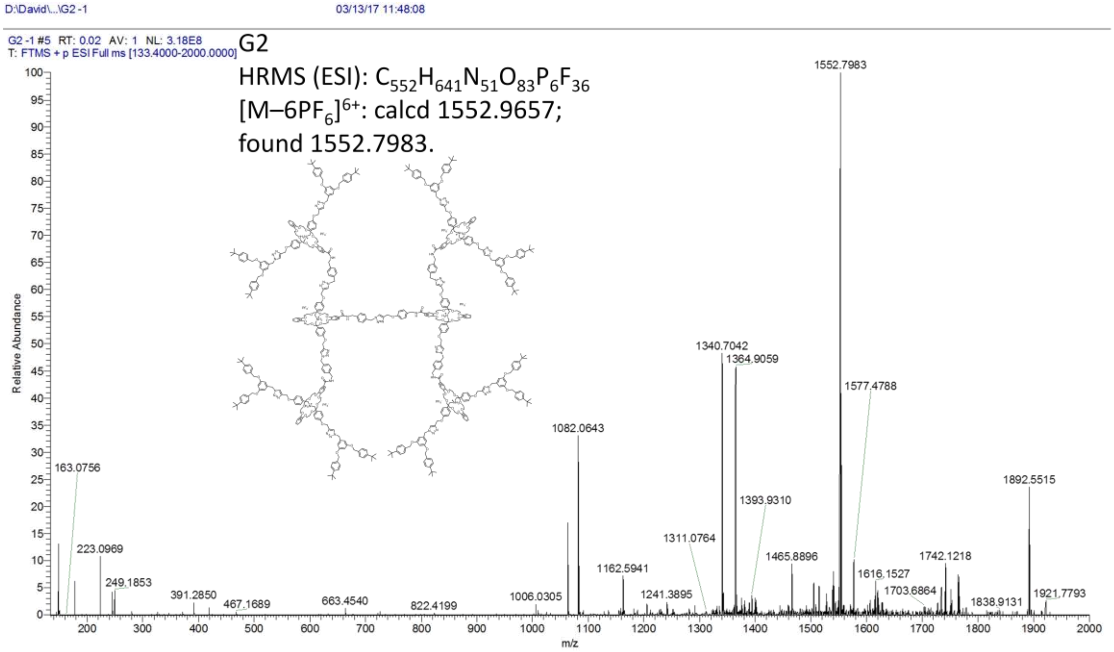

Supplementary Figure 136: HRMS ESI of G2 [7]Rotaxane Dendrimer.

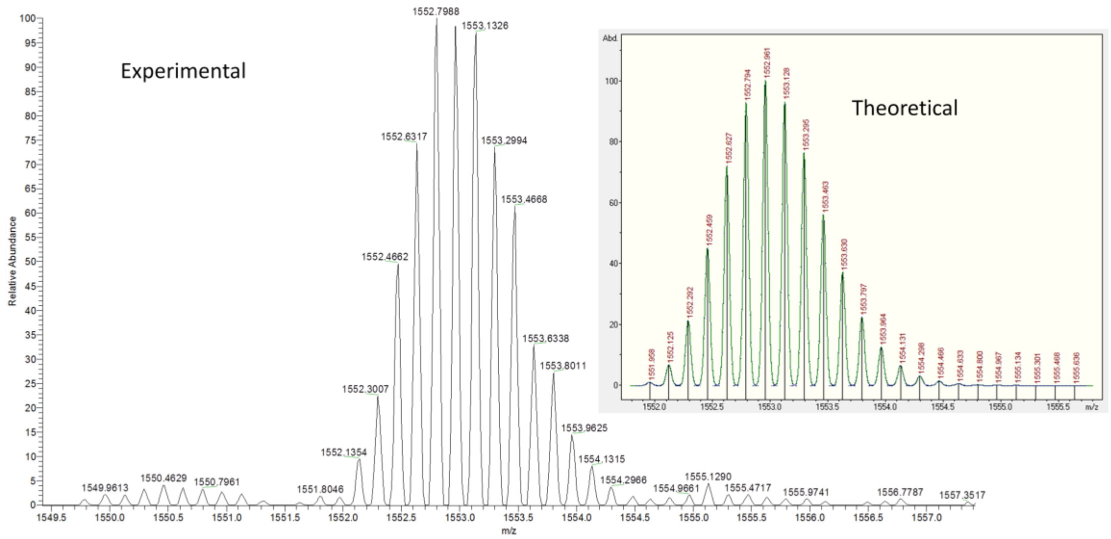

Supplementary Figure 137: Expanded HRMS ESI of G2 [7]Rotaxane Dendrimer of $[M–6PF_6]^{6+}$ ion.

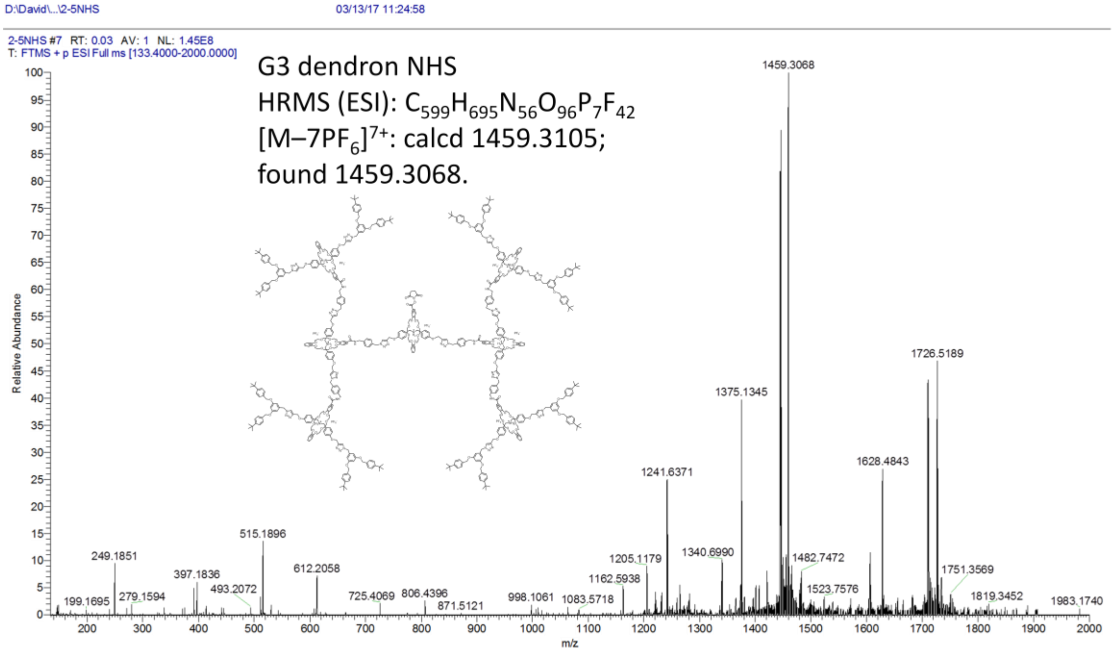

Supplementary Figure 138: HRMS ESI of G3 [8]Rotaxane Dendron-NHS.

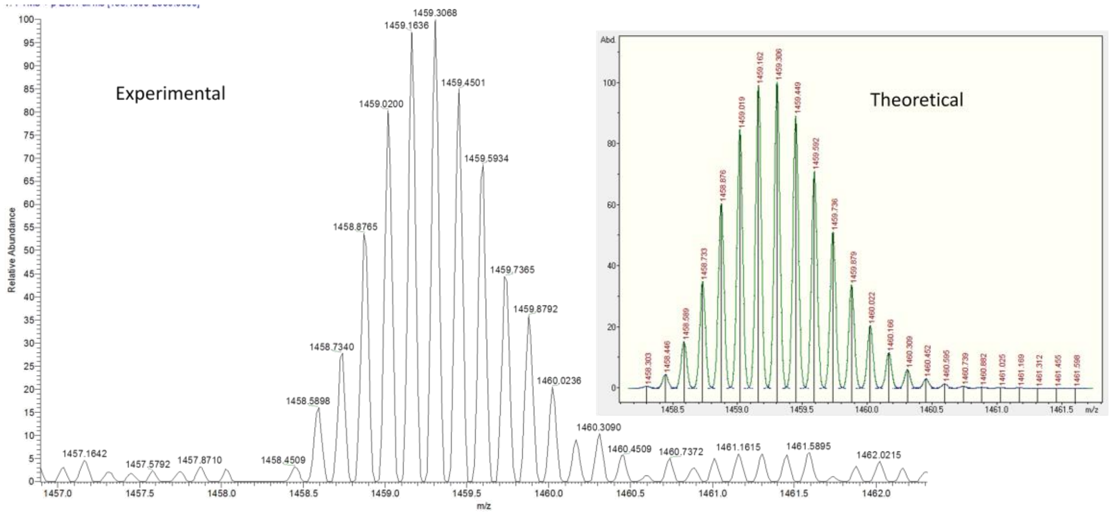

Supplementary Figure 139: Expanded HRMS ESI of G3 [8]Rotaxane Dendron-NHS of $[M{-}7PF_6]^{7+}$ ion.

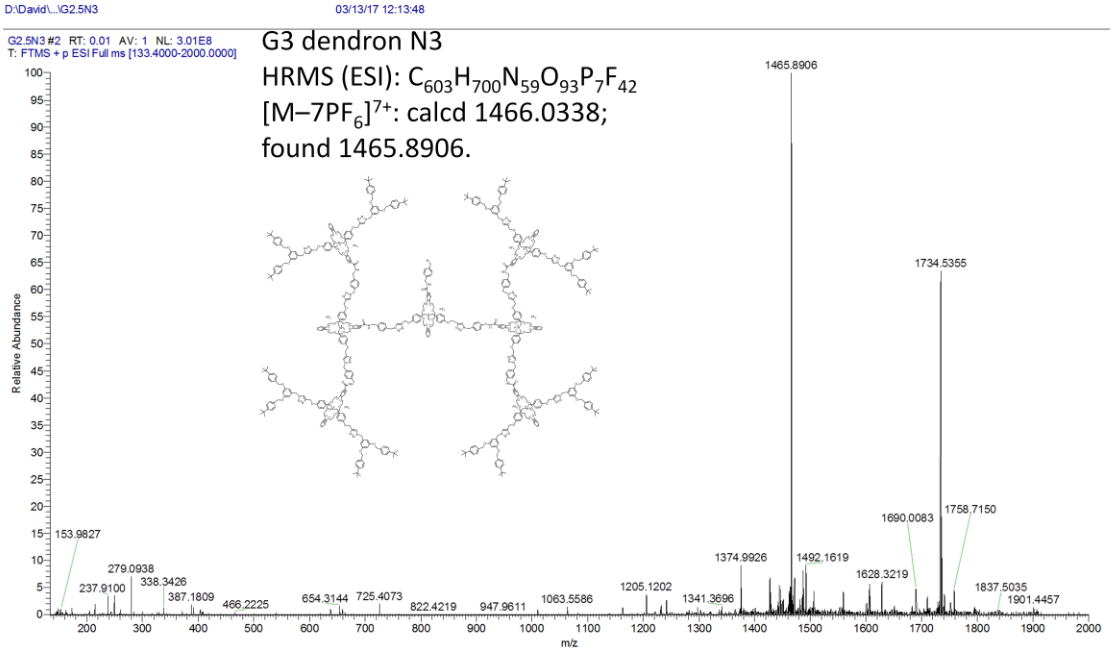

Supplementary Figure 140: HRMS ESI of G3 [8]Rotaxane Dendron-Azide.

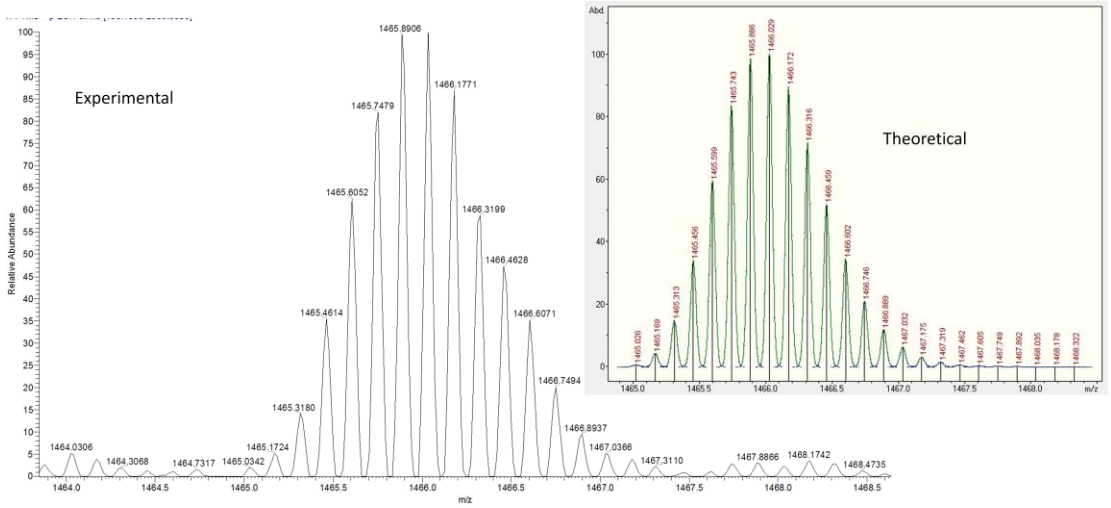

Supplementary Figure 141: Expanded HRMS ESI of G3 [8]Rotaxane Dendron-Azide of $[M–7PF_6]^{7+}$ ion.

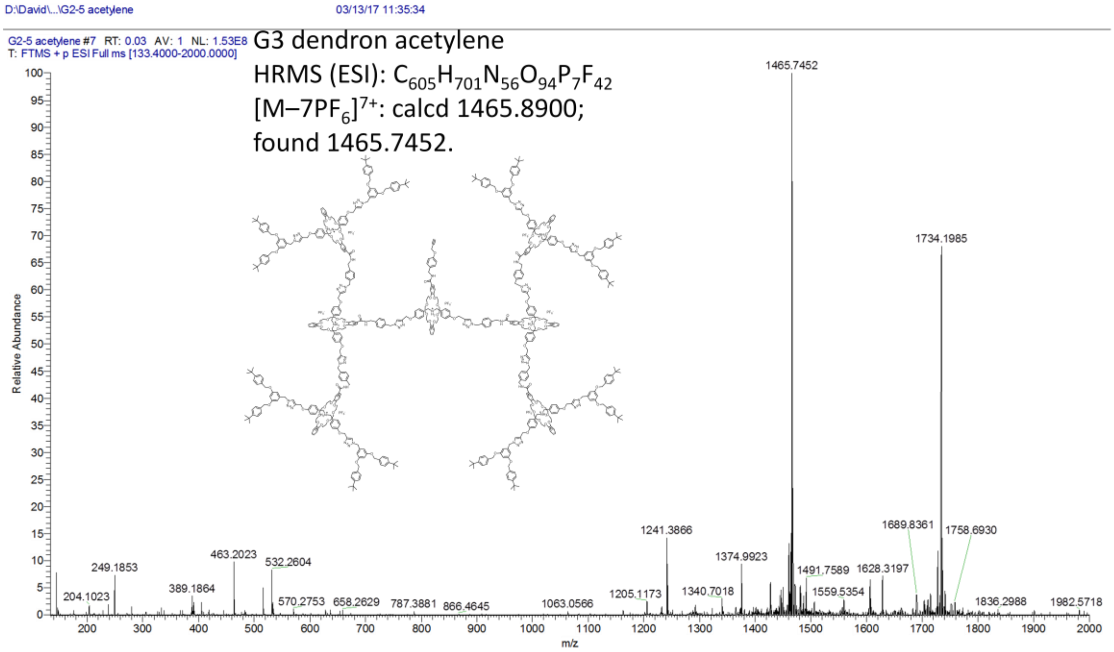

Supplementary Figure 142: HRMS ESI of G3 [8]Rotaxane Dendron-Acetylene.

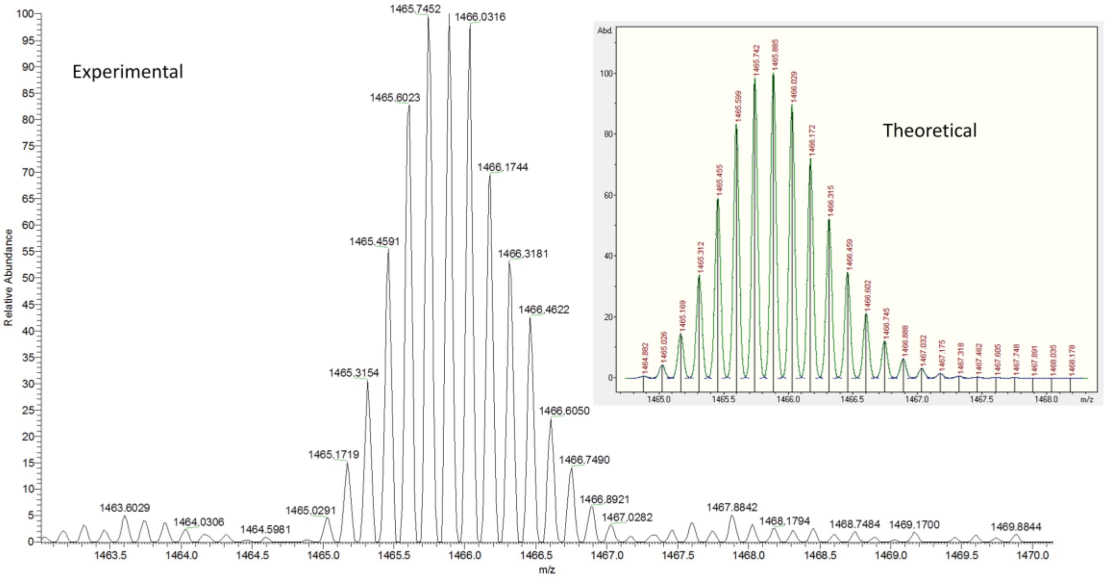

Supplementary Figure 143: Expanded HRMS ESI of G3 [8]Rotaxane Dendron-Acetylene of $[M{-}7PF_6]^{7+}$ ion.

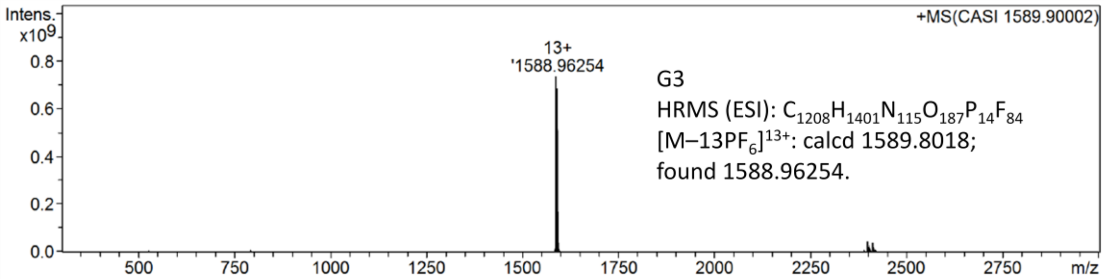

Supplementary Figure 144: HRMS ESI of G3 [15]Rotaxane Dendrimer.

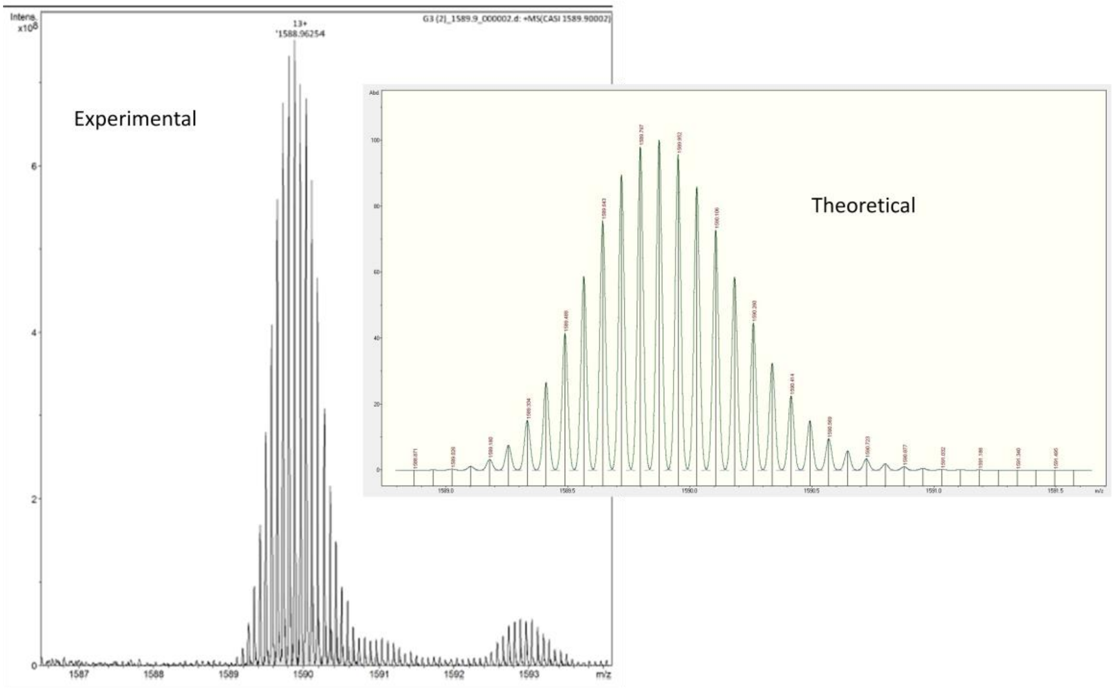

Supplementary Figure 145: Expanded HRMS ESI of G3 [15]Rotaxane Dendrimer of $[M{-}13PF_6]^{13+}$ ion.

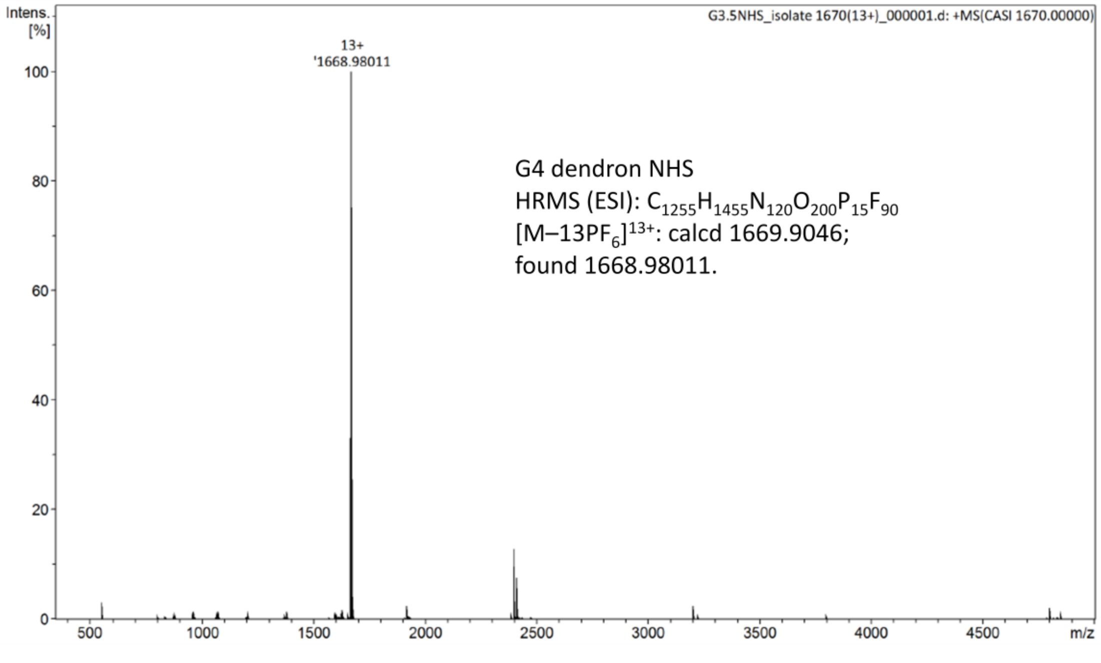

Supplementary Figure 146: HRMS ESI of G4 [16]Rotaxane Dendron-NHS.

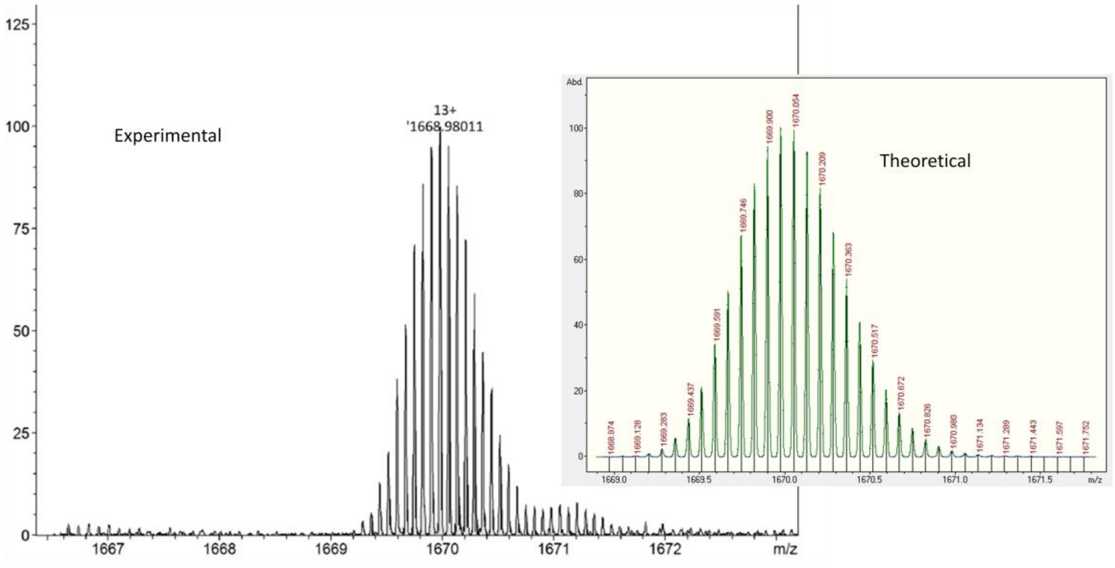

Supplementary Figure 147: Expanded HRMS ESI of G4 [16]Rotaxane Dendron-NHS of $[M–13PF_6]^{13+}$ ion.

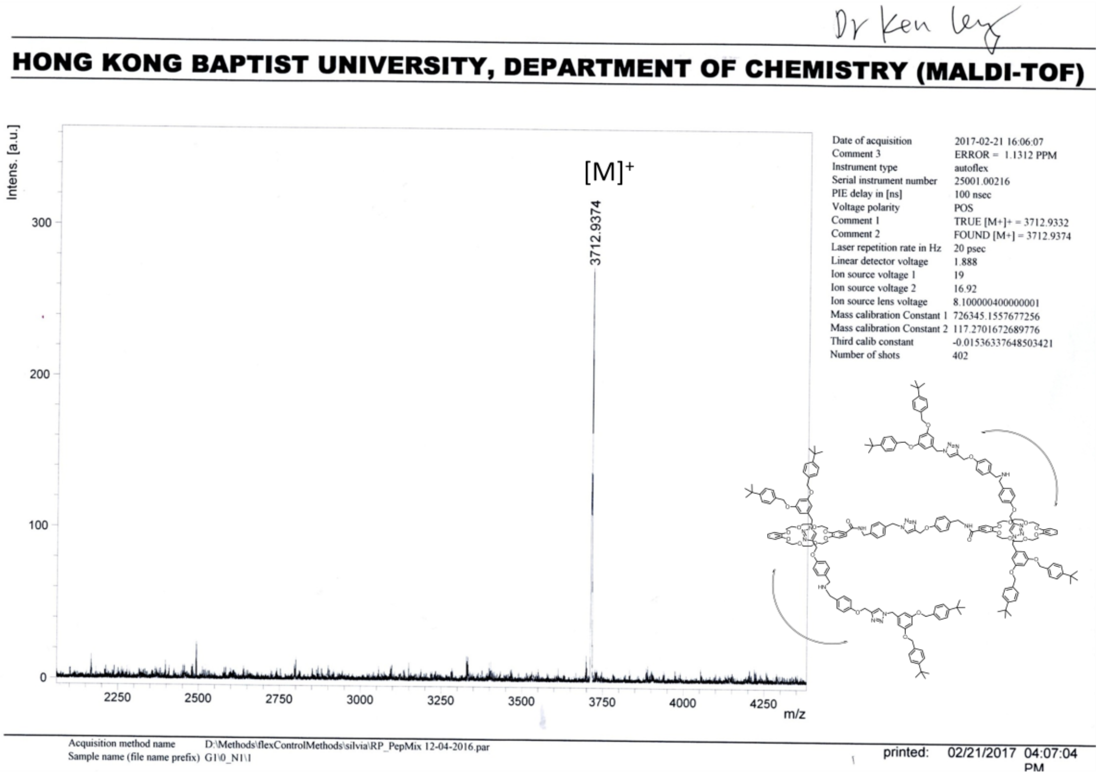

Supplementary Figure 148: HRMS MALDI-TOF of Neutral G1 [2]Rotaxane Dendrimer

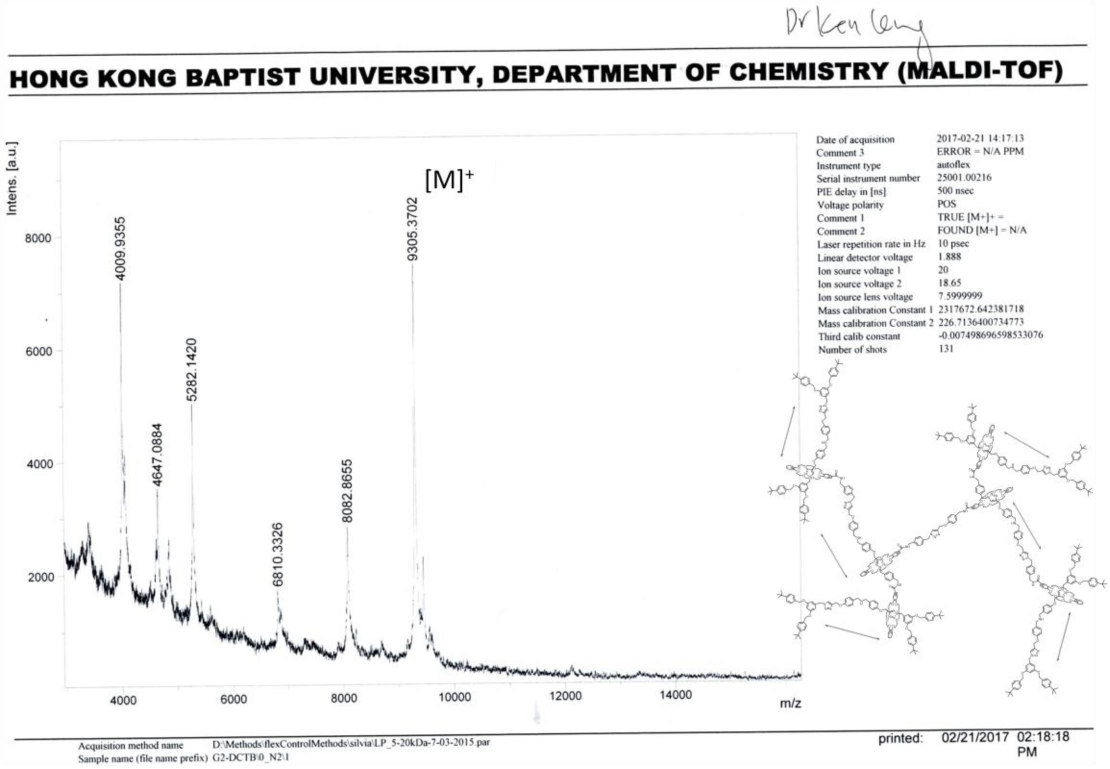

Supplementary Figure 149: HRMS MALDI-TOF of Neutral G2 [7]Rotaxane Dendrimer

Supplementary References

1. Jones, J. W.; Bryant, W. S.; Bosman, A. W.; Janssen, A. J.; Meijer, E. W.; Gibson, H. W. Crowned Dendrimers: pH-Responsive Pseudorotaxane Formation. *J. Org. Chem.* **68**, 2385–2389 (2003).
2. Zhang, Z.-J.; Zhang, H.-Y.; Wang, H.; Liu, Y. A Twin-Axial Hetero[7]rotaxane. *Angew. Chem. Int. Ed.* **50**, 10834–10838 (2011).
3. Iqbal, P. *et al.* Surface Molecular Tailoring Using pH-Switchable Supramolecular Dendron-Ligand. Assemblies *ACS Appl. Mater. Interfaces* **6**, 6264–6274 (2014).
4. Lau, K.-N.; Chow, H.-F.; Chan, M.-C.; Wong, K.-W. Dendronized Polymer Organogels from Click Chemistry: A Remarkable Gelation Property Owing to Synergistic Functional-Group Binding and Dendritic Size Effects. *Angew. Chem. Int. Ed.* **47**, 6912–6916 (2008).
5. Ho, W. K.-W. *et al.* Type III-B rotaxane dendrimers. *Chem. Commun.* **49**, 10781–10783 (2013).
6. Aradi, B.; Hourahine, B.; Frauenheim, T. DFTB+, a sparse matrix-based implementation of the DFTB method. *J Phys. Chem. A* **111**, 5678–5684 (2007).
7. Frauenheim, T. *et al.* Atomistic simulations of complex materials: groundstate and excited-state properties. *J. Phys. Cond. Matter* **14**, 3015–3047 (2002).
8. Zhechkov, L. Heine, T.; Patchkovskii, S.; Seifert, G.; Duarte, H. A. An efficient a posteriori treatment for dispersion interaction in density-functional-based tight binding. *J. Chem. Theory Comput.* **1**, 841–847 (2005).
9. Wang, M.; Gong, X.; Hu, J.; Yu, Y.; Chen, Q.; Cheng, Y. Understanding the Binding Interactions between Dendrimer and 18 Common Amino Acids by NMR Techniques. *J. Phys. Chem. B* **115**, 12728–12735 (2011).
10. Müller, T. *et al* AFM Studies of High-Generation PAMAM Dendrimers at the Liquid/Solid Interface. *Langmuir* **18**, 7452–7455 (2002).
11. Thordarson, P. Determining association constants from titration experiments in supramolecular chemistry. *Chem. Soc. Rev.* **40**, 1305 –1323 (2011).
